# An Energy Model Based on Molecular Structure for Predicting Histone Modification Levels at lncRNA Promoter Regions in HepG2 Cells

**DOI:** 10.3390/ijms27135653

**Published:** 2026-06-23

**Authors:** Menglan Li, Yingli Chen, Qianzhong Li, Pengyu Du, Dimeng Zhang, Yuanyuan Zhao

**Affiliations:** 1 Inner Mongolia Autonomous Region Key Laboratory of Biophysics and Bioinformatics, School of Physical Science and Technology, Inner Mongolia University, Hohhot 010021, China; 2 State Key Laboratory of Reproductive Regulation and Breeding of Grassland Livestock, Inner Mongolia University, Hohhot 010021, China

**Keywords:** hepatocellular carcinoma, long non-coding RNA, histone modification, statistical physics model, interaction energy

## Abstract

In hepatocellular carcinoma (HepG2), aberrant histone modifications are closely linked to long non-coding RNA (*lncRNA*) expression. However, existing computational models lack physical interpretability at specific promoter coordinates. To address this, we developed a position-specific statistical scoring model based on adjacent and next-adjacent nucleotide frequencies. We trained two independent, position-specific matrices representing increased and decreased modification states across 600 bp promoter windows centered on the true signal summits. Finally, ten-fold cross-validation revealed that significant energy differences between sequences with increased and decreased histone signals enable excellent classification performance. These results indicted a strong correlation between the total energy of local DNA structures and histone modification signal.

## 1. Introduction

In hepatocellular carcinoma (HepG2), aberrant histone modifications are linked to long non-coding RNA (*lncRNA*) dysregulation.

HepG2 is one of the leading causes of cancer death in the world, and its development is a complex process involving dysregulated gene expression [1,2]. More and more evidence has shown that *lncRNA* are aberrantly expressed in HepG2, participating in key oncogenic processes including proliferation, invasion, metastasis, and drug resistance [3,4,5]. Recently, research on *lncRNA*s in cancer has rapidly expanded into new areas such as neutrophil extracellular traps (NETs) and various modes of programmed cell death like ferroptosis. These advancements provide significant clinical value for enhancing prognosis assessment and biomarker development [6,7]. In addition to these approaches, the dysregulated expression of *lncRNA* in HepG2 is closely associated with epigenetic modifications in their promoter regions, in which histone modifications play a particularly critical role [8,9]. Therefore, analyzing the changes of abnormal histone modifications in the promoter regions of *lncRNA* is crucial for finding the underlying mechanisms of HepG2 pathogenesis [10].

In HepG2, the patterns of histone modifications are often significantly different from those of normal hepatocytes [11,12]. A number of studies have demonstrated these alterations at multiple levels. First, at the protein-coding gene level, it has been found that signals of activating modifications such as H3K4me3 and H3K27ac are aberrantly enhanced in the promoter regions of oncogenes like MYC and CCND1, while repressive modifications like H3K27me3 and H3K9me3 are related to the silencing of tumor suppressor genes [13,14,15]. Second, in the non-coding RNA level, similar modification patterns regulate the expression levels of oncogenic *lncRNA* (e.g., MALAT1) and tumor-suppressive *lncRNA* (e.g., DAW), directly affecting tumor proliferation and metastasis [16,17]. Additionally, recent research has highlighted the coregulatory effects of multiple histone modifications on key ferroptosis-related genes, emphasizing the complexity of epigenetic modifications [18]. Furthermore, recent studies have revealed that aberrant histone acetylation in HepG2 can enhance the expression of specific m6A-related *lncRNA*, which act as signaling molecules to further cooperate with key pathways such as Wnt/β-catenin, jointly influencing immune response and therapeutic resistance [19,20]. Finally, in clinical applications, these abnormal modification patterns have been verified as key signals for assessing HepG2 subtypes and predicting patient prognosis and diagnosis [21,22,23]. In summary, these findings highlight the great potential of histone modifications and *lncRNA*s as biomarkers and potential therapeutic targets for cancer [24,25].

Although current experimental techniques can effectively reveal the distribution of histone modifications or observe their effects on chromatin structure and gene expression [26,27,28], these methods still face significant limitations. On the technical level, approaches such as Chromatin Immunoprecipitation Sequencing (Chip-seq) and enzyme inhibitor treatments are often costly and time-consuming, while High-throughput Chromatin Conformation Capture (Hi-C) technology struggles to resolve the fine local structure of DNA over short distances. More importantly, at the mechanistic level, most of these experimental methods can only provide descriptive correlations and fail to delve into the physical level of molecular interaction [29].

To overcome the limitations of purely experimental approaches, a series of predictive algorithms have been developed in the field of bioinformatics [30,31,32,33,34,35,36,37]. For example, machine learning and deep learning models, such as those based on CNN or Transformer architectures, integrate multi-omics data including genomic sequences, chromatin accessibility, methylation, and histone modifications. These models have demonstrated good potential in identifying genome-wide histone modification regions, inferring the effects of histone modification signals on *lncRNA* expression, and evaluating the prognosis of HepG2 patients based on multiple epigenetic modification data.

However, a key issue exists with these mainstream computational methods. They tend to focus on using complex parameters to analyze the correlation between sequences and epigenetic modifications and their effects on gene expression; most of these works were based on analyzing the association between sequence features and epigenetic modifications, with no regard for the influence and interpretation of DNA’s physical properties, such as its structure. This lack of a physical perspective results in a critical limitation: while these models can accurately predict the impact, they fail to fundamentally explain the underlying physical reasons. This limitation highlights the necessity of constructing interpretable analytical models based on physical principles [38,39].

Therefore, from a physical perspective, identifying and understanding the histone modification signal levels in *lncRNA* promoter regions is crucial for studying the molecular origins of cancer development [40,41]. Based on the physical principle that molecular structure is determined by its energy, a statistical physics energy model using the interaction energy between adjacent dinucleotides has been proposed. This model has been successfully applied to the recognition of prokaryotic promoters, confirming an intrinsic correlation between the interaction energy of DNA segments and their biological functions [42].

To better understand the cooperative effects of adjacent bases on local DNA structure, this study aimed to develop an improved trinucleotide energy model by integrating bioinformatics and statistical physics. In this model, the total energy of a promoter region is computed as the sum of interaction energies between adjacent bases. Applying this model, we used the data of 11 key histone modifications in HepG2 to calculate the total energy of local structures in the promoter regions of differentially expressed *lncRNA* where modification signals were significantly increased or decreased. Excellent results were obtained, providing a new theoretical tool for understanding the changes in epigenetic signals from a physical perspective.

## 2. Results

### 2.1. Analysis of the lncRNA Differential Expression Profile

**Figure 1:**
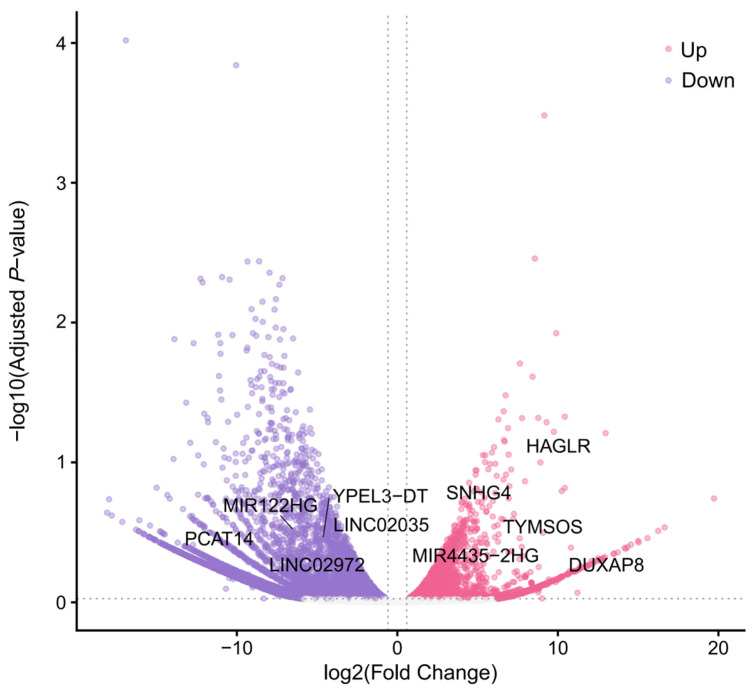
A volcano plot of differentially expressed *lncRNAs* (*DElncRNAs*). The x-axis displays the $\log_{2}\left(fold\;change\right)$, and the y-axis represents the statistical significance as −log10(adjusted *p*-value). The horizontal dashed line indicates the significance threshold (adjusted *p*-value = 0.05), while the vertical dashed lines represent the fold-change threshold (|$\log_{2}FC$| = 1). Compared to the normal group, points colored in pink represent significantly up-regulated *lncRNA*s in the tumor group, points in dark purple represent significantly down-regulated *lncRNA*s, and points in gray represent non-significantly expressed *lncRNA*s.

To identify *lncRNA* with significant expression changes in HepG2, we analyzed total RNA-seq data from the normal hepatocyte and HepG2 cell lines. From the annotation file, we first extracted a total of 13,880 *lncRNA*. Subsequently, by applying the thresholds of an adjusted *p*-value < 0.05 and |log_2_*FC*| > 1, we identified 930 significantly differentially expressed *lncRNAs* (*DElncRNAs*) (Figure 1).

### 2.2. Construction and Screening of Model Input Datasets

Following the identification of *DElncRNA* promoter regions, we analyzed the signals of 11 histone modifications within these regions and identified regions where these signals were significantly altered.

Finally, we constructed the positive sets (sequences from regions with significantly enhanced signals in *DElncRNA* promoters) and negative sets (sequences from regions with significantly weakened signals).

This is because gene up-regulation can be driven not only by the enrichment (increased) of activating marks (e.g., H3K4me3) but also by the depletion (decreased) of repressive marks (e.g., H3K27me3, known as the derepression effect). Enforcing a strict directional restriction would lead to the loss of biologically significant samples (e.g., repressive mark depletion leading to gene activation). Therefore, we used differentially expressed (both up and down) *lncRNAs* as our target genes, and captured both increased and decreased histone modification regions within their promoters.

### 2.3. Energy Values and Energy Differences in Positive and Negative Sample Sets

To visually analyze how the energy model distinguishes between *lncRNA* promoters with increased (positive set) and decreased (negative set) modification levels in HepG2, we plotted the distribution of energy values and the energy difference (ΔE) for the eleven histone modifications. The analysis was performed on the test sets in a 10-fold cross-validation.

Energy analysis revealed a consistent pattern across all eleven histone modifications: sequences from regions with increased signals exhibited significantly lower energy values than those from regions with decreased signals. Consequently, the Δ*E* values for the positive set were consistently negative, which means the model can effectively distinguish between the two states.

**Figure 2:**
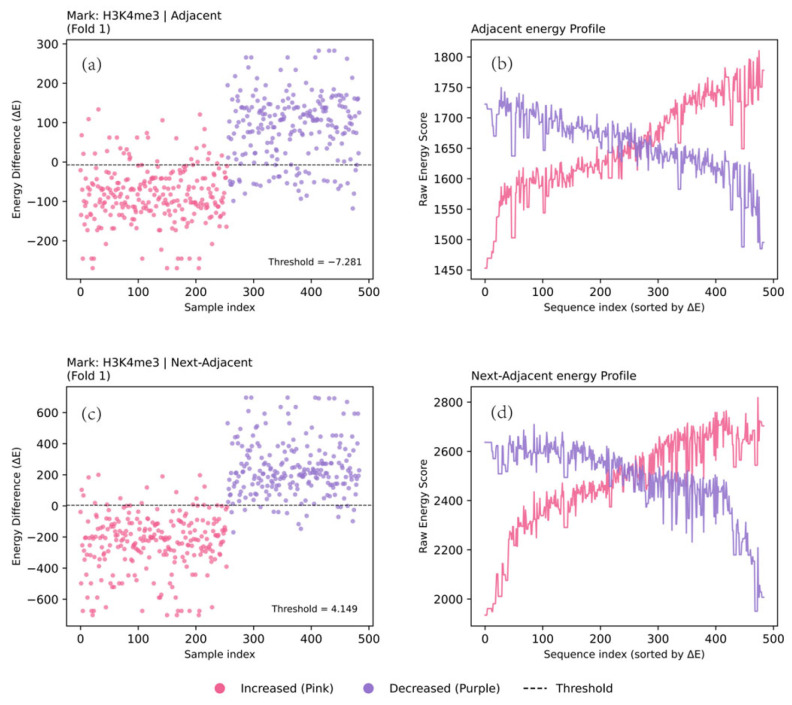
Comparison of dinucleotide and trinucleotide models for discriminating H3K4me3c signals. (**a**,**b**) The results of the dinucleotide model for a representative 10-fold cross-validation fold. (**c**,**d**) The results of the trinucleotide model for a representative 10-fold cross-validation fold. (**a**,**c**) The distribution of the energy difference (ΔE) for each sequence in the test sets, where the horizontal dashed line indicates the classification threshold. (**b**,**d**) The score curves of the positive ($E_{positive}$) and negative ($E_{negative}$) energies for the test sequences sorted by their ΔE values, providing the energy profile.

We illustrate these findings using four key modifications as examples: H3K4me3, H3K27ac, H3K27me3, and H3K36me3. To validate the model’s effectiveness, we first analyzed the energy distribution features for H3K4me3 (Figure 2).

The energy difference (ΔE) distribution plots (Figure 2a,c) showed that points representing increased samples (pink) were predominantly clustered in the negative region of the ΔE axis, whereas decreased samples (purple) were mainly distributed in the positive region. This trend was further confirmed in the energy plots (Figure 2b,d), where the energy curve for the positive model ($E_{positive}$, pink) was globally lower than that of the negative model ($E_{negative}$, purple) for sequences with lower ΔE values, clearly demonstrating the model’s discriminative ability.

Similarly, other key modifications such as H3K27ac (Figure 3), H3K27me3 (Figure 4), and H3K36me3 (Figure 5) exhibited highly similar patterns in their energy and energy difference distributions (see Supplementary Figures S1–S11 for the detailed cross‑validation results of all 11 histone modifications).

### 2.4. Performance Comparison of Trinucleotide and Dinucleotide Models

We next systematically evaluated the model’s performance using a 10-fold cross-validation procedure. This analysis confirmed that the model could effectively distinguish between *lncRNA* promoters with increased and decreased histone modification levels in HepG2. For all eight modifications tested, the energy difference distributions between the positive and negative sets were significantly different.

**Figure 5:**
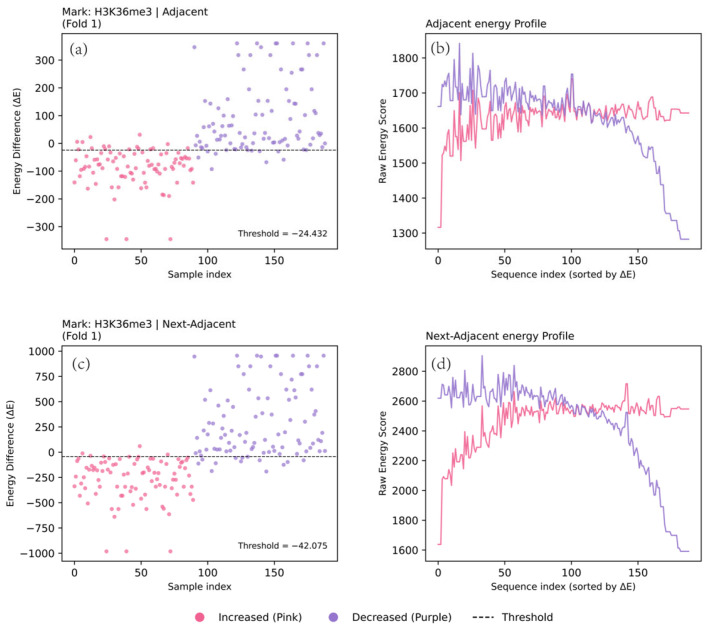
Comparison of dinucleotide and trinucleotide models for discriminating H3K36me3 signals. (**a**,**b**) The results of the dinucleotide model for a representative 10-fold cross-validation fold. (**c**,**d**) The results of the trinucleotide model for a representative 10-fold cross-validation fold. (**a**,**c**) The distribution of the energy difference (ΔE) for each sequence in the test sets, where the horizontal dashed line indicates the classification threshold. (**b**,**d**) The score curves of the positive ($E_{positive}$) and negative ($E_{negative}$) energies for the test sequences sorted by their ΔE values, providing the energy profile.

The adjacent model performed well across all eleven histone modifications, achieving an average MCC of 0.718 (Table 1, Table 2). However, the next-adjacent (trinucleotide) energy model consistently outperformed the adjacent model for every modification (Table 1, Table 2, Figure 5). With the next-adjacent model, the average MCC increased to 0.785 (ranging from 0.772 to 0.852). These results suggest that considering next-adjacent base dependencies captures additional sequence-dependent local structural features.

## 3. Discussion

The results of this study demonstrate that the local structural energy of DNA sequences can effectively distinguish between *lncRNA* promoter regions with increased and decreased histone modification signals in HepG2.

In this study, we constructed and validated a statistical physics model based on trinucleotide interaction energies to investigate the physical basis of histone modification signal changes associated with *lncRNA* expression in HepG2. The results confirm that the local interaction energy of DNA is an effective physical parameter for discriminating between *lncRNA* promoter regions with increased and decreased modification signals.

In contrast to deep learning models that optimize parameters, our approach provides position-specific sequence scoring model based on frequencies. However, this statistical approach does not identify biological protein-binding motifs (such as TF consensus sequences) or resolve three-dimensional DNA structures or complex biological pathways. Instead, it shows how nucleotide frequencies vary across different promoter positions and links these frequency variations to changes in histone modification signals. Our findings also show that the trinucleotide model is better at distinguishing the two groups than the dinucleotide model. It is likely that the DNA structural changes are influenced by the synergistic effects of adjacent bases. A dinucleotide interaction model may not adequately capture these local structural features, which are dependent on a longer sequence context. By incorporating information from more extensive neighboring interactions, the trinucleotide model more sensitively detects local DNA structural alterations associated with histone modifications, thereby more accurately reflecting the energy differences between distinct modification levels.

In summary, the results of this study show that a statistical physics model based on trinucleotide interaction energies can effectively identify DNA sequences associated with different histone modification levels. This model not only provides a new theoretical tool for understanding epigenetic regulation in HepG2 from a physical perspective but also demonstrates that the local structural energy of DNA could serve as a potential biomarker, offering new directions for future cancer diagnosis and treatment.

## 4. Materials and Methods

### 4.1. Data Acquisition and Pre-Processing

The raw count data from RNA-sequencing (RNA-seq) and the Chip-seq data for 11 key histone modifications (H3K4me1, H3K4me2, H3K4me3, H3K9me3, H3K27me3, H3K36me3, H3K79me2, H4K20me1, H3K9ac, H3K27ac, and H2AFZ) for the HepG2 cell line and normal hepatocytes were downloaded from the ENCODE project (ENCODE; https://www.encodeproject.org/, accessed on 5 August 2025). The genome annotation and *lncRNA* coordinates (GRCh38 version, Release 49) were sourced from the GENCODE database (GENCODE; https://www.gencodegenes.org/human/, accessed on 5 August 2025). All genomic coordinates were standardized to the hg38 reference genome.

Modifications: All raw data and processed files from ENCODE, including ChIP-seq and RNA-seq, are detailed with their respective Accession IDs (ENCFF…) in Table S1 of the Supplementary Material.

### 4.2. Differential Expression Analysis of lncRNAs

To identify differentially expressed *lncRNAs* in the HepG2 cell line, we performed differential expression analysis on the RNA-seq data using the Deseq2 package [43]. *LncRNAs* were considered significantly differentially expressed if they met the criteria of an adjusted *p*-value (FDR) < 0.05 and $\left|\log_{2}FC\right|$ > 1.

### 4.3. Identification of Differential Histone Modification Regions

To accurately identify *lncRNA* promoter regions with differential histone modification signals between the HepG2 cell line and the normal control (hepatocyte), we developed a custom quantitative differential framework tailored to the biophysical occupancy profiles of different modifications. For each of the 11 candidate histone marks, we utilized the official biological replicate-validated peak files (narrowPeak.gz and broadPeak.gz) and depth-normalized, control-subtracted SignalPval tracks (bigwig format) downloaded from ENCODE.

The quantitative signals were extracted across the defined 3000 bp *lncRNA* promoter regions (TSS −2000/+1000 bp). To filter out background noise, we enforced a statistical significance threshold, requiring that the signal intensity within the target region must reach a minimum of −lg(p-value)≥1.301 (corresponding to a single-point statistical test of p<0.05).

The differential status for each candidate promoter region was evaluated using the $\log_{2}\left(\mathrm{FoldChange}\right)$ of the chromatin signals:

(1) $$\log_{2}FC=\log_{2}\left(\frac{{\mathrm{Signal}}_{HepG2,i}+\beta }{{\mathrm{Signal}}_{Hepa,i}+\beta }\right)$$

where ${\mathrm{Signal}}_{HepG2,i}$ and ${\mathrm{Signal}}_{Hepa,i}$ represent the signal intensities of region i in the HepG2 and hepatocyte cell lines, respectively. Parameter β is a background-stabilizing pseudocount set to 0.01 to prevent false positive inflation on low-intensity background signals.

Considering the spatial distribution differences of the 11 modifications, the quantitative metrics and differential thresholds were defined as follows:

For narrow marks (H3K4me3, H3K27ac, H3K9ac, H3K4me2, and H2AFZ), we extracted the Maximum Signal Intensity (Max) within the promoter window, setting the differential enrichment threshold at $\log_{2}FC>0.58$ (approximately a 1.5-fold change).

For broad marks (H3K27me3, H3K36me3, H3K9me3, H3K79me2, H4K20me1, and H3K4me1), we calculated the Sum of Signals (Integral) across the promoter window, setting a broader differential threshold at $\log_{2}FC>0.38$ (approximately a 1.3-fold change) to capture the cumulative dosage effects.

Furthermore, to capture binary ‘on/off’ switches (presence/absence), any promoter region exhibiting a robust signal (intensity > 5.0) in one group and minimal background signal (intensity < 0.5) in the other was directly classified as increased or decreased, bypassing the strict fold-change threshold.

### 4.4. Identification of Differential Histone Modification Regions Within lncRNA Promoters

To focus on key functional regions directly associated with transcriptional regulation, the core promoter regions for each differentially *DElncRNAs* was defined as the interval spanning from 2000 bp upstream to 1000 bp downstream of its transcription start site (TSS). Subsequently, to identify the differential histone modification peaks located within these promoters, only those peaks that spatially overlapped with the defined promoter regions were retained for further analysis.

### 4.5. Construction of DNA Sequence Fragments

To prepare a standardized input dataset for the energy model, we extracted DNA sequence fragments of a uniform length (600 bp) from the differential histone modification regions within the *lncRNA* promoters identified previously. For narrow peaks, the summit position (point of maximum signal) was selected as the center. For broad peaks, we calculated and used the geometric center of the peak region. The final 600 bp DNA fragments were obtained by extending 300 bp upstream and 300 bp downstream from each defined anchor point (i.e., summit or geometric center).

A 600 bp window was selected because it appropriately covers a complete nucleosome (~147 bp) and its adjacent linker DNA (20–90 bp). This length represents a typical spatial context for the interplay of histone modifications and better captures the features of local DNA structure.

### 4.6. Physical Principles of Energy Model

Based on the fundamental biological principle that ‘structure determines function,’ we supposed that changes in histone modification levels directly influence local DNA structure, thereby affecting gene transcription. Compared to normal hepatocytes, the histone modification signals in promoter regions were significantly increased or decreased in HepG2. Due to their distinct biological functions, these chromatin regions are hypothesized to possess different physical structures. Therefore, it was proposed that the interaction energies should be different for distinct histone modification levels. As the total energy is the sum of the interaction energies of these units across all sites, the total local structural energy of the DNA must be different between these two groups of regions.

### 4.7. Construction of Model

To prepare for the energy model analysis, we first constructed an independent input dataset for each histone modification. The sequences were partitioned into two classes based on their signal intensity changes in HepG2 cells: (1) positive set, consisting of sequences regions with significantly enhanced histone modification signals; and (2) negative set, consisting of sequences from regions with significantly weakened signals.

### 4.8. Position Correlation Probability Matrix (PCPM) and Pseudocount

To estimate the interaction energies defined in our model, we first calculated the positional probabilities for all trinucleotides from the sequence data. This was accomplished by constructing a Position Correlation Probability Matrix (PCPM).

In this framework, each *lncRNA* promoter fragment of length *L* (*L* = 600 in this study) was treated as a chain of *L*-2 (i.e., 598) overlapping trinucleotides. For a given histone modification state ζ(ζ∈({pos,neg}), the probability ($p_{j\zeta }^{i}$) of a specific trinucleotide j (where j ∈ {AAA, AAC, …, TTT}) occurring at site i was calculated using a pseudocount method to improve the estimate, as follows [42]:

(2) $$p_{j\zeta }^{i}=\frac{n_{j\zeta }^{i}+b}{N_{\zeta }+B}$$

where $p_{j\zeta }^{i}$ is the estimated probability of trinucleotide j at position  i   for the sequence set in state ζ; $n_{j\zeta }^{i}$ is the observed count of trinucleotide j at position i for the sequence set ζ; $N_{\zeta }$ is the total number of sequences in the sample set ζ; and b and B are the pseudocount parameters calculated as follows:

(3) $$B=\sqrt{N_{\zeta }},$$

(4) $$b=p_{0}\sqrt{N_{\zeta }}$$

where $\;p_{0}$ represents the average background frequency of a trinucleotide.

### 4.9. Construction of Energy Model

The function of a biomolecule is determined by its structure, which in turn is established by the process of energy minimization. For a 600 bp DNA sequence (e.g., AACTTGGTCATGCTTTT...GGGAAATCAT, where A, C, G, and T represent the four deoxynucleotides), its physical conformation is closely associated with the total energy of the fragment. If we consider the interaction energies between adjacent positions (i and i+1) and next-adjacent positions (i and i+2), there are 64 possible trinucleotide combinations at each site. Further increasing the k-mer length (e.g., using tetranucleotides, pentanucleotides, etc.) essentially redundantizes the extraction of these existing adjacent and next-adjacent interactions, thereby creating computational redundancy.

For DNA fragments with increased and decreased histone modification levels, they are expected to exhibit distinct conformations due to their association with different chromatin states, transcription factor recruitment, and DNA methylation patterns. Therefore, even for the identical trinucleotide unit occurring at the same position, its effective interaction energy should differ between the increased and decreased groups. Because the total energy of each sequence is the sum of the interaction energies of all trinucleotide units across the 598 distinct positions, the total energy of the same DNA fragment under increased and decreased modification states will also be different.

According to statistical physics, if a DNA fragment is in a conformational state ξ, and the interaction energy of the j-th trinucleotide unit at the i-th position is $\epsilon_{j}^{i\xi }$, then the probability $p_{j}^{i\xi }$ of this trinucleotide unit appearing at this site follows the Boltzmann distribution:

(5) $$z_{i\zeta }=\sum_{allj}^{64}e^{-\beta \varepsilon_{j\zeta }^{i}}$$

where$\;\;\varepsilon_{j\zeta }^{i}$ is the interaction energy of the physical unit (trinucleotide j) at site j, and $\beta =\frac{1}{kT}$, with k being the Boltzmann constant and T the absolute temperature.

Based on the assumption that the probability of a specific unit occurring at this site follows the Boltzmann distribution, we have the following:

(6) $$p_{j\zeta }^{i}=\frac{1}{z_{i\zeta }}e^{-\beta \varepsilon_{j\zeta }^{i}}$$

By rearranging the terms in the Boltzmann distribution equation, the interaction energy of the physical unit at site i can be derived as follows:

(7) $$\varepsilon_{j\zeta }^{i}=-\frac{1}{\beta }(\ln p_{j\zeta }^{i}+lnz_{i\zeta })$$

where $\varepsilon_{j\zeta }^{i}$ is the interaction energy for trinucleotide j at position i under state ζ. Here, the local constant $C_{i}$ is defined as follows:

(8) $$C_{i}=-kT\ln z_{i\zeta }$$

This equation allows for the direct calculation of interaction energies from the previously computed probabilities ($p_{j\zeta }^{i}$) and partition functions ($z_{j\zeta }^{i}$).

To account for the continuous and holistic nature of the physical properties along the DNA fragment, we defined the total probability $P_{\zeta }$, and the total partition function$\;Z_{\zeta }$ for the entire fragment. These global parameters were calculated as the product of their respective local parameters across all sites.

(9) $$P_{\zeta }=\prod_{i=1}^{L-2}p_{j\zeta }^{i}$$

(10) $$Z_{\zeta }=\prod_{i=1}^{L-2}z_{j\zeta }^{i}$$

Here, the total probability $P_{\zeta }$ is the joint probability of observing the specific sequence of trinucleotides ($j_{1},j_{2},\ldots ,j_{L-2}$) that constitute the fragment. The total partition function$\;Z_{\zeta }$ aggregates the statistical weights from all *L*−2 sites.

The total structural energy *E* of a 600 bp DNA sequence under a specific modification state is defined as the sum of the local interaction energies across all 598 positions:

(11) $$E=\overset{598}{\underset{i=1}{\sum }}\epsilon_{j\zeta }^{i}$$

According to the principles of statistical mechanics, this total structural energy *E* of the local DNA structure is related to the total probability $P_{\zeta }$ and the total partition function ${\;Z}_{\zeta }\;$ by the following equation:

(12) $$E=-kT\left(\ln P_{\zeta }+\ln Z_{\zeta }\right)$$

By substituting Equations (8) and (9) into Equation (10), an expression for the total energy as a sum of local contributions was obtained:

(13) $$E=-kT\overset{598}{\underset{i=1}{\sum }}\ln p_{j}^{i\xi }+C$$

Here, the constant C  is the sum of the reference free energies across all positions:

(14) $$C=\overset{598}{\underset{i=1}{\sum }}C_{i}=-kT\overset{598}{\underset{i=1}{\sum }}\ln z_{i\zeta }$$

Therefore, by calculating the position-specific probability distribution, we are actually using statistical physics principles to infer the underlying energy states under different histone modification conditions, thereby deducing their structural differences.

The term *C* is a constant determined by the local physical environments under different modification levels and serves as the decision threshold for the energy model. In practice, if a query region satisfies ΔE<C, the DNA fragment is classified as a region with significantly increased histone modification signals; otherwise, it is classified as a region with significantly decreased signals.

### 4.10. Performance Evaluation of the Model

To comprehensively evaluate the effectiveness and reliability of our energy model in discriminating between differential histone modification regions, we employed four key metrics: Sensitivity ($S_{n}$), Specificity ($S_{p}$), Accuracy (Acc), and the Matthews Correlation Coefficient (Mcc). The definitions and formulas for these metrics are as follows:

Sensitivity ($S_{n}$): represents the model’s ability to correctly identify regions with significantly increased histone modification signals (the positive set).

(15) $$S_{n}=\frac{TP}{TP+FN}$$

Specificity ($S_{p}$): represents the model’s ability to correctly identify regions with significantly decreased histone modification signals (the negative set).

(16) $$S_{p}=\frac{TN}{TN+FP}$$

Accuracy (Acc): reflects the overall proportion of samples correctly classified in both the positive and negative sets.

(17) $$Acc=\frac{TP+TN}{TP+TN+FP+FN}$$

Matthews Correlation Coefficient (Mcc): a comprehensive metric for classification quality that is robust to class imbalance. The Mcc value ranges from −1 to +1, where a value closer to +1 indicates a stronger ability to distinguish between the physical features.

(18) $$Mcc=\frac{TP\times TN-FP\times FN}{\sqrt{\left(TP+FN\right)\left(TP+FP\right)\left(TN+FP\right)\left(TN+FN\right)}}$$

In the above formulas, TP (true positive) is the number of correctly identified positive samples; TN (true negative) is the number of correctly identified negative samples; FP (false positive) is the number of negative samples incorrectly classified as positive; and FN (false negative) is the number of positive samples incorrectly classified as negative.

## 5. Conclusions

The core finding of this model is that the identical DNA sequence exhibits significant energy differences when scored by the ‘increased’ (positive) matrix versus the ‘decreased’ (negative) matrix. This variation does not stem from changes in the nucleotide composition of the sequence itself; rather, it reflects the structural and epigenetic state variations of the same sequence under two different regulatory matrices (e.g., due to differences in nucleosome binding density or 3D conformations). In this manner, we establish a quantitative link between static sequence organization and dynamic epigenetic remodeling from a statistical physics perspective.

However, as a scoring system based on position-specific frequency statistics, our model has certain limitations. We observed during evaluation that the model is highly sensitive to spatial displacement when processing unseen sequences. Because each of the 600 columns in our matrices is strictly aligned point to point, even a minor offset (as small as a 1–2 bp displacement) of the physical feature sites relative to the coordinates defined by the training matrix will affect the cumulative log-probability summation, thus altering the final energy scores and energy differences. Future iterations of this model will introduce spatial smoothing filter algorithms to enhance its tolerance toward such minor local displacements.

## Figures and Tables

**Figure 3:**
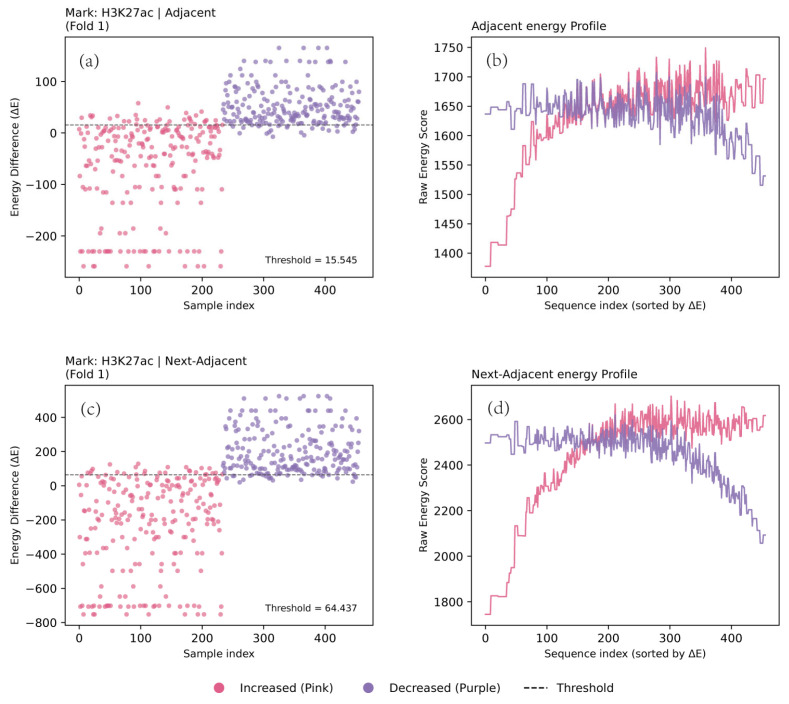
Comparison of dinucleotide and trinucleotide models for discriminating H3K27ac signals. (**a**,**b**) The results of the dinucleotide model for a representative 10-fold cross-validation fold. (**c**,**d**) The results of the trinucleotide model for a representative 10-fold cross-validation fold. (**a**,**c**) The distribution of the energy difference (ΔE) for each sequence in the test sets, where the horizontal dashed line indicates the classification threshold. (**b**,**d**) The score curves of the positive ($E_{positive}$) and negative ($E_{negative}$) energies for the test sequences sorted by their ΔE values, providing the energy profile.

**Figure 4:**
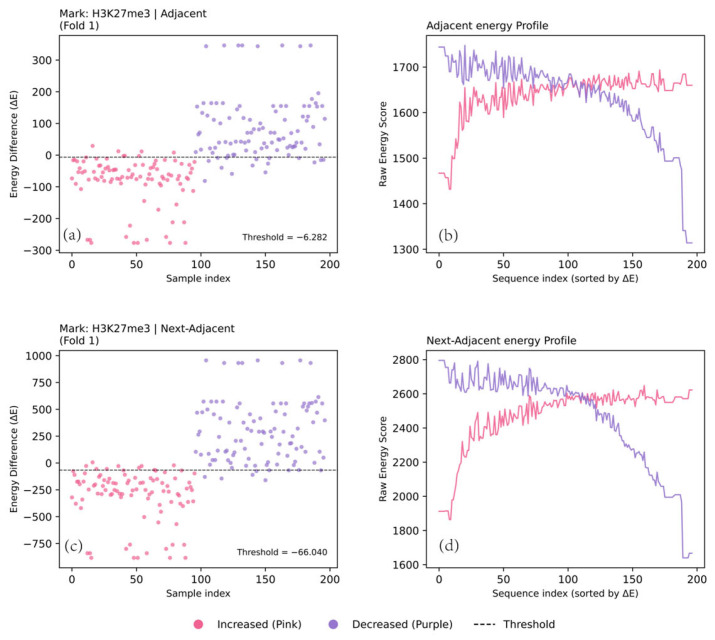
Comparison of dinucleotide and trinucleotide models for discriminating H3K27me3 signals. (**a**,**b**) The results of the dinucleotide model for a representative 10-fold cross-validation fold. (**a**) The distribution of the energy difference (ΔE) for each sequence in the test sets, where the horizontal dashed line indicates the classification threshold. (**b**) The score curves of the positive ($E_{positive}$) and negative ($E_{negative}$) energies for the test sequences sorted by their ΔE values. (**c**,**d**) The corresponding results for the trinucleotide model, with (**c**) representing the ΔE distribution and (**d**) showing the raw energy separation profile.

**Table 1:** Predictive performance of the dinucleotide and trinucleotide energy models in distinguishing increased versus decreased histone modification states in hepatocellular carcinoma (10-fold cross-validation).

Mark	Adjacent _S_n_ (%)	Adjacent _S_p_ (%)	Adjacent _MCC	Adjacent _ACC (%)
H2AFZ	92.100 ± 3.100	80.900 ± 3.100	0.737 ± 0.033	86.800 ± 2.900
H3K27ac	85.700 ± 1.600	88.900 ± 2.000	0.746 ± 0.009	87.400 ± 2.400
H3K27me3	91.300 ± 2.200	86.200 ± 2.100	0.777 ± 0.028	89.200 ± 4.000
H3K36me3	89.600 ± 3.100	88.100 ± 1.400	0.778 ± 0.037	89.600 ± 6.000
H3K4me1	83.400 ± 2.800	87.400 ± 2.500	0.711 ± 0.018	85.700 ± 2.800
H3K4me2	82.200 ± 2.600	85.800 ± 2.300	0.682 ± 0.023	84.500 ± 4.100
H3K4me3	87.500 ± 1.400	79.900 ± 3.000	0.677 ± 0.029	83.800 ± 2.500
H3K79me2	85.100 ± 2.700	89.000 ± 2.100	0.743 ± 0.024	87.400 ± 3.700
H3K9ac	77.700 ± 3.700	87.200 ± 2.500	0.654 ± 0.029	82.700 ± 3.100
H3K9me3	90.900 ± 3.000	89.100 ± 2.700	0.802 ± 0.035	91.700 ± 8.000
H4K20me1	91.600 ± 1.700	83.400 ± 2.600	0.752 ± 0.030	88.000 ± 4.800

**Table 2:** Predictive performance of the adjacent three-base energy models in distinguishing increased versus decreased histone modification states in hepG2 cell (10-fold cross-validation).

Mark	Next-Adjacent _S_n_ (%)	Next-Adjacent _S_p_ (%)	Next-Adjacent _MCC	Next-Adjacent _ACC (%)
H2AFZ	93.600 ± 1.300	91.600 ± 0.800	0.852 ± 0.015	92.900 ± 3.400
H3K27ac	89.700 ± 1.200	90.800 ± 1.300	0.805 ± 0.009	90.500 ± 2.700
H3K27me3	88.900 ± 1.000	93.400 ± 1.400	0.824 ± 0.017	91.600 ± 4.600
H3K36me3	93.700 ± 2.200	88.100 ± 2.100	0.818 ± 0.029	91.400 ± 5.200
H3K4me1	90.000 ± 1.000	90.100 ± 1.900	0.800 ± 0.019	90.200 ± 2.700
H3K4me2	86.100 ± 1.700	91.200 ± 1.600	0.774 ± 0.026	89.100 ± 4.300
H3K4me3	91.300 ± 1.200	90.100 ± 2.300	0.813 ± 0.025	90.900 ± 3.000
H3K79me2	88.900 ± 2.000	92.500 ± 1.000	0.815 ± 0.020	91.100 ± 3.500
H3K9ac	85.900 ± 2.100	91.200 ± 1.900	0.772 ± 0.026	88.800 ± 2.900
H3K9me3	84.800 ± 3.500	96.400 ± 1.900	0.817 ± 0.039	92.200 ± 8.600
H4K20me1	92.200 ± 1.400	88.200 ± 1.500	0.804 ± 0.018	90.800 ± 4.800

## Data Availability

All the data for this study were obtained from publicly available databases such as ENCODE (https://www.encodeproject.org/, accessed on 6 August 2025).

## Supplementary Materials

The following supporting information can be downloaded at https://www.mdpi.com/article/10.3390/ijms27135653/s1.

## Abbreviations

The following abbreviations are used in this manuscript:

lncRNA	long non-coding RNA
DElncRNAs	differentially expressed lncRNAs
RNA-seq	RNA sequencing
ChIP-seq	Chromatin Immunoprecipitation Sequencing
TSS	transcription start site
ΔE	energy difference
PCPM	Position Correlation Probability Matrix
