# Supplementary material for: An Energy Model Based on Molecular Structure for Predicting Histone Modification Levels at lncRNA Promoter Regions in HepG2 Cells
Source: Int J Mol Sci. 2026 Jun 23;27(13):5653. doi: 10.3390/ijms27135653 (PMC13361589; doi:10.3390/ijms27135653)
Supplement: Supplementary file 1 [file ijms-27-05653-s001.zip › Figure_S10_H3K27me3_Report.pdf]

Performance Metrics: H3K27me3
(Folds 2 to 10)

Table S10__H3K27me3. Supplementary table showing per-fold quantitative metrics for H3K27me3. All values are presented as mean ± confidence

Model	Fold	Sn (%)	Sp (%)	Ac (%)	MCC	auROC
Adjacent	2	95.918	87.879	92.347	0.84	0.964
Adjacent	3	86.408	84.043	81.553	0.705	0.935
Adjacent	4	85.149	91.667	86.139	0.769	0.968
Adjacent	5	94.286	81.522	82.857	0.769	0.953
Adjacent	6	91.089	89.583	88.119	0.807	0.963
Adjacent	7	90.196	82.979	83.333	0.735	0.941
Adjacent	8	90.722	90.909	91.753	0.816	0.958
Adjacent	9	90.0	82.759	105.0	0.717	0.952
Adjacent	10	97.0	85.417	89.5	0.831	0.974
Next-Adjacent	2	89.796	94.949	92.857	0.849	0.986
Next-Adjacent	3	86.408	89.362	83.981	0.757	0.972
Next-Adjacent	4	86.139	95.833	88.614	0.822	0.983
Next-Adjacent	5	91.429	90.217	85.238	0.816	0.978
Next-Adjacent	6	88.119	92.708	88.119	0.808	0.982
Next-Adjacent	7	88.235	94.681	87.745	0.829	0.976
Next-Adjacent	8	88.66	94.949	92.784	0.838	0.983
Next-Adjacent	9	90.0	92.241	111.875	0.821	0.981
Next-Adjacent	10	90.0	96.875	91.5	0.87	0.992

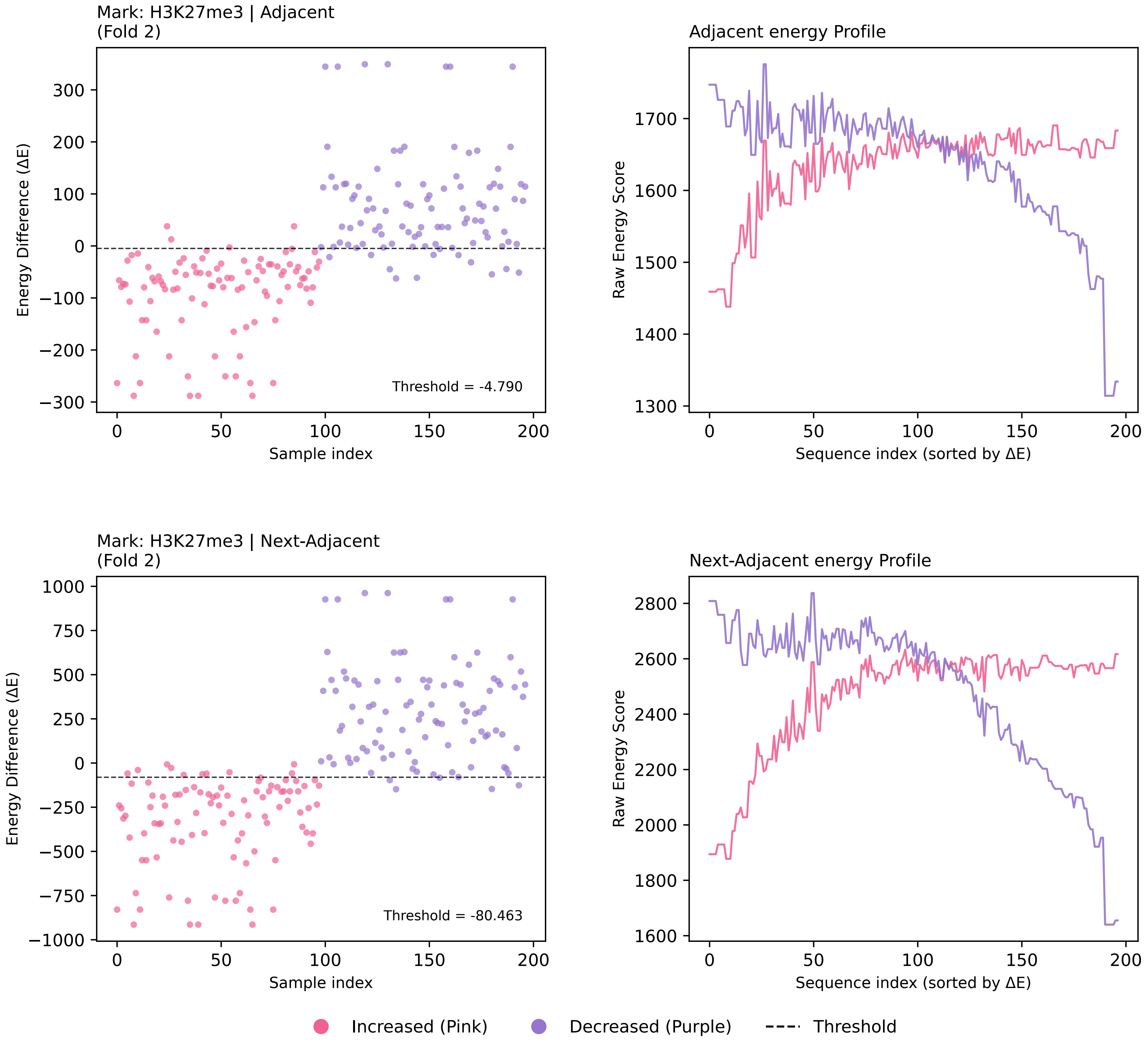

Figure S_Core_Remain_H3K27me3 (Fold 2). Top: Adjacent; Bottom: Next-Adjacent.
Left panels: Scatter plots of energy differences (ΔE); Right panels: Raw energy score profile curves along the sorted sequences.

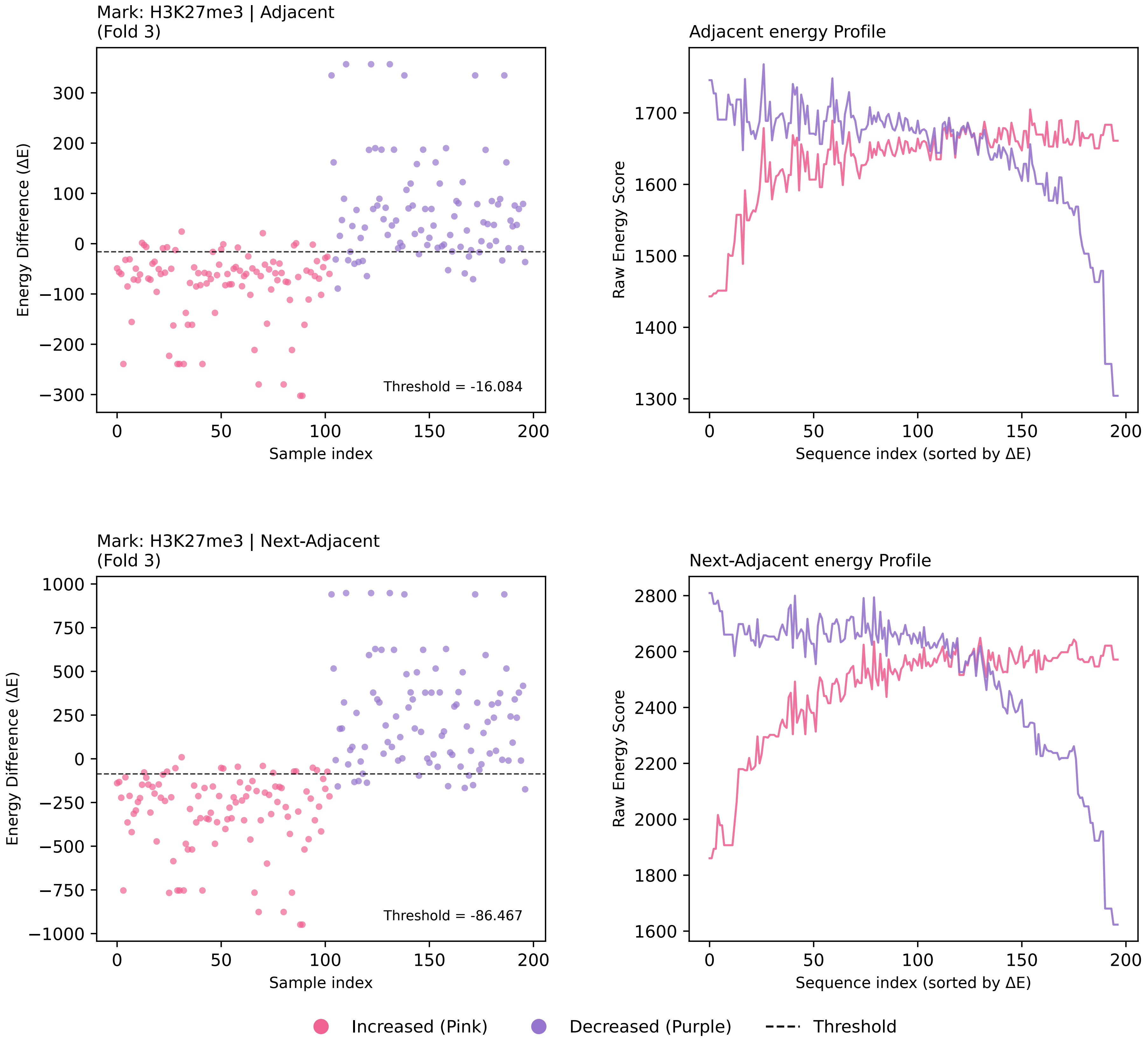

Figure S_Core_Remain_H3K27me3 (Fold 3). Top: Adjacent; Bottom: Next-Adjacent.
Left panels: Scatter plots of energy differences (ΔE); Right panels: Raw energy score profile curves along the sorted sequences.

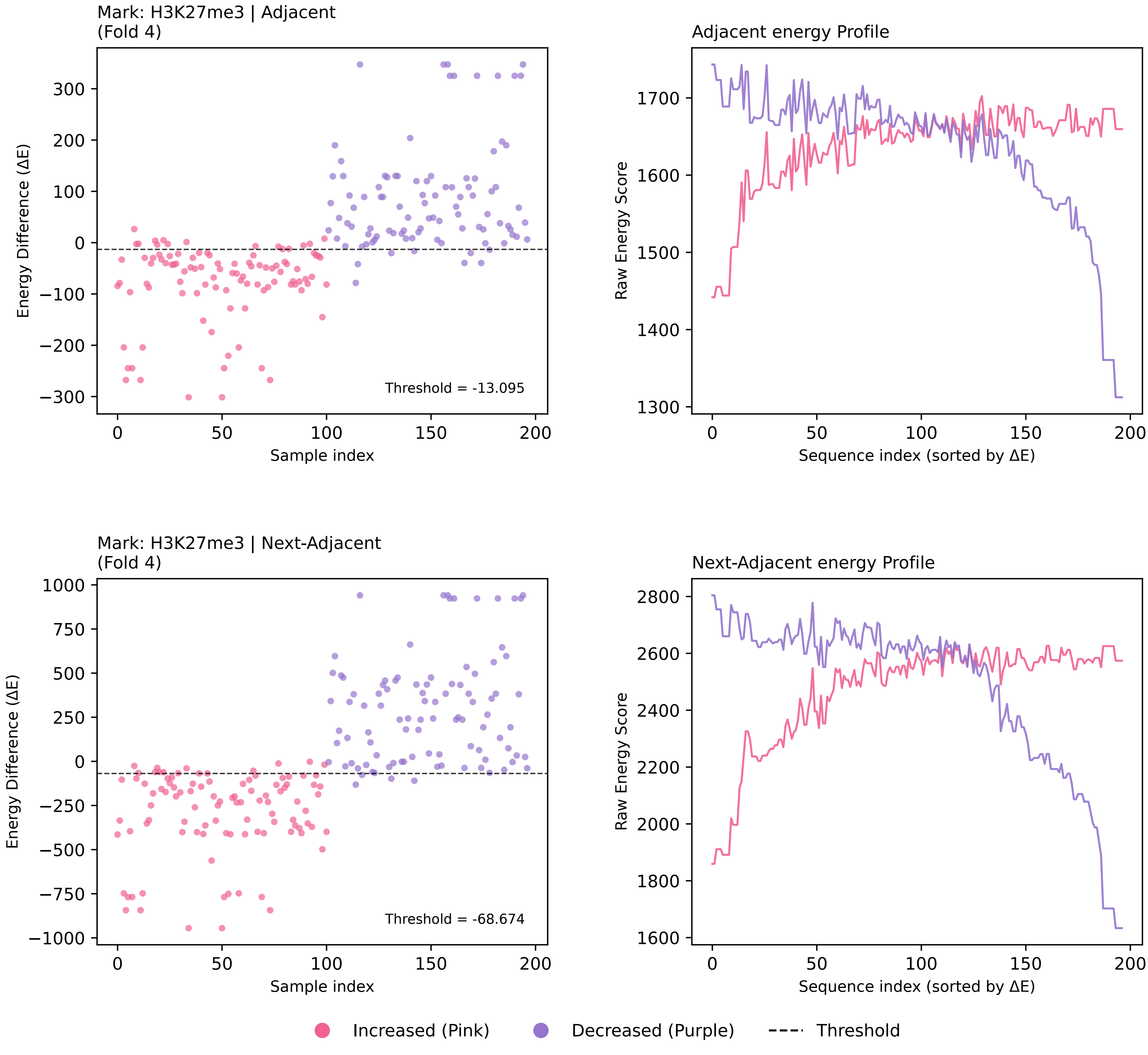

Figure S_Core_Remain_H3K27me3 (Fold 4). Top: Adjacent; Bottom: Next-Adjacent.
Left panels: Scatter plots of energy differences (ΔE); Right panels: Raw energy score profile curves along the sorted sequences.

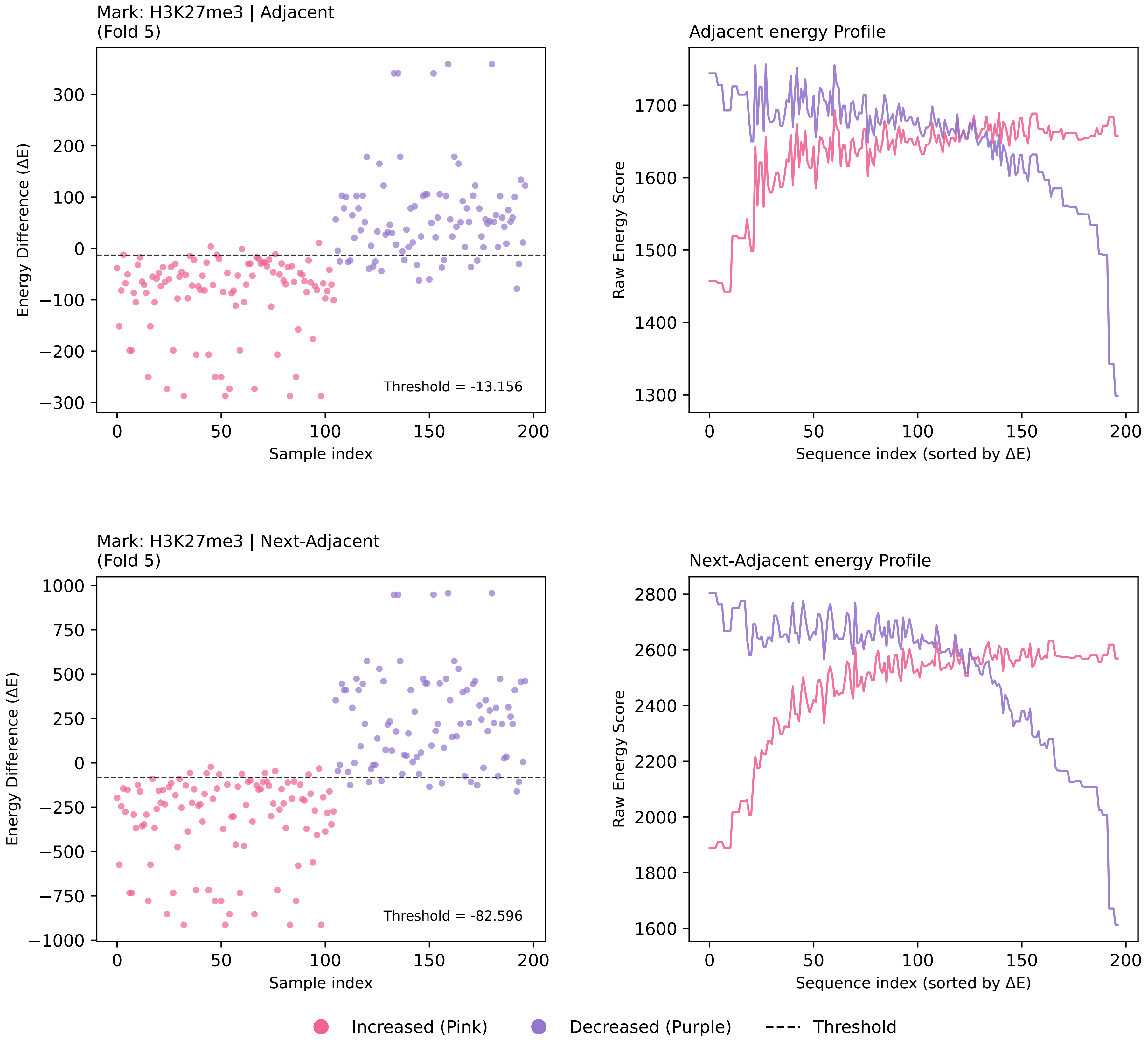

Figure S_Core_Remain_H3K27me3 (Fold 5). Top: Adjacent; Bottom: Next-Adjacent.
Left panels: Scatter plots of energy differences (ΔE); Right panels: Raw energy score profile curves along the sorted sequences.

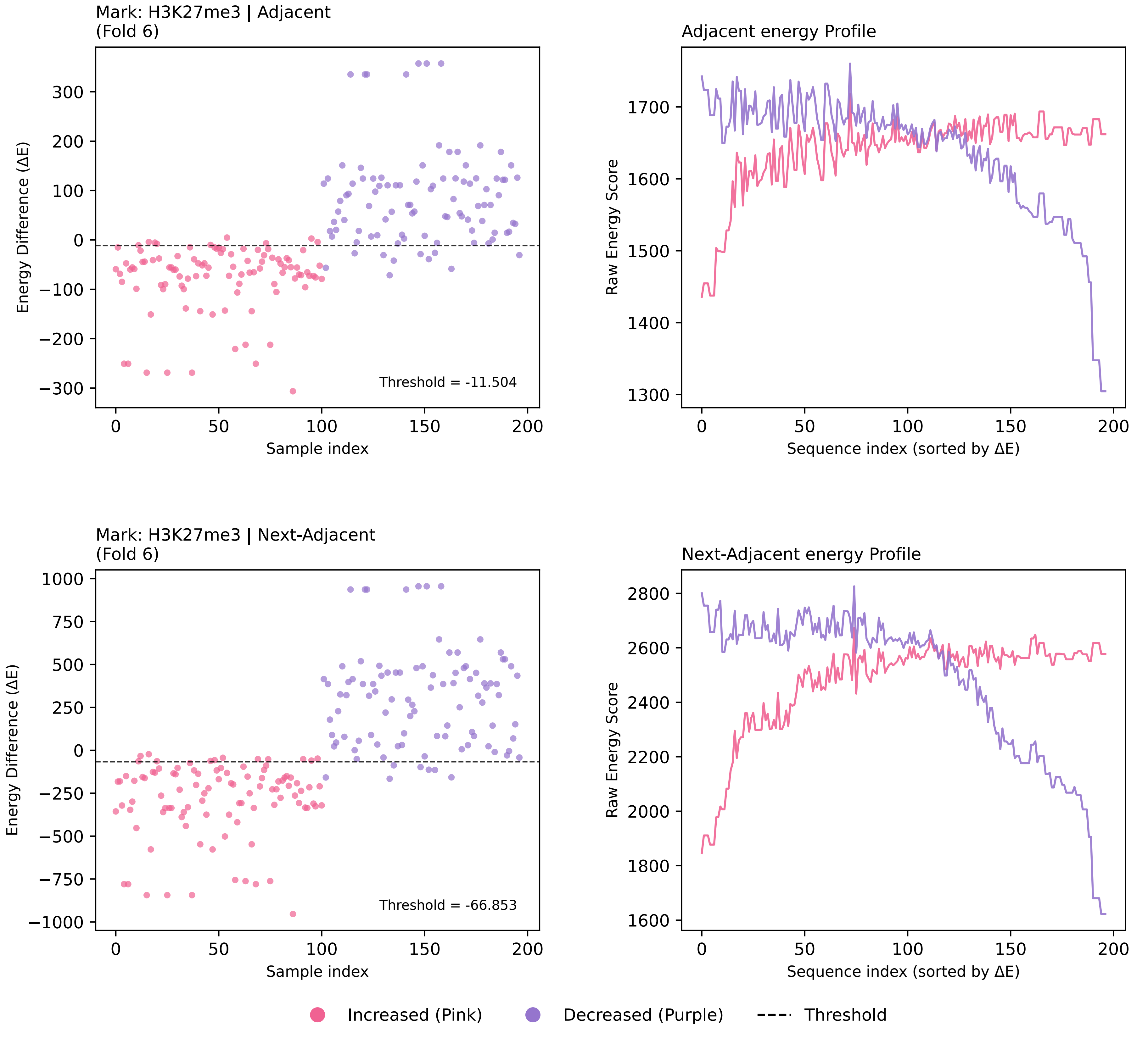

Figure S_Core_Remain_H3K27me3 (Fold 6). Top: Adjacent; Bottom: Next-Adjacent.
Left panels: Scatter plots of energy differences (ΔE); Right panels: Raw energy score profile curves along the sorted sequences.

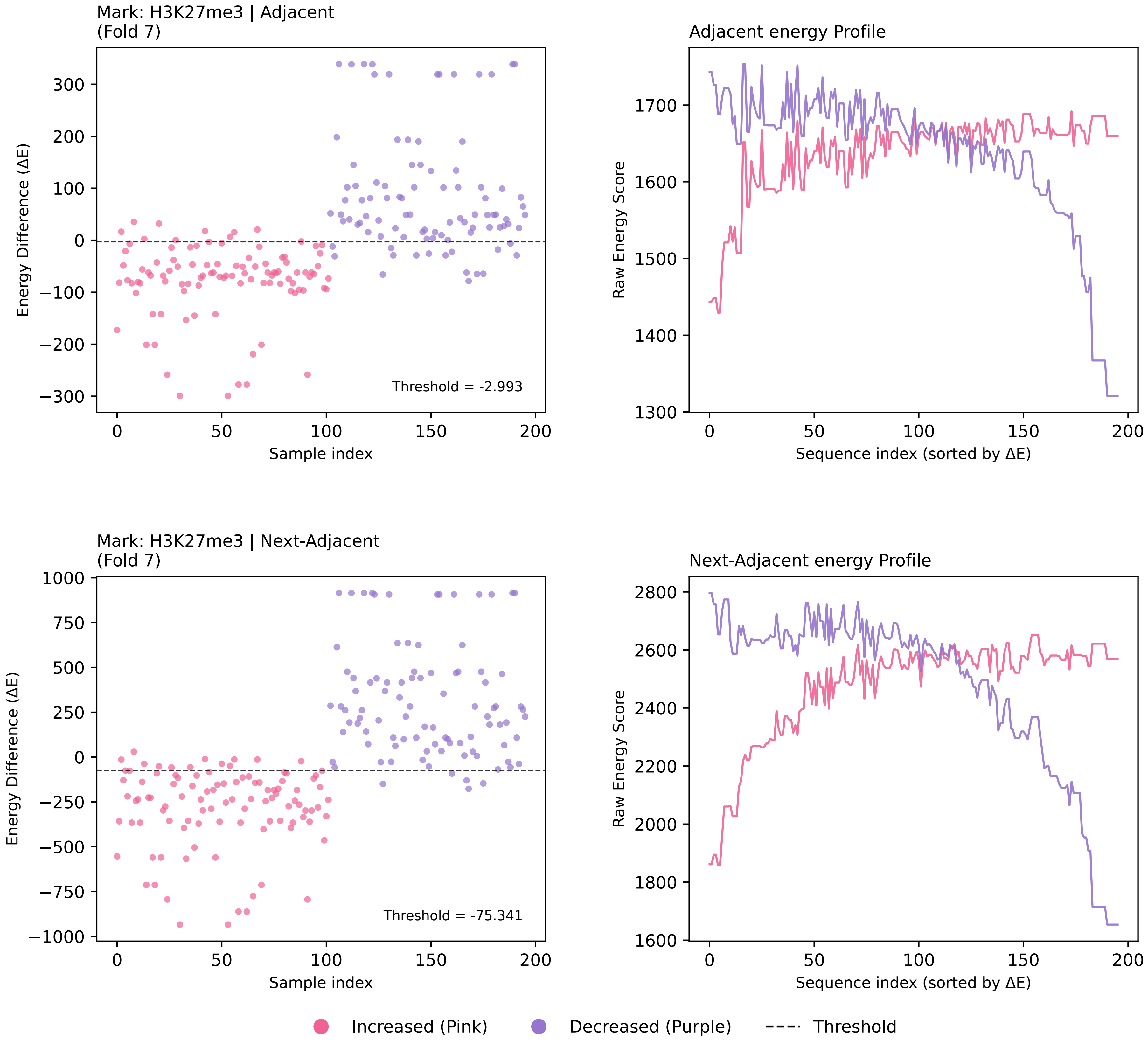

Figure S_Core_Remain_H3K27me3 (Fold 7). Top: Adjacent; Bottom: Next-Adjacent.
Left panels: Scatter plots of energy differences (ΔE); Right panels: Raw energy score profile curves along the sorted sequences.

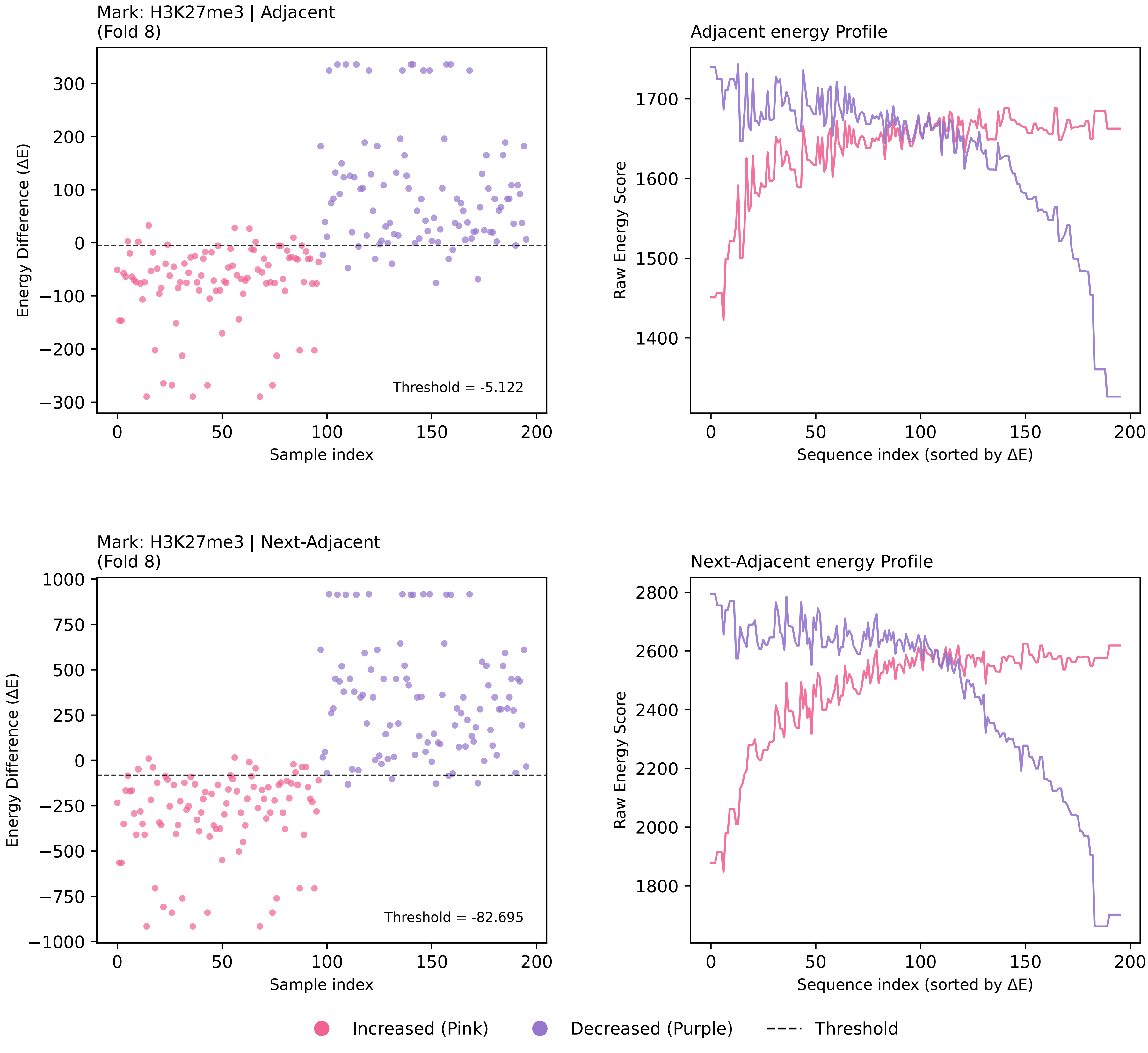

Figure S_Core_Remain_H3K27me3 (Fold 8). Top: Adjacent; Bottom: Next-Adjacent.
Left panels: Scatter plots of energy differences (ΔE); Right panels: Raw energy score profile curves along the sorted sequences.

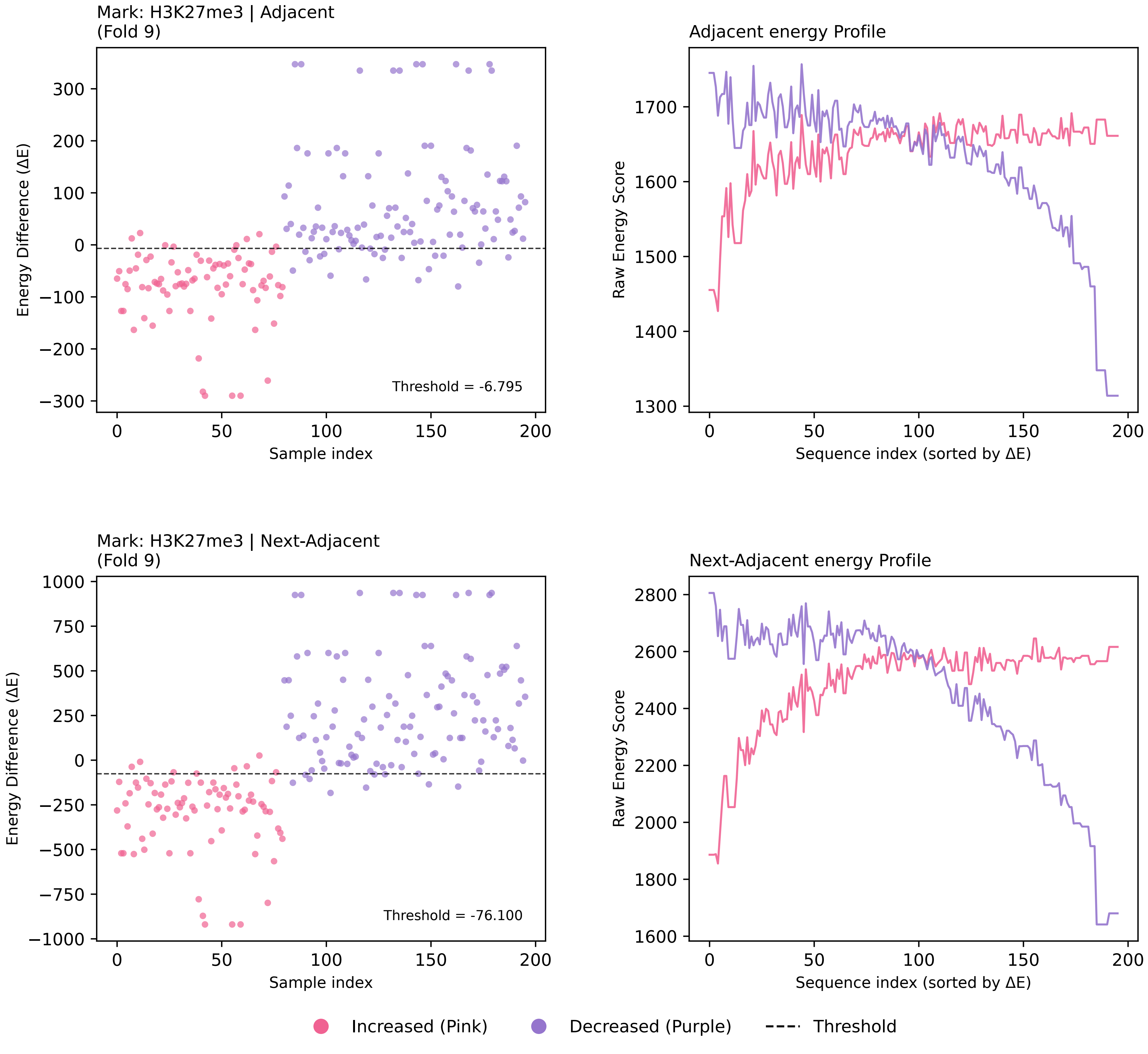

Figure S_Core_Remain_H3K27me3 (Fold 9). Top: Adjacent; Bottom: Next-Adjacent.
Left panels: Scatter plots of energy differences (ΔE); Right panels: Raw energy score profile curves along the sorted sequences.

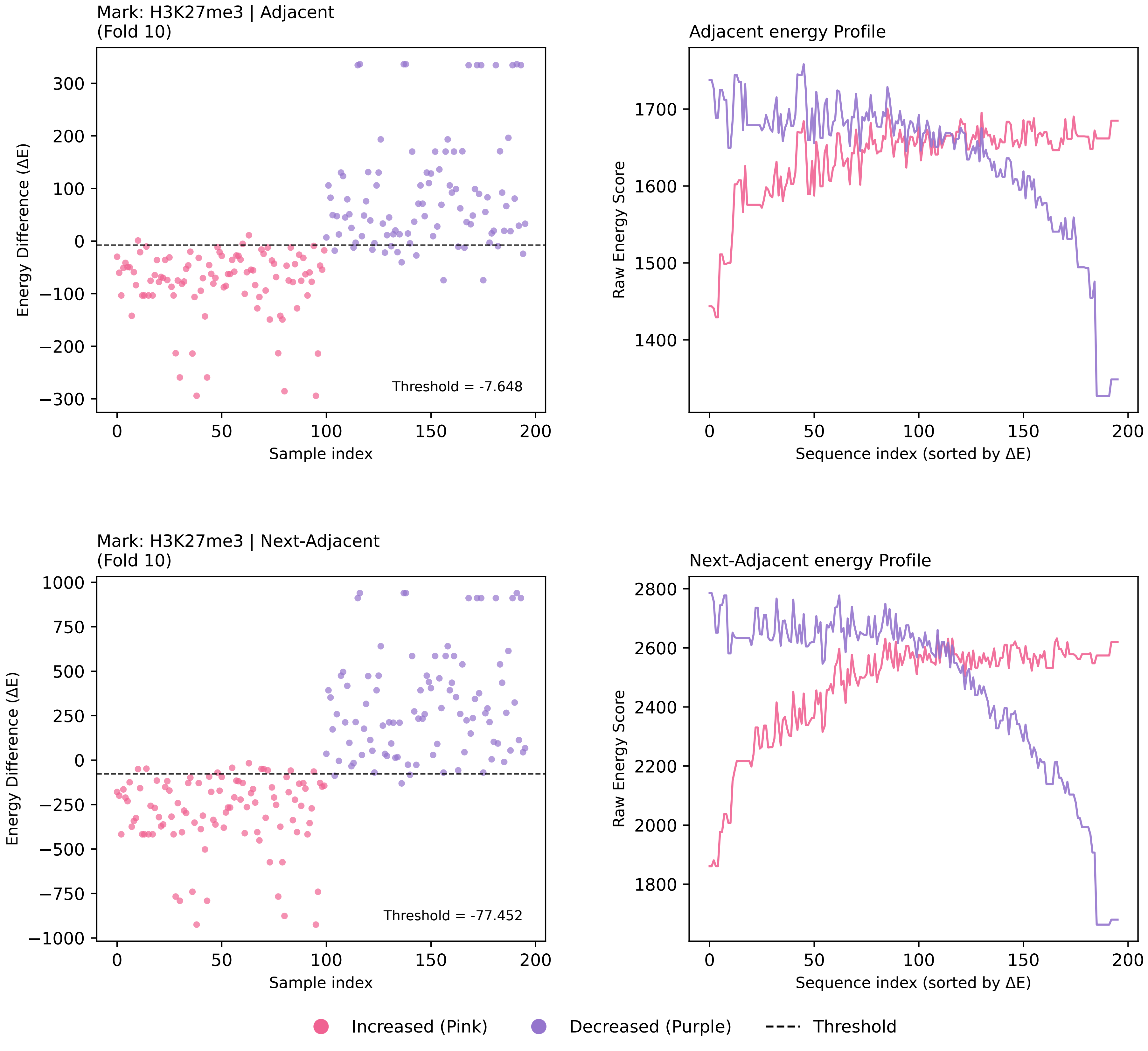

Figure S_Core_Remain_H3K27me3 (Fold 10). Top: Adjacent; Bottom: Next-Adjacent.
Left panels: Scatter plots of energy differences (ΔE); Right panels: Raw energy score profile curves along the sorted sequences.
