# Supplementary material for: An Energy Model Based on Molecular Structure for Predicting Histone Modification Levels at lncRNA Promoter Regions in HepG2 Cells
Source: Int J Mol Sci. 2026 Jun 23;27(13):5653. doi: 10.3390/ijms27135653 (PMC13361589; doi:10.3390/ijms27135653)
Supplement: Supplementary file 1 [file ijms-27-05653-s001.zip › Figure_S11_H3K36me3_Report.pdf]

Performance Metrics: H3K36me3
(Folds 2 to 10)

Table S11__H3K36me3. Supplementary table showing per-fold quantitative metrics for H3K36me3. All values are presented as mean ± confidence

Model	Fold	Sn (%)	Sp (%)	Ac (%)	MCC	auROC
Adjacent	2	80.612	86.813	80.612	0.674	0.943
Adjacent	3	86.316	85.106	85.263	0.714	0.954
Adjacent	4	96.0	88.764	87.5	0.853	0.979
Adjacent	5	95.0	87.5	86.0	0.83	0.973
Adjacent	6	90.909	87.64	84.848	0.787	0.965
Adjacent	7	92.708	88.043	88.542	0.809	0.979
Adjacent	8	88.043	85.417	88.587	0.734	0.94
Adjacent	9	94.667	92.92	117.333	0.869	0.985
Adjacent	10	89.691	87.912	86.082	0.776	0.963
Next-Adjacent	2	89.796	93.407	88.265	0.831	0.976
Next-Adjacent	3	90.526	87.234	88.421	0.778	0.972
Next-Adjacent	4	98.0	86.517	87.5	0.855	0.983
Next-Adjacent	5	95.0	86.364	85.5	0.82	0.982
Next-Adjacent	6	91.919	88.764	85.859	0.808	0.972
Next-Adjacent	7	93.75	94.565	92.188	0.883	0.99
Next-Adjacent	8	88.043	82.292	86.957	0.704	0.959
Next-Adjacent	9	100.0	86.726	115.333	0.85	0.991
Next-Adjacent	10	94.845	87.912	88.66	0.831	0.981

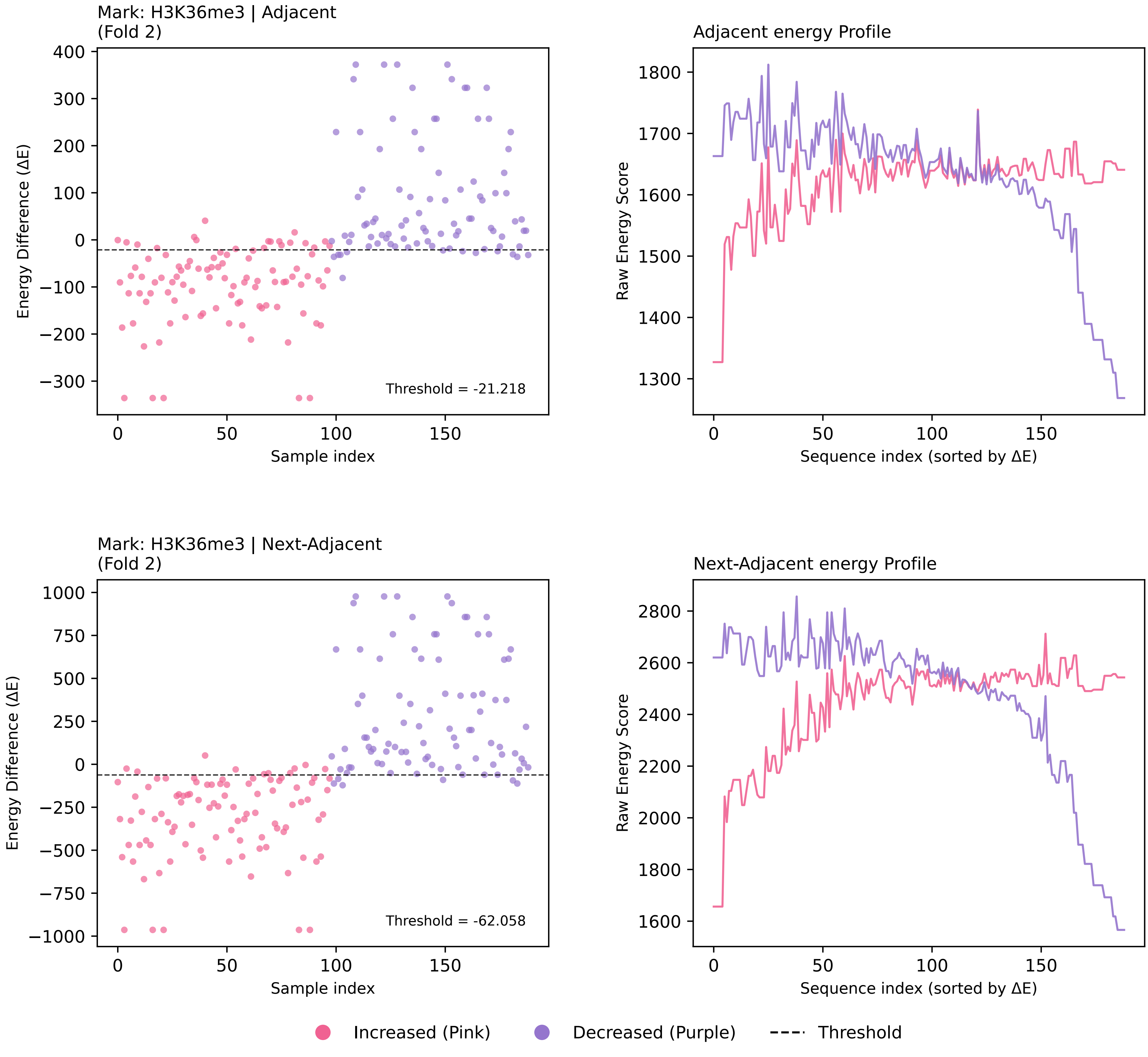

Figure S_Core_Remain_H3K36me3 (Fold 2). Top: Adjacent; Bottom: Next-Adjacent.
Left panels: Scatter plots of energy differences (ΔE); Right panels: Raw energy score profile curves along the sorted sequences.

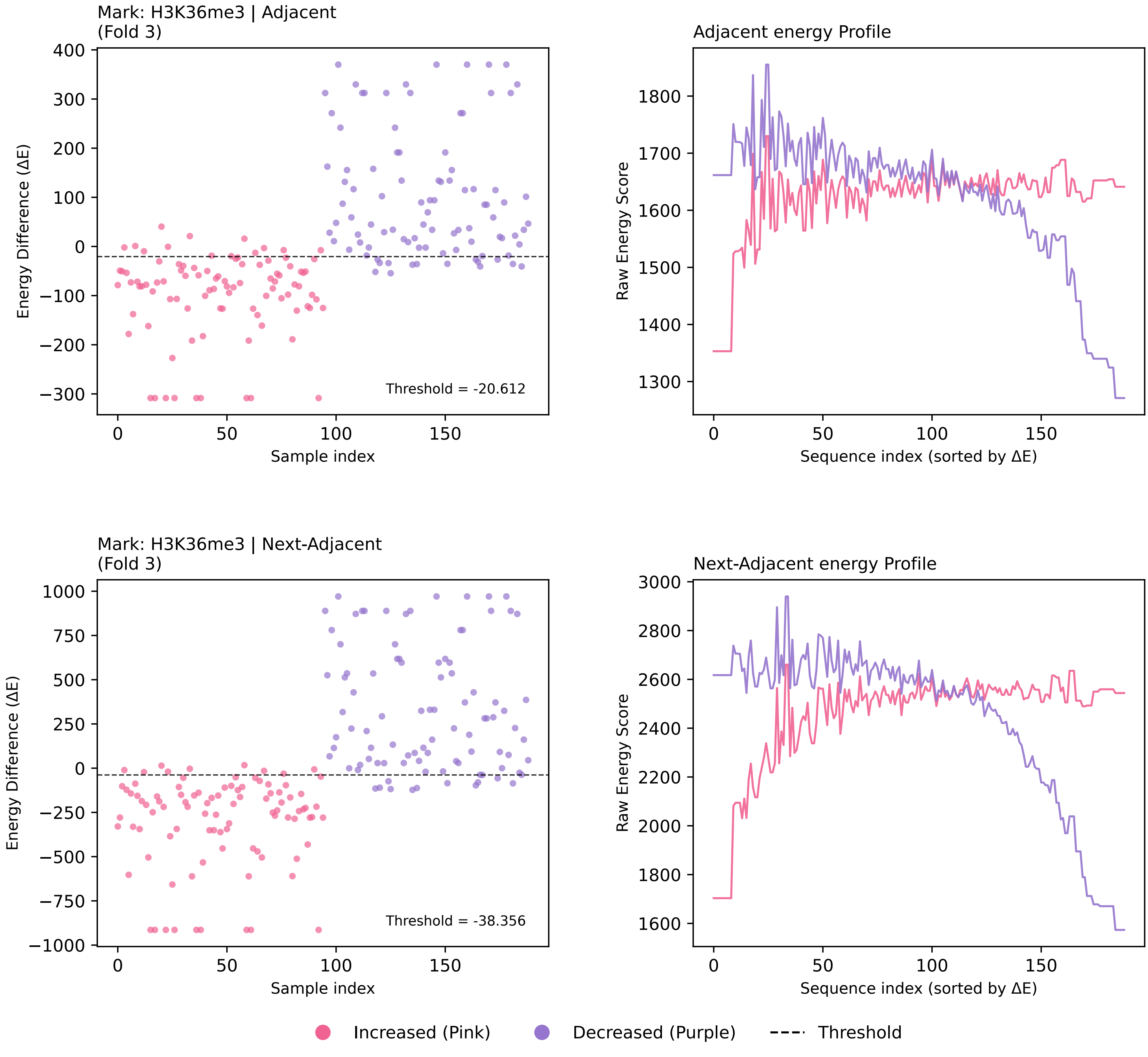

Figure S_Core_Remain_H3K36me3 (Fold 3). Top: Adjacent; Bottom: Next-Adjacent.
Left panels: Scatter plots of energy differences (ΔE); Right panels: Raw energy score profile curves along the sorted sequences.

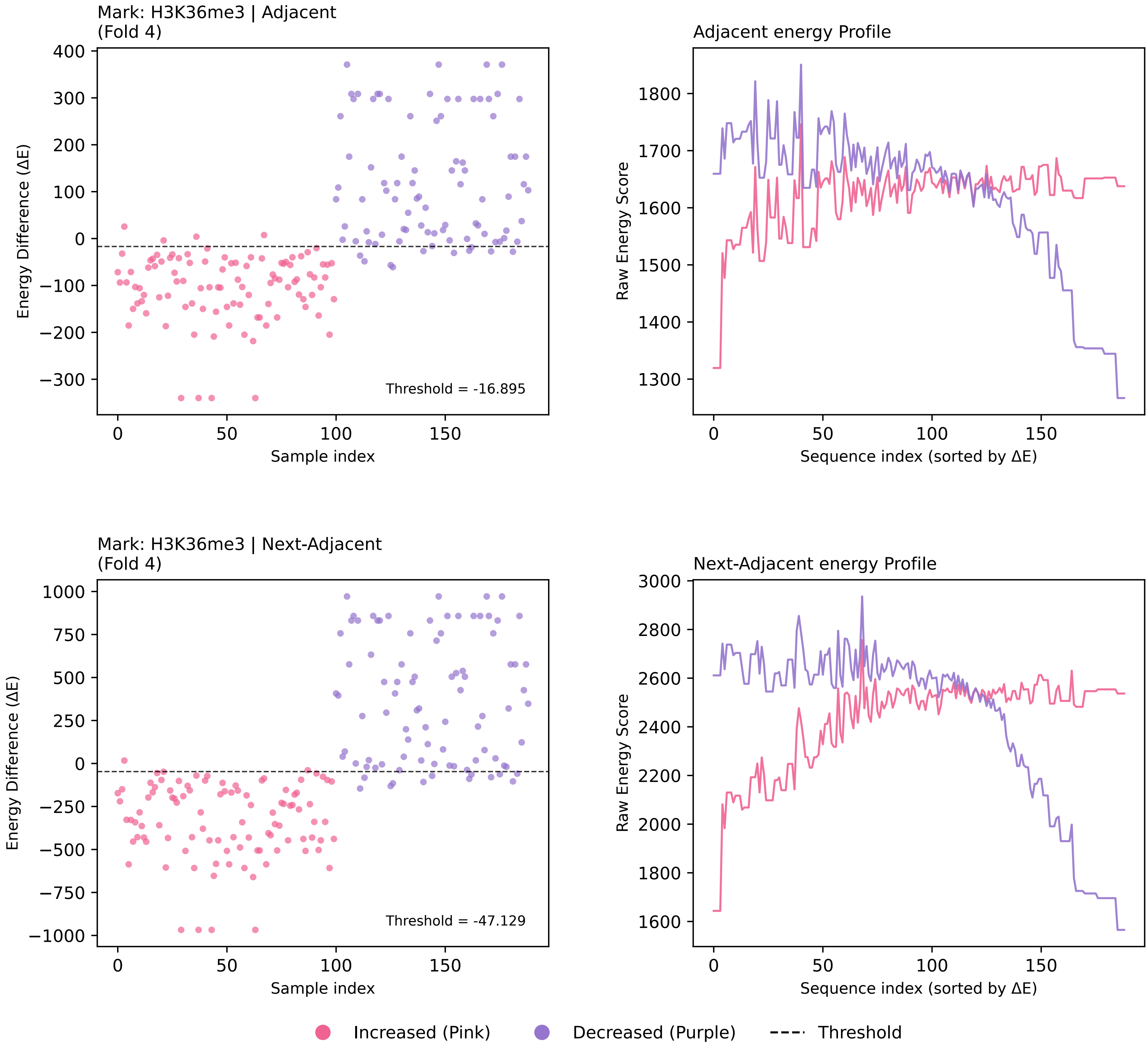

Figure S_Core_Remain_H3K36me3 (Fold 4). Top: Adjacent; Bottom: Next-Adjacent.
Left panels: Scatter plots of energy differences (ΔE); Right panels: Raw energy score profile curves along the sorted sequences.

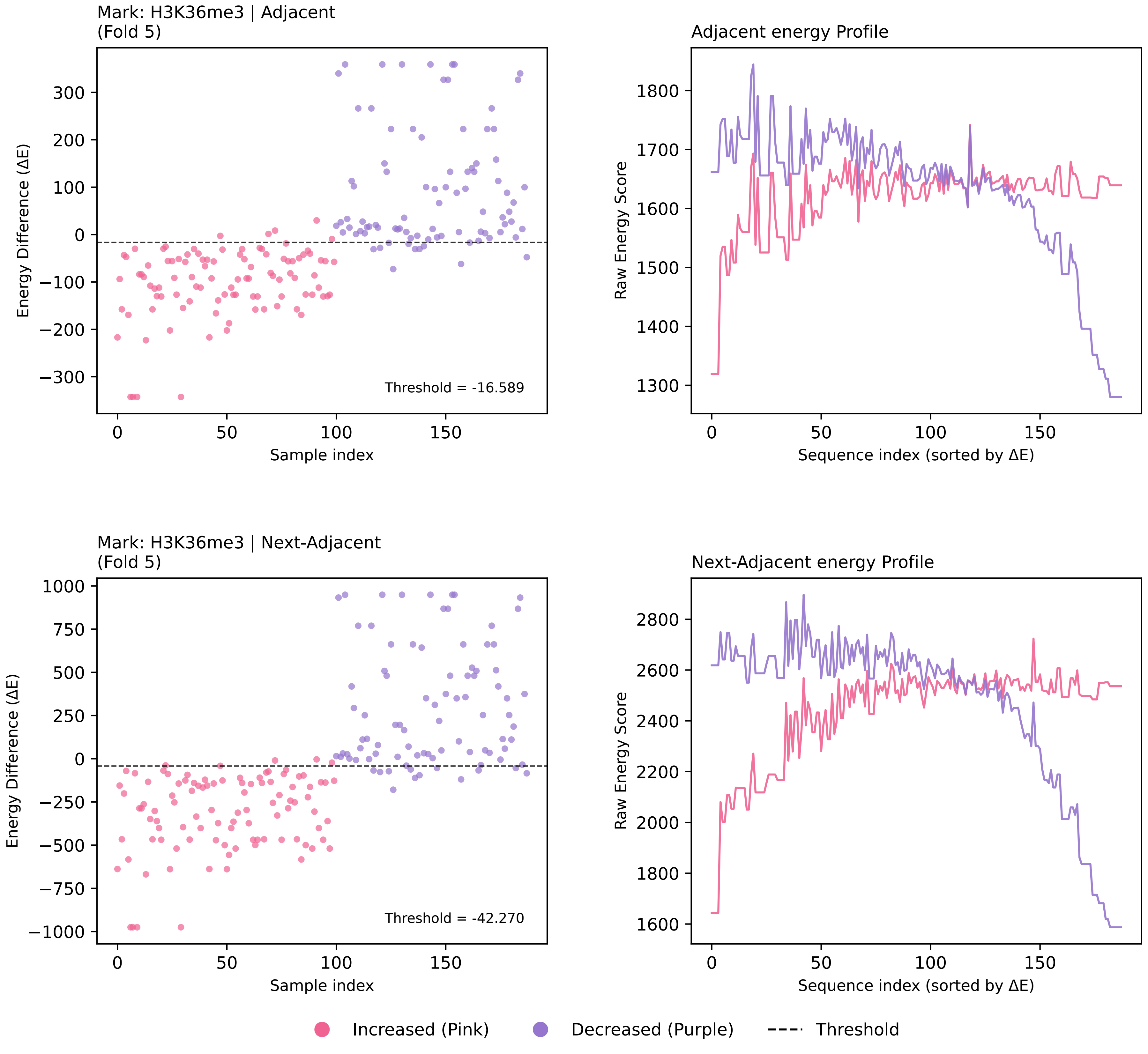

Figure S_Core_Remain_H3K36me3 (Fold 5). Top: Adjacent; Bottom: Next-Adjacent.
Left panels: Scatter plots of energy differences (ΔE); Right panels: Raw energy score profile curves along the sorted sequences.

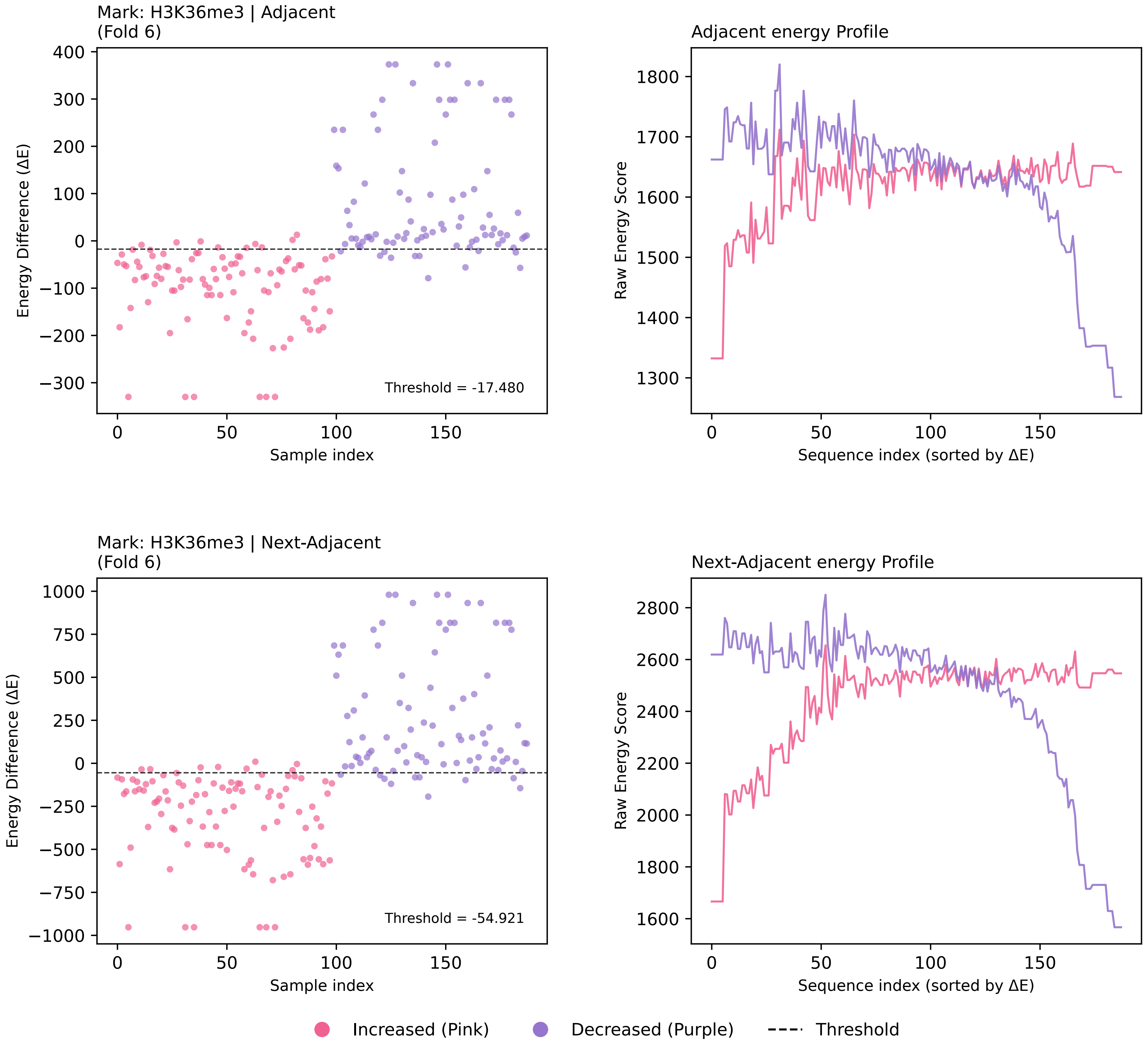

Figure S_Core_Remain_H3K36me3 (Fold 6). Top: Adjacent; Bottom: Next-Adjacent.
Left panels: Scatter plots of energy differences (ΔE); Right panels: Raw energy score profile curves along the sorted sequences.

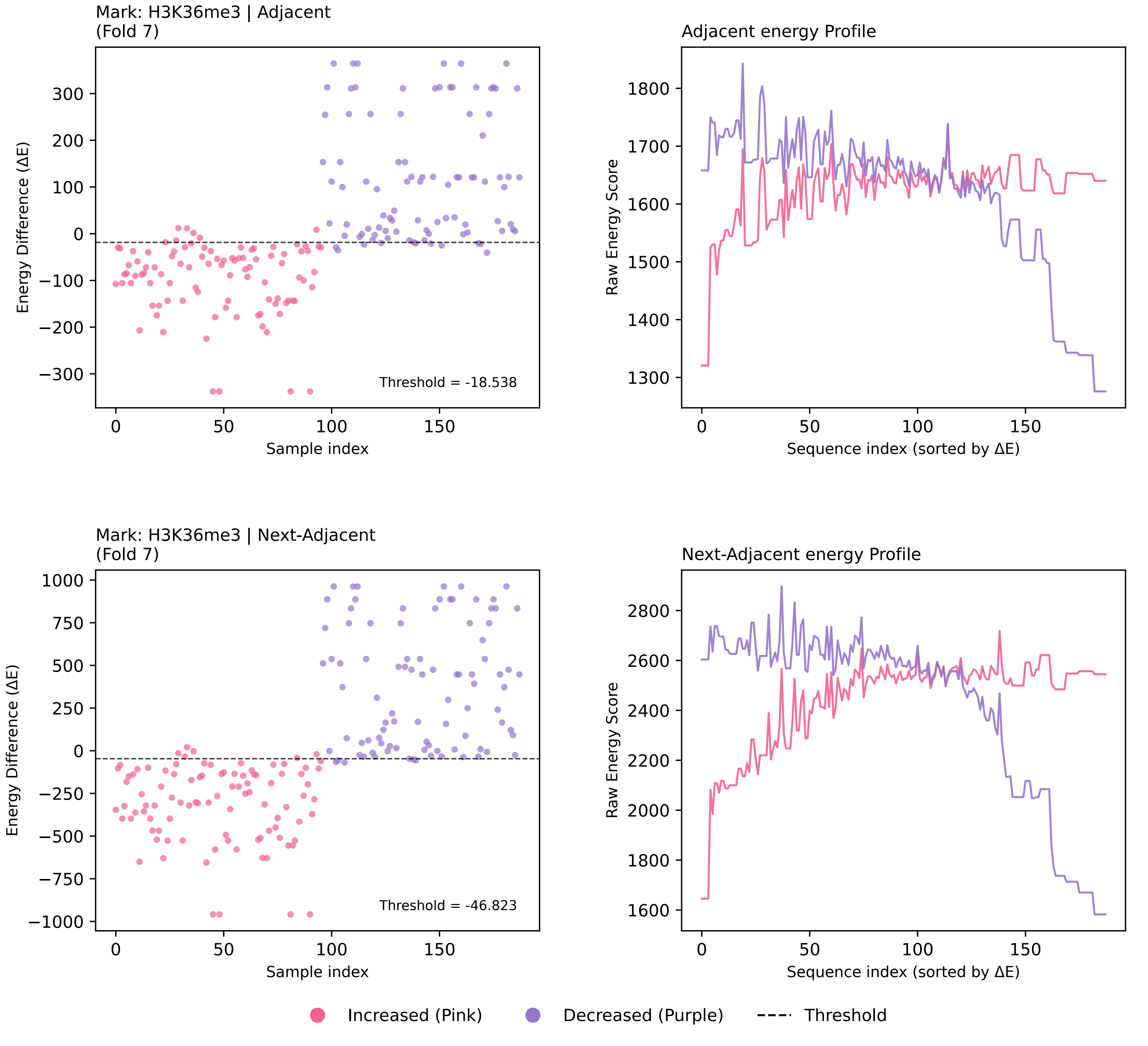

Figure S_Core_Remain_H3K36me3 (Fold 7). Top: Adjacent; Bottom: Next-Adjacent.
Left panels: Scatter plots of energy differences (ΔE); Right panels: Raw energy score profile curves along the sorted sequences.

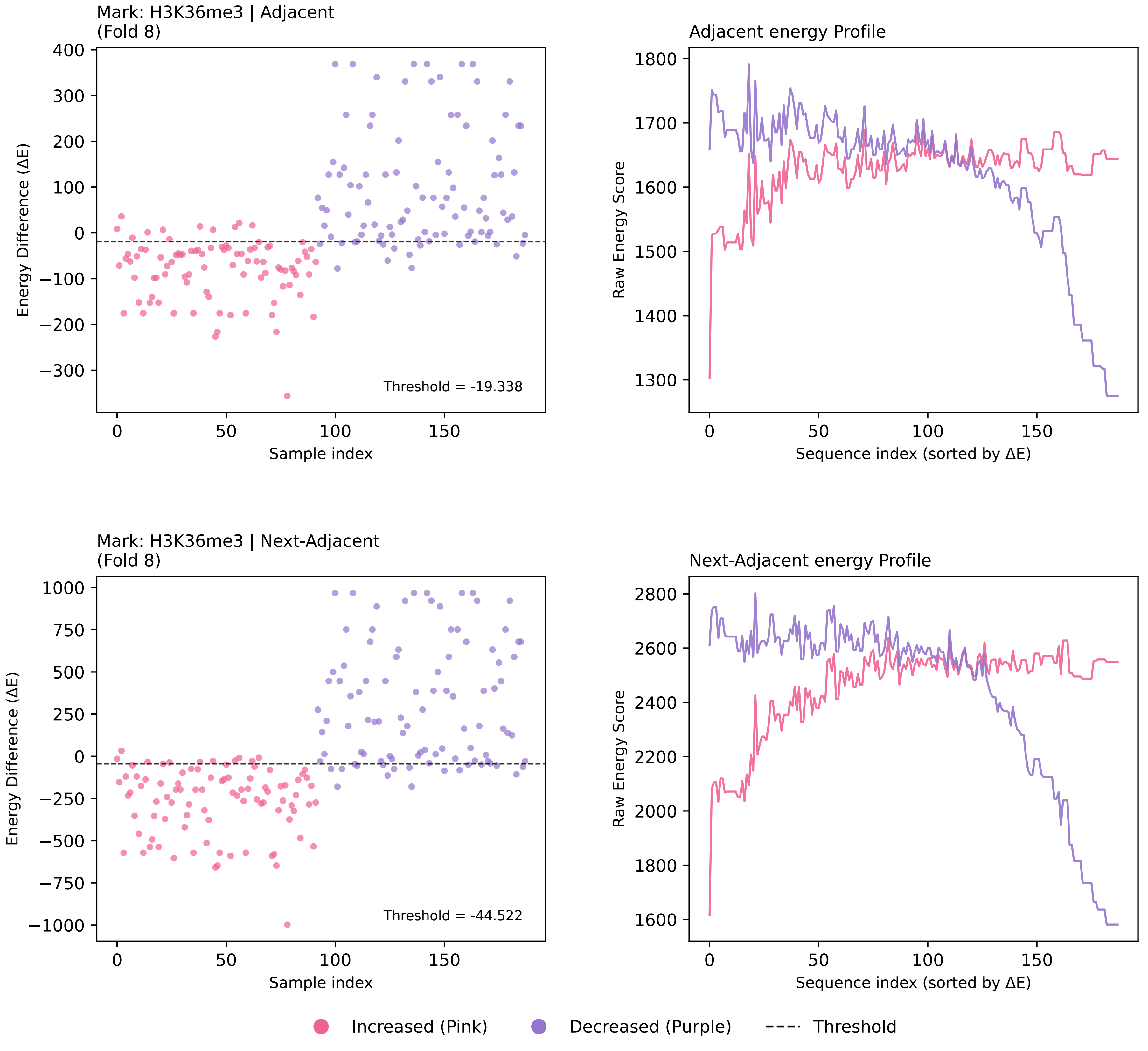

Figure S_Core_Remain_H3K36me3 (Fold 8). Top: Adjacent; Bottom: Next-Adjacent.
Left panels: Scatter plots of energy differences (ΔE); Right panels: Raw energy score profile curves along the sorted sequences.

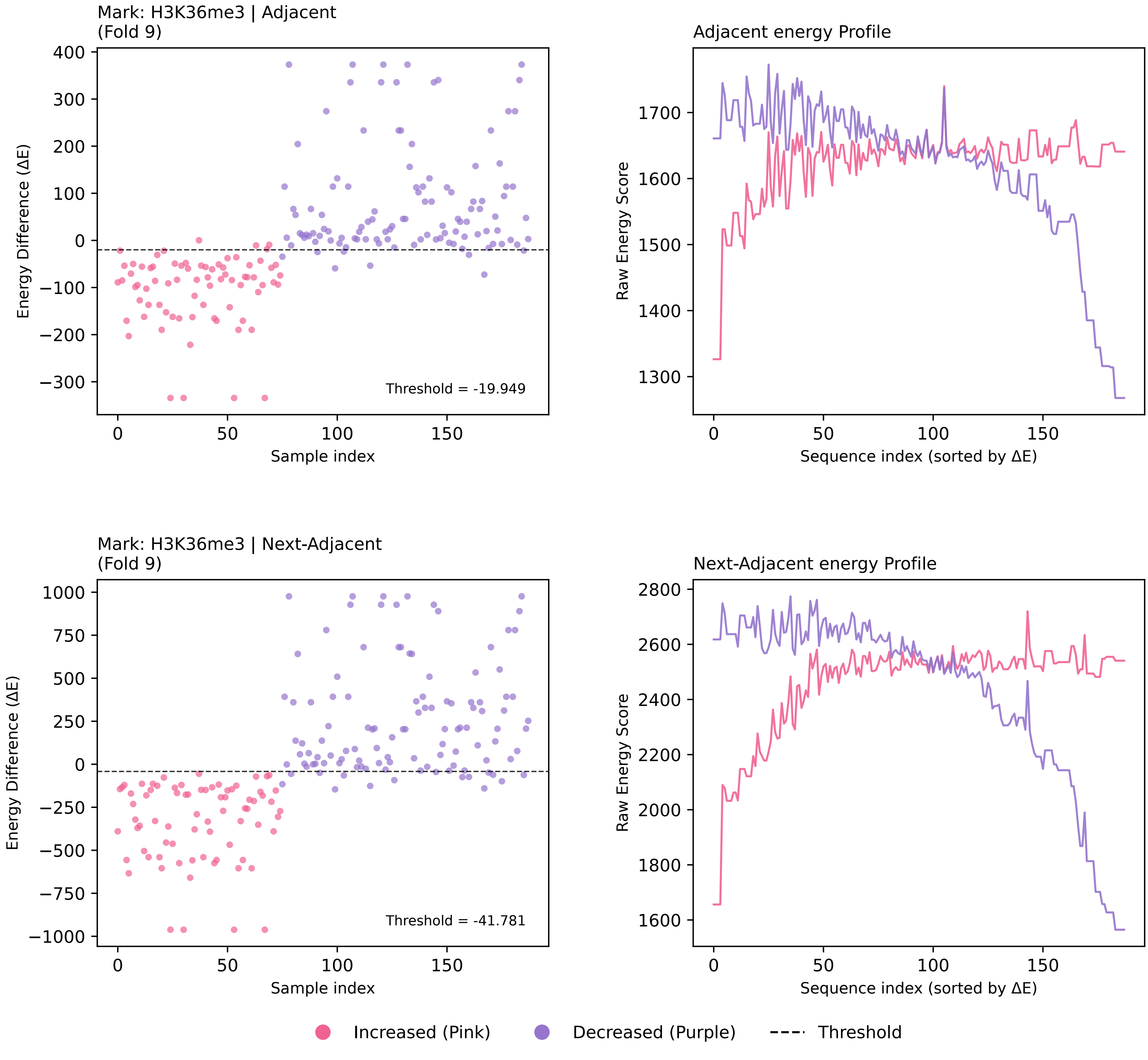

Figure S_Core_Remain_H3K36me3 (Fold 9). Top: Adjacent; Bottom: Next-Adjacent.
Left panels: Scatter plots of energy differences (ΔE); Right panels: Raw energy score profile curves along the sorted sequences.

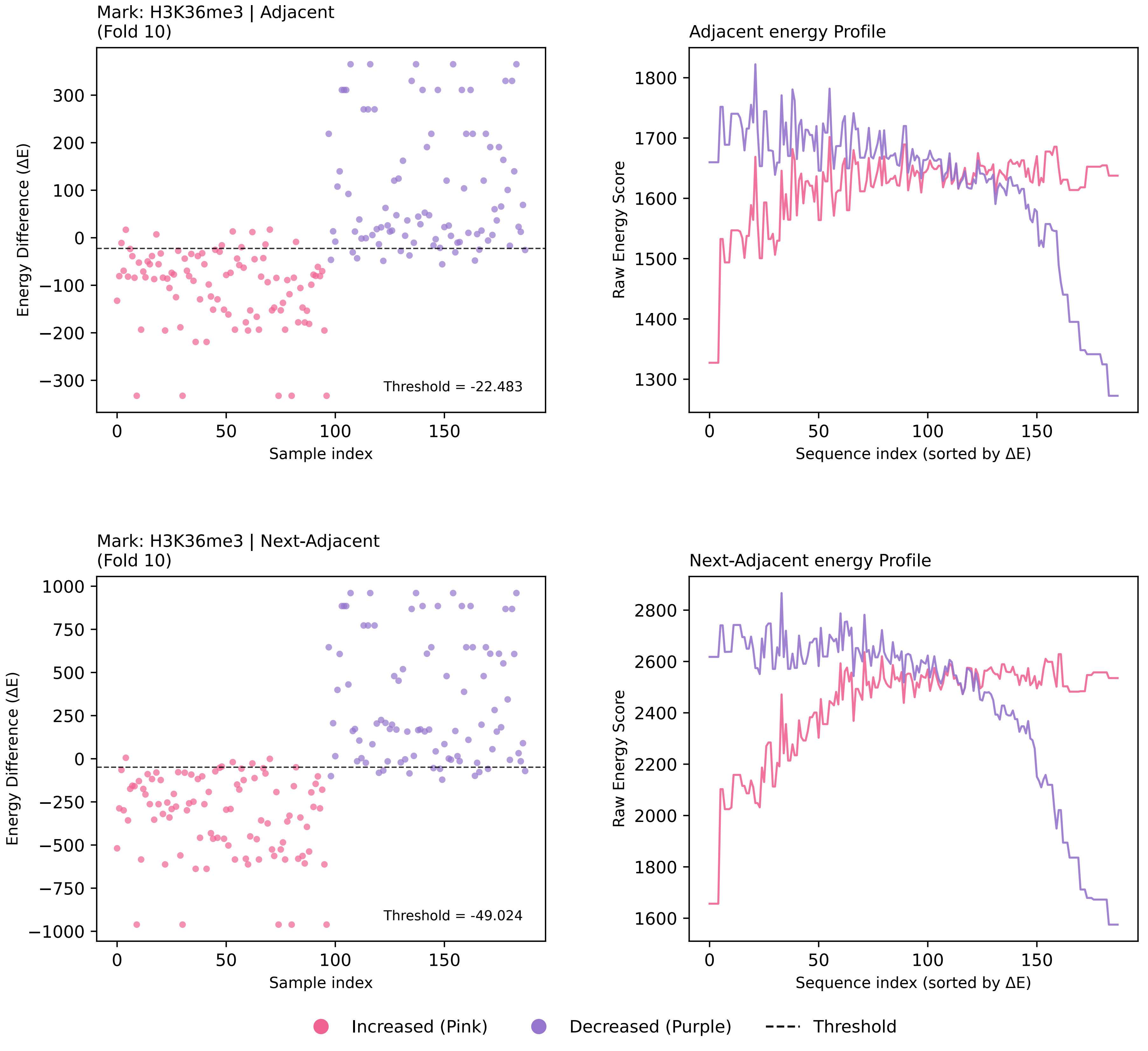

Figure S_Core_Remain_H3K36me3 (Fold 10). Top: Adjacent; Bottom: Next-Adjacent.
Left panels: Scatter plots of energy differences (ΔE); Right panels: Raw energy score profile curves along the sorted sequences.
