# Supplementary material for: An Energy Model Based on Molecular Structure for Predicting Histone Modification Levels at lncRNA Promoter Regions in HepG2 Cells
Source: Int J Mol Sci. 2026 Jun 23;27(13):5653. doi: 10.3390/ijms27135653 (PMC13361589; doi:10.3390/ijms27135653)
Supplement: Supplementary file 1 [file ijms-27-05653-s001.zip › Figure_S1_H2AFZ_Report.pdf]

Performance Metrics: H2AFZ
(Folds 1 to 10)

Table S1. Supplementary table showing per-fold quantitative metrics for H2AFZ. All values are presented as mean ± confidence interval

Model	Fold	Sn (%)	Sp (%)	Ac (%)	MCC	auROC
Adjacent	1	95.489	79.231	86.466	0.758	0.943
Adjacent	2	97.794	82.677	87.5	0.817	0.95
Adjacent	3	97.6	71.739	88.4	0.711	0.936
Adjacent	4	87.77	87.903	83.094	0.756	0.957
Adjacent	5	83.077	84.211	84.615	0.673	0.942
Adjacent	6	96.97	83.206	89.773	0.81	0.962
Adjacent	7	90.083	77.465	90.496	0.675	0.935
Adjacent	8	95.0	82.114	83.571	0.782	0.95
Adjacent	9	91.489	73.77	77.66	0.668	0.939
Adjacent	10	85.593	86.897	96.186	0.724	0.944
Next-Adjacent	1	95.489	92.308	92.857	0.879	0.979
Next-Adjacent	2	91.912	92.126	88.971	0.84	0.975
Next-Adjacent	3	93.6	88.406	95.6	0.819	0.973
Next-Adjacent	4	94.245	91.129	87.77	0.855	0.983
Next-Adjacent	5	89.231	92.481	91.923	0.818	0.977
Next-Adjacent	6	95.455	93.13	93.939	0.886	0.986
Next-Adjacent	7	91.736	92.254	100.0	0.839	0.98
Next-Adjacent	8	95.0	92.683	88.214	0.878	0.981
Next-Adjacent	9	92.908	90.164	85.461	0.832	0.978
Next-Adjacent	10	96.61	91.034	104.237	0.872	0.981

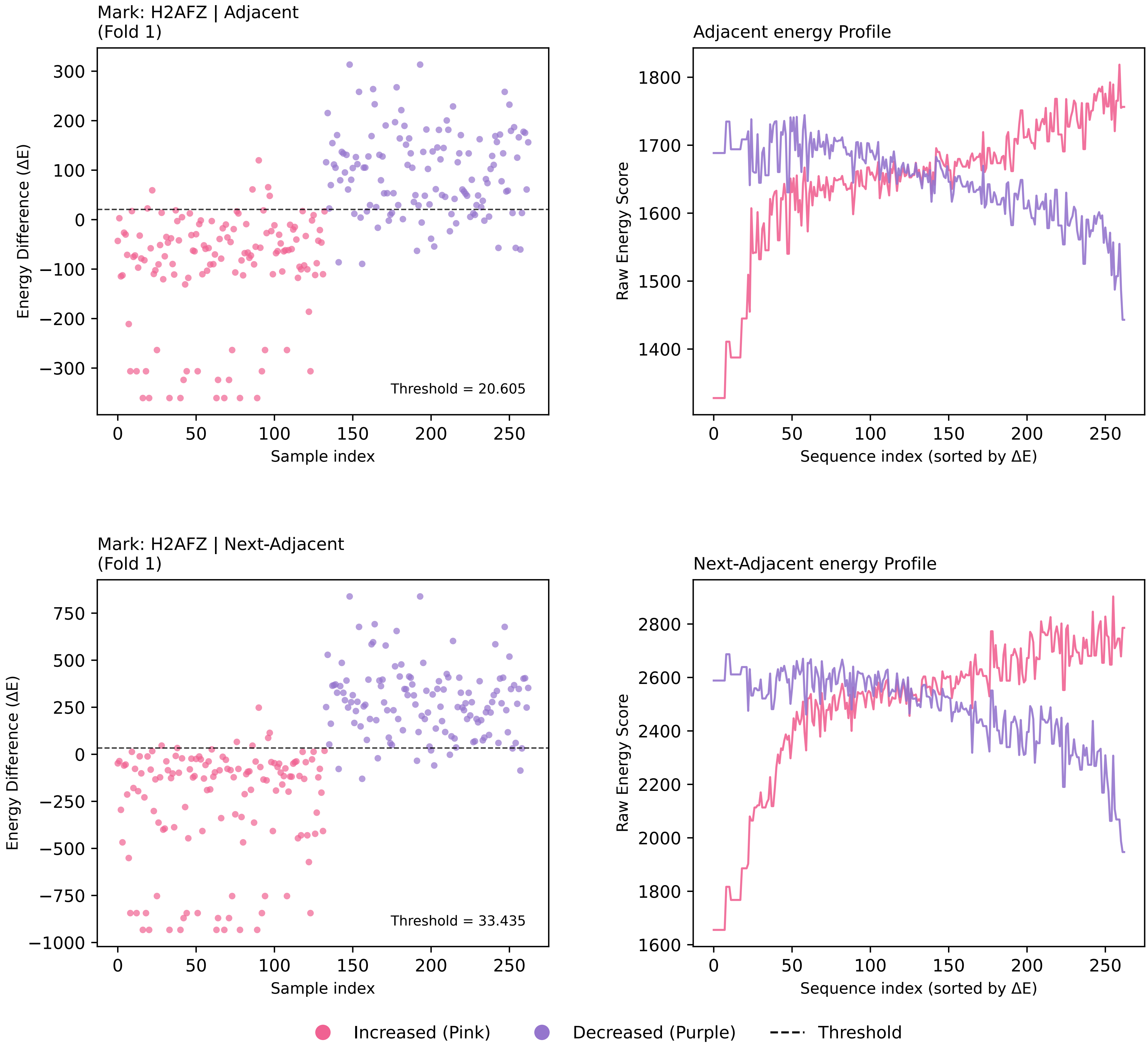

Figure S1 (Fold 1). Top: Adjacent; Bottom: Next-Adjacent.
Left panels: Scatter plots of energy differences (ΔE); Right panels: Raw energy score profile curves along the sorted sequences.

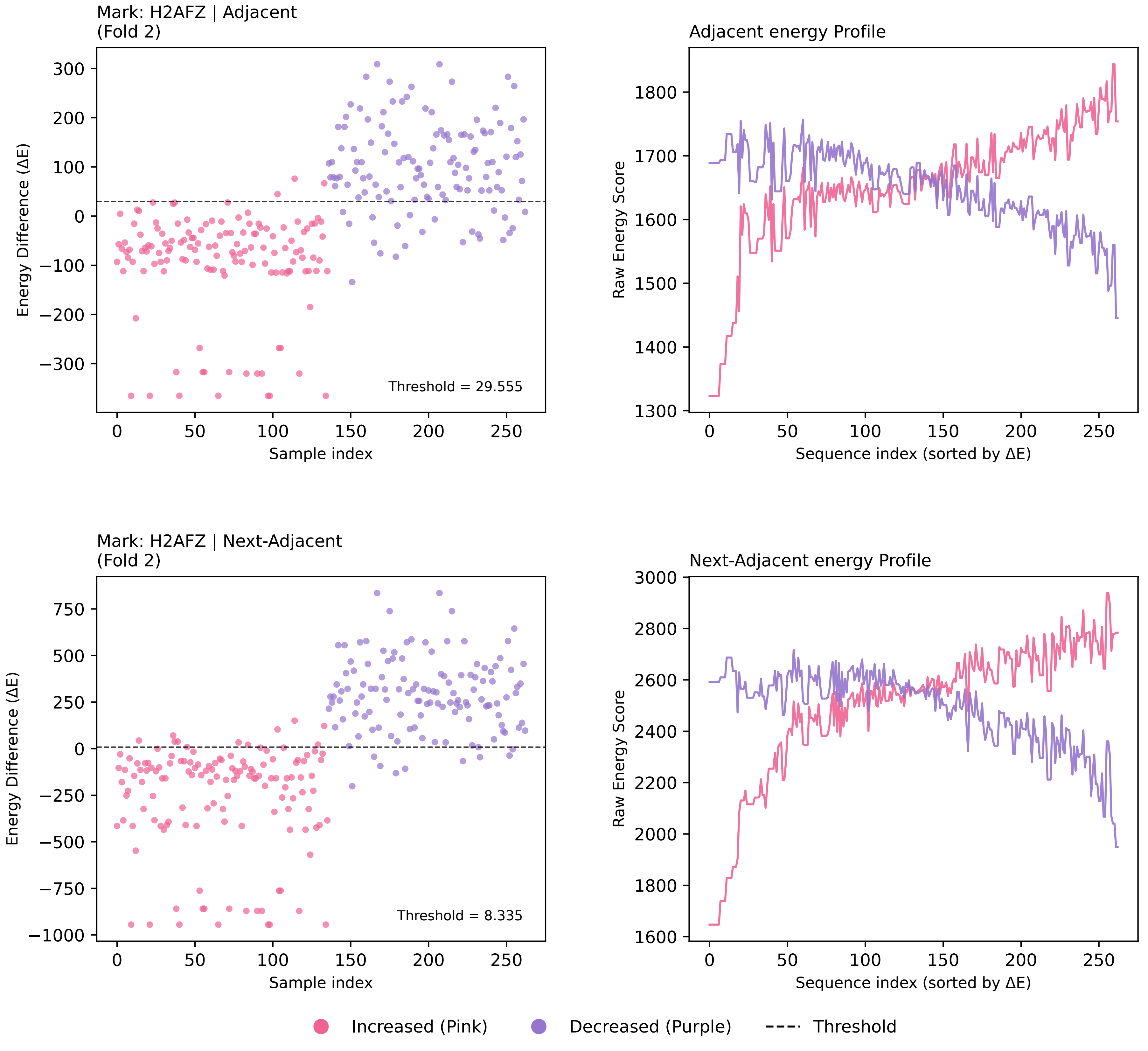

Figure S1 (Fold 2). Top: Adjacent; Bottom: Next-Adjacent.
Left panels: Scatter plots of energy differences (ΔE); Right panels: Raw energy score profile curves along the sorted sequences.

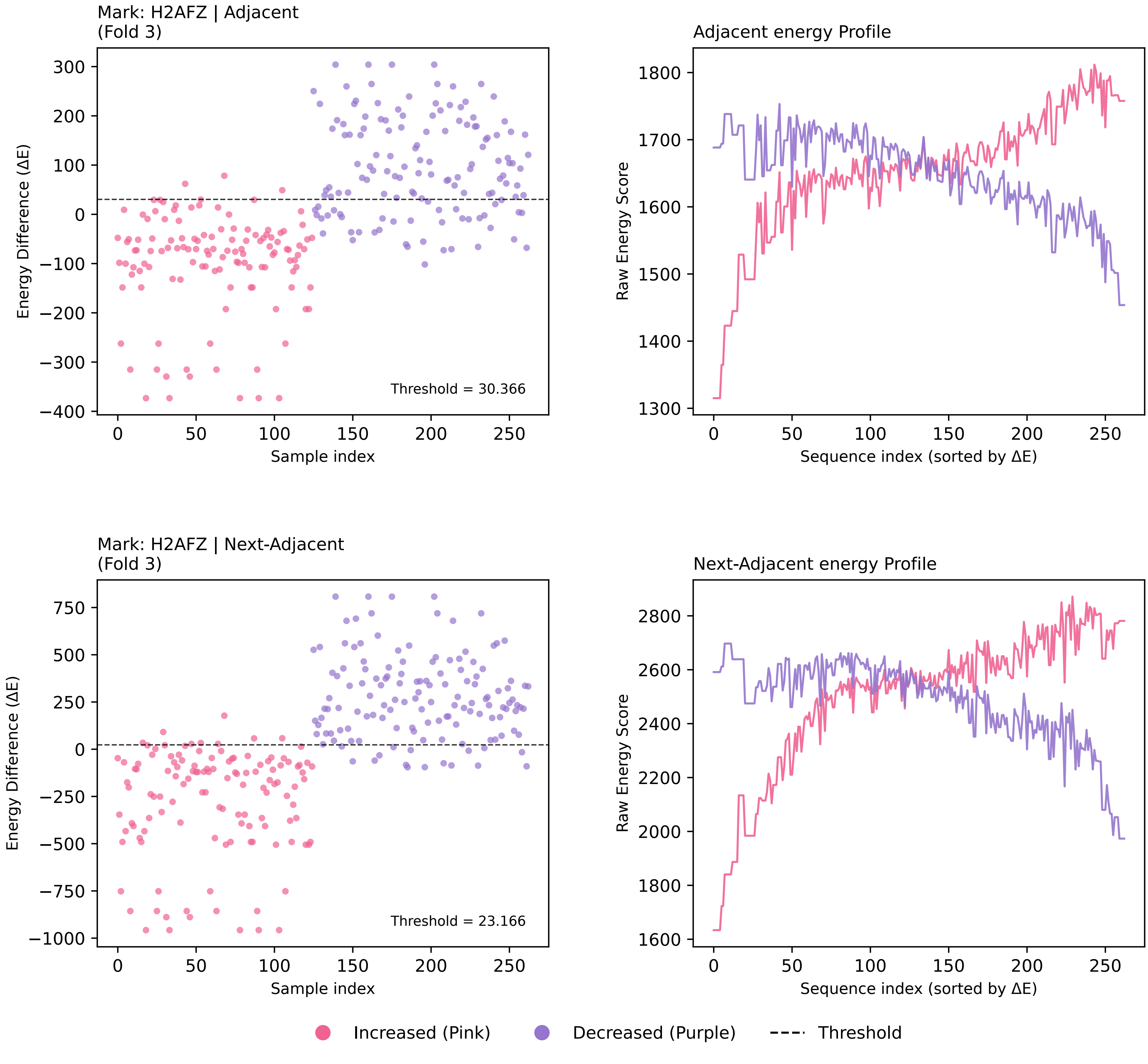

Figure S1 (Fold 3). Top: Adjacent; Bottom: Next-Adjacent.
Left panels: Scatter plots of energy differences (ΔE); Right panels: Raw energy score profile curves along the sorted sequences.

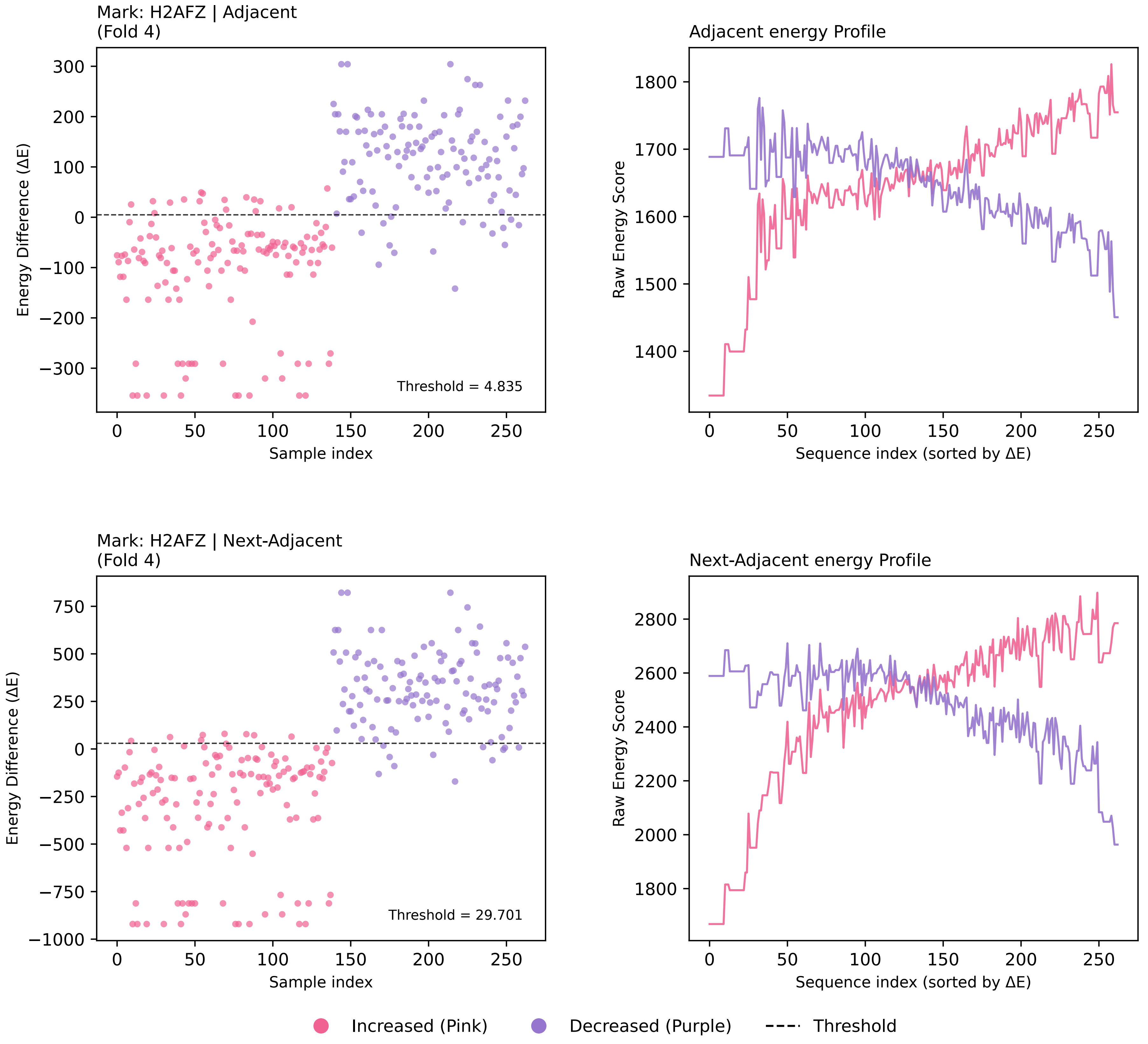

Figure S1 (Fold 4). Top: Adjacent; Bottom: Next-Adjacent.
Left panels: Scatter plots of energy differences (ΔE); Right panels: Raw energy score profile curves along the sorted sequences.

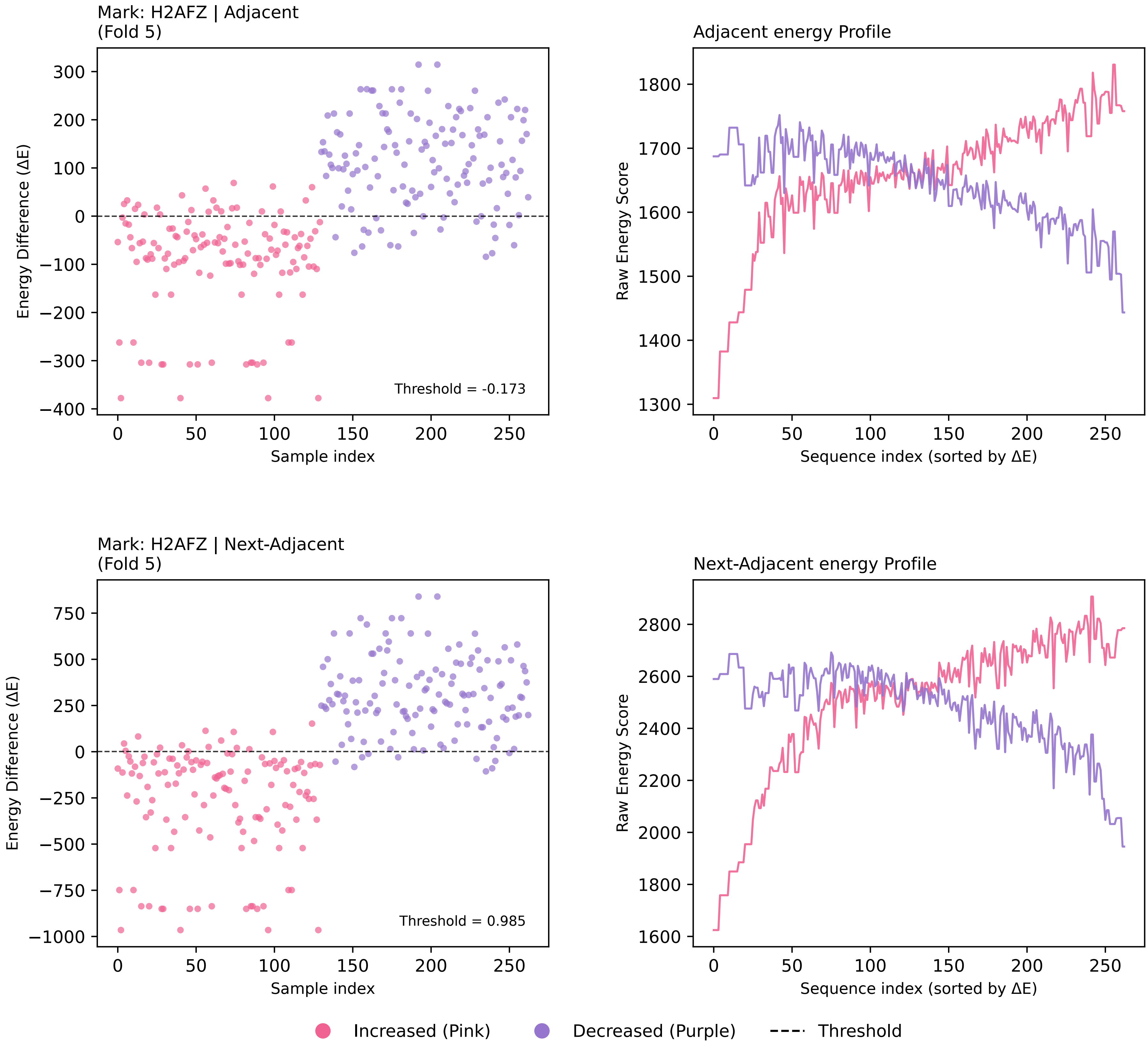

Figure S1 (Fold 5). Top: Adjacent; Bottom: Next-Adjacent.
Left panels: Scatter plots of energy differences (ΔE); Right panels: Raw energy score profile curves along the sorted sequences.

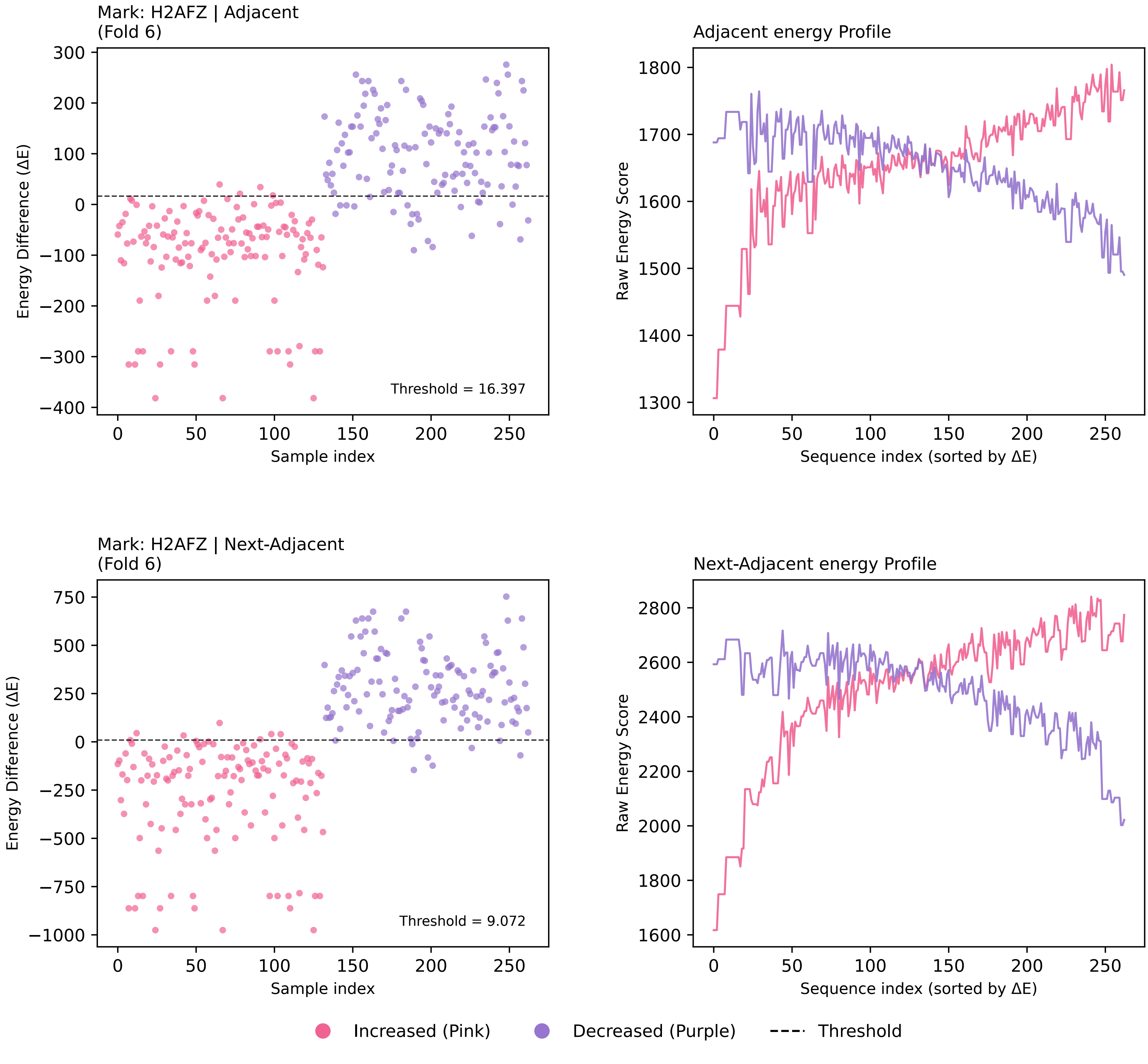

Figure S1 (Fold 6). Top: Adjacent; Bottom: Next-Adjacent.
Left panels: Scatter plots of energy differences (ΔE); Right panels: Raw energy score profile curves along the sorted sequences.

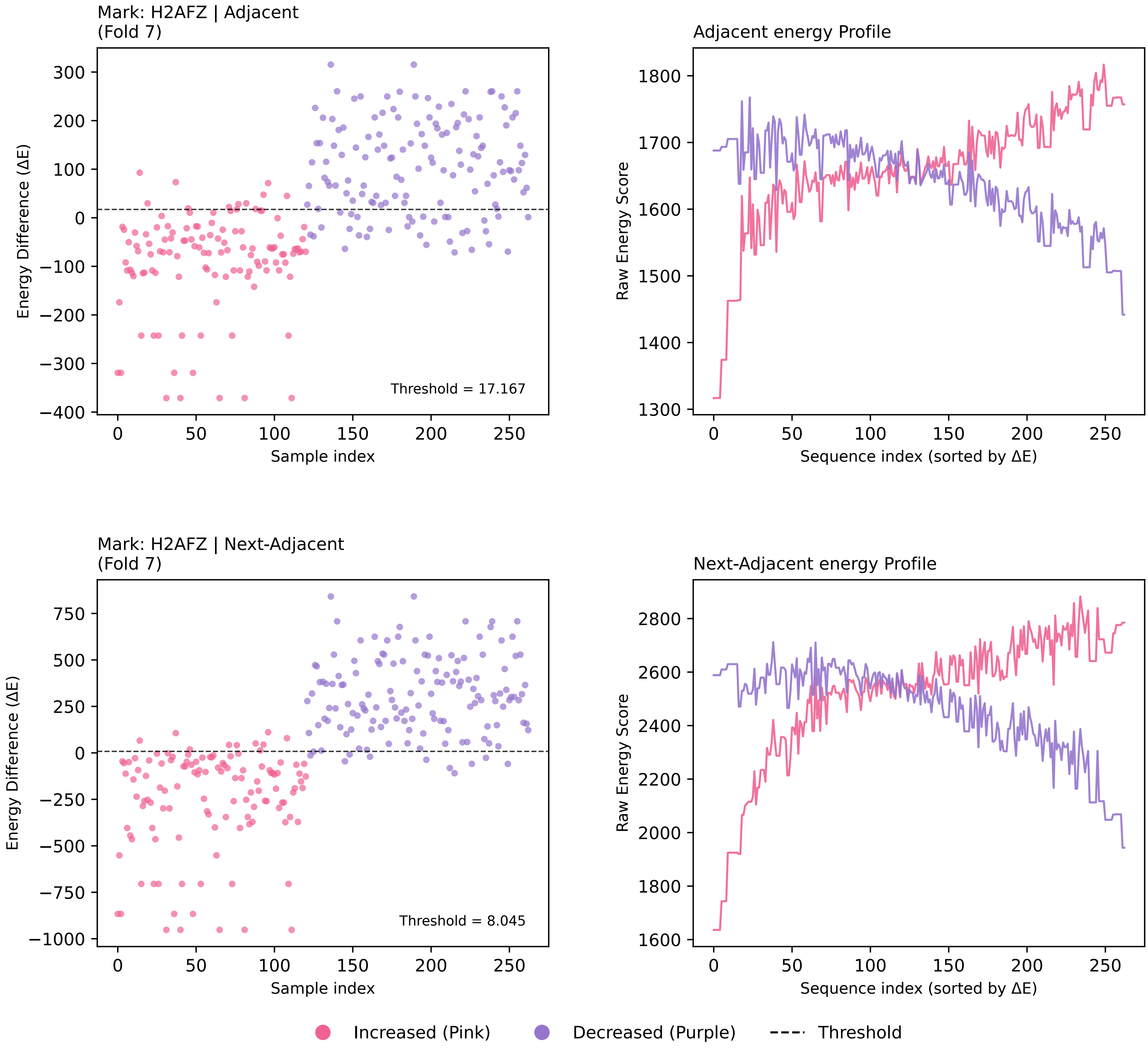

Figure S1 (Fold 7). Top: Adjacent; Bottom: Next-Adjacent.
Left panels: Scatter plots of energy differences (ΔE); Right panels: Raw energy score profile curves along the sorted sequences.

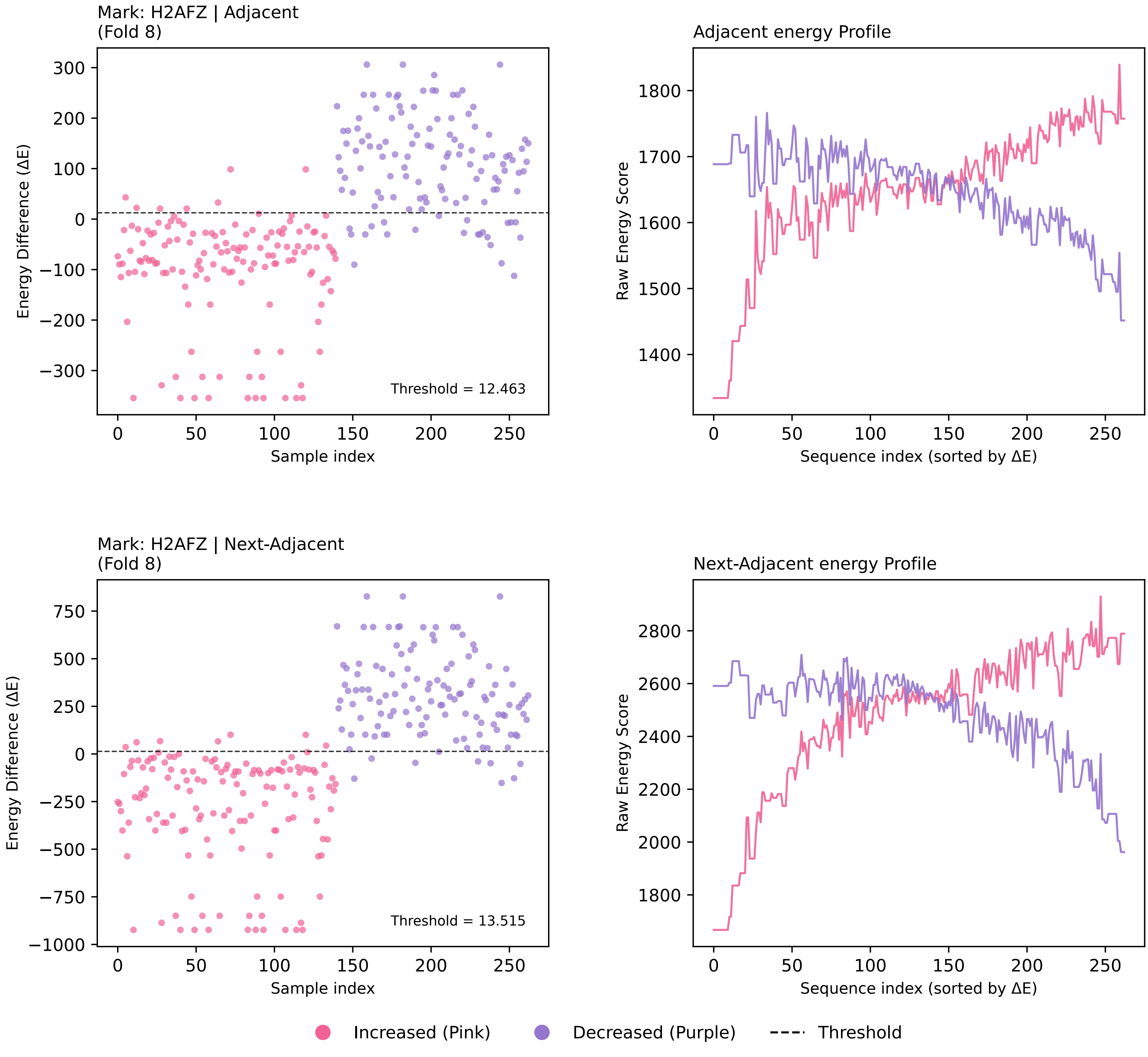

Figure S1 (Fold 8). Top: Adjacent; Bottom: Next-Adjacent.
Left panels: Scatter plots of energy differences (ΔE); Right panels: Raw energy score profile curves along the sorted sequences.

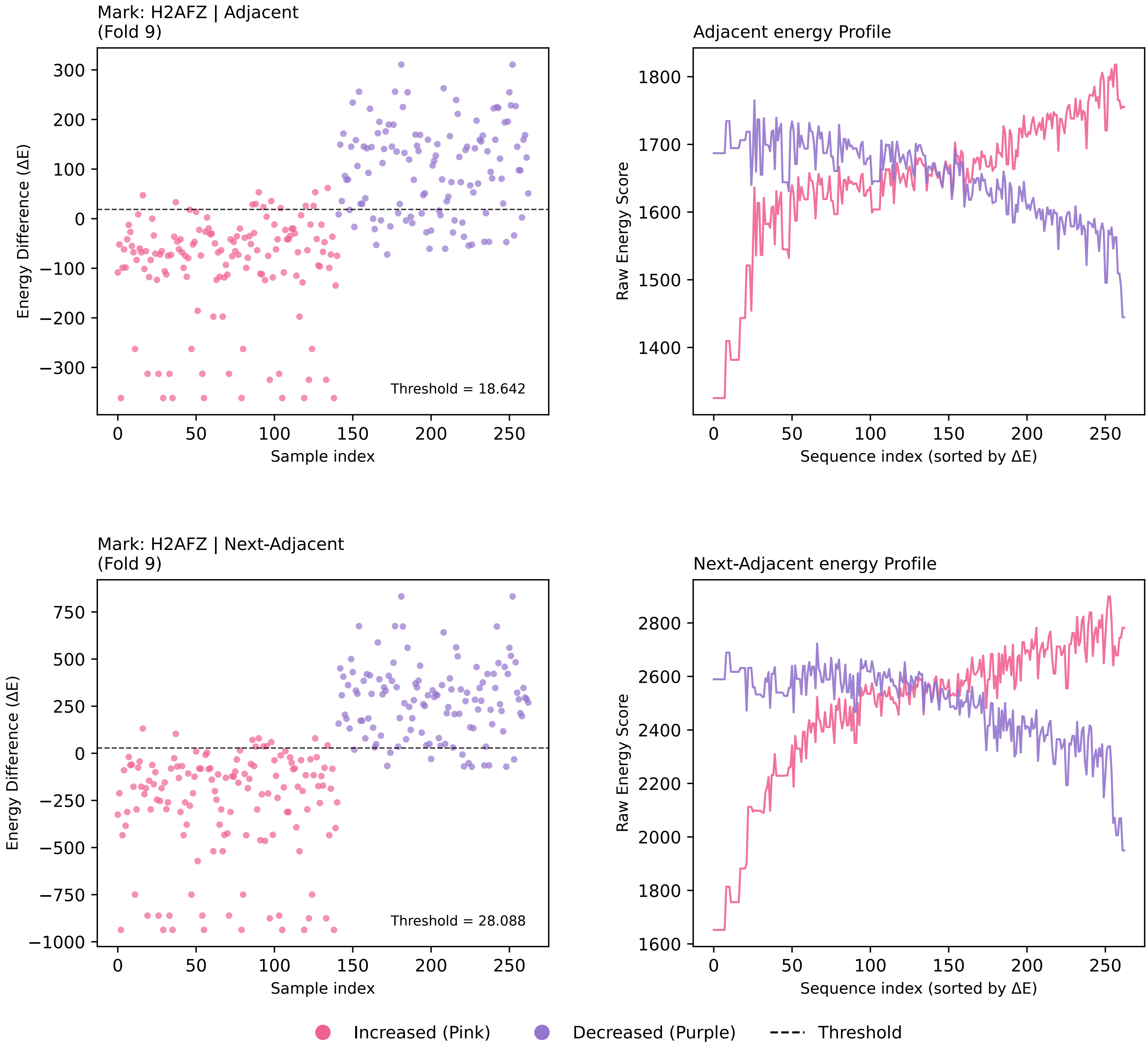

Figure S1 (Fold 9). Top: Adjacent; Bottom: Next-Adjacent.
Left panels: Scatter plots of energy differences (ΔE); Right panels: Raw energy score profile curves along the sorted sequences.

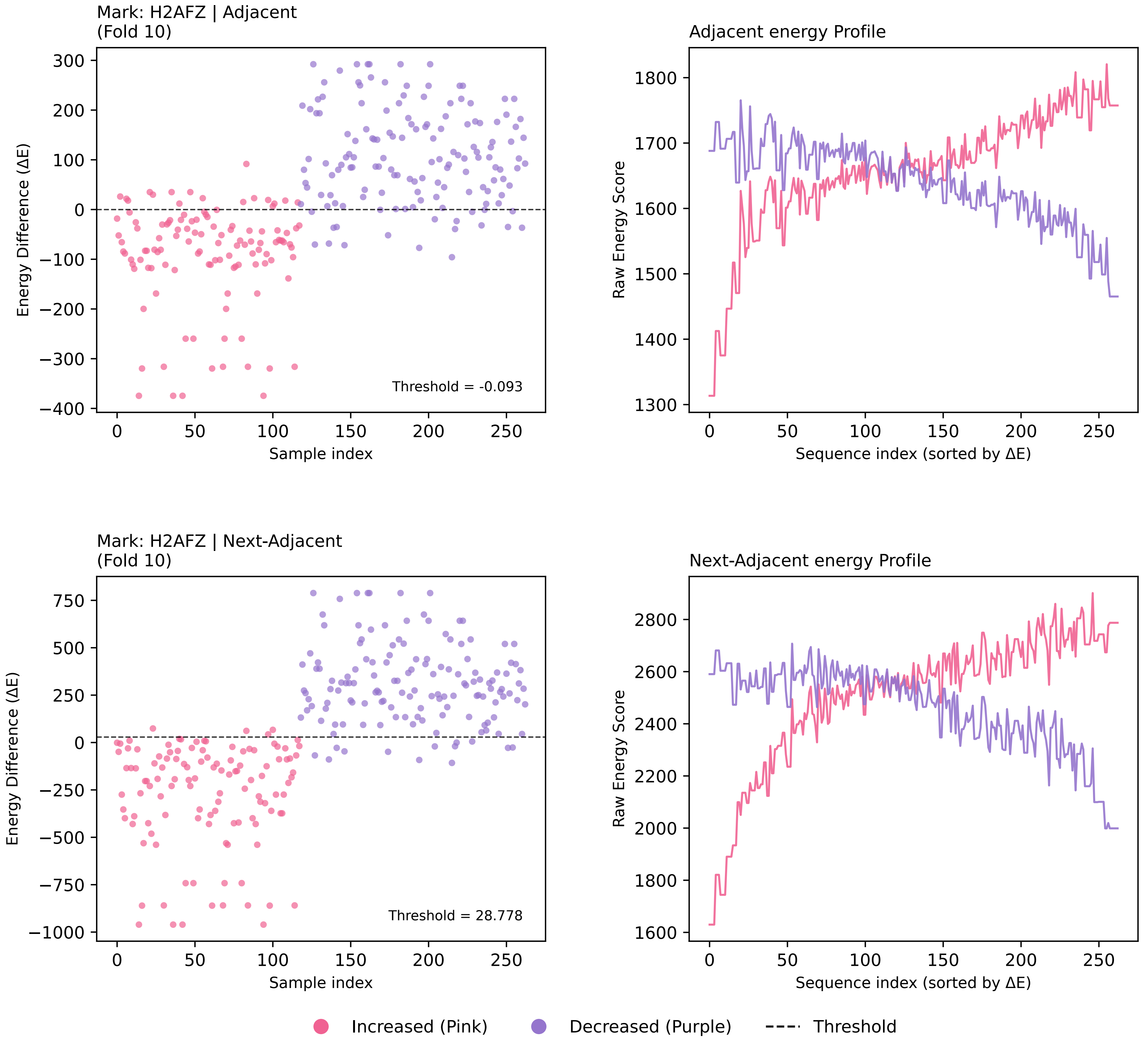

Figure S1 (Fold 10). Top: Adjacent; Bottom: Next-Adjacent.
Left panels: Scatter plots of energy differences (ΔE); Right panels: Raw energy score profile curves along the sorted sequences.
