# Supplementary material for: An Energy Model Based on Molecular Structure for Predicting Histone Modification Levels at lncRNA Promoter Regions in HepG2 Cells
Source: Int J Mol Sci. 2026 Jun 23;27(13):5653. doi: 10.3390/ijms27135653 (PMC13361589; doi:10.3390/ijms27135653)
Supplement: Supplementary file 1 [file ijms-27-05653-s001.zip › Figure_S2_H3K4me1_Report.pdf]

Performance Metrics: H3K4me1
(Folds 1 to 10)

Table S2. Supplementary table showing per-fold quantitative metrics for H3K4me1. All values are presented as mean ± confidence interval

Model	Fold	Sn (%)	Sp (%)	Ac (%)	MCC	auROC
Adjacent	1	81.702	89.352	81.915	0.711	0.95
Adjacent	2	88.182	81.818	87.045	0.701	0.938
Adjacent	3	84.553	88.78	79.268	0.731	0.946
Adjacent	4	73.113	92.05	88.443	0.668	0.918
Adjacent	5	86.321	85.714	91.274	0.72	0.948
Adjacent	6	89.27	83.41	83.476	0.729	0.952
Adjacent	7	78.448	93.119	82.974	0.721	0.947
Adjacent	8	83.019	92.017	93.16	0.756	0.96
Adjacent	9	85.95	86.058	79.959	0.719	0.948
Adjacent	10	83.654	81.818	89.423	0.653	0.912
Next-Adjacent	1	91.064	90.741	87.234	0.818	0.976
Next-Adjacent	2	93.182	84.848	91.136	0.782	0.967
Next-Adjacent	3	89.837	94.146	84.146	0.837	0.981
Next-Adjacent	4	88.679	89.54	94.811	0.782	0.966
Next-Adjacent	5	91.981	88.235	95.519	0.801	0.974
Next-Adjacent	6	90.987	94.47	89.485	0.854	0.98
Next-Adjacent	7	88.362	92.202	87.5	0.805	0.976
Next-Adjacent	8	88.208	91.176	95.283	0.795	0.978
Next-Adjacent	9	88.843	89.904	83.058	0.786	0.972
Next-Adjacent	10	88.462	85.537	93.99	0.738	0.959

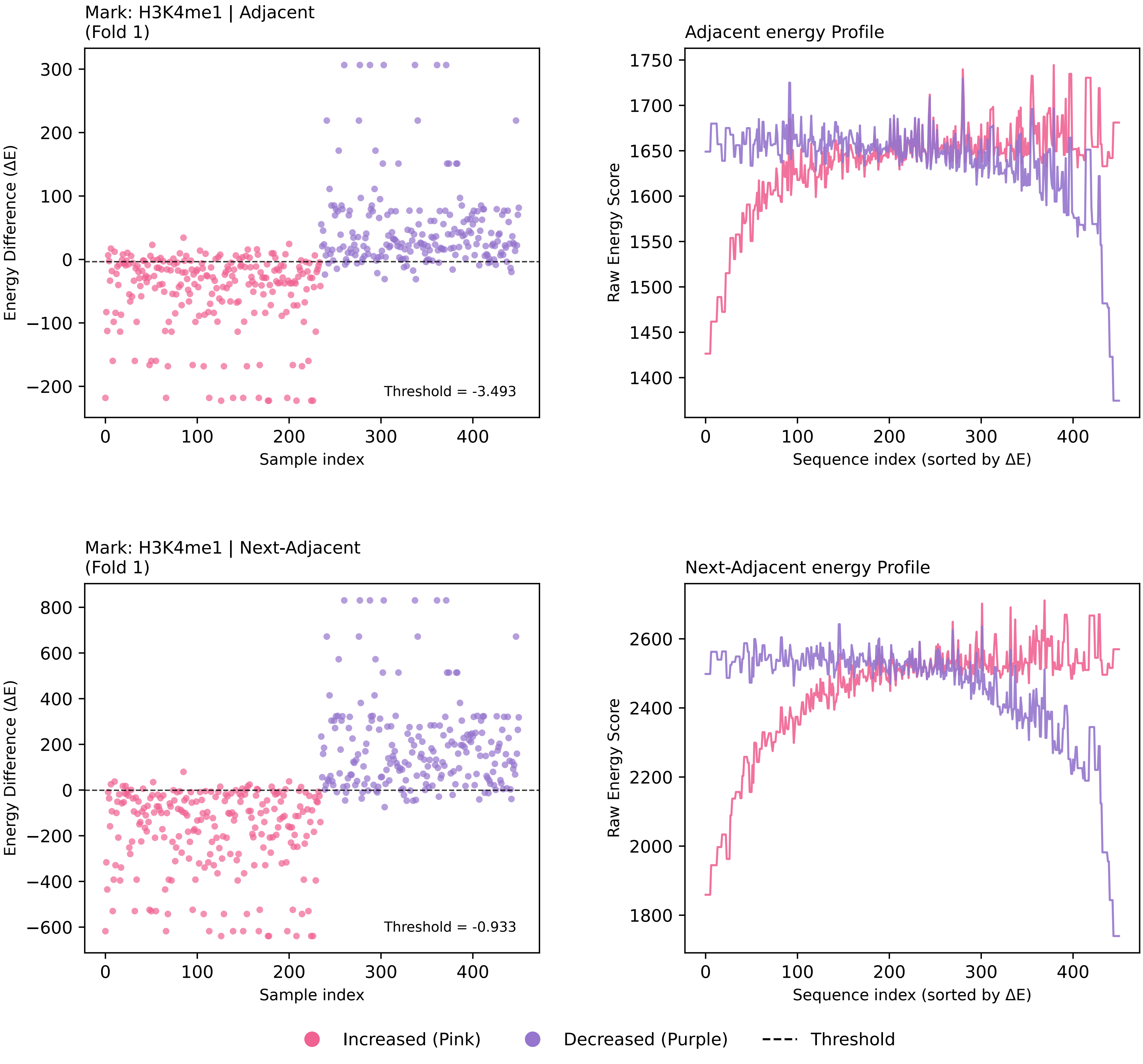

Figure S2 (Fold 1). Top: Adjacent; Bottom: Next-Adjacent.
Left panels: Scatter plots of energy differences (ΔE); Right panels: Raw energy score profile curves along the sorted sequences.

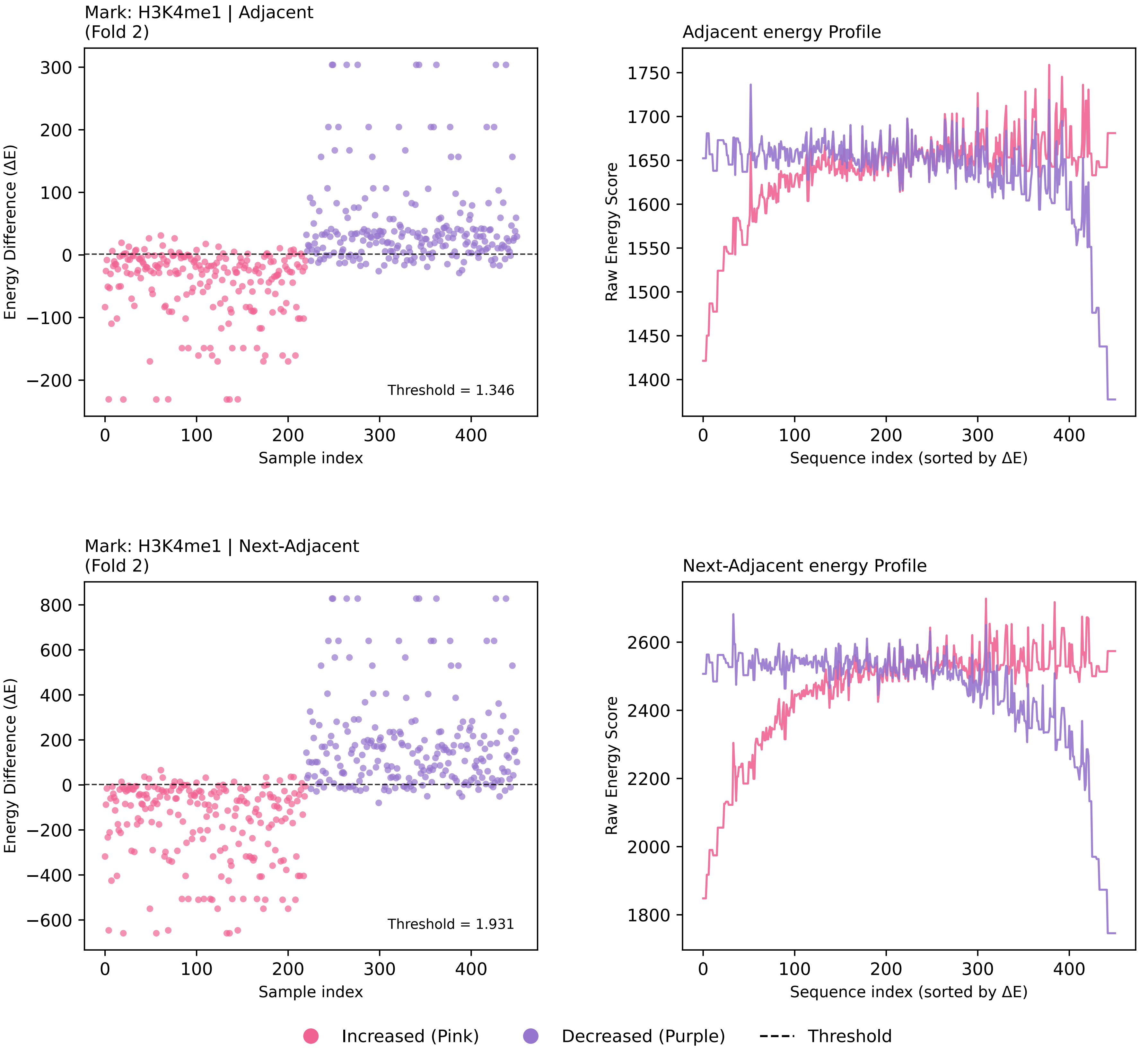

Figure S2 (Fold 2). Top: Adjacent; Bottom: Next-Adjacent.
Left panels: Scatter plots of energy differences (ΔE); Right panels: Raw energy score profile curves along the sorted sequences.

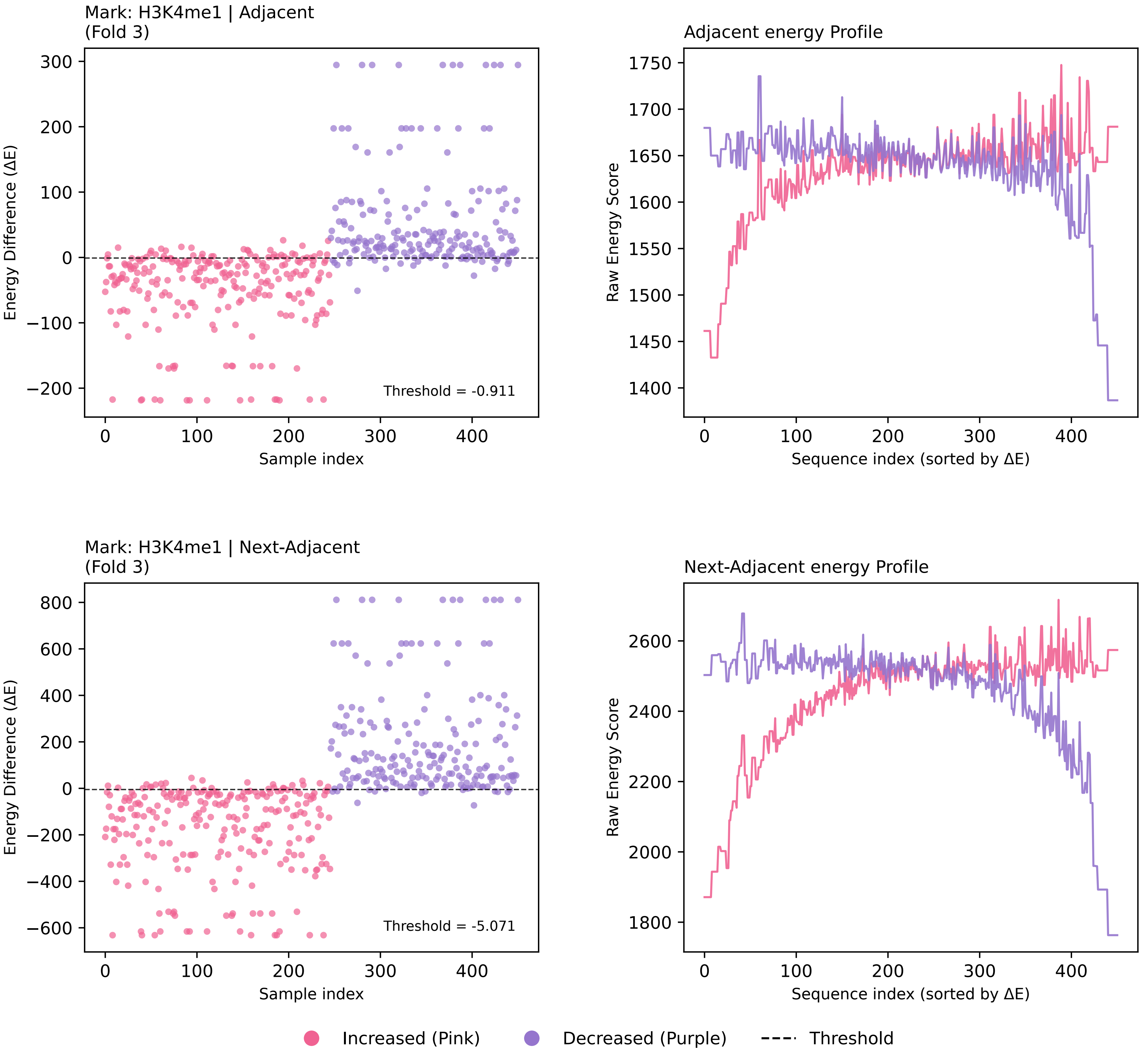

Figure S2 (Fold 3). Top: Adjacent; Bottom: Next-Adjacent.
Left panels: Scatter plots of energy differences (ΔE); Right panels: Raw energy score profile curves along the sorted sequences.

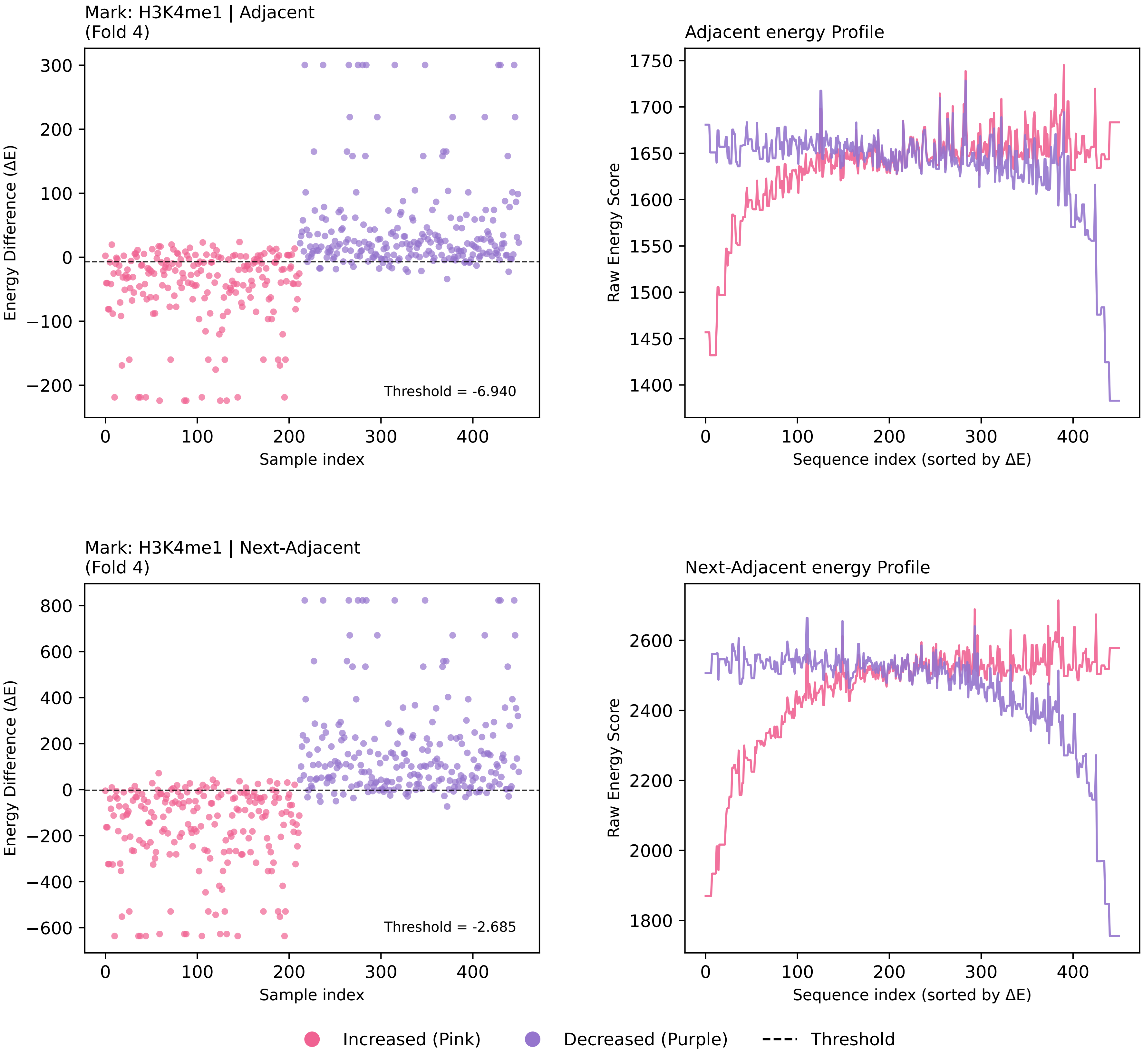

Figure S2 (Fold 4). Top: Adjacent; Bottom: Next-Adjacent.
Left panels: Scatter plots of energy differences (ΔE); Right panels: Raw energy score profile curves along the sorted sequences.

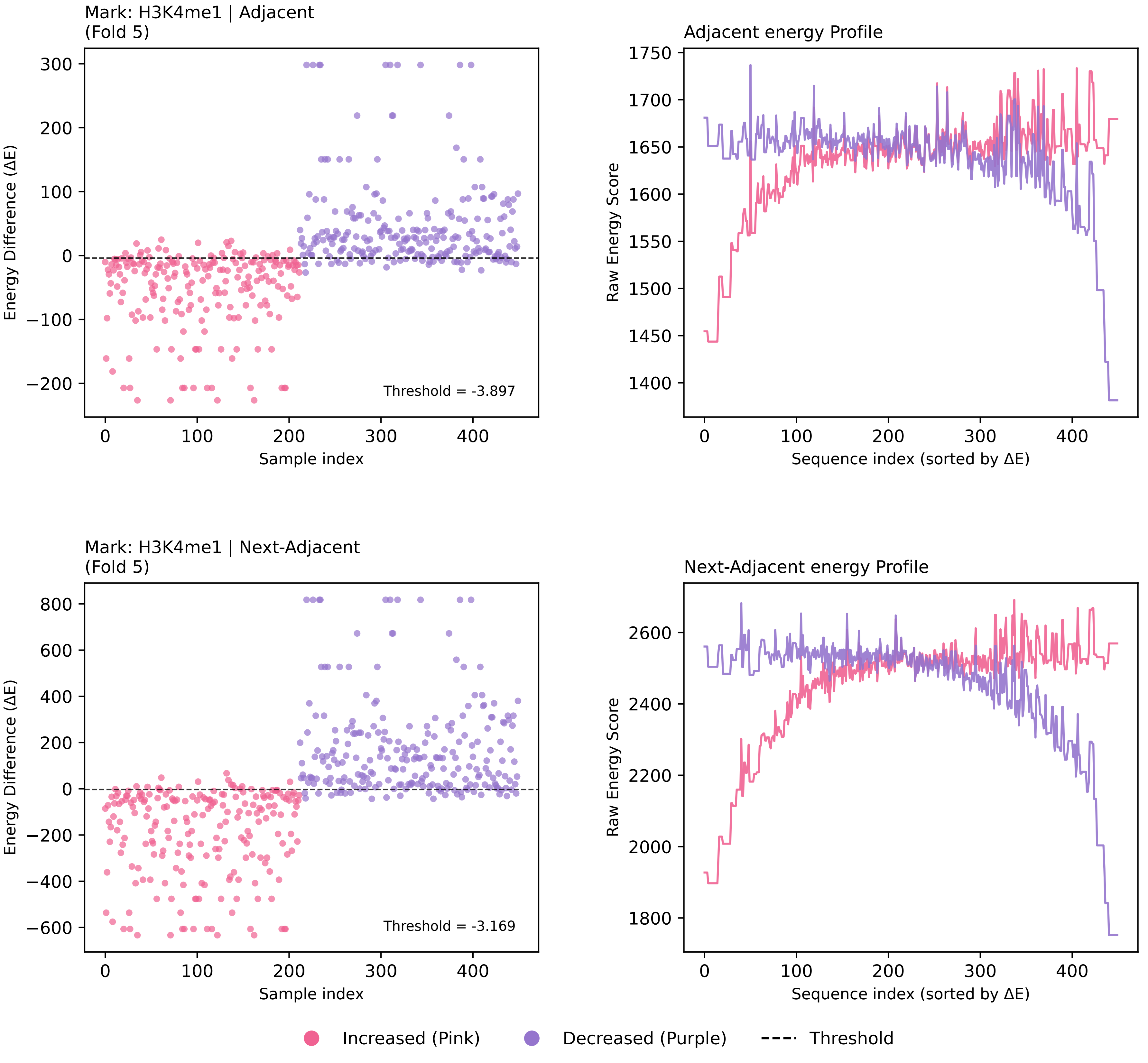

Figure S2 (Fold 5). Top: Adjacent; Bottom: Next-Adjacent.
Left panels: Scatter plots of energy differences (ΔE); Right panels: Raw energy score profile curves along the sorted sequences.

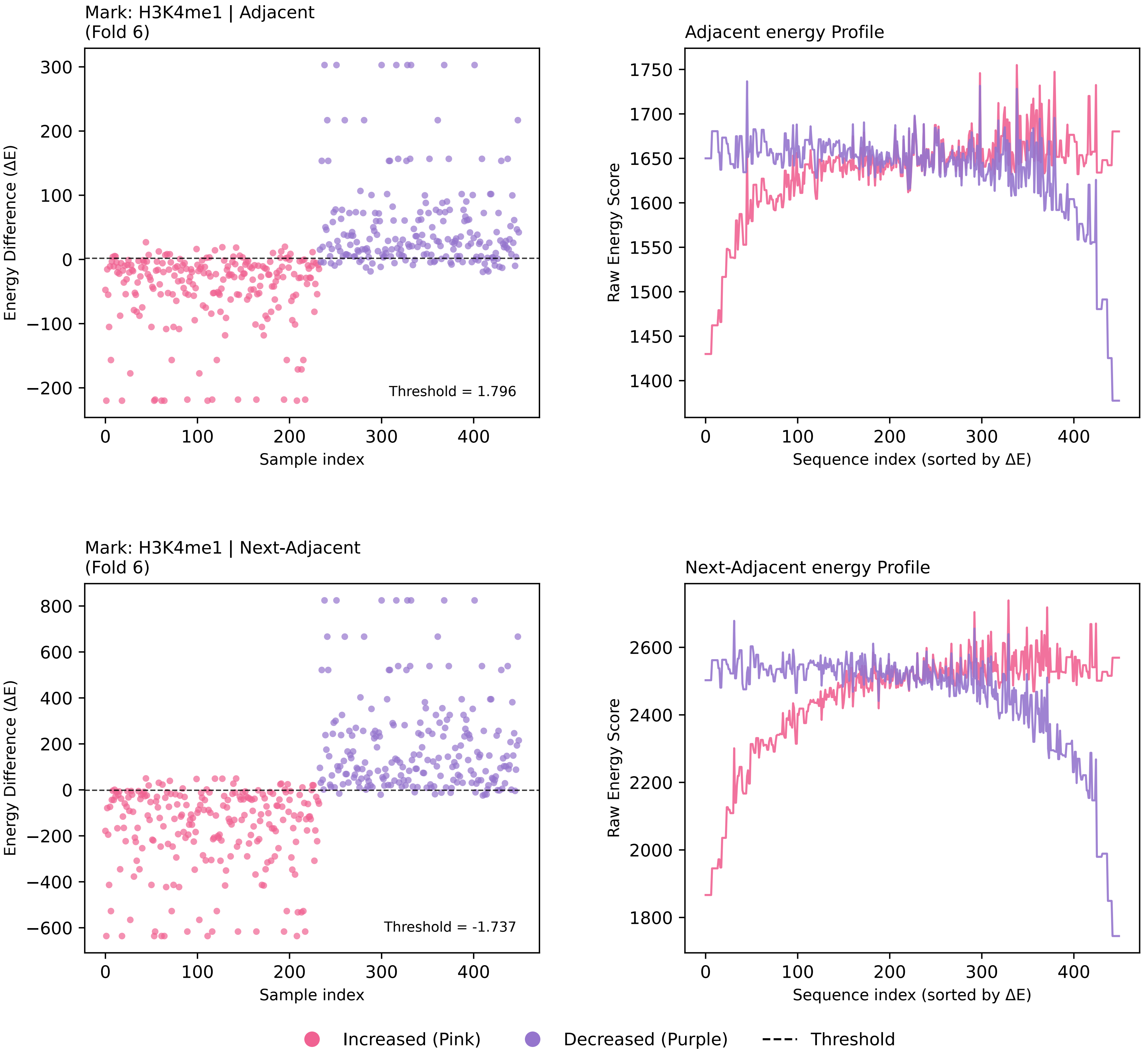

Figure S2 (Fold 6). Top: Adjacent; Bottom: Next-Adjacent.
Left panels: Scatter plots of energy differences (ΔE); Right panels: Raw energy score profile curves along the sorted sequences.

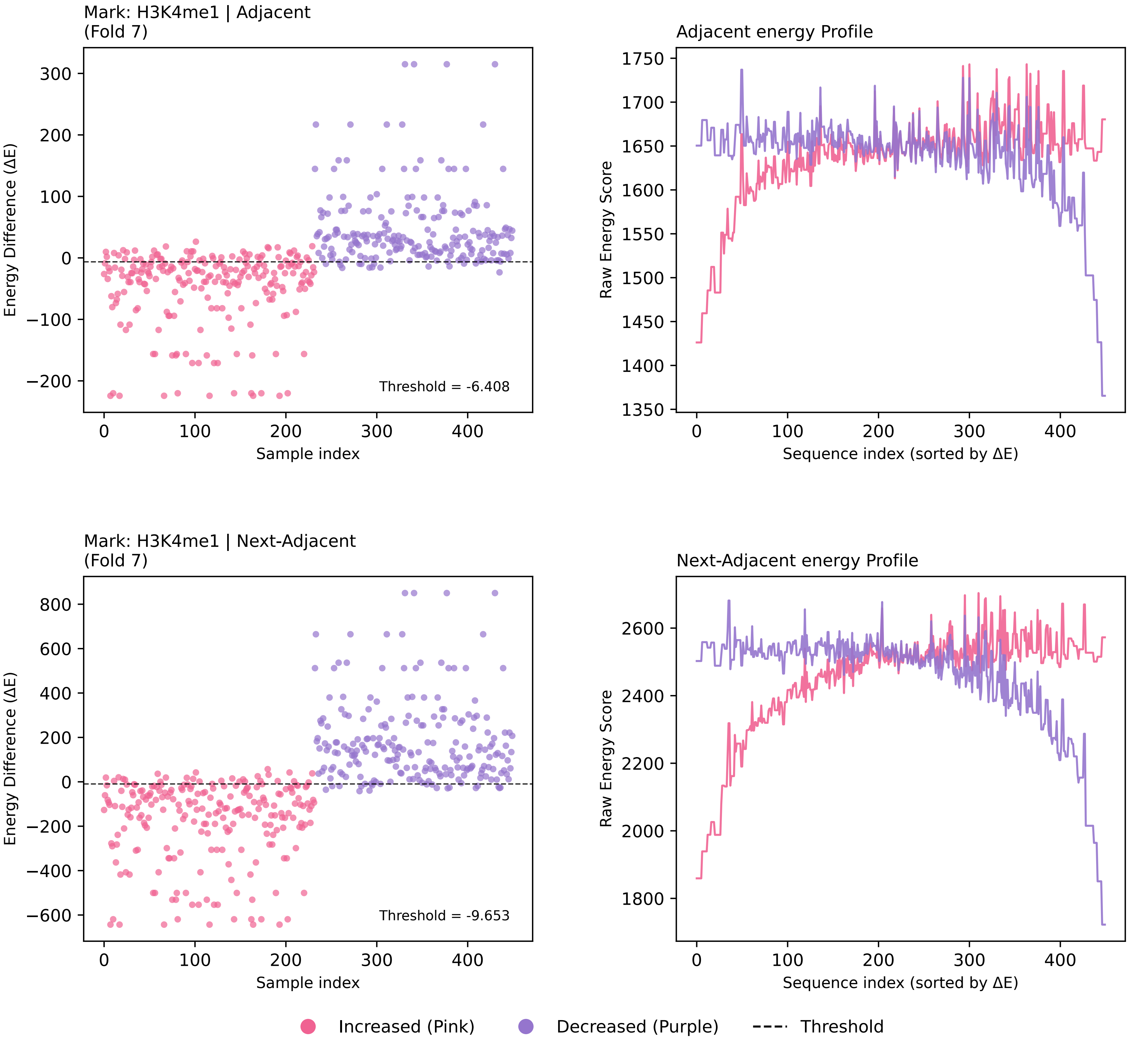

Figure S2 (Fold 7). Top: Adjacent; Bottom: Next-Adjacent.
Left panels: Scatter plots of energy differences (ΔE); Right panels: Raw energy score profile curves along the sorted sequences.

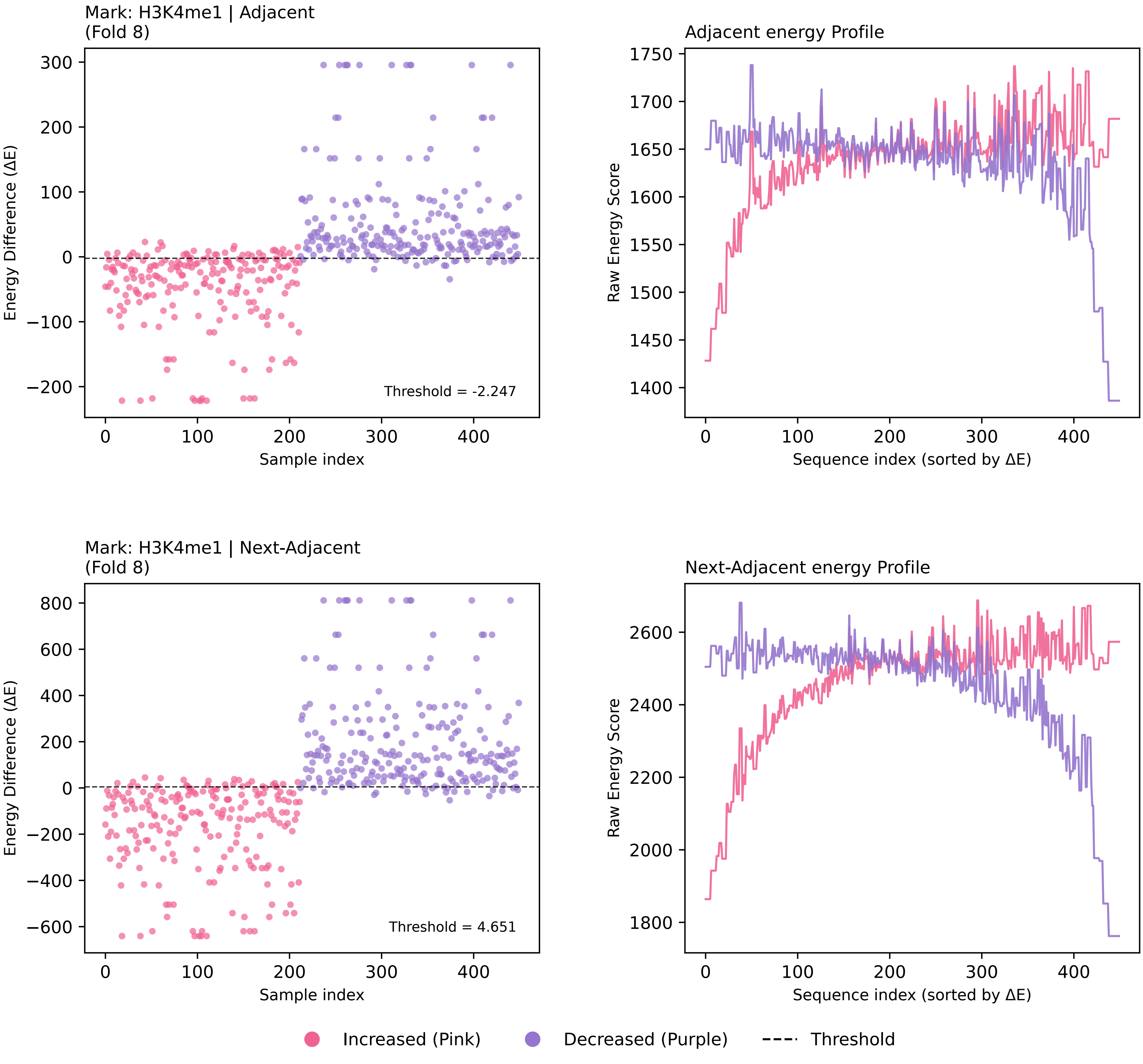

Figure S2 (Fold 8). Top: Adjacent; Bottom: Next-Adjacent.
Left panels: Scatter plots of energy differences (ΔE); Right panels: Raw energy score profile curves along the sorted sequences.

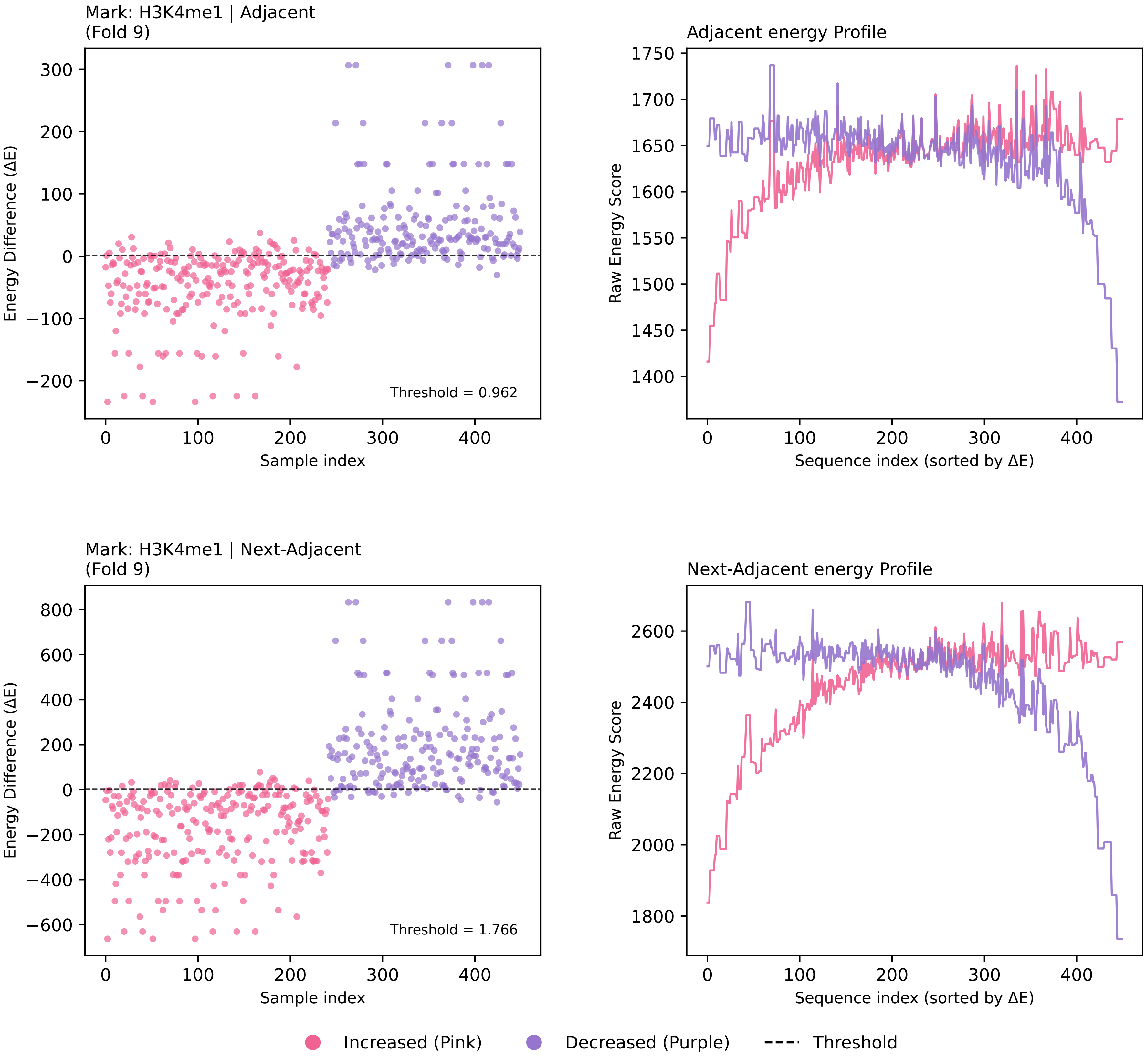

Figure S2 (Fold 9). Top: Adjacent; Bottom: Next-Adjacent.
Left panels: Scatter plots of energy differences (ΔE); Right panels: Raw energy score profile curves along the sorted sequences.

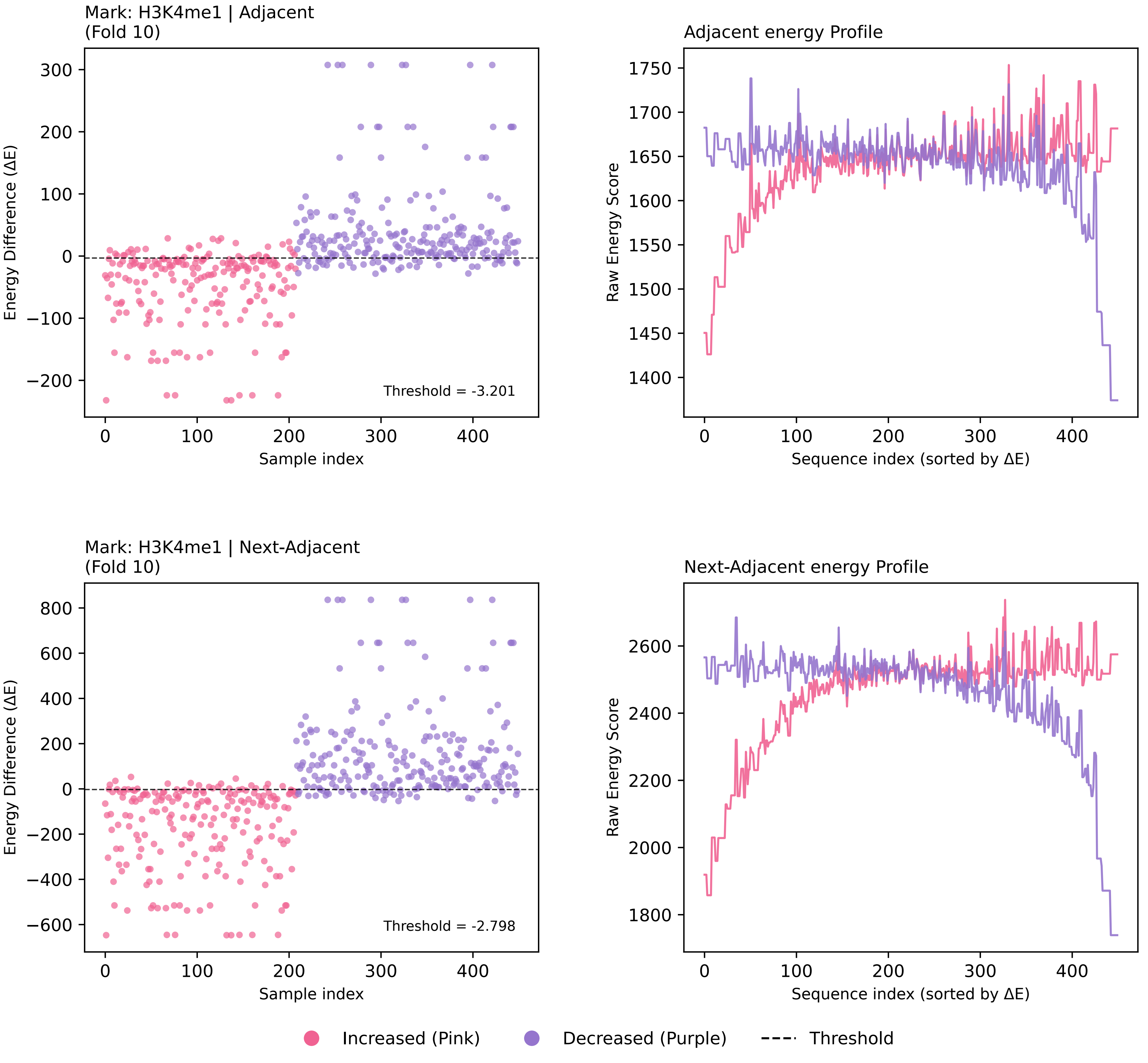

Figure S2 (Fold 10). Top: Adjacent; Bottom: Next-Adjacent.
Left panels: Scatter plots of energy differences (ΔE); Right panels: Raw energy score profile curves along the sorted sequences.
