# Supplementary material for: An Energy Model Based on Molecular Structure for Predicting Histone Modification Levels at lncRNA Promoter Regions in HepG2 Cells
Source: Int J Mol Sci. 2026 Jun 23;27(13):5653. doi: 10.3390/ijms27135653 (PMC13361589; doi:10.3390/ijms27135653)
Supplement: Supplementary file 1 [file ijms-27-05653-s001.zip › Figure_S3_H3K4me2_Report.pdf]

Performance Metrics: H3K4me2
(Folds 1 to 10)

Table S3. Supplementary table showing per-fold quantitative metrics for H3K4me2. All values are presented as mean ± confidence interval

Model	Fold	Sn (%)	Sp (%)	Ac (%)	MCC	auROC
Adjacent	1	77.551	91.935	77.551	0.695	0.941
Adjacent	2	78.626	87.857	86.26	0.669	0.926
Adjacent	3	85.034	83.065	77.551	0.681	0.932
Adjacent	4	78.049	84.459	89.837	0.627	0.916
Adjacent	5	90.226	81.159	87.218	0.716	0.939
Adjacent	6	83.465	88.194	91.732	0.718	0.937
Adjacent	7	80.147	90.37	84.926	0.709	0.946
Adjacent	8	88.276	82.54	80.0	0.71	0.945
Adjacent	9	79.452	80.8	74.315	0.601	0.918
Adjacent	10	80.833	88.079	95.833	0.693	0.95
Next-Adjacent	1	87.075	93.548	82.993	0.803	0.978
Next-Adjacent	2	83.206	88.571	88.931	0.72	0.962
Next-Adjacent	3	85.034	91.935	81.293	0.767	0.969
Next-Adjacent	4	85.366	85.135	93.902	0.703	0.95
Next-Adjacent	5	85.714	92.754	90.977	0.787	0.975
Next-Adjacent	6	91.339	91.667	97.638	0.83	0.978
Next-Adjacent	7	88.971	92.593	90.441	0.816	0.977
Next-Adjacent	8	88.276	91.27	83.793	0.794	0.976
Next-Adjacent	9	81.507	90.4	79.452	0.717	0.962
Next-Adjacent	10	85.0	94.04	101.667	0.798	0.979

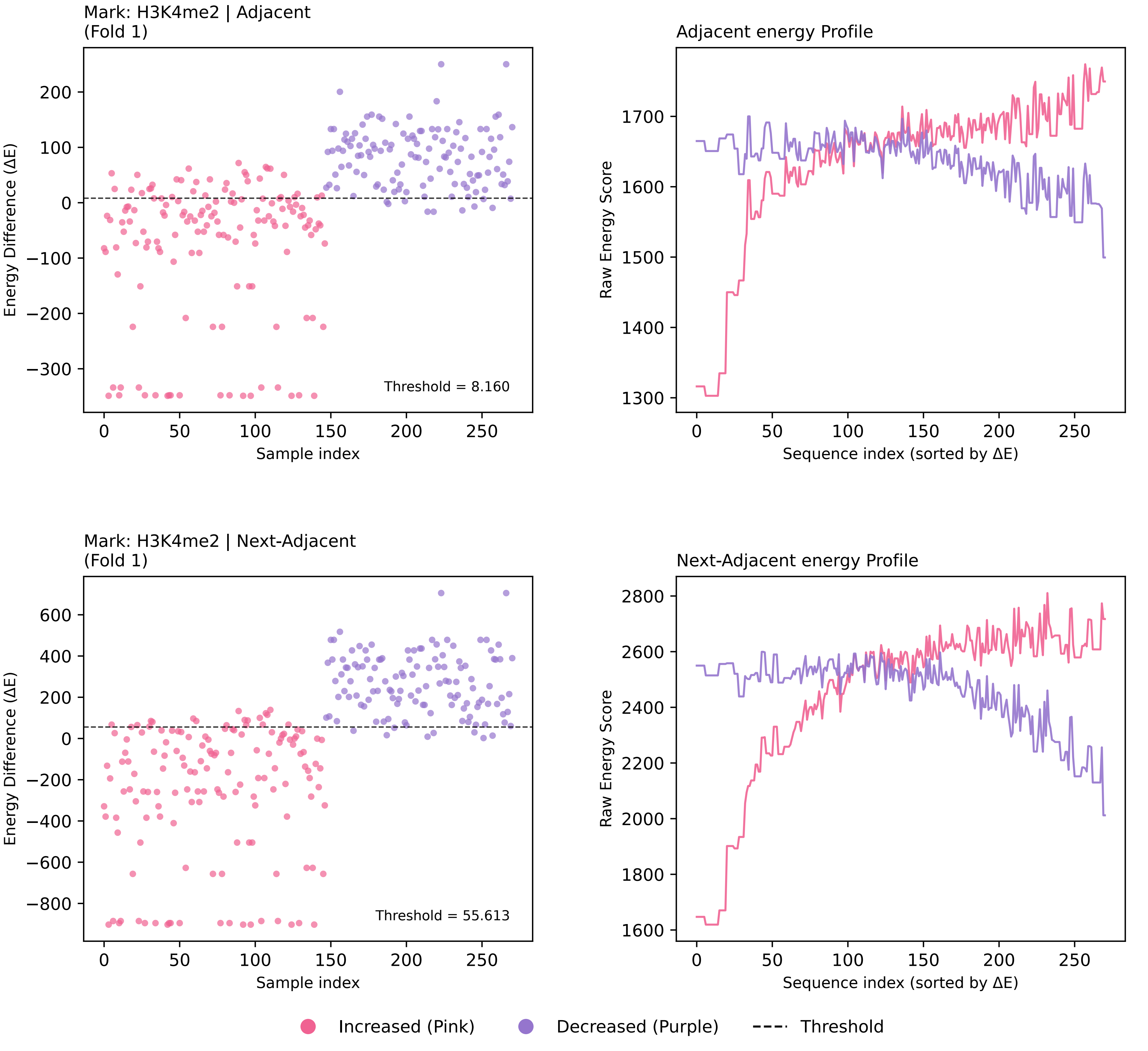

Figure S3 (Fold 1). Top: Adjacent; Bottom: Next-Adjacent.
Left panels: Scatter plots of energy differences (ΔE); Right panels: Raw energy score profile curves along the sorted sequences.

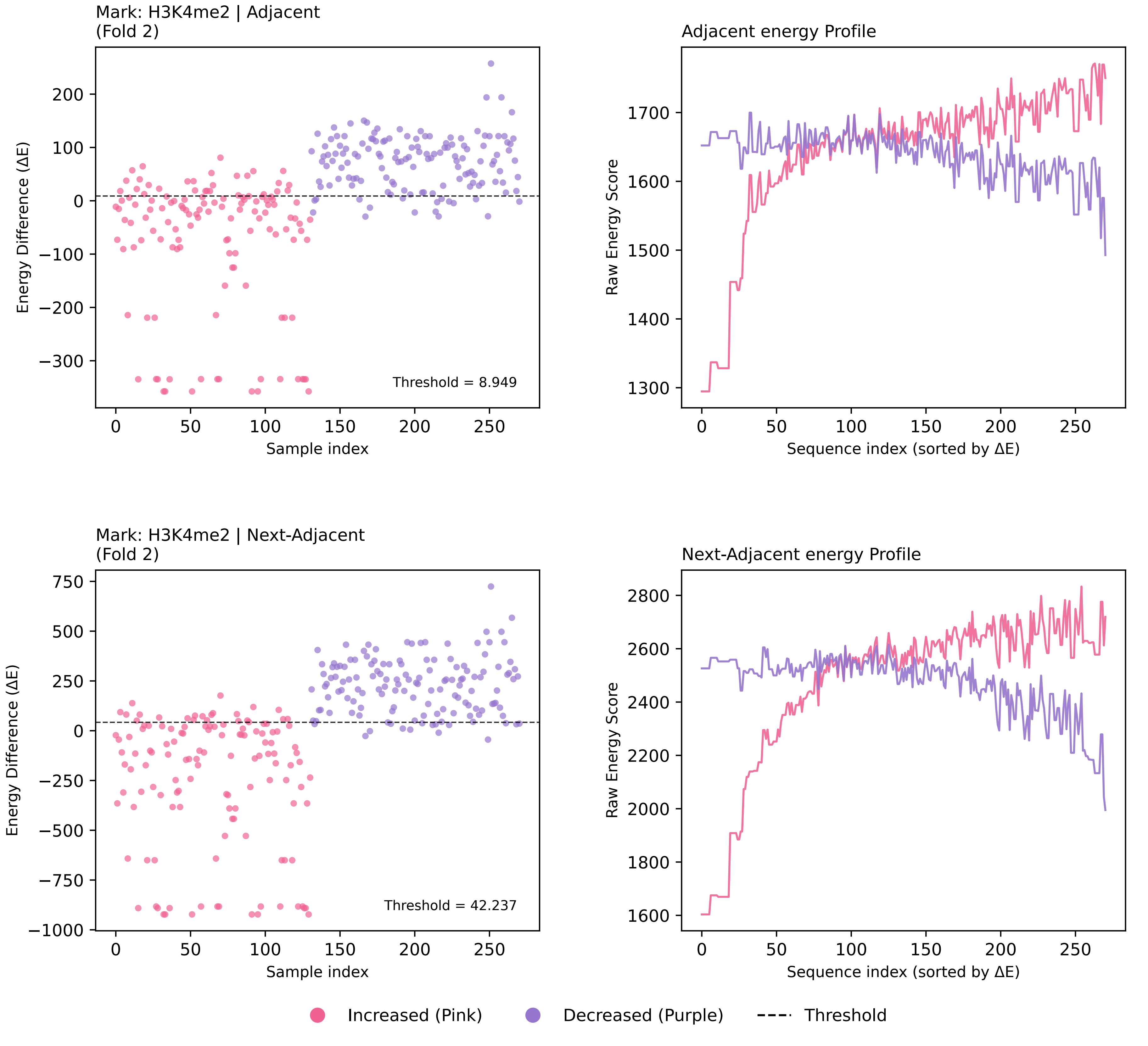

Figure S3 (Fold 2). Top: Adjacent; Bottom: Next-Adjacent.
Left panels: Scatter plots of energy differences (ΔE); Right panels: Raw energy score profile curves along the sorted sequences.

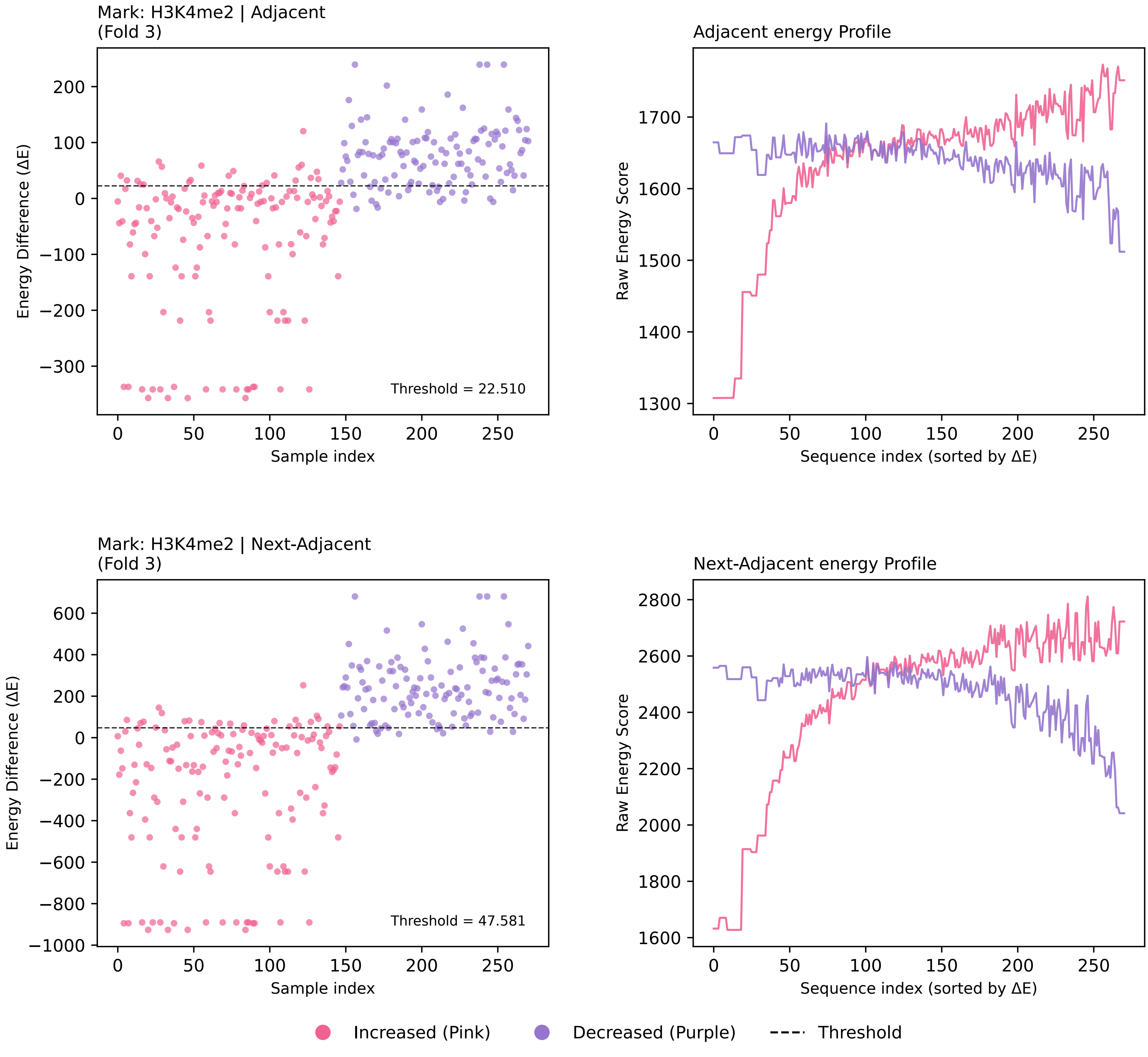

Figure S3 (Fold 3). Top: Adjacent; Bottom: Next-Adjacent.
Left panels: Scatter plots of energy differences (ΔE); Right panels: Raw energy score profile curves along the sorted sequences.

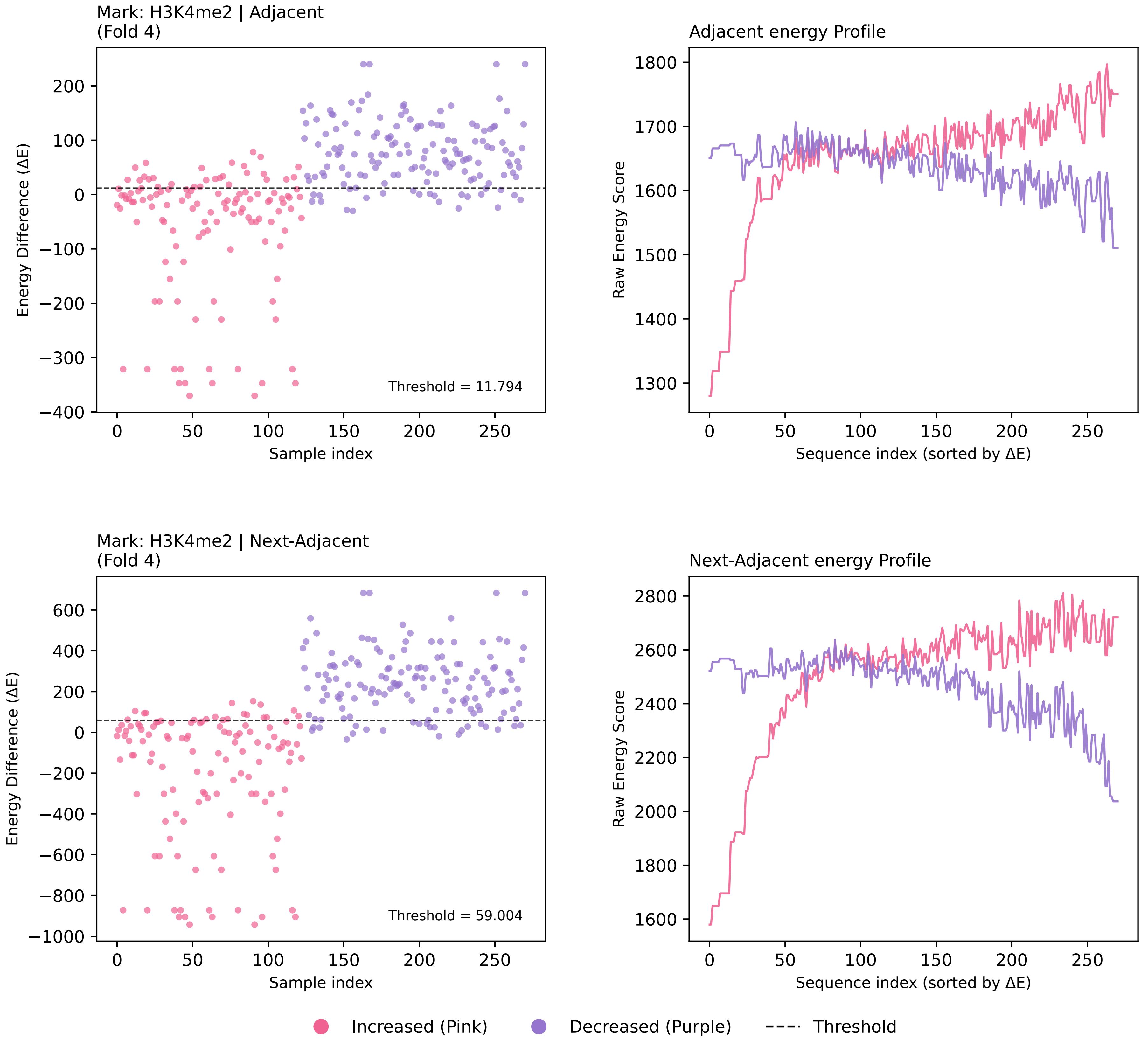

Figure S3 (Fold 4). Top: Adjacent; Bottom: Next-Adjacent.
Left panels: Scatter plots of energy differences (ΔE); Right panels: Raw energy score profile curves along the sorted sequences.

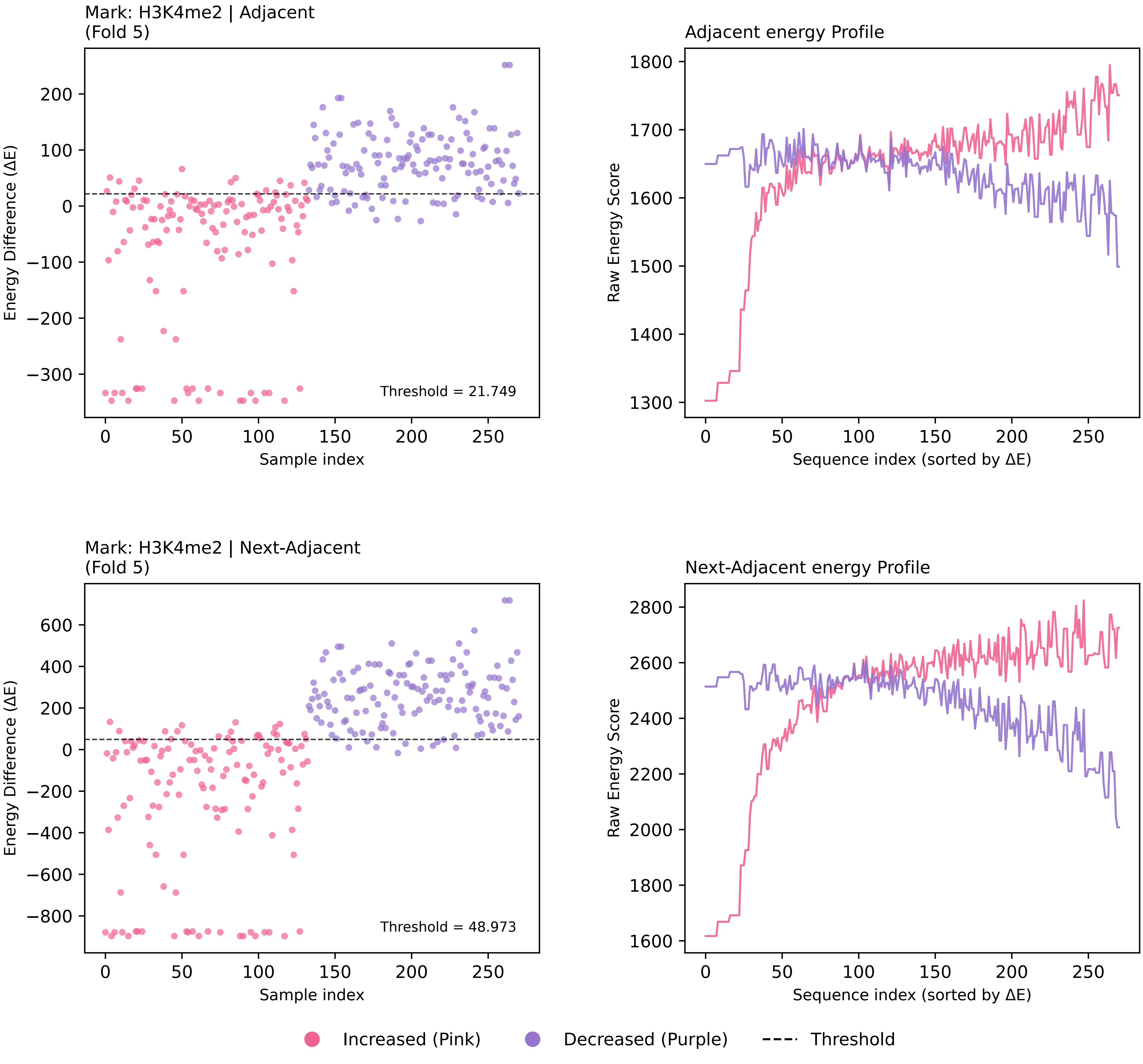

Figure S3 (Fold 5). Top: Adjacent; Bottom: Next-Adjacent.
Left panels: Scatter plots of energy differences (ΔE); Right panels: Raw energy score profile curves along the sorted sequences.

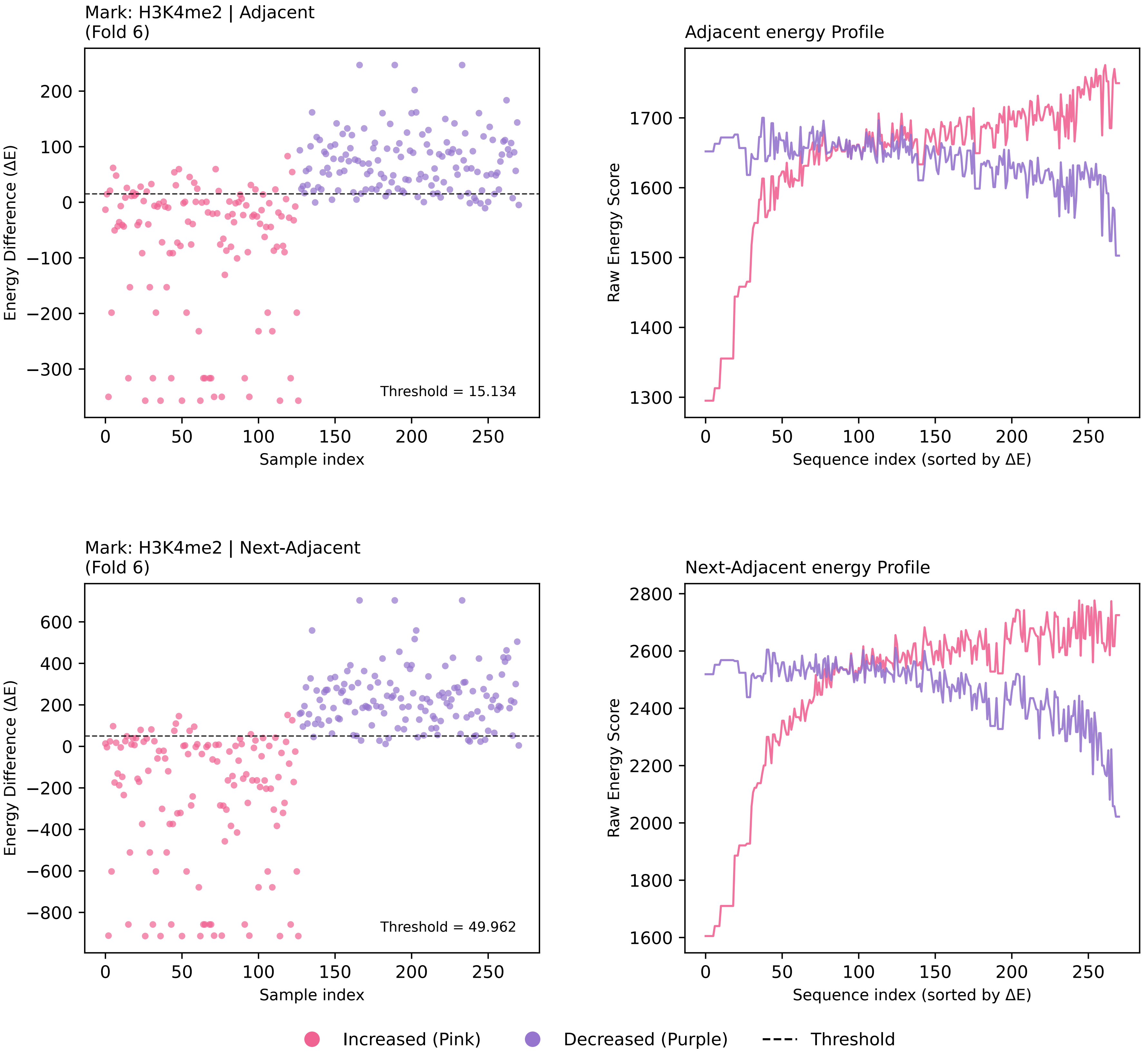

Figure S3 (Fold 6). Top: Adjacent; Bottom: Next-Adjacent.
Left panels: Scatter plots of energy differences (ΔE); Right panels: Raw energy score profile curves along the sorted sequences.

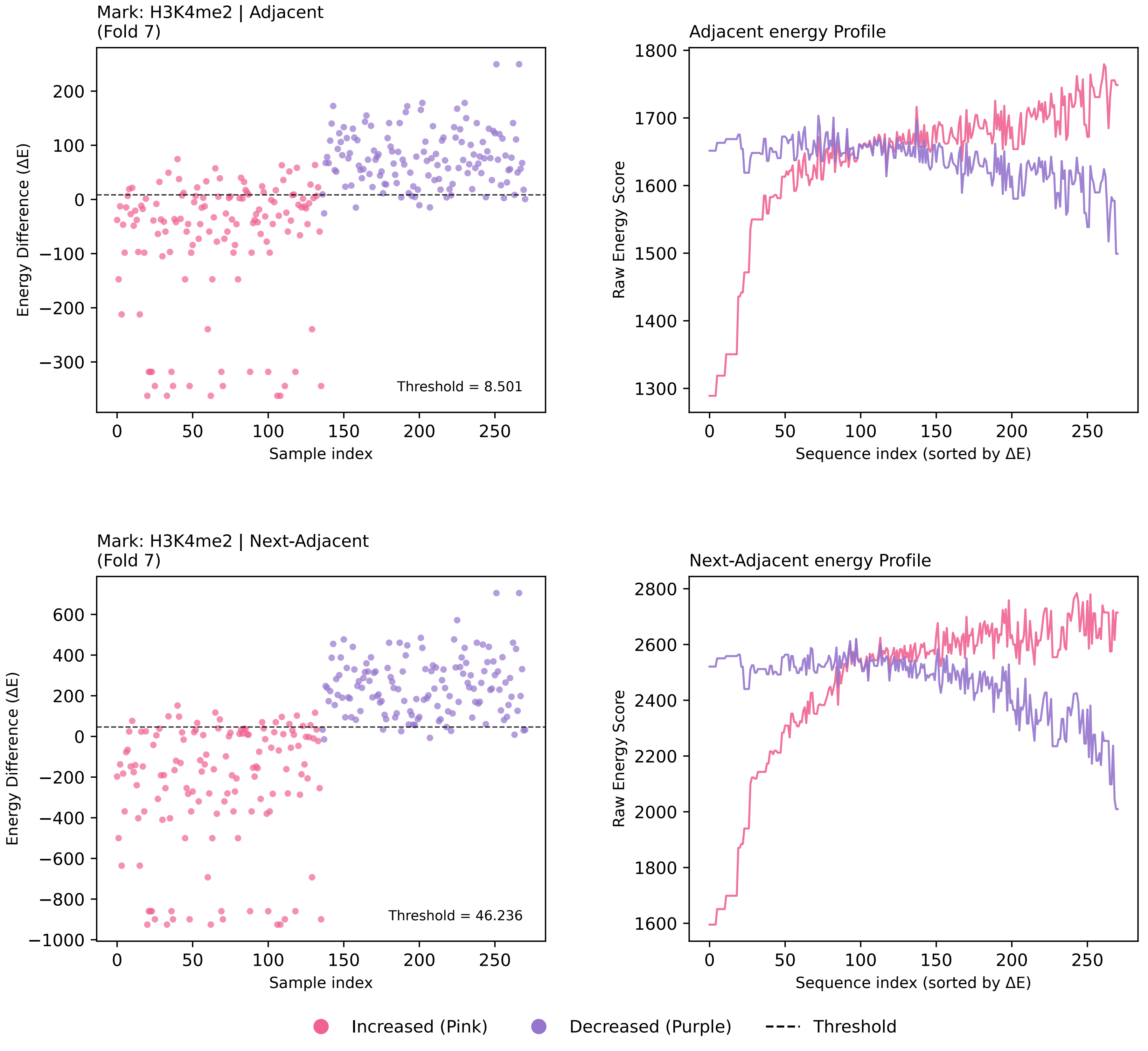

Figure S3 (Fold 7). Top: Adjacent; Bottom: Next-Adjacent.
Left panels: Scatter plots of energy differences (ΔE); Right panels: Raw energy score profile curves along the sorted sequences.

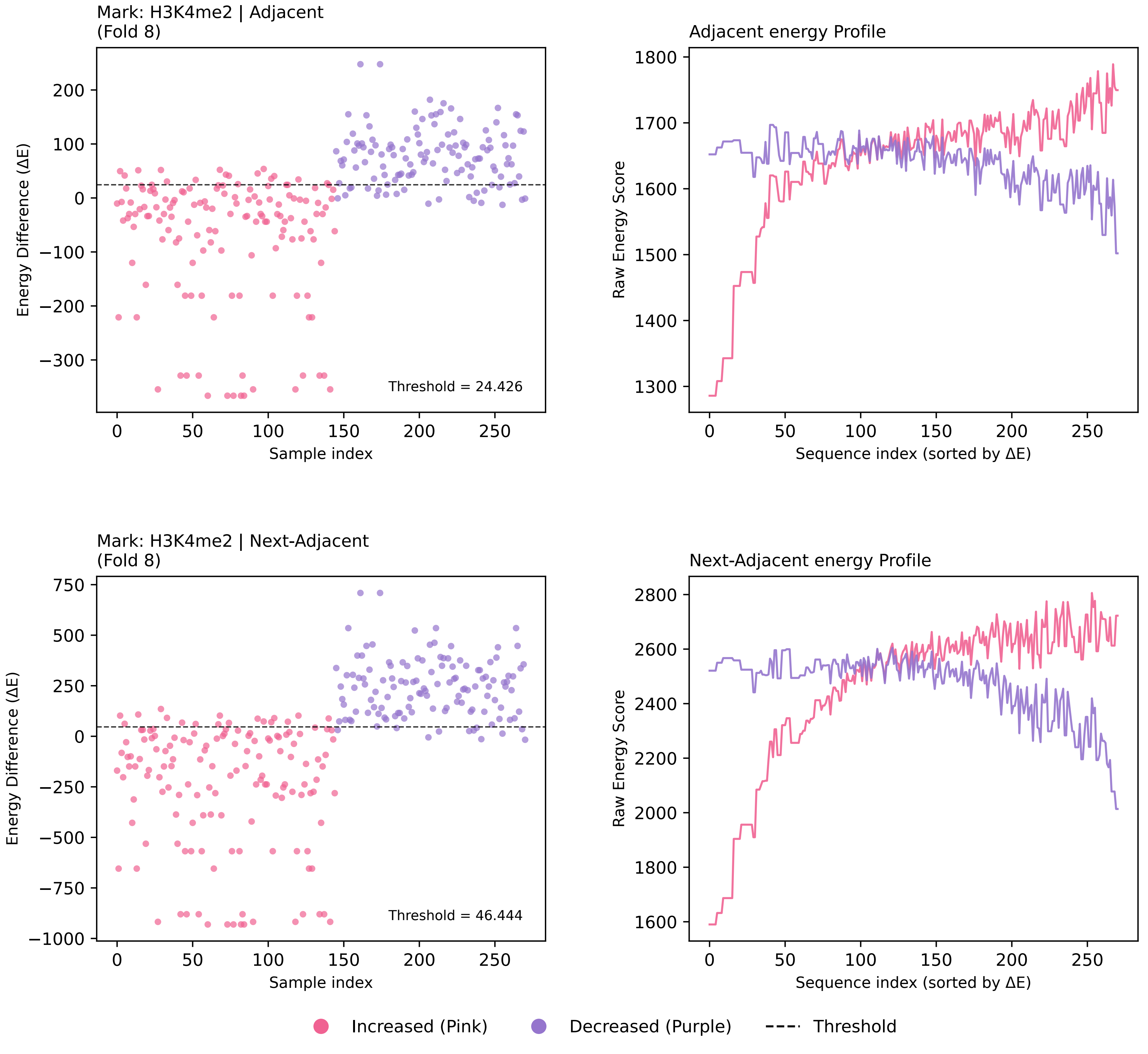

Figure S3 (Fold 8). Top: Adjacent; Bottom: Next-Adjacent.
Left panels: Scatter plots of energy differences (ΔE); Right panels: Raw energy score profile curves along the sorted sequences.

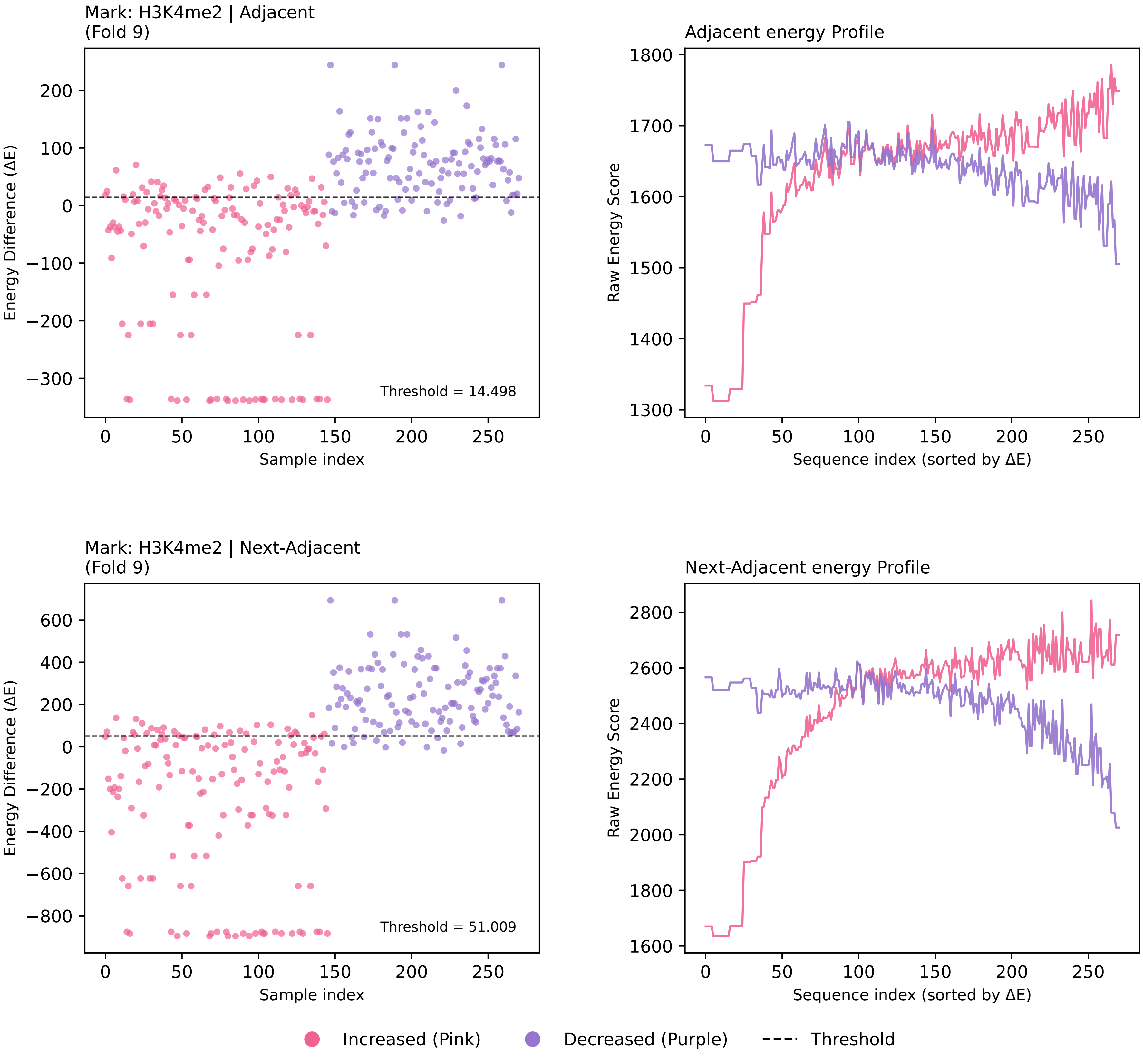

Figure S3 (Fold 9). Top: Adjacent; Bottom: Next-Adjacent.
Left panels: Scatter plots of energy differences (ΔE); Right panels: Raw energy score profile curves along the sorted sequences.

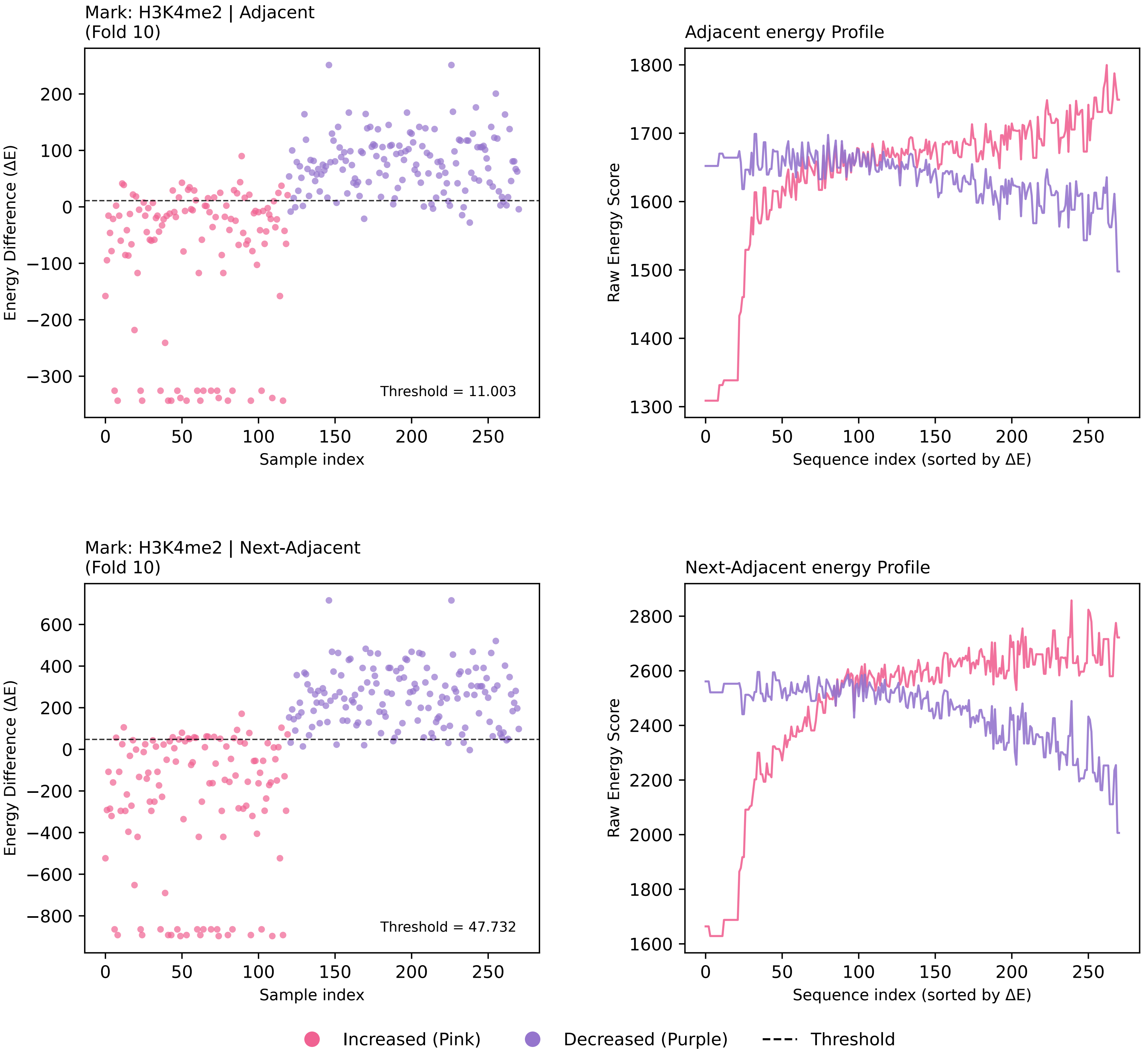

Figure S3 (Fold 10). Top: Adjacent; Bottom: Next-Adjacent.
Left panels: Scatter plots of energy differences (ΔE); Right panels: Raw energy score profile curves along the sorted sequences.
