# Supplementary material for: An Energy Model Based on Molecular Structure for Predicting Histone Modification Levels at lncRNA Promoter Regions in HepG2 Cells
Source: Int J Mol Sci. 2026 Jun 23;27(13):5653. doi: 10.3390/ijms27135653 (PMC13361589; doi:10.3390/ijms27135653)
Supplement: Supplementary file 1 [file ijms-27-05653-s001.zip › Figure_S4_H3K79me2_Report.pdf]

Performance Metrics: H3K79me2
(Folds 1 to 10)

Table S4. Supplementary table showing per-fold quantitative metrics for H3K79me2. All values are presented as mean ± confidence interval

Model	Fold	Sn (%)	Sp (%)	Ac (%)	MCC	auROC
Adjacent	1	82.857	93.706	89.286	0.771	0.953
Adjacent	2	79.021	91.429	84.266	0.709	0.944
Adjacent	3	91.429	84.615	88.929	0.762	0.963
Adjacent	4	78.146	88.636	77.815	0.668	0.923
Adjacent	5	81.061	92.715	93.561	0.746	0.944
Adjacent	6	88.889	92.568	95.185	0.816	0.968
Adjacent	7	86.986	89.781	85.616	0.767	0.958
Adjacent	8	89.333	85.714	82.667	0.752	0.969
Adjacent	9	85.526	87.023	80.263	0.724	0.948
Adjacent	10	87.302	84.076	96.032	0.71	0.953
Next-Adjacent	1	90.0	90.909	91.429	0.809	0.973
Next-Adjacent	2	86.014	92.143	88.112	0.783	0.967
Next-Adjacent	3	93.571	89.51	92.5	0.831	0.98
Next-Adjacent	4	84.768	92.424	82.781	0.77	0.958
Next-Adjacent	5	87.879	94.702	98.106	0.83	0.975
Next-Adjacent	6	89.63	93.243	95.926	0.83	0.987
Next-Adjacent	7	92.466	91.971	89.384	0.844	0.984
Next-Adjacent	8	93.333	94.737	88.667	0.88	0.986
Next-Adjacent	9	86.842	91.603	82.895	0.782	0.971
Next-Adjacent	10	84.921	93.631	100.794	0.793	0.972

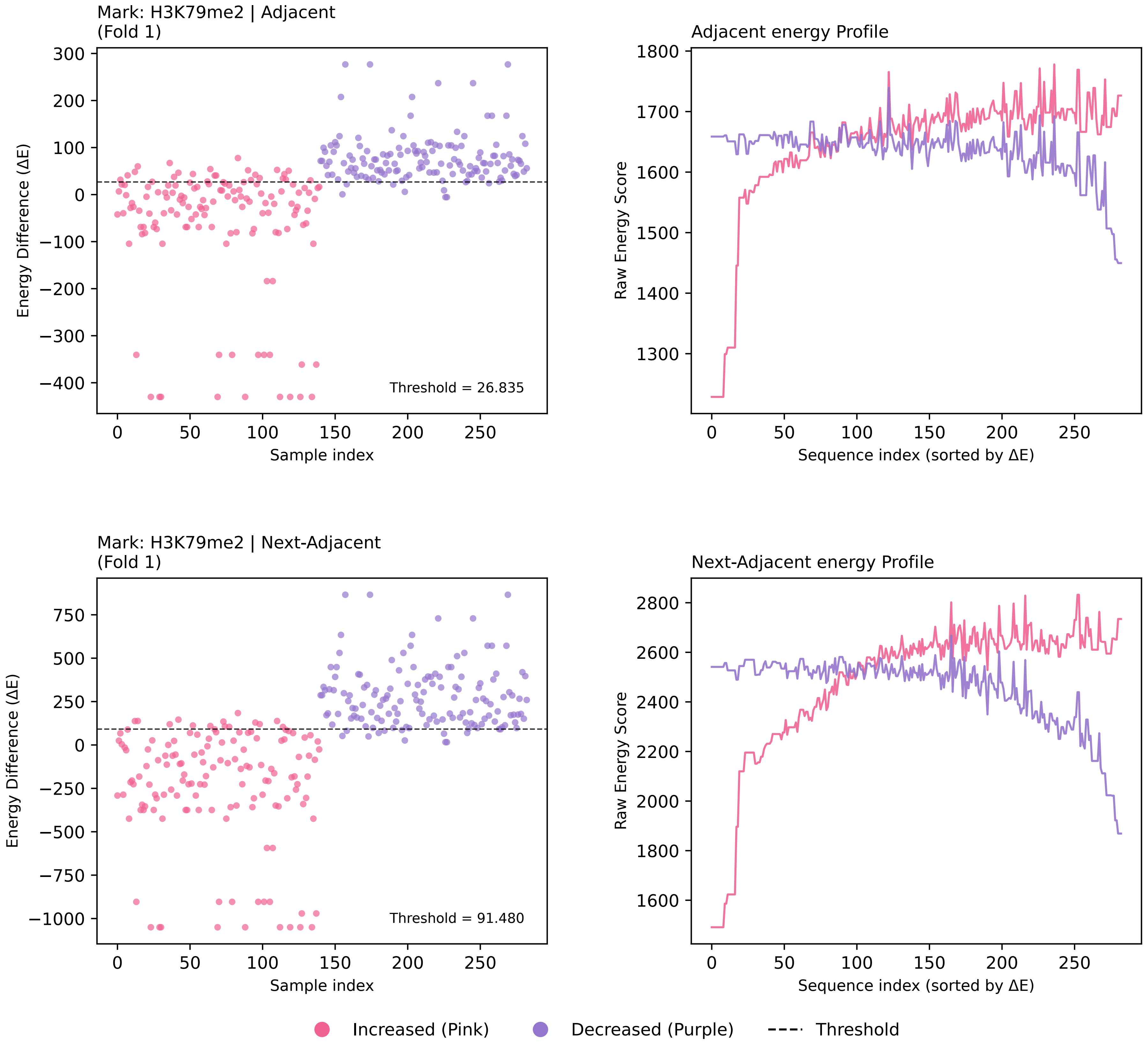

Figure S4 (Fold 1). Top: Adjacent; Bottom: Next-Adjacent.
Left panels: Scatter plots of energy differences (ΔE); Right panels: Raw energy score profile curves along the sorted sequences.

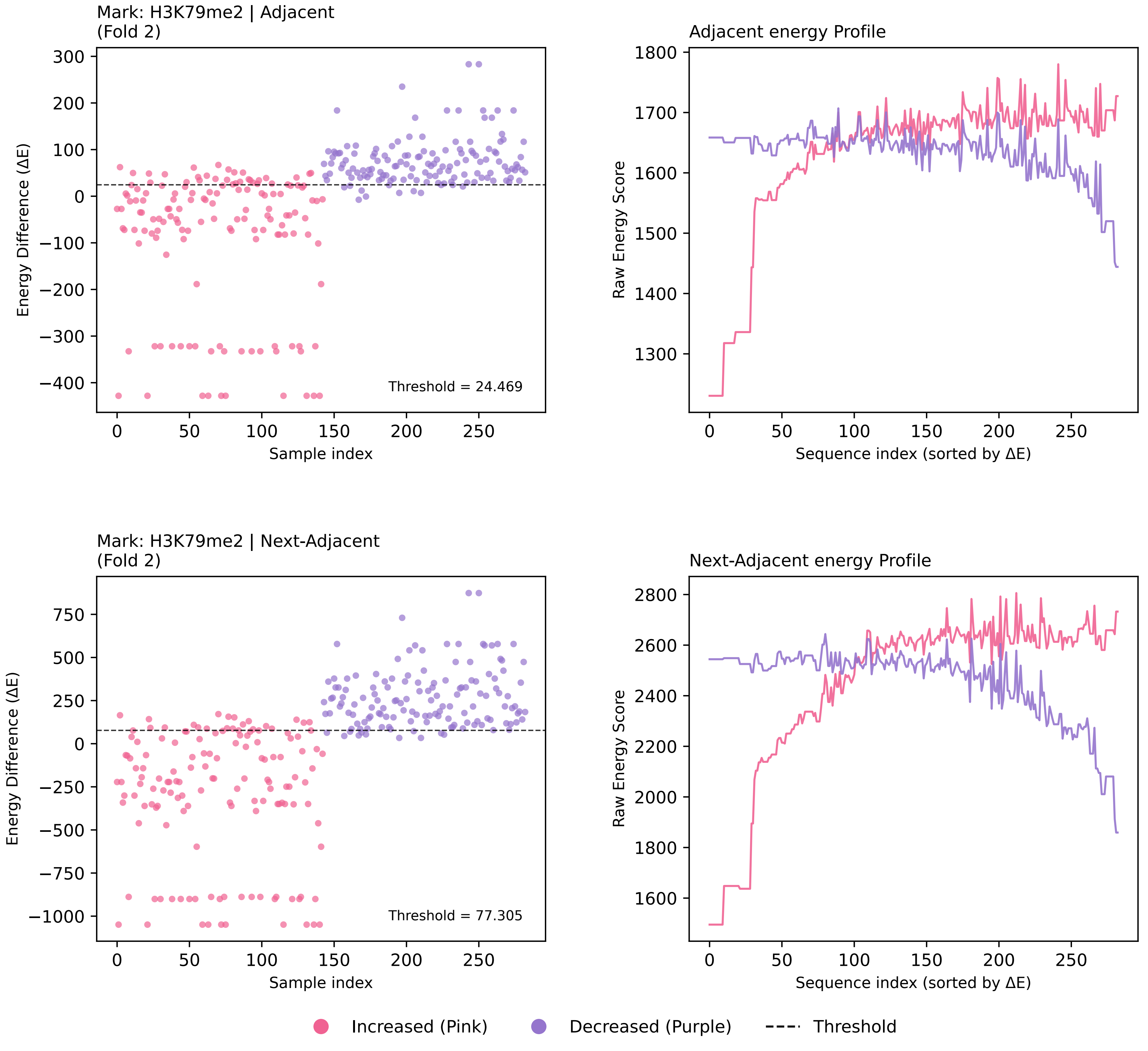

Figure S4 (Fold 2). Top: Adjacent; Bottom: Next-Adjacent.
Left panels: Scatter plots of energy differences (ΔE); Right panels: Raw energy score profile curves along the sorted sequences.

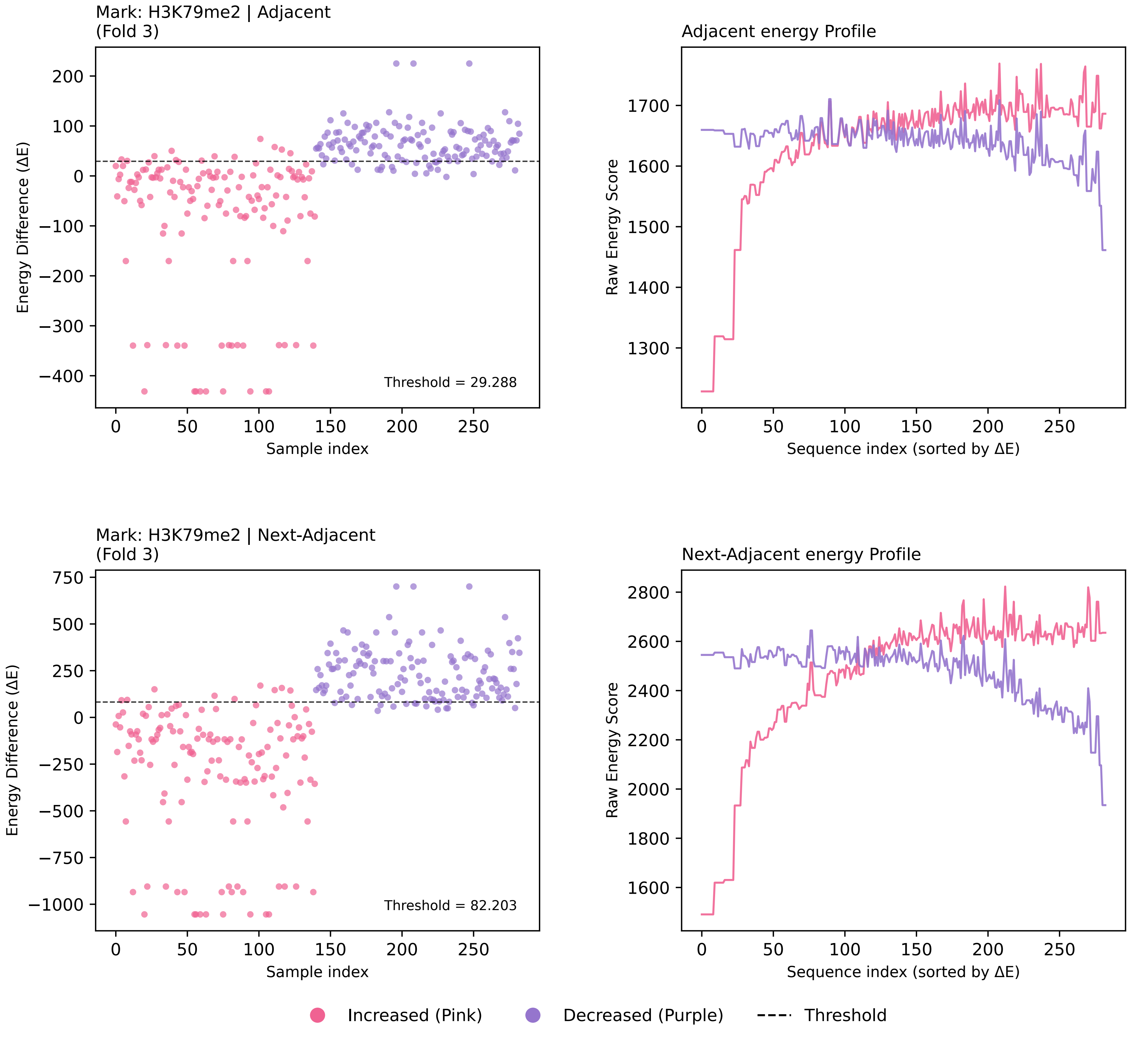

Figure S4 (Fold 3). Top: Adjacent; Bottom: Next-Adjacent.
Left panels: Scatter plots of energy differences (ΔE); Right panels: Raw energy score profile curves along the sorted sequences.

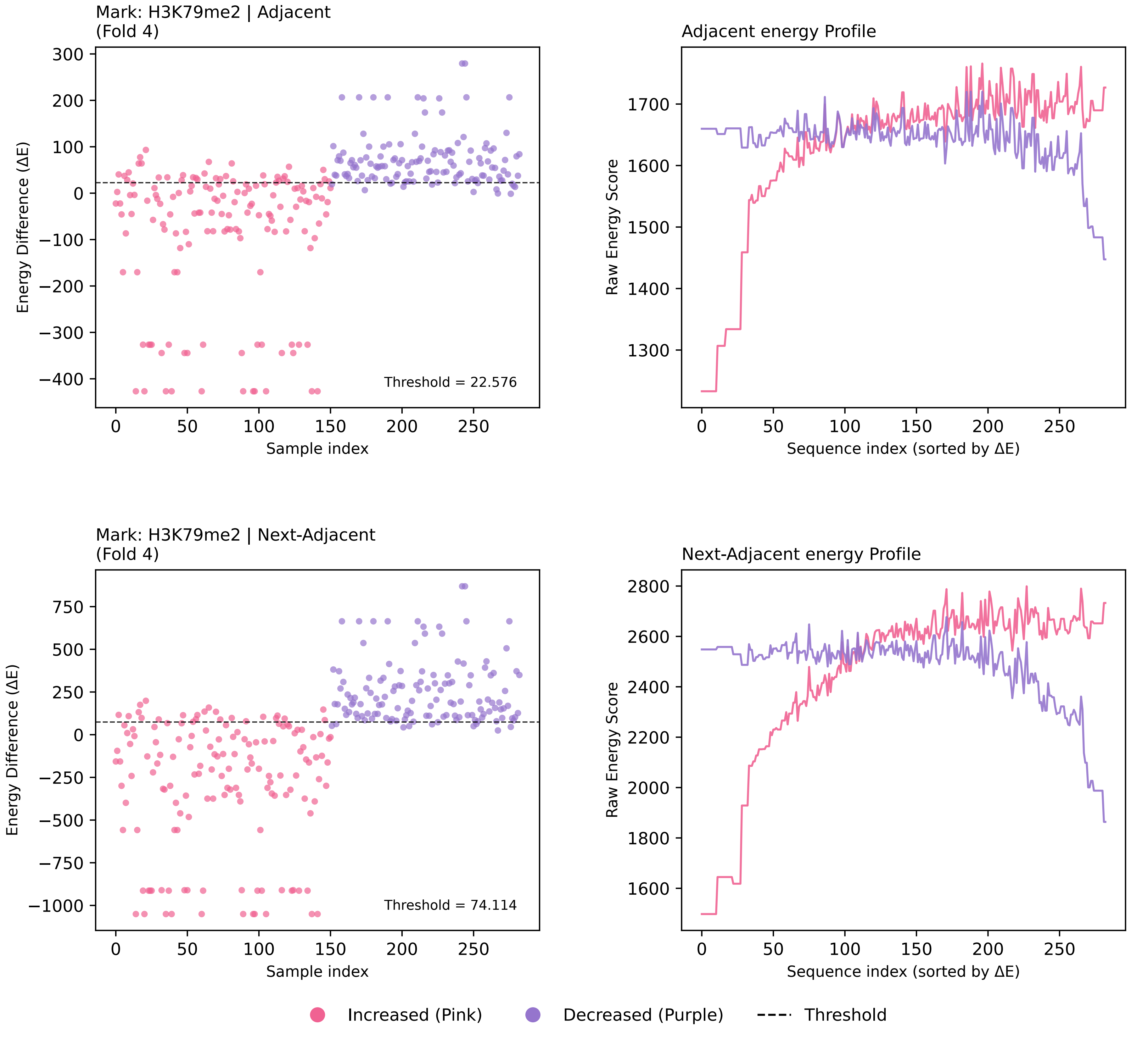

Figure S4 (Fold 4). Top: Adjacent; Bottom: Next-Adjacent.
Left panels: Scatter plots of energy differences (ΔE); Right panels: Raw energy score profile curves along the sorted sequences.

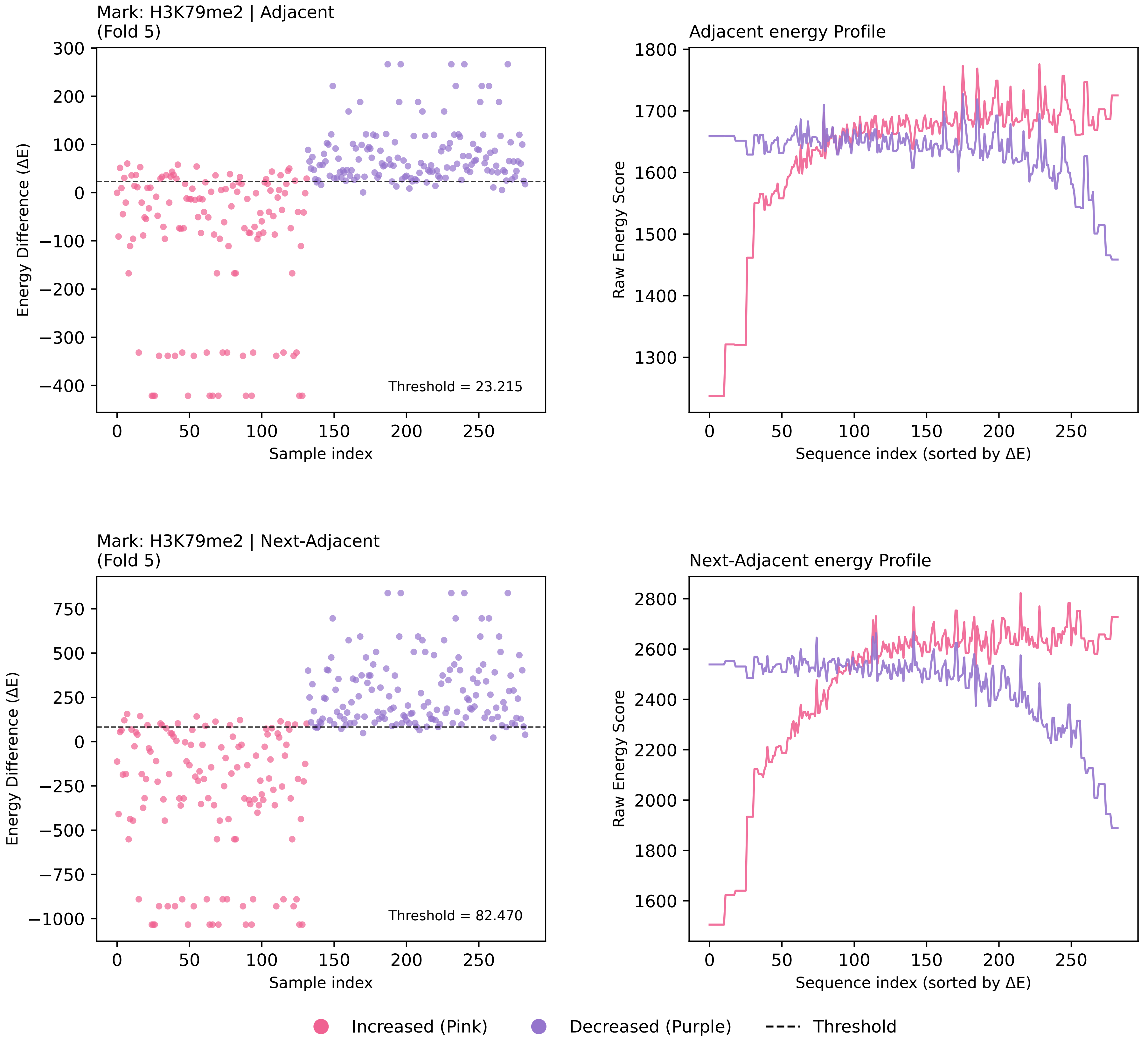

Figure S4 (Fold 5). Top: Adjacent; Bottom: Next-Adjacent.
Left panels: Scatter plots of energy differences (ΔE); Right panels: Raw energy score profile curves along the sorted sequences.

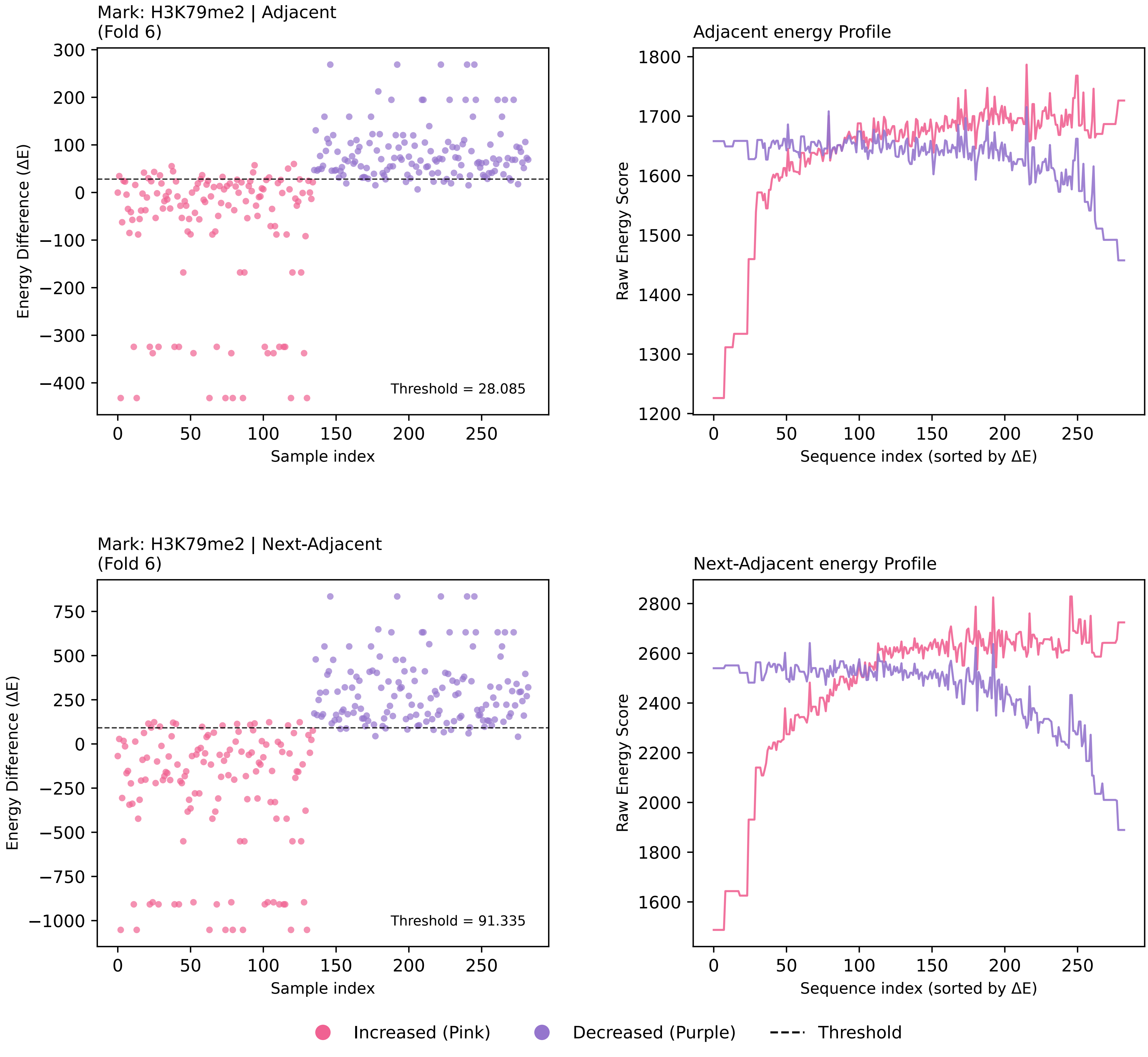

Figure S4 (Fold 6). Top: Adjacent; Bottom: Next-Adjacent.
Left panels: Scatter plots of energy differences (ΔE); Right panels: Raw energy score profile curves along the sorted sequences.

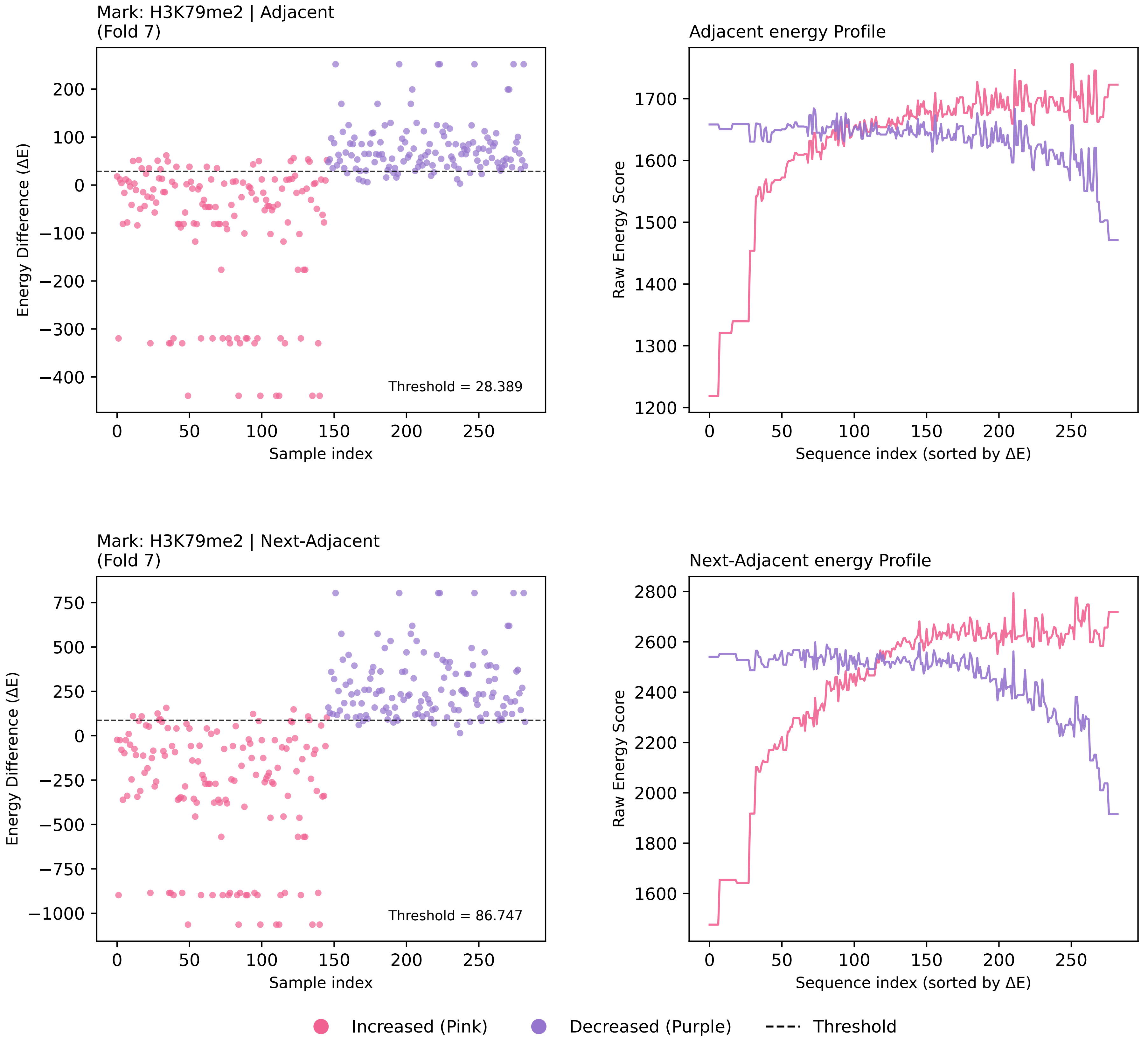

Figure S4 (Fold 7). Top: Adjacent; Bottom: Next-Adjacent.
Left panels: Scatter plots of energy differences (ΔE); Right panels: Raw energy score profile curves along the sorted sequences.

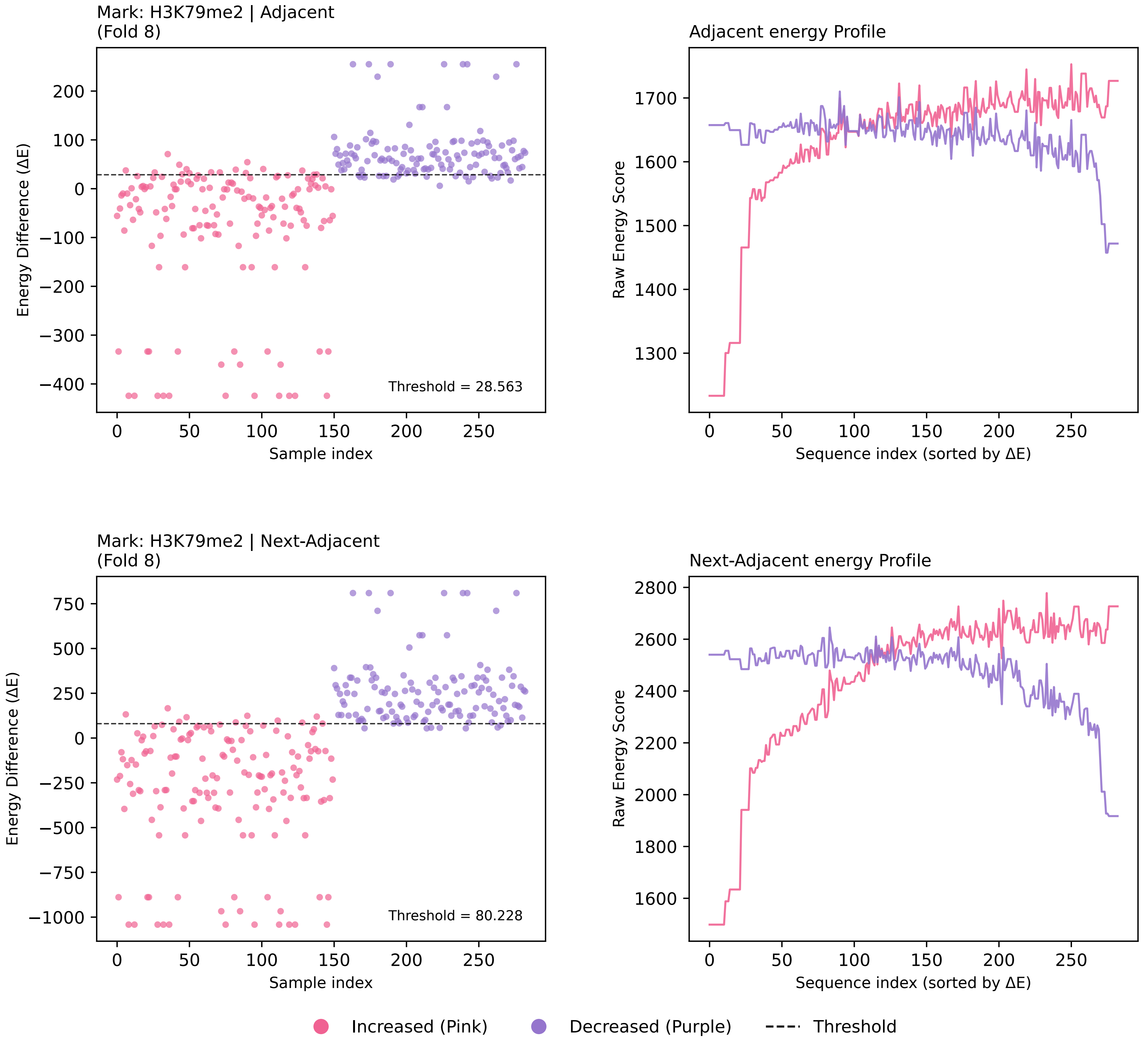

Figure S4 (Fold 8). Top: Adjacent; Bottom: Next-Adjacent.
Left panels: Scatter plots of energy differences (ΔE); Right panels: Raw energy score profile curves along the sorted sequences.

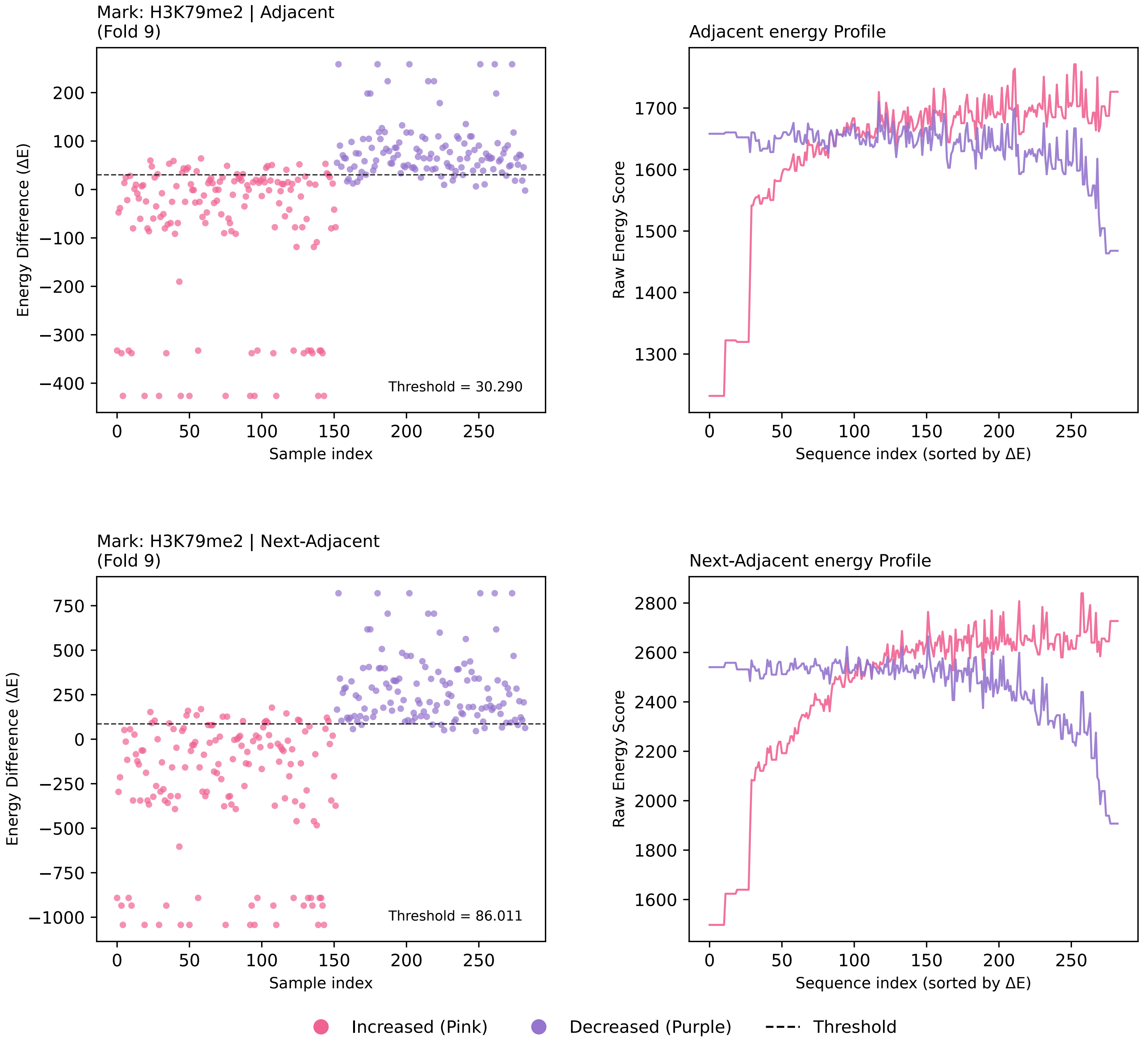

Figure S4 (Fold 9). Top: Adjacent; Bottom: Next-Adjacent.
Left panels: Scatter plots of energy differences (ΔE); Right panels: Raw energy score profile curves along the sorted sequences.

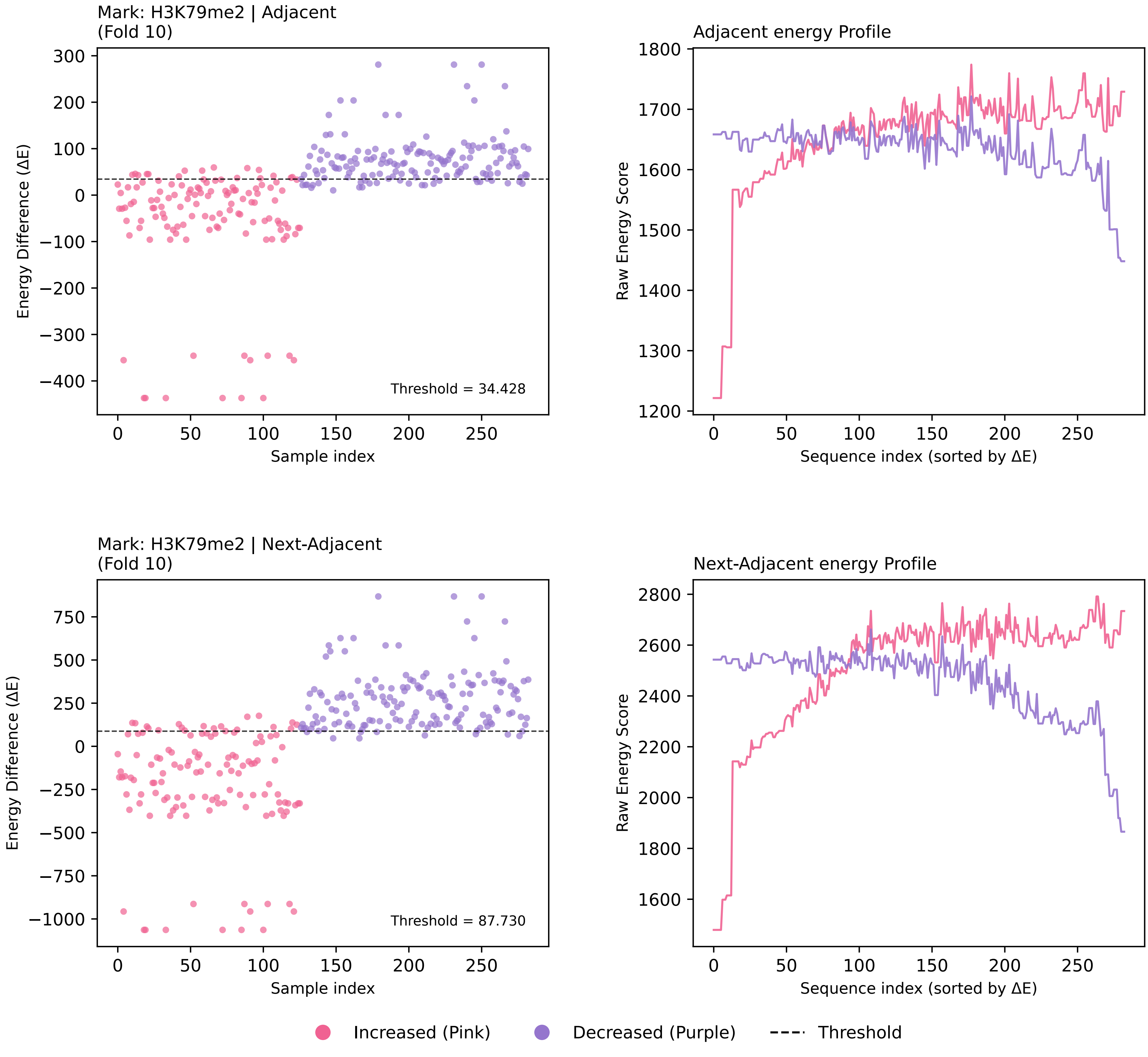

Figure S4 (Fold 10). Top: Adjacent; Bottom: Next-Adjacent.
Left panels: Scatter plots of energy differences (ΔE); Right panels: Raw energy score profile curves along the sorted sequences.
