# Supplementary material for: An Energy Model Based on Molecular Structure for Predicting Histone Modification Levels at lncRNA Promoter Regions in HepG2 Cells
Source: Int J Mol Sci. 2026 Jun 23;27(13):5653. doi: 10.3390/ijms27135653 (PMC13361589; doi:10.3390/ijms27135653)
Supplement: Supplementary file 1 [file ijms-27-05653-s001.zip › Figure_S5_H3K9ac_Report.pdf]

Performance Metrics: H3K9ac
(Folds 1 to 10)

Table S5. Supplementary table showing per-fold quantitative metrics for H3K9ac. All values are presented as mean ± confidence interval

Model	Fold	Sn (%)	Sp (%)	Ac (%)	MCC	auROC
Adjacent	1	82.54	78.481	74.074	0.611	0.902
Adjacent	2	65.517	90.173	77.586	0.574	0.884
Adjacent	3	77.957	90.683	78.226	0.687	0.938
Adjacent	4	85.455	87.363	90.909	0.728	0.949
Adjacent	5	72.093	90.857	82.267	0.642	0.915
Adjacent	6	75.758	85.714	85.152	0.619	0.899
Adjacent	7	72.152	90.426	89.873	0.642	0.918
Adjacent	8	82.584	89.881	83.708	0.725	0.941
Adjacent	9	79.545	87.059	81.818	0.667	0.929
Adjacent	10	83.529	81.25	83.824	0.648	0.909
Next-Adjacent	1	86.772	87.975	80.159	0.746	0.962
Next-Adjacent	2	81.609	88.439	84.77	0.702	0.957
Next-Adjacent	3	85.484	94.41	83.602	0.797	0.977
Next-Adjacent	4	92.727	90.11	96.061	0.827	0.981
Next-Adjacent	5	83.14	91.429	88.081	0.749	0.965
Next-Adjacent	6	88.485	86.813	92.121	0.752	0.961
Next-Adjacent	7	81.013	92.021	95.253	0.739	0.967
Next-Adjacent	8	88.202	95.833	89.326	0.841	0.982
Next-Adjacent	9	85.227	95.294	88.636	0.808	0.973
Next-Adjacent	10	86.471	89.773	89.706	0.763	0.96

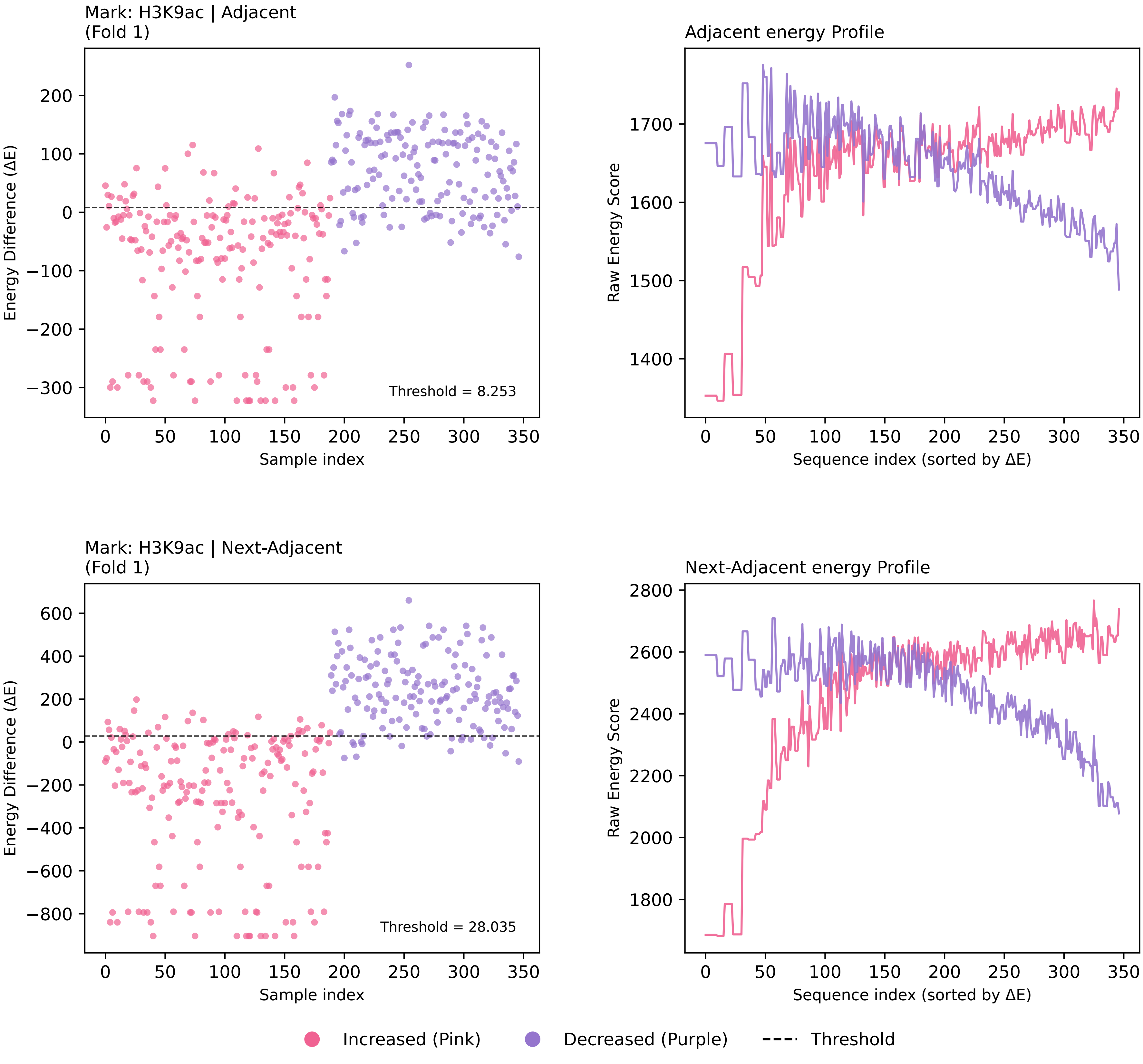

Figure S5 (Fold 1). Top: Adjacent; Bottom: Next-Adjacent.
Left panels: Scatter plots of energy differences (ΔE); Right panels: Raw energy score profile curves along the sorted sequences.

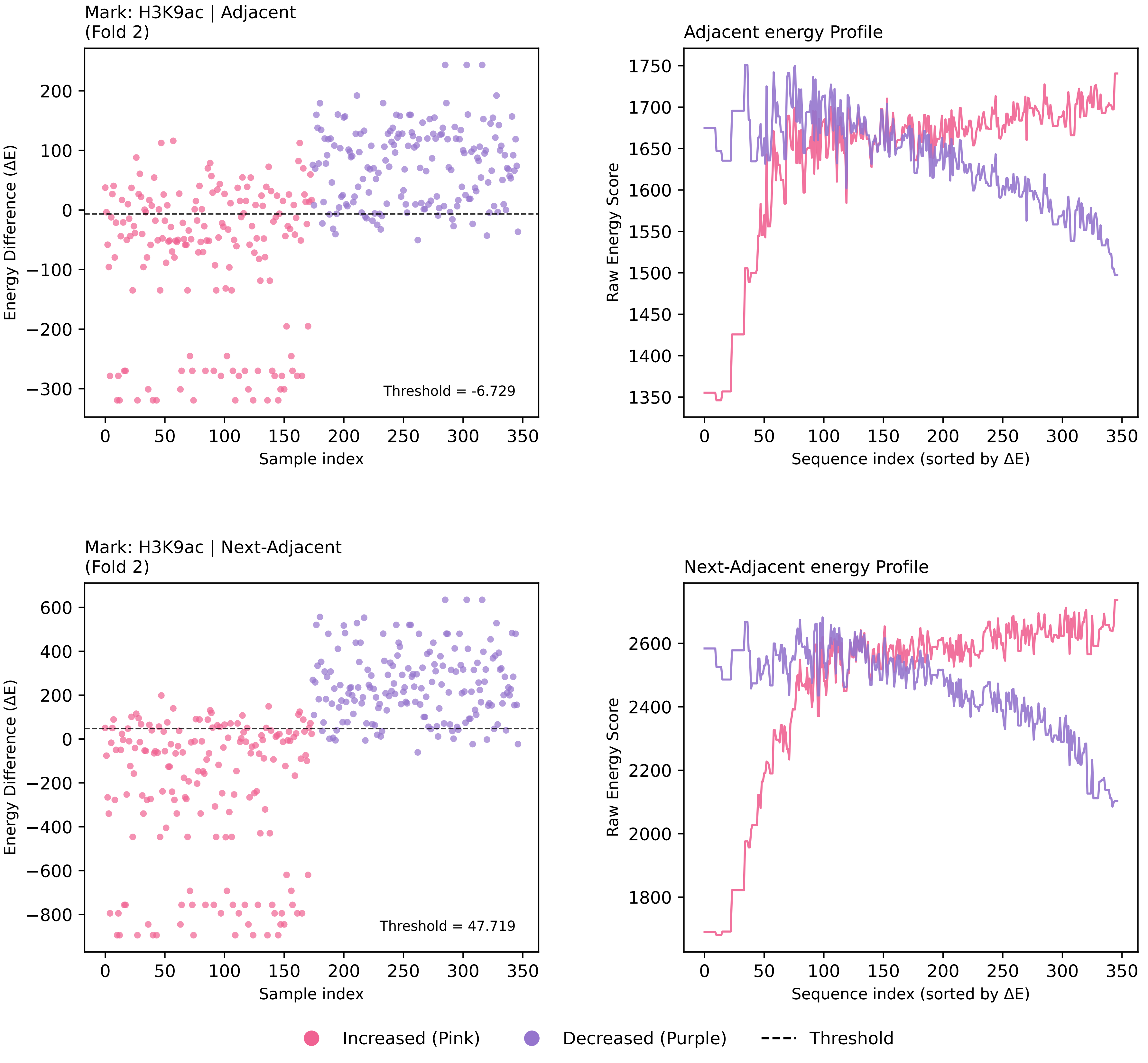

Figure S5 (Fold 2). Top: Adjacent; Bottom: Next-Adjacent.
Left panels: Scatter plots of energy differences (ΔE); Right panels: Raw energy score profile curves along the sorted sequences.

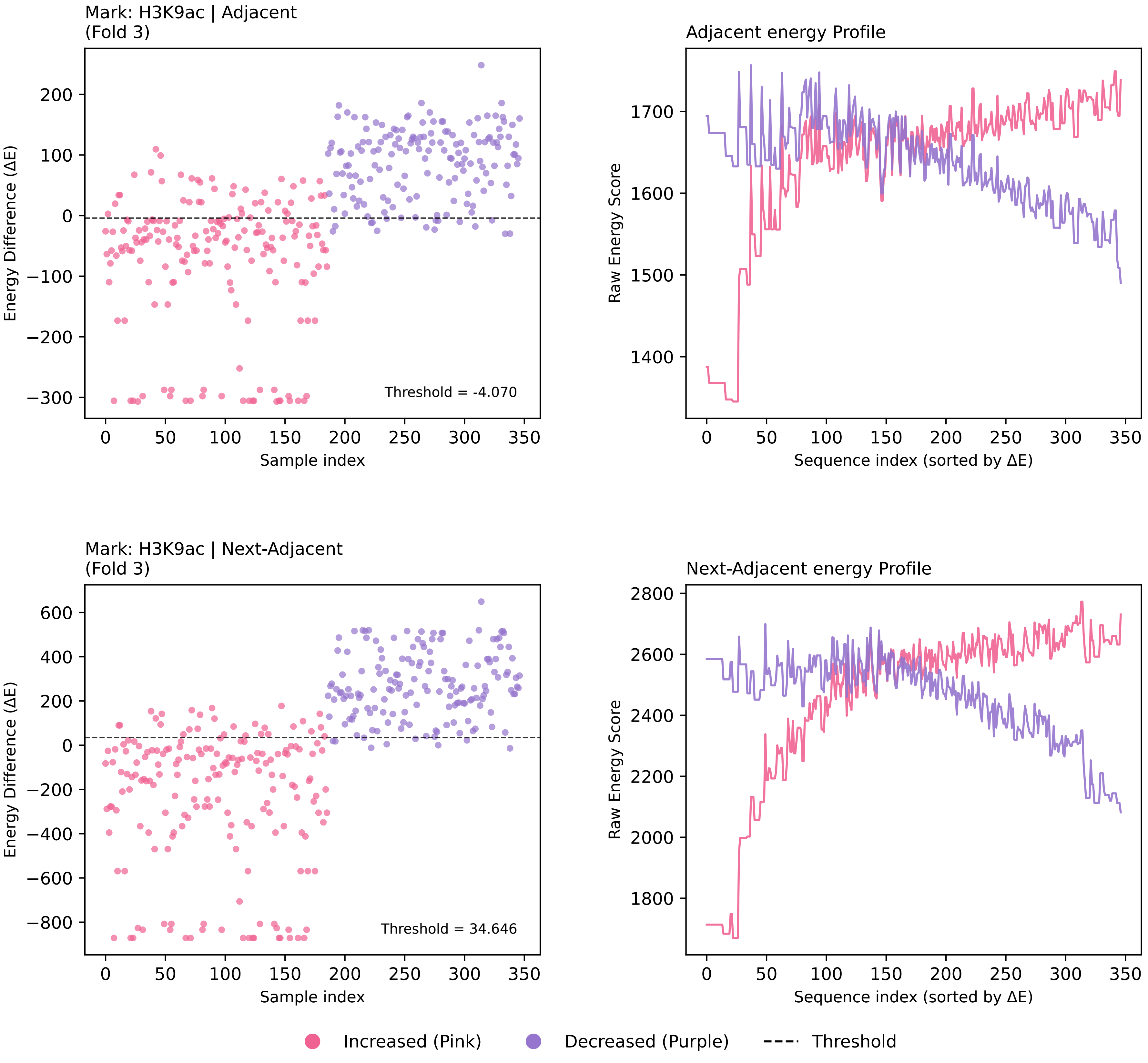

Figure S5 (Fold 3). Top: Adjacent; Bottom: Next-Adjacent.
Left panels: Scatter plots of energy differences (ΔE); Right panels: Raw energy score profile curves along the sorted sequences.

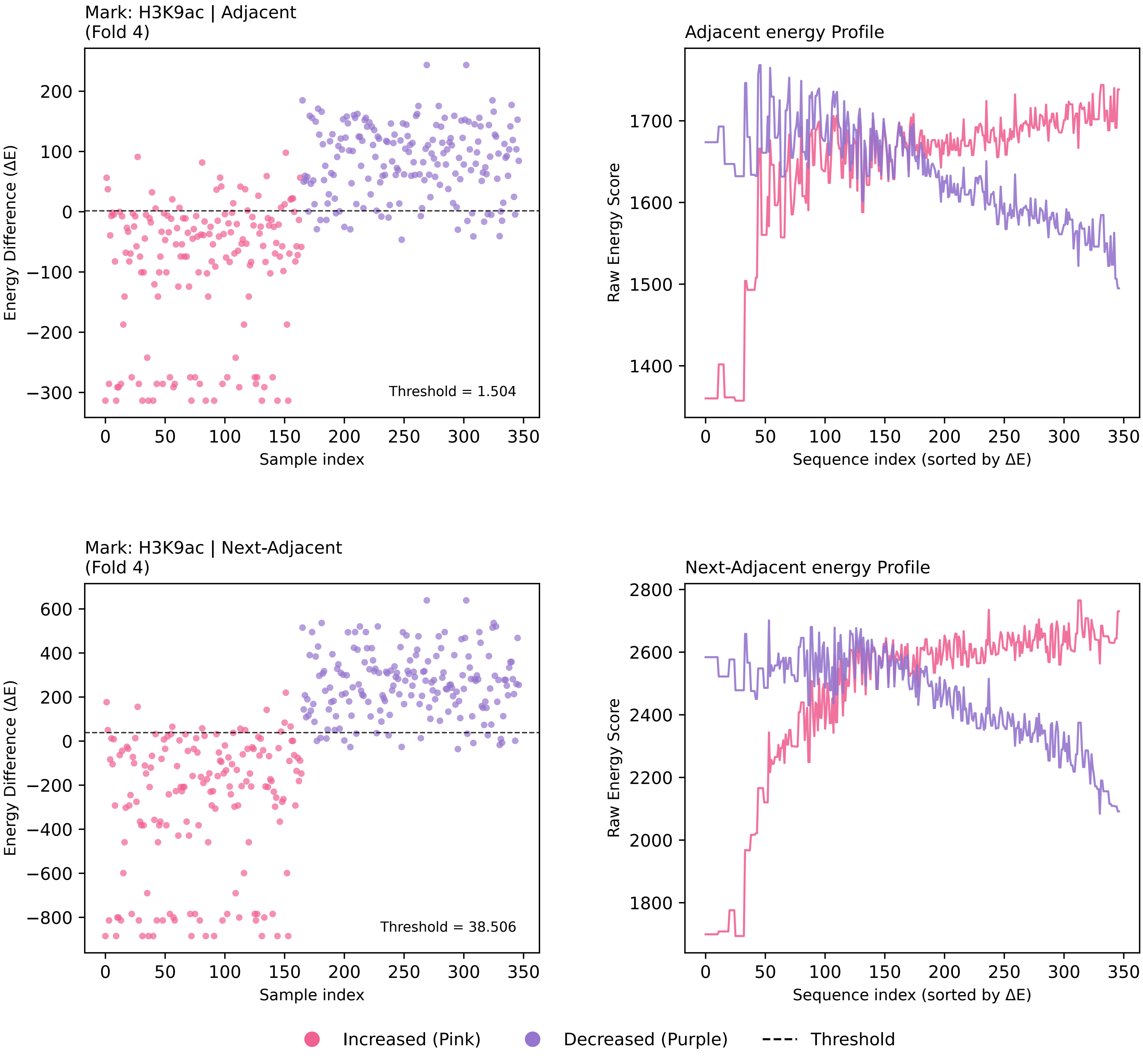

Figure S5 (Fold 4). Top: Adjacent; Bottom: Next-Adjacent.
Left panels: Scatter plots of energy differences (ΔE); Right panels: Raw energy score profile curves along the sorted sequences.

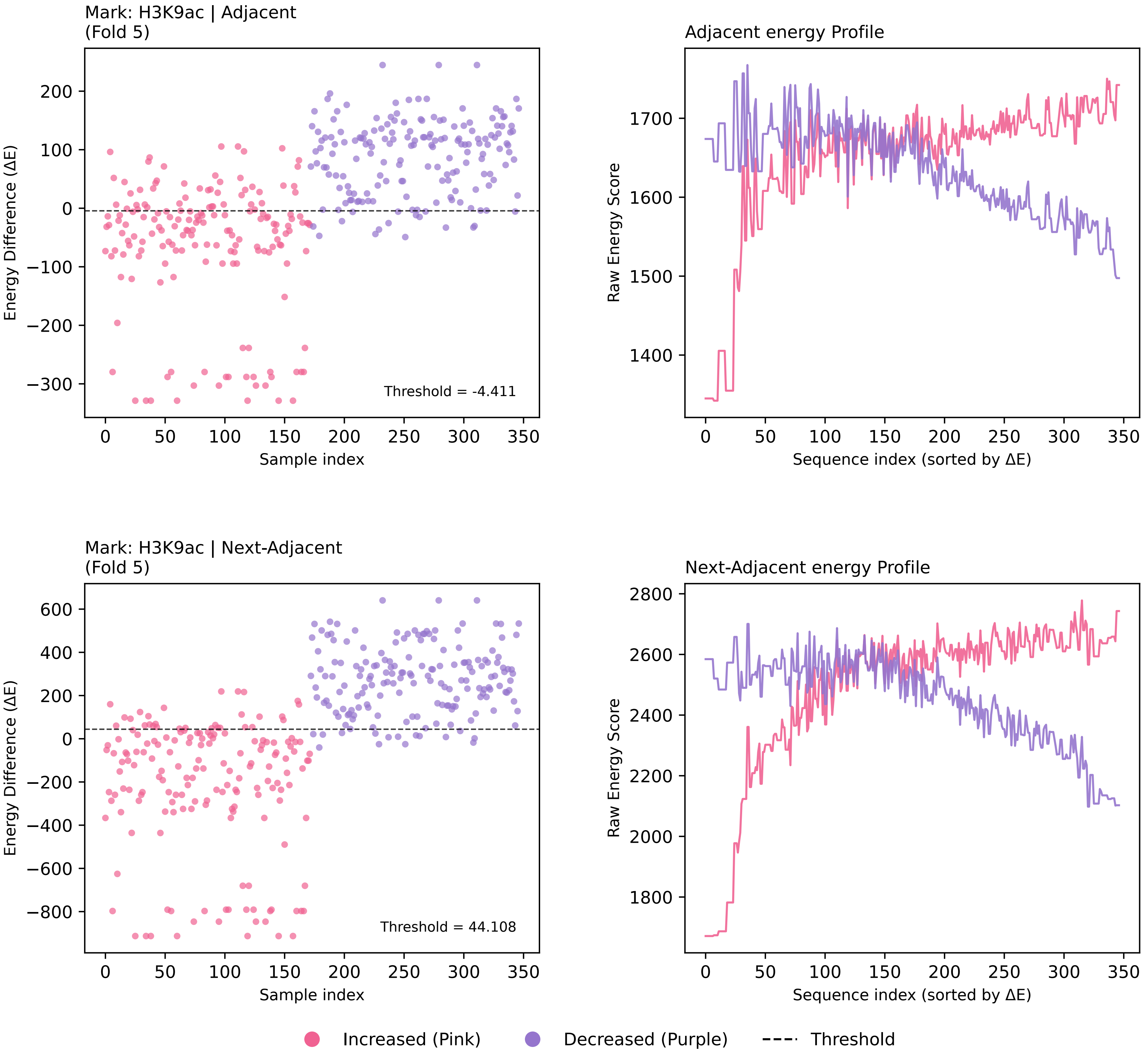

Figure S5 (Fold 5). Top: Adjacent; Bottom: Next-Adjacent.
Left panels: Scatter plots of energy differences (ΔE); Right panels: Raw energy score profile curves along the sorted sequences.

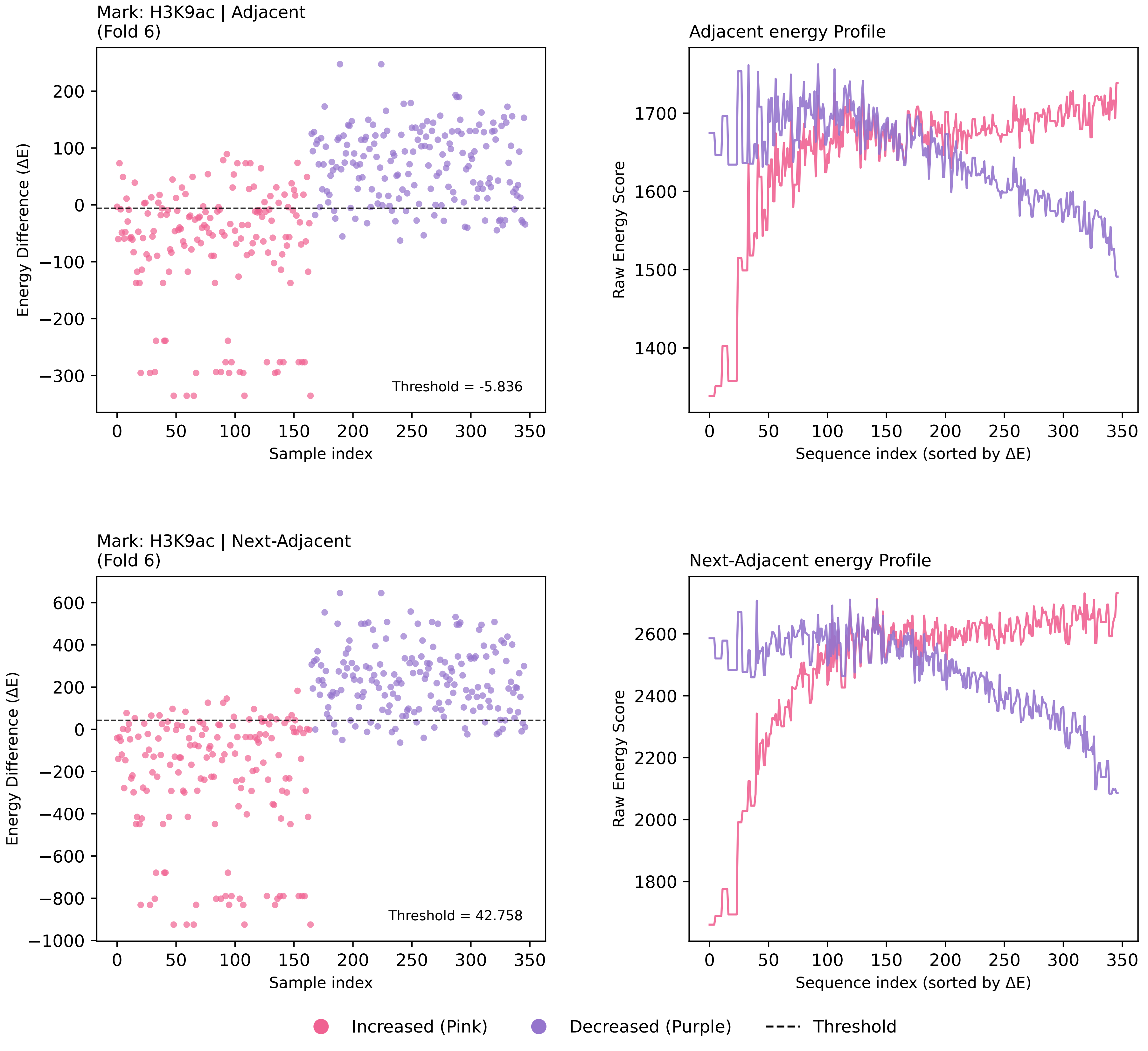

Figure S5 (Fold 6). Top: Adjacent; Bottom: Next-Adjacent.
Left panels: Scatter plots of energy differences (ΔE); Right panels: Raw energy score profile curves along the sorted sequences.

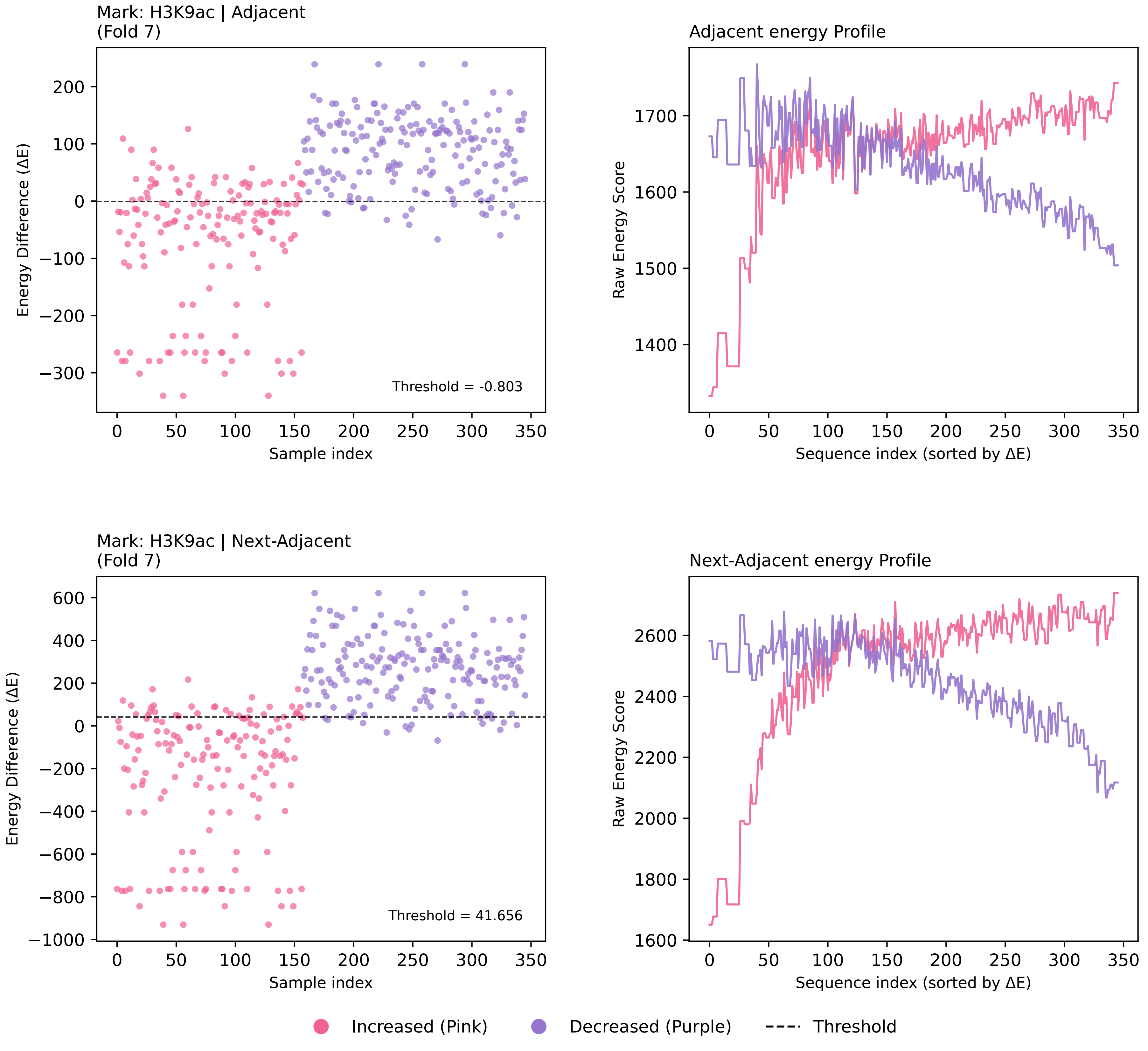

Figure S5 (Fold 7). Top: Adjacent; Bottom: Next-Adjacent.
Left panels: Scatter plots of energy differences (ΔE); Right panels: Raw energy score profile curves along the sorted sequences.

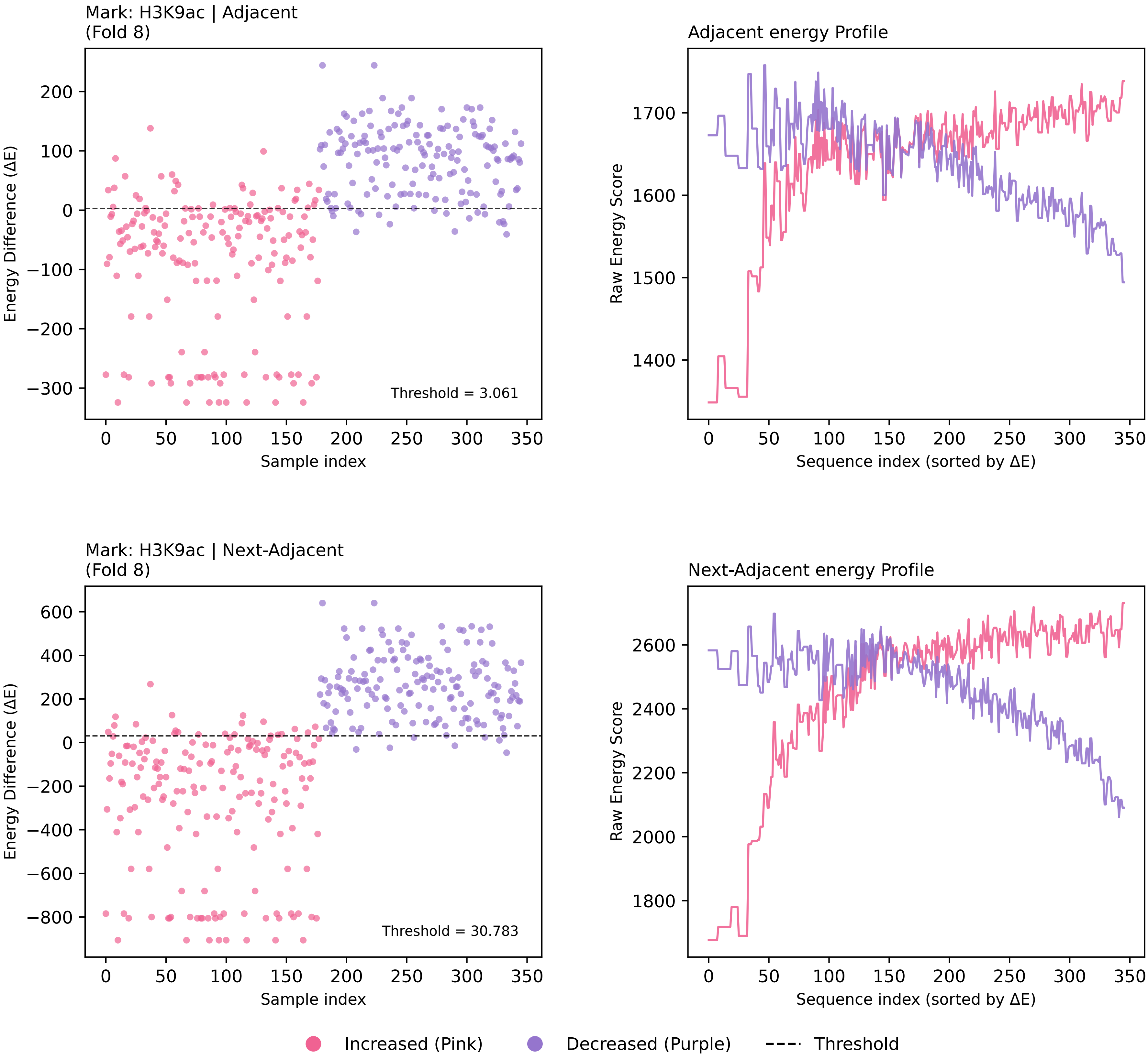

Figure S5 (Fold 8). Top: Adjacent; Bottom: Next-Adjacent.
Left panels: Scatter plots of energy differences (ΔE); Right panels: Raw energy score profile curves along the sorted sequences.

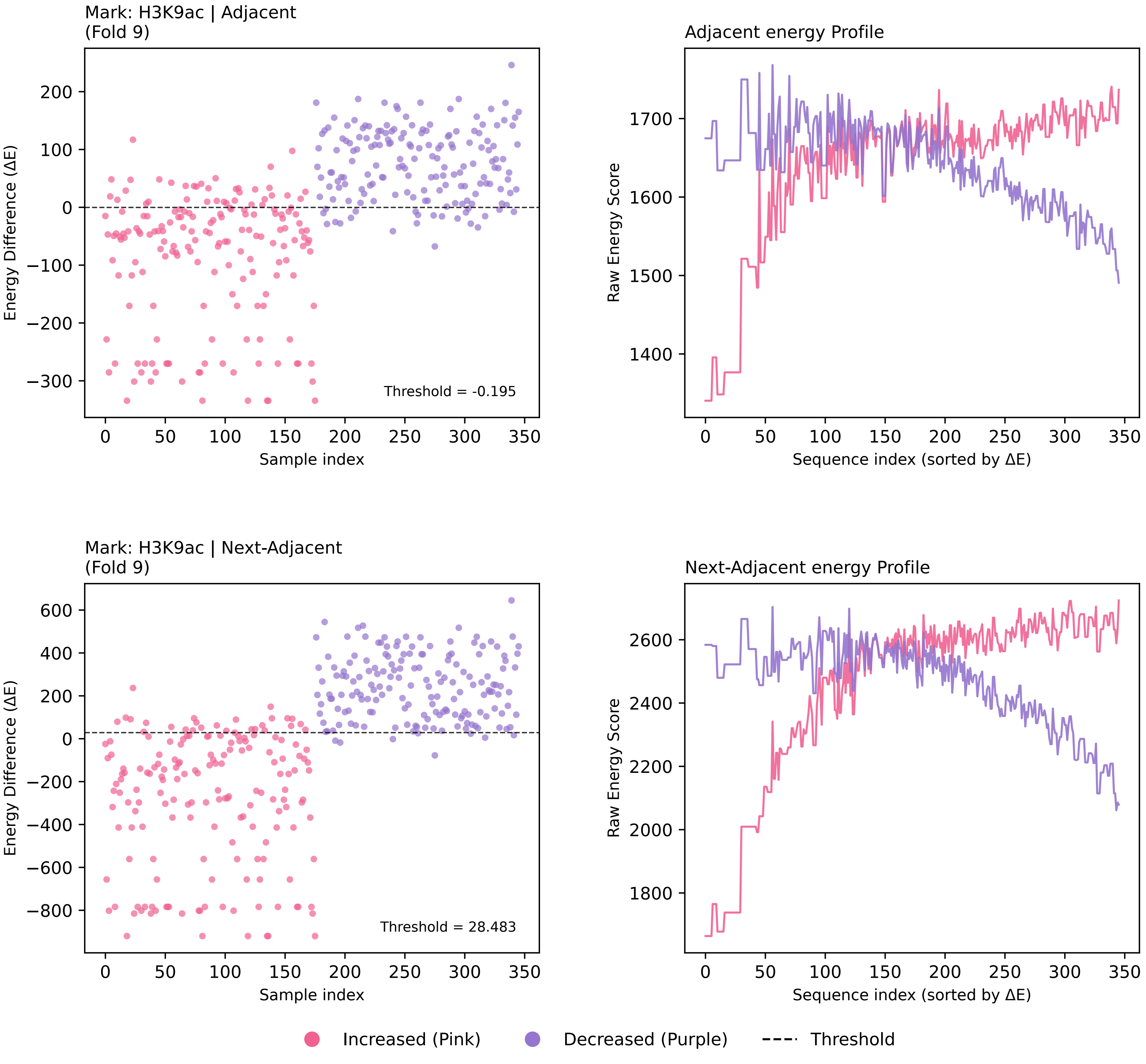

Figure S5 (Fold 9). Top: Adjacent; Bottom: Next-Adjacent.
Left panels: Scatter plots of energy differences (ΔE); Right panels: Raw energy score profile curves along the sorted sequences.

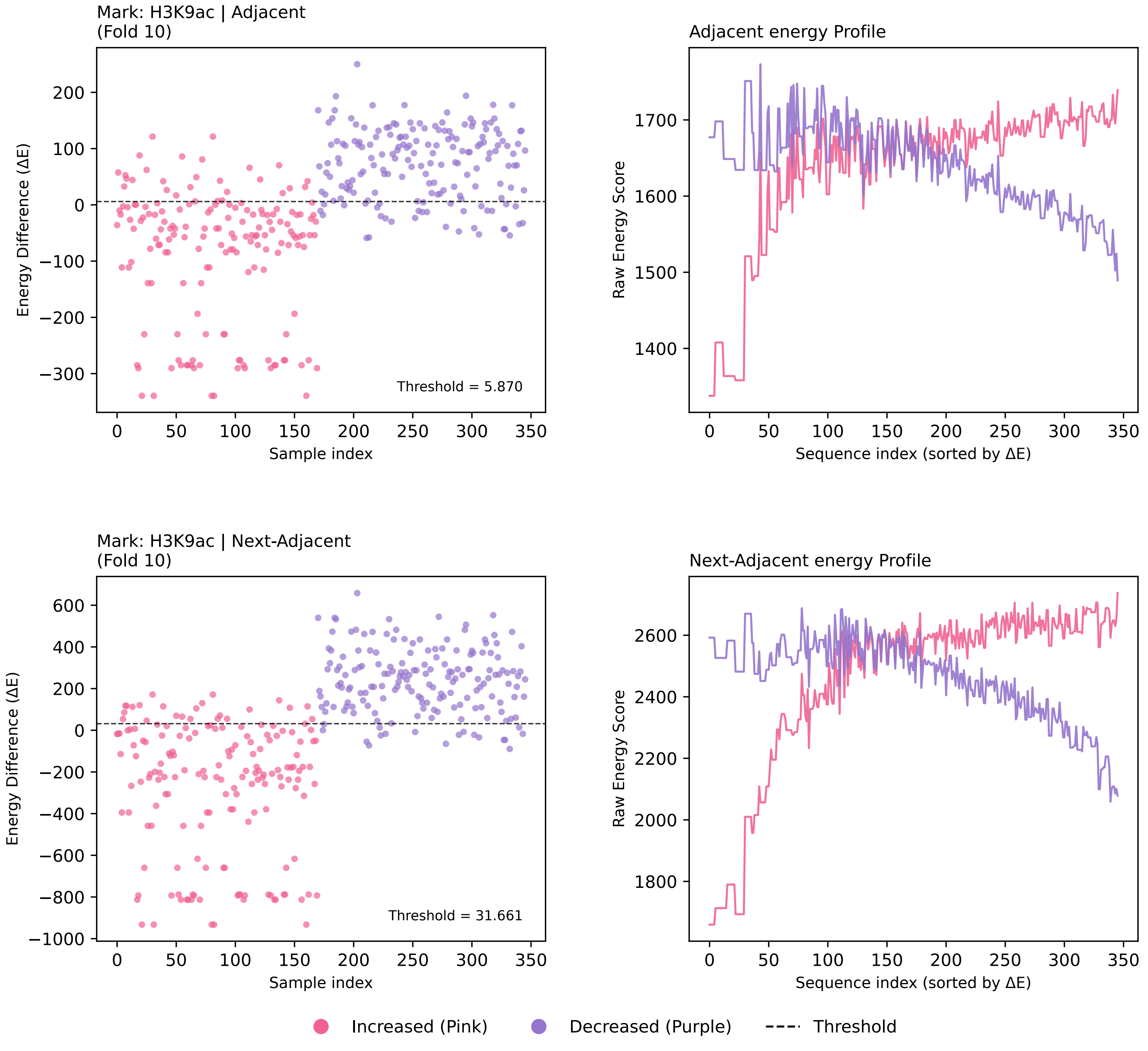

Figure S5 (Fold 10). Top: Adjacent; Bottom: Next-Adjacent.
Left panels: Scatter plots of energy differences (ΔE); Right panels: Raw energy score profile curves along the sorted sequences.
