# Supplementary material for: An Energy Model Based on Molecular Structure for Predicting Histone Modification Levels at lncRNA Promoter Regions in HepG2 Cells
Source: Int J Mol Sci. 2026 Jun 23;27(13):5653. doi: 10.3390/ijms27135653 (PMC13361589; doi:10.3390/ijms27135653)
Supplement: Supplementary file 1 [file ijms-27-05653-s001.zip › Figure_S6_H3K9me3_Report.pdf]

# Performance Metrics: H3K9me3
# (Folds 1 to 10)

Table S6. Supplementary table showing per-fold quantitative metrics for H3K9me3. All values are presented as mean ± confidence interval

| Model | Fold | Sn (%) | Sp (%) | Ac (%) | MCC | auROC |
|---|---|---|---|---|---|---|
| Adjacent | 1 | 90.0 | 87.5 | 87.0 | 0.775 | 0.971 |
| Adjacent | 2 | 91.111 | 86.792 | 96.667 | 0.777 | 0.973 |
| Adjacent | 3 | 77.551 | 91.837 | 84.694 | 0.701 | 0.951 |
| Adjacent | 4 | 94.828 | 80.0 | 75.0 | 0.768 | 0.967 |
| Adjacent | 5 | 92.453 | 91.111 | 84.906 | 0.836 | 0.966 |
| Adjacent | 6 | 94.444 | 95.455 | 86.111 | 0.897 | 0.992 |
| Adjacent | 7 | 93.182 | 88.679 | 100.0 | 0.815 | 0.971 |
| Adjacent | 8 | 94.737 | 94.915 | 121.053 | 0.893 | 0.986 |
| Adjacent | 9 | 88.095 | 89.091 | 102.381 | 0.77 | 0.962 |
| Adjacent | 10 | 92.727 | 85.714 | 79.091 | 0.79 | 0.969 |
| Next-Adjacent | 1 | 88.0 | 100.0 | 92.0 | 0.884 | 0.981 |
| Next-Adjacent | 2 | 86.667 | 96.226 | 100.0 | 0.837 | 0.979 |
| Next-Adjacent | 3 | 75.51 | 95.918 | 85.714 | 0.73 | 0.959 |
| Next-Adjacent | 4 | 84.483 | 92.5 | 74.138 | 0.758 | 0.978 |
| Next-Adjacent | 5 | 77.358 | 95.556 | 79.245 | 0.732 | 0.969 |
| Next-Adjacent | 6 | 90.741 | 100.0 | 86.111 | 0.903 | 0.998 |
| Next-Adjacent | 7 | 79.545 | 98.113 | 98.864 | 0.8 | 0.98 |
| Next-Adjacent | 8 | 94.737 | 94.915 | 121.053 | 0.893 | 0.992 |
| Next-Adjacent | 9 | 83.333 | 100.0 | 107.143 | 0.86 | 0.987 |
| Next-Adjacent | 10 | 87.273 | 90.476 | 78.182 | 0.772 | 0.978 |

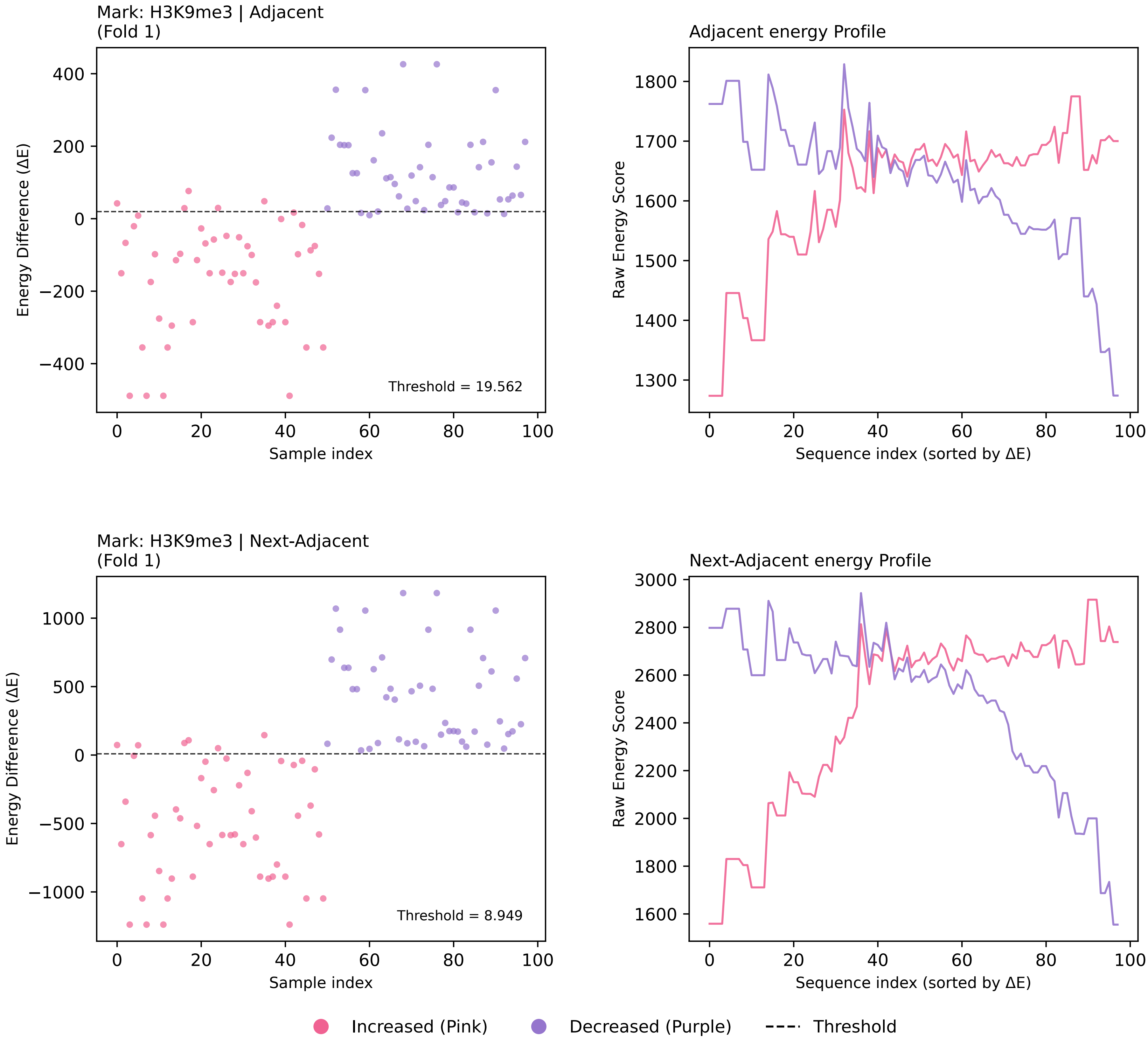


Figure S6 (Fold 1). Top: Adjacent; Bottom: Next-Adjacent.
Left panels: Scatter plots of energy differences (ΔE); Right panels: Raw energy score profile curves along the sorted sequences.

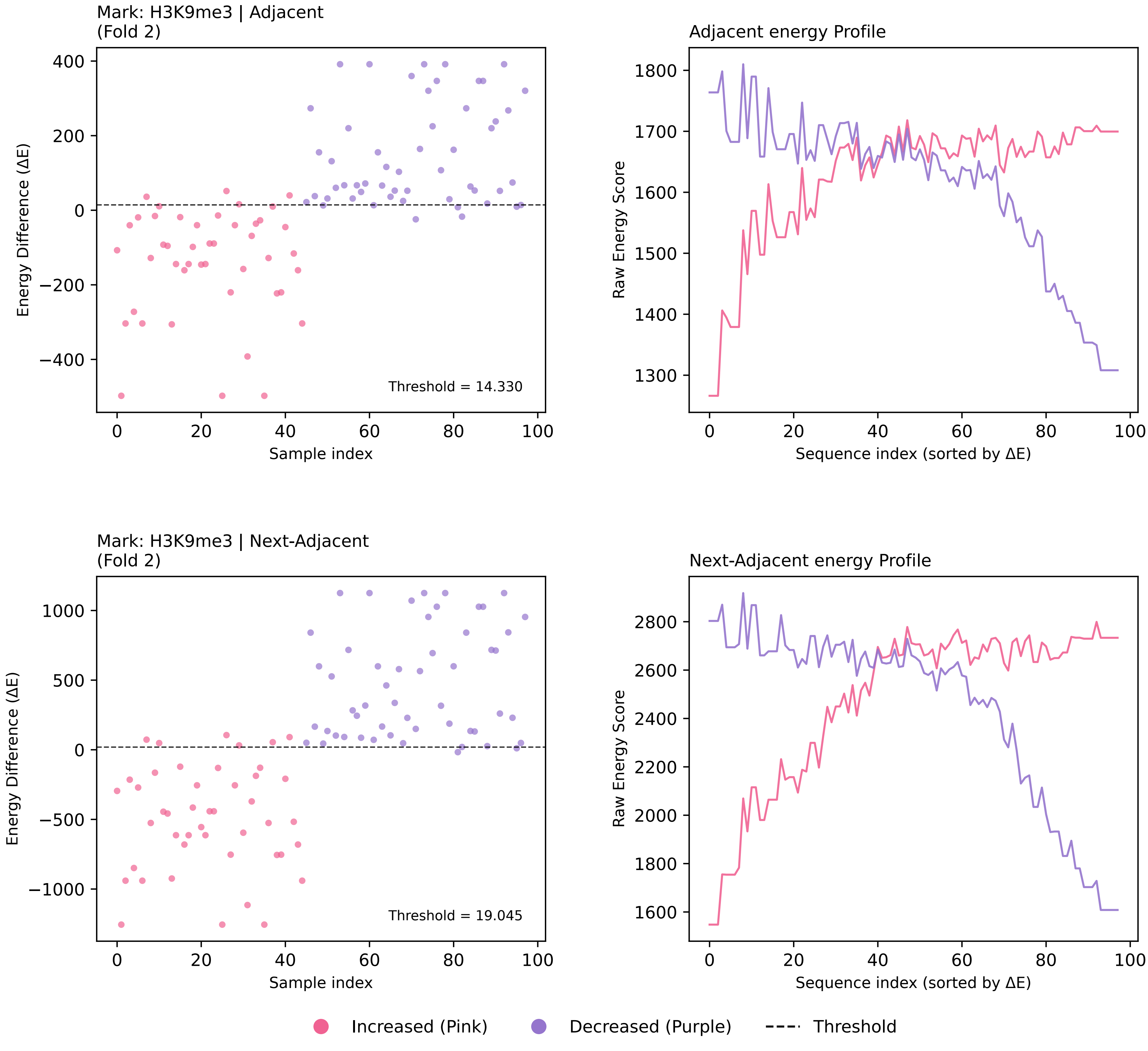


Figure S6 (Fold 2). Top: Adjacent; Bottom: Next-Adjacent.
Left panels: Scatter plots of energy differences (ΔE); Right panels: Raw energy score profile curves along the sorted sequences.

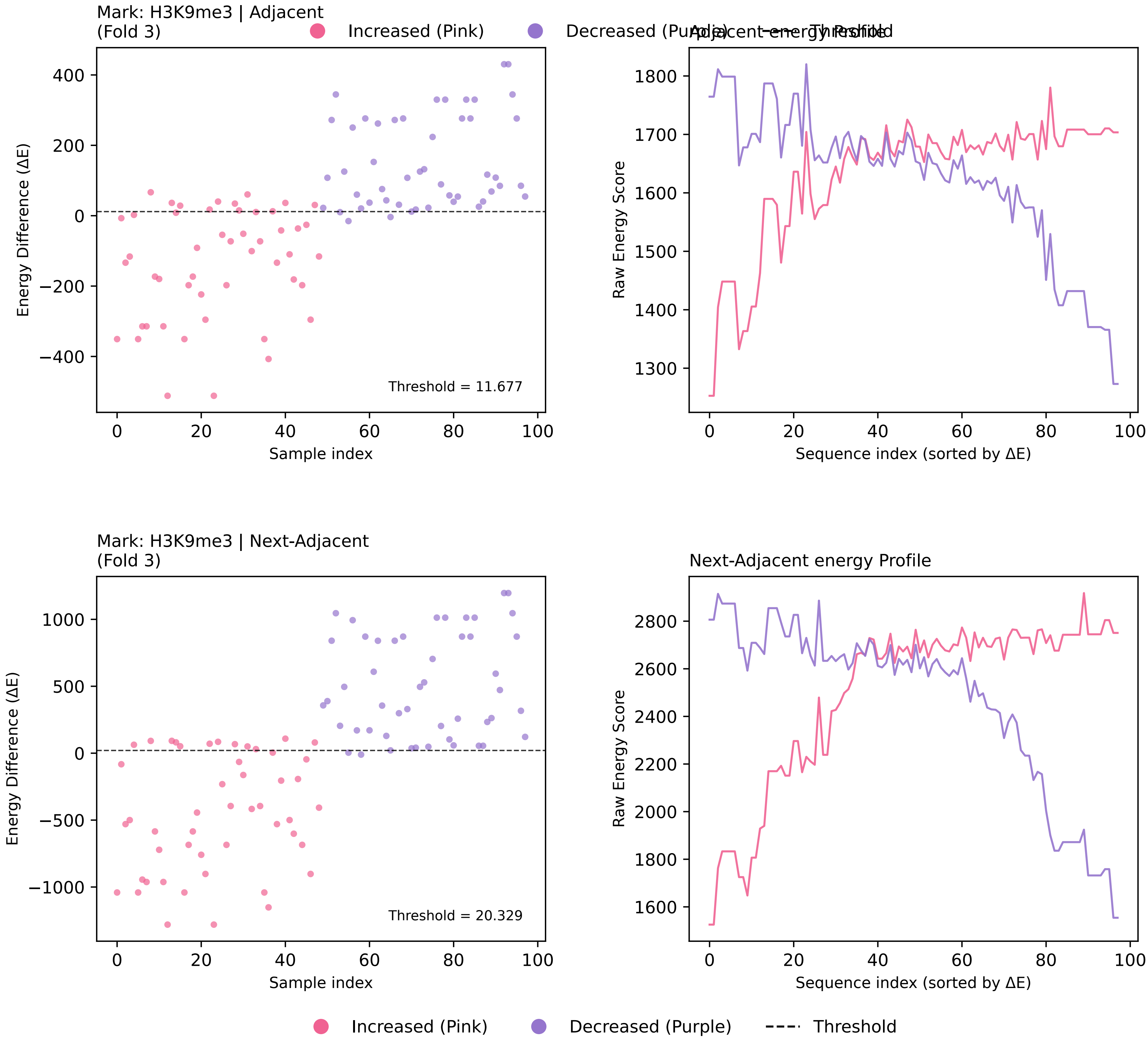


Figure S6 (Fold 3). Top: Adjacent; Bottom: Next-Adjacent.
Left panels: Scatter plots of energy differences (ΔE); Right panels: Raw energy score profile curves along the sorted sequences.

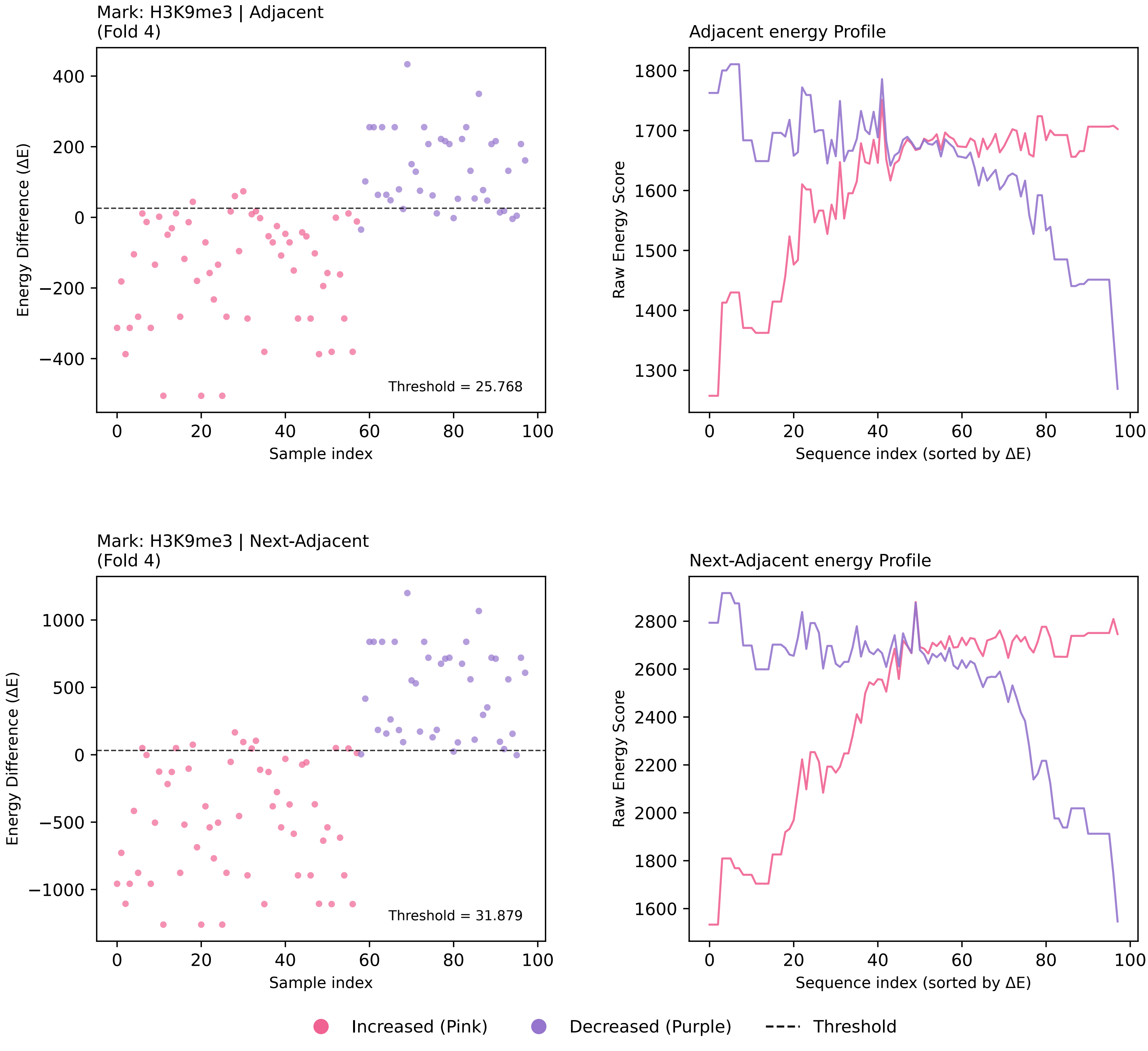


Figure S6 (Fold 4). Top: Adjacent; Bottom: Next-Adjacent.
Left panels: Scatter plots of energy differences (ΔE); Right panels: Raw energy score profile curves along the sorted sequences.

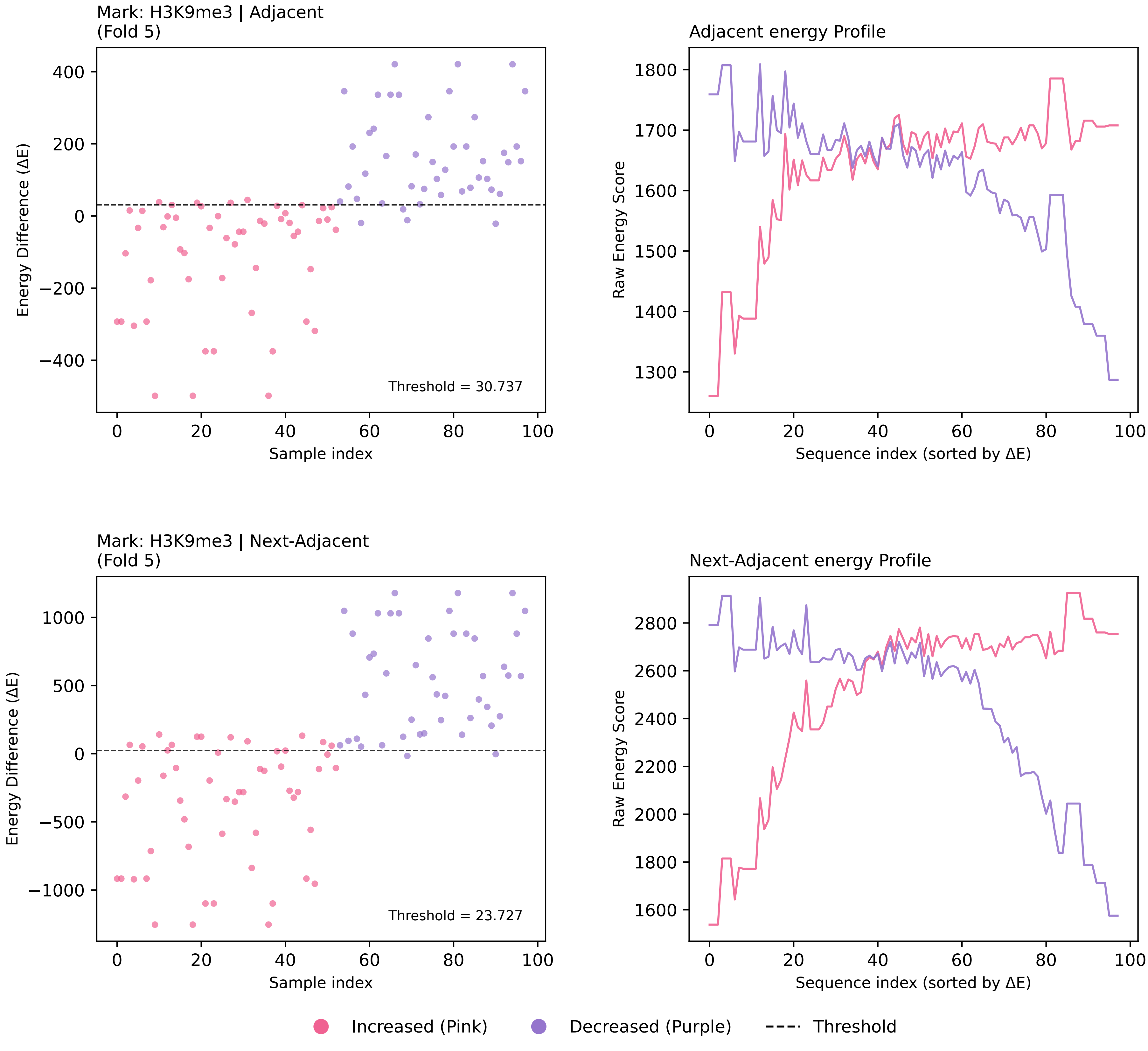


Figure S6 (Fold 5). Top: Adjacent; Bottom: Next-Adjacent.
Left panels: Scatter plots of energy differences (ΔE); Right panels: Raw energy score profile curves along the sorted sequences.

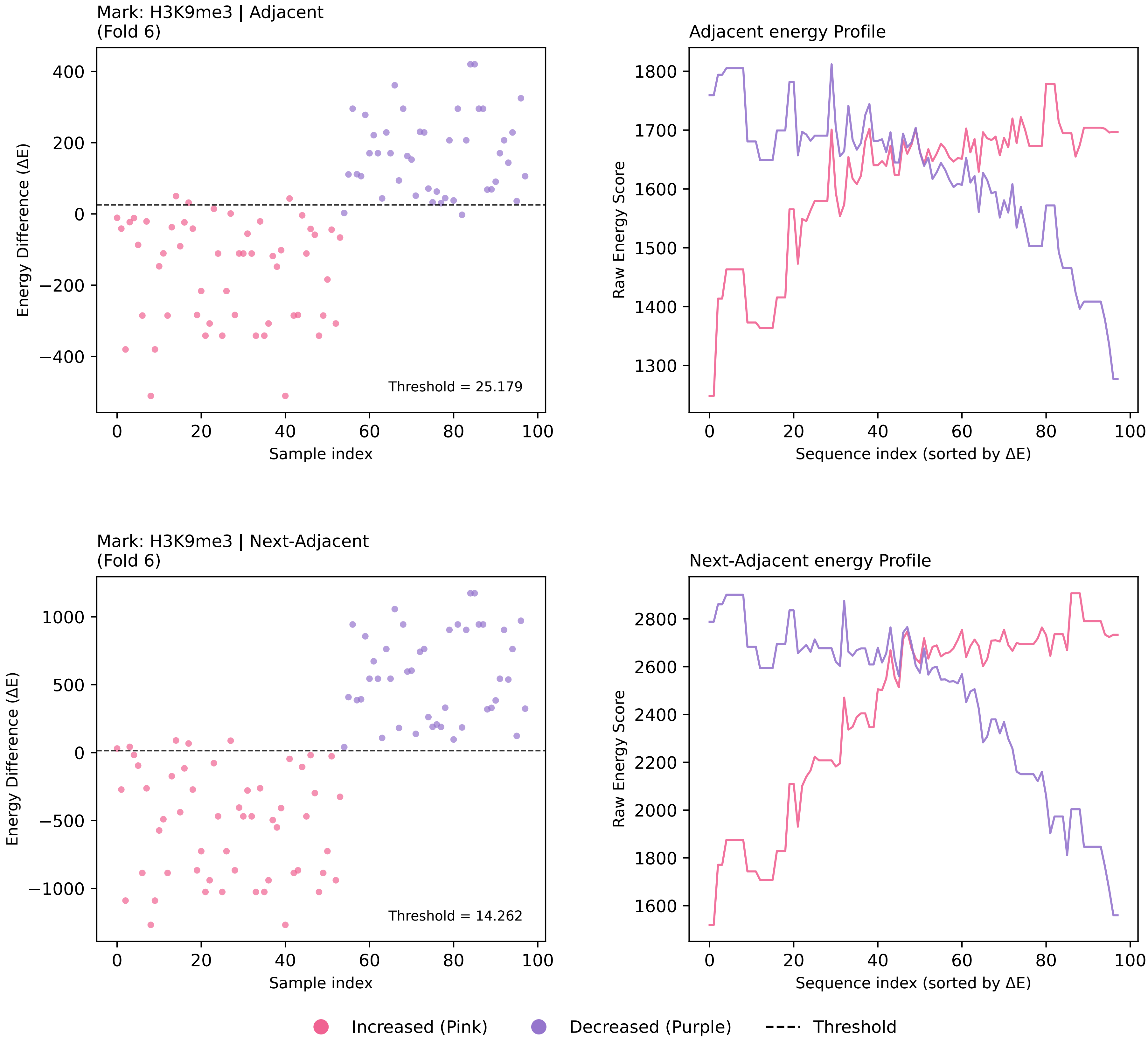


Figure S6 (Fold 6). Top: Adjacent; Bottom: Next-Adjacent.
Left panels: Scatter plots of energy differences (ΔE); Right panels: Raw energy score profile curves along the sorted sequences.

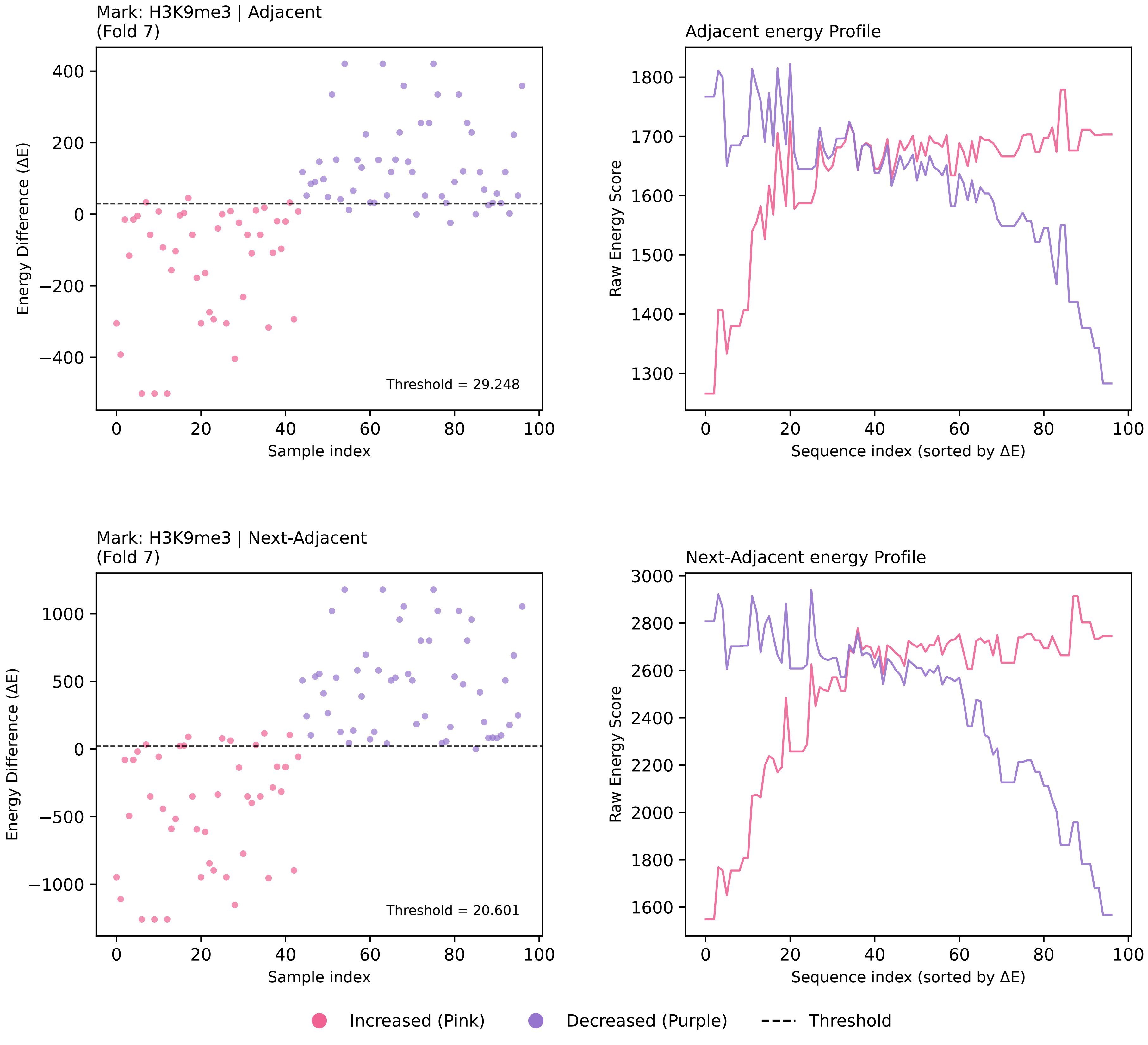


Figure S6 (Fold 7). Top: Adjacent; Bottom: Next-Adjacent.
Left panels: Scatter plots of energy differences (ΔE); Right panels: Raw energy score profile curves along the sorted sequences.

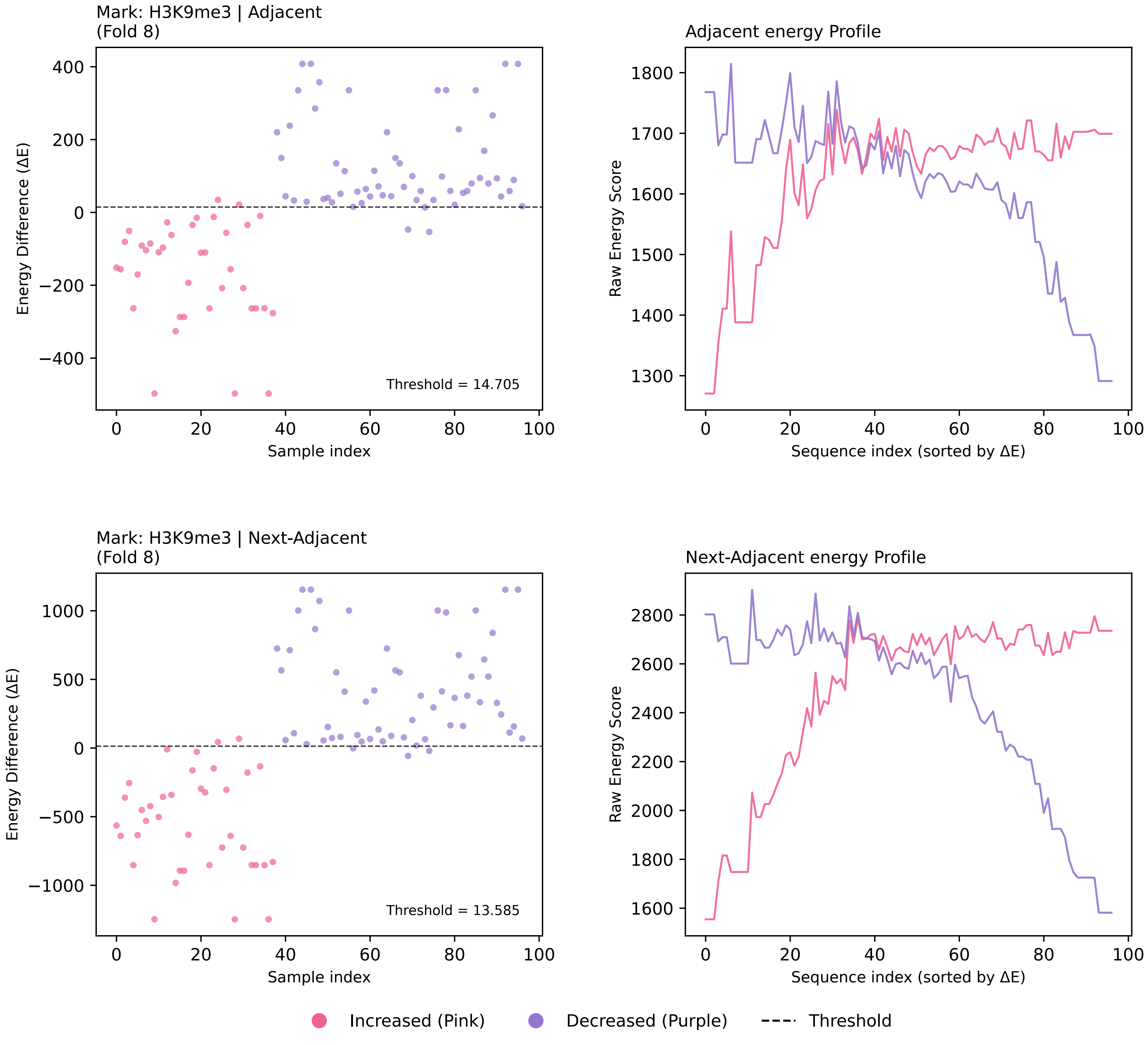


Figure S6 (Fold 8). Top: Adjacent; Bottom: Next-Adjacent.
Left panels: Scatter plots of energy differences (ΔE); Right panels: Raw energy score profile curves along the sorted sequences.

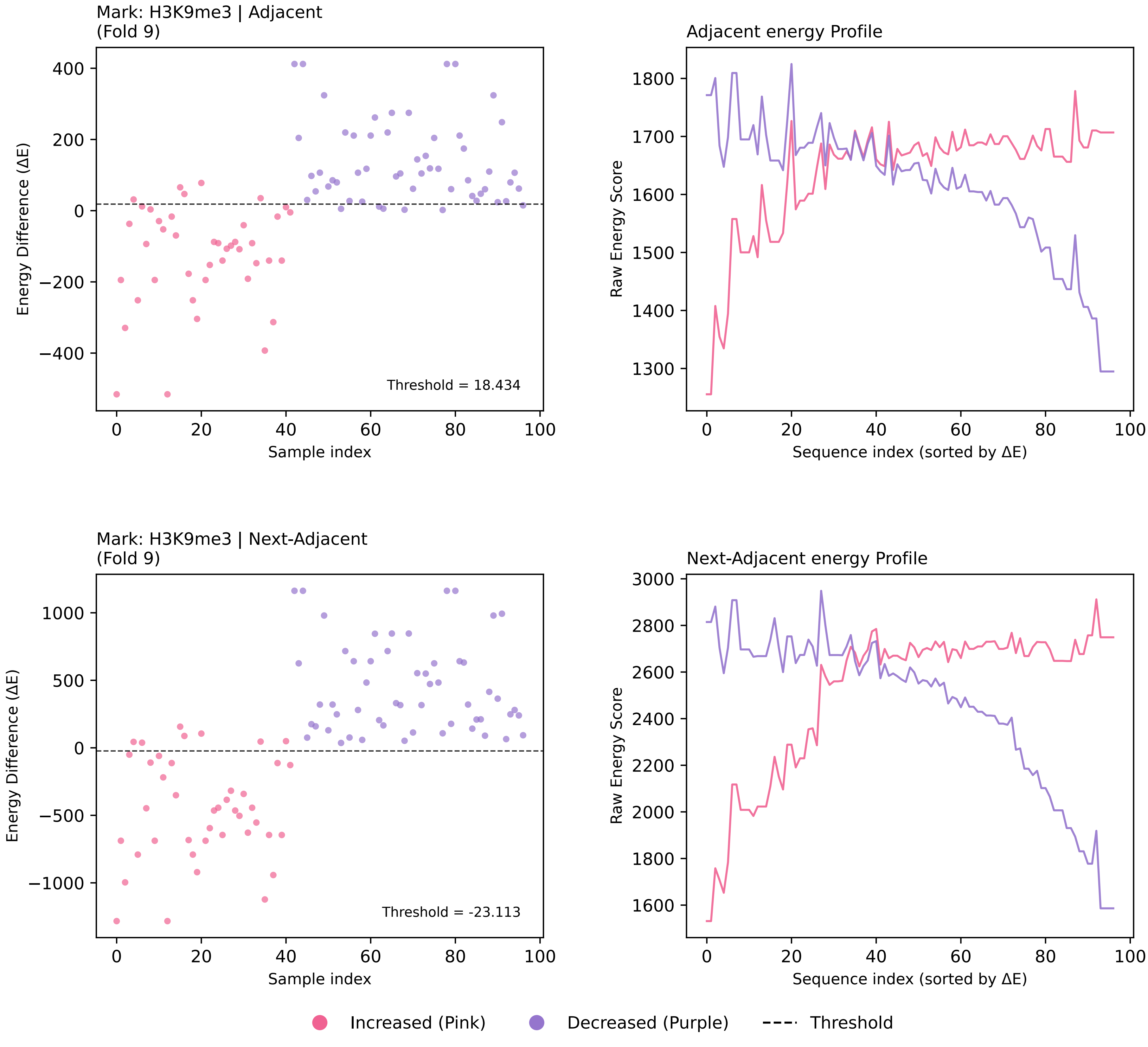


Figure S6 (Fold 9). Top: Adjacent; Bottom: Next-Adjacent.
Left panels: Scatter plots of energy differences (ΔE); Right panels: Raw energy score profile curves along the sorted sequences.

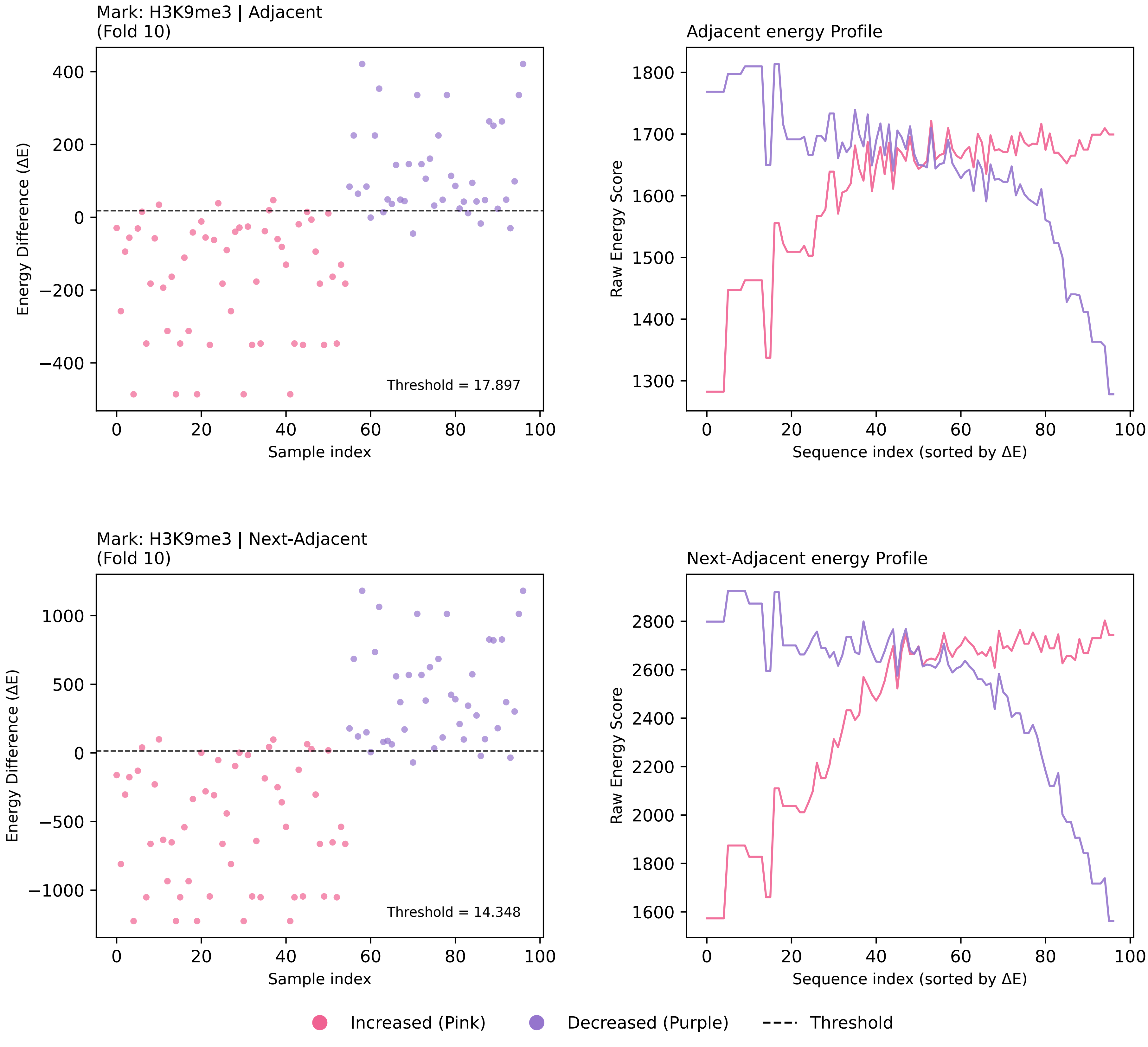


Figure S6 (Fold 10). Top: Adjacent; Bottom: Next-Adjacent.
Left panels: Scatter plots of energy differences (ΔE); Right panels: Raw energy score profile curves along the sorted sequences.
