# Supplementary material for: An Energy Model Based on Molecular Structure for Predicting Histone Modification Levels at lncRNA Promoter Regions in HepG2 Cells
Source: Int J Mol Sci. 2026 Jun 23;27(13):5653. doi: 10.3390/ijms27135653 (PMC13361589; doi:10.3390/ijms27135653)
Supplement: Supplementary file 1 [file ijms-27-05653-s001.zip › Figure_S7_H4K20me1_Report.pdf]

Performance Metrics: H4K20me1
(Folds 1 to 10)

Table S7. Supplementary table showing per-fold quantitative metrics for H4K20me1. All values are presented as mean ± confidence interval

Model	Fold	Sn (%)	Sp (%)	Ac (%)	MCC	auROC
Adjacent	1	90.217	82.0	89.674	0.723	0.928
Adjacent	2	91.429	85.057	80.952	0.769	0.954
Adjacent	3	94.382	83.495	95.506	0.778	0.962
Adjacent	4	95.098	90.0	87.255	0.854	0.966
Adjacent	5	86.408	86.364	80.097	0.727	0.936
Adjacent	6	94.059	75.556	80.693	0.713	0.924
Adjacent	7	90.625	88.421	89.062	0.791	0.953
Adjacent	8	90.323	79.592	87.097	0.702	0.938
Adjacent	9	94.937	83.929	106.962	0.777	0.949
Adjacent	10	88.66	79.787	82.99	0.688	0.932
Next-Adjacent	1	90.217	87.0	92.391	0.772	0.966
Next-Adjacent	2	94.286	86.207	82.857	0.811	0.976
Next-Adjacent	3	93.258	88.35	97.753	0.814	0.982
Next-Adjacent	4	95.098	90.0	87.255	0.854	0.982
Next-Adjacent	5	91.262	92.045	84.951	0.832	0.975
Next-Adjacent	6	90.099	85.556	83.168	0.758	0.964
Next-Adjacent	7	91.667	85.263	88.021	0.771	0.97
Next-Adjacent	8	91.398	87.755	91.935	0.791	0.97
Next-Adjacent	9	96.203	87.5	110.127	0.826	0.975
Next-Adjacent	10	88.66	92.553	89.175	0.812	0.97

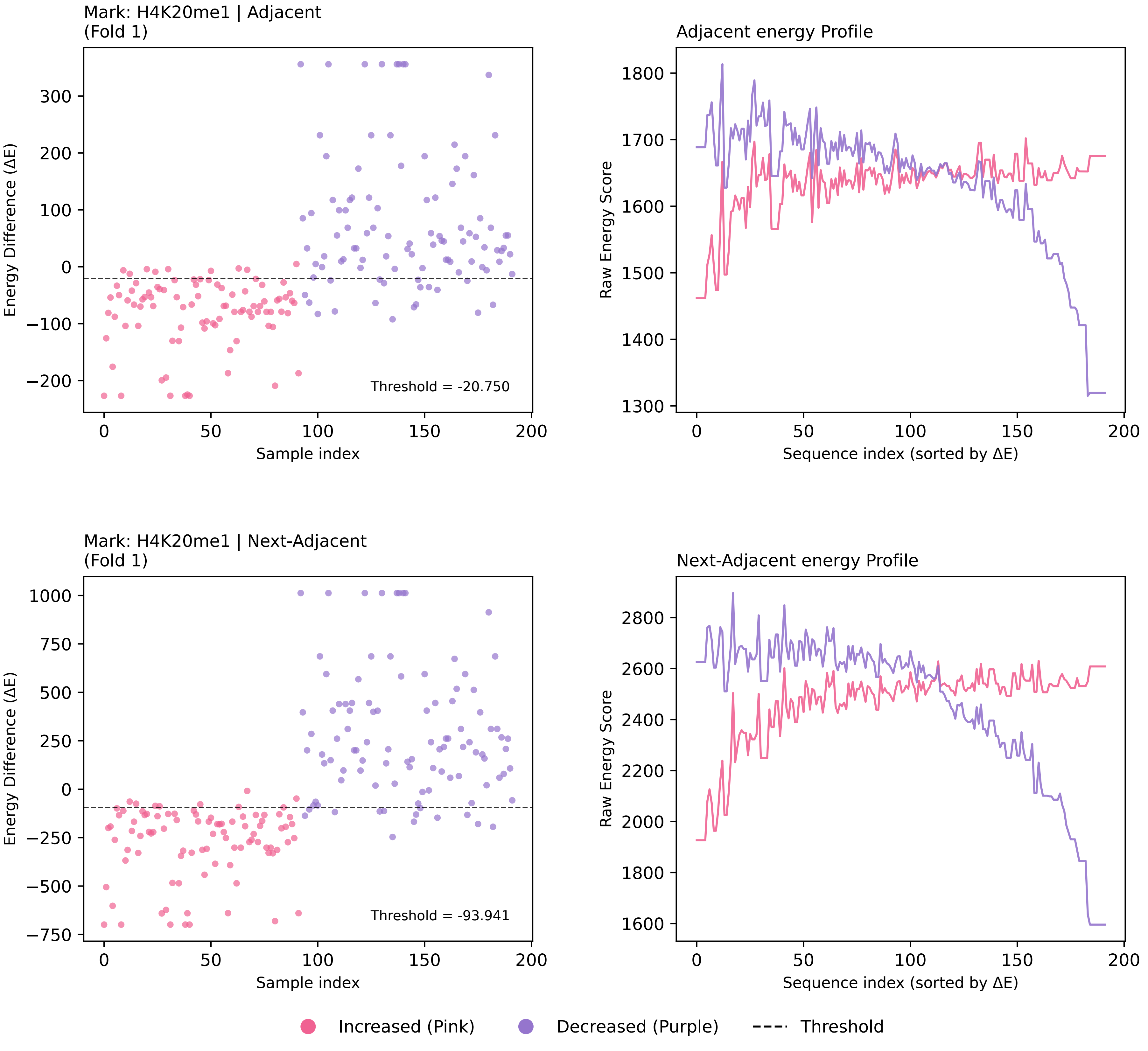

Figure S7 (Fold 1). Top: Adjacent; Bottom: Next-Adjacent.
Left panels: Scatter plots of energy differences (ΔE); Right panels: Raw energy score profile curves along the sorted sequences.

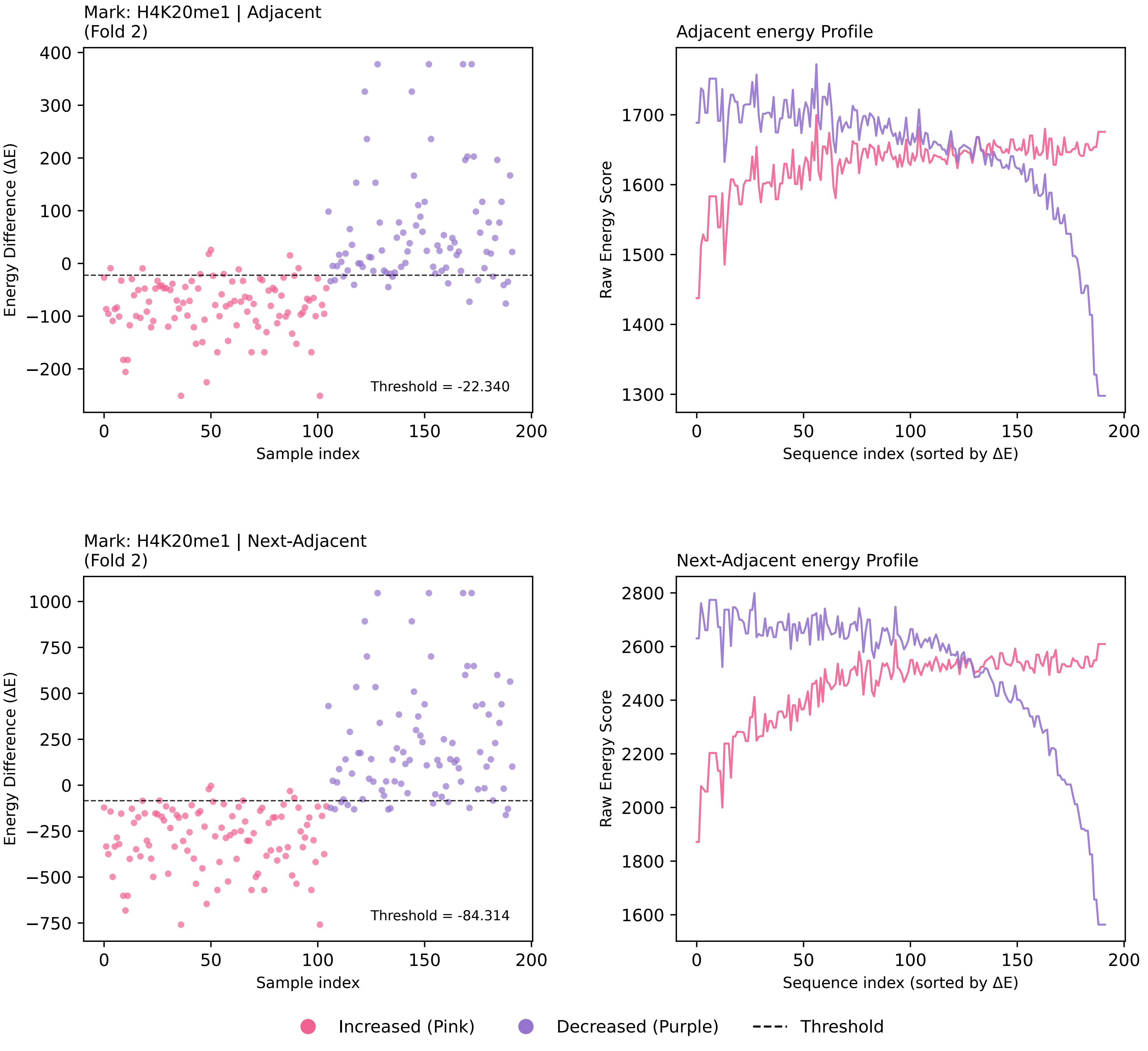

Figure S7 (Fold 2). Top: Adjacent; Bottom: Next-Adjacent.
Left panels: Scatter plots of energy differences (ΔE); Right panels: Raw energy score profile curves along the sorted sequences.

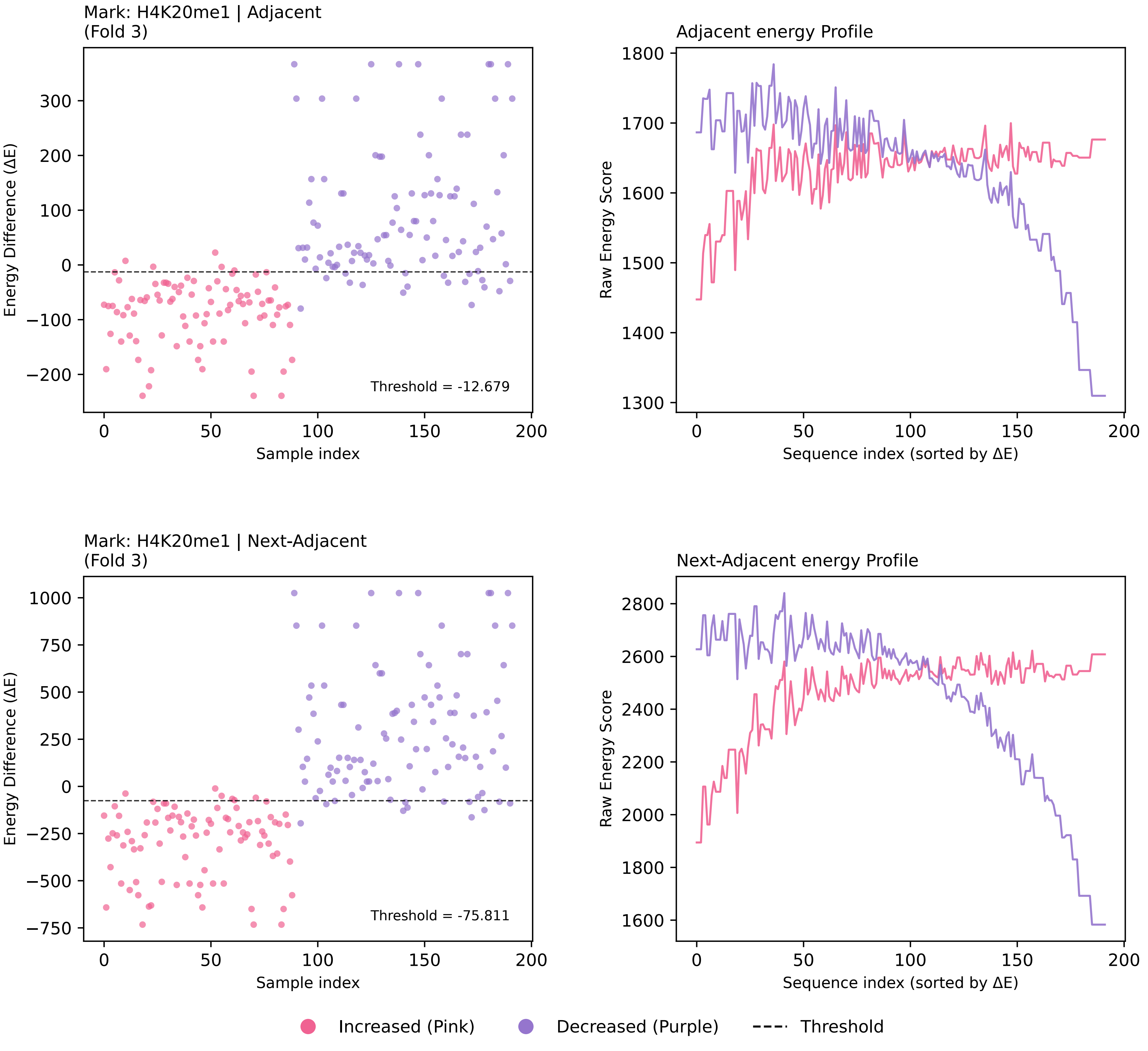

Figure S7 (Fold 3). Top: Adjacent; Bottom: Next-Adjacent.
Left panels: Scatter plots of energy differences (ΔE); Right panels: Raw energy score profile curves along the sorted sequences.

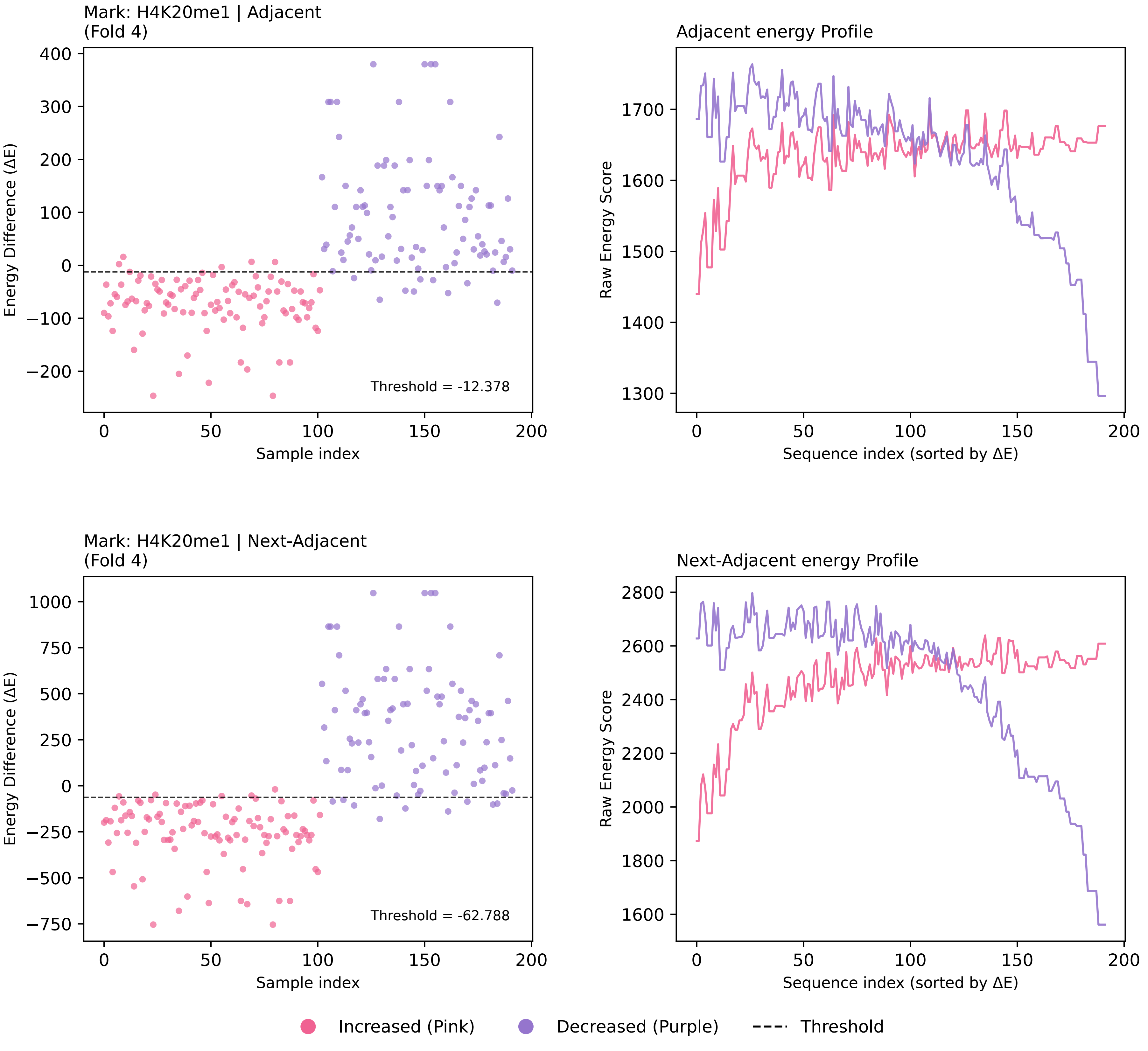

Figure S7 (Fold 4). Top: Adjacent; Bottom: Next-Adjacent.
Left panels: Scatter plots of energy differences (ΔE); Right panels: Raw energy score profile curves along the sorted sequences.

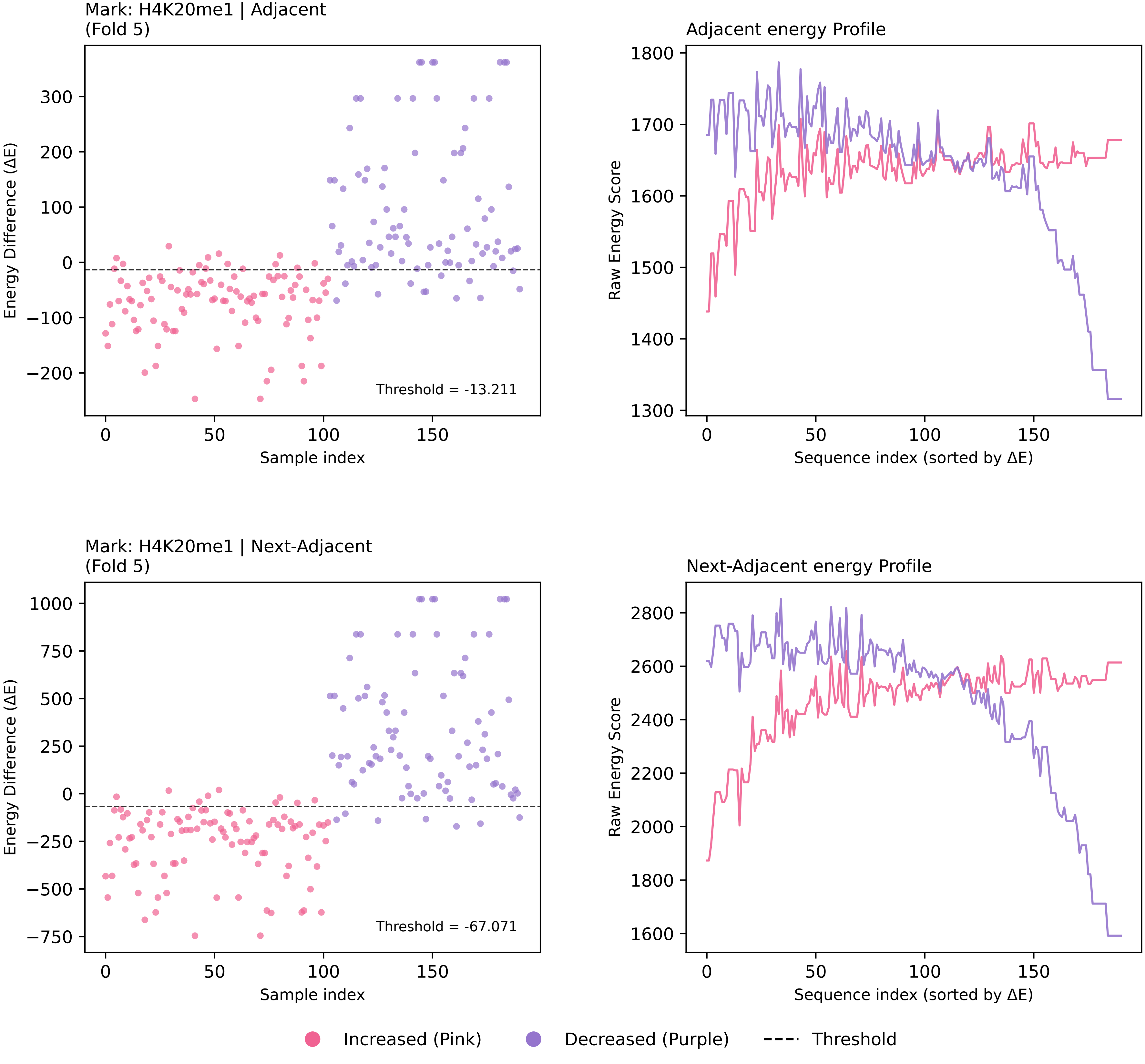

Figure S7 (Fold 5). Top: Adjacent; Bottom: Next-Adjacent.
Left panels: Scatter plots of energy differences (ΔE); Right panels: Raw energy score profile curves along the sorted sequences.

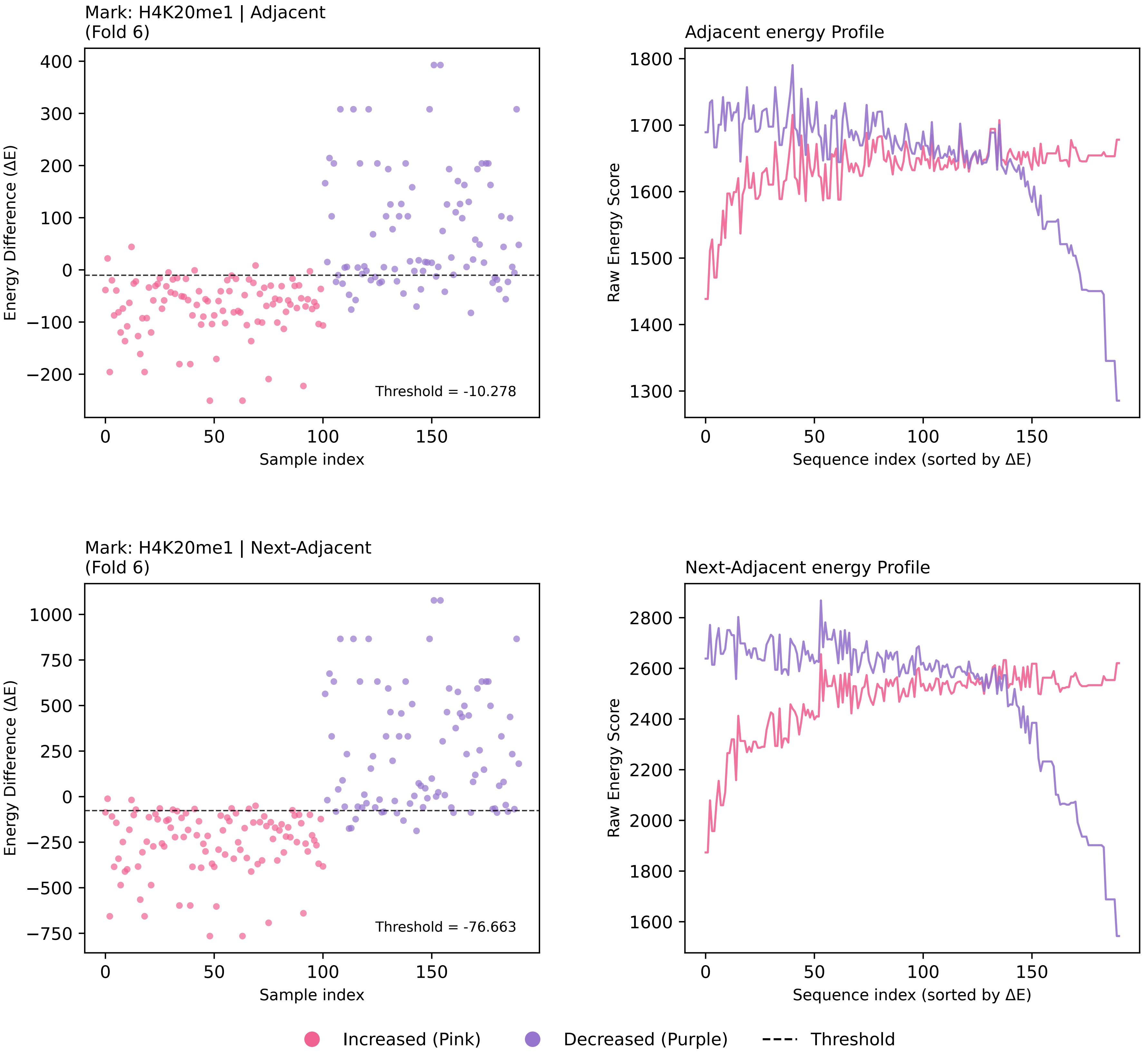

Figure S7 (Fold 6). Top: Adjacent; Bottom: Next-Adjacent.
Left panels: Scatter plots of energy differences (ΔE); Right panels: Raw energy score profile curves along the sorted sequences.

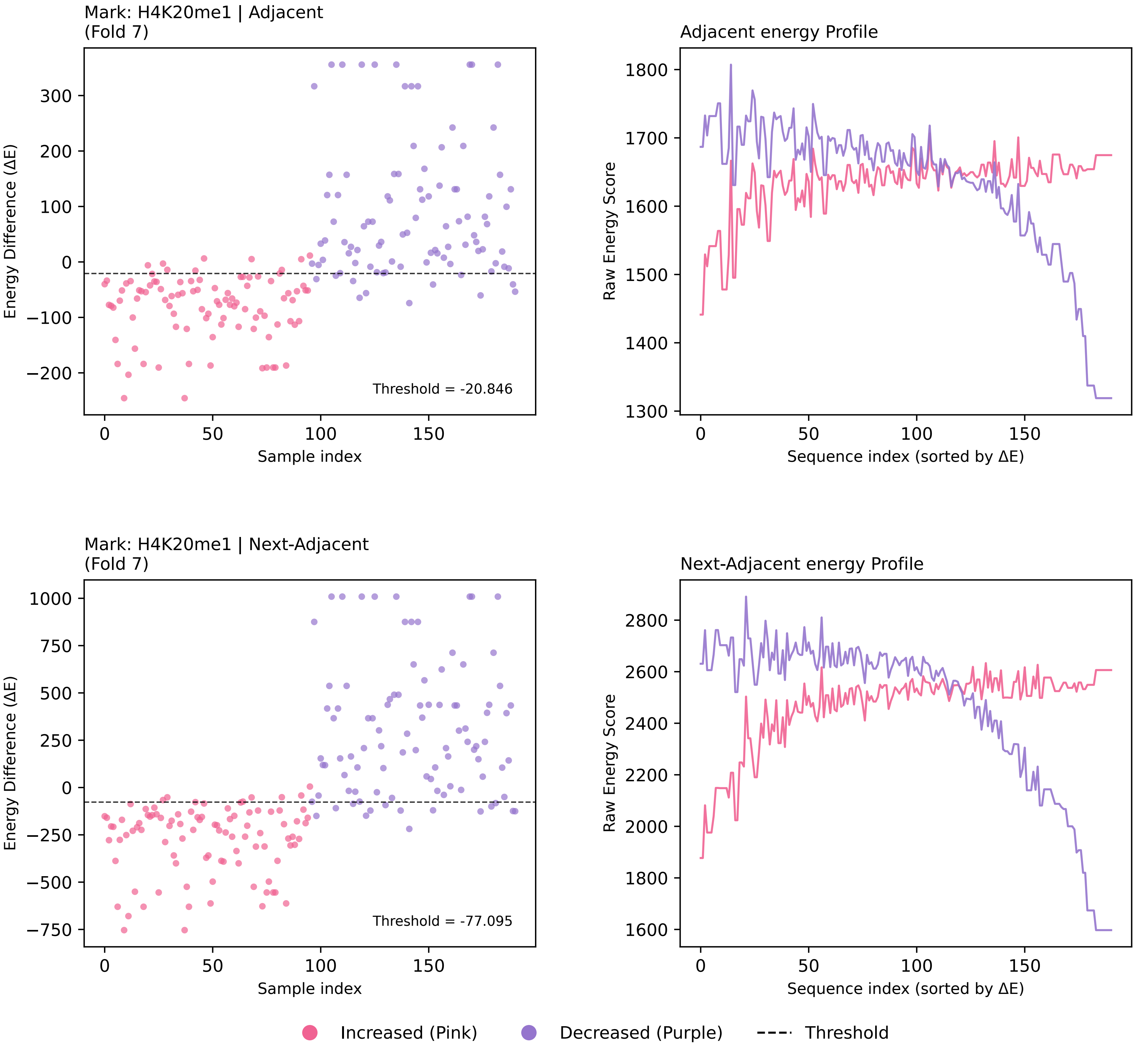

Figure S7 (Fold 7). Top: Adjacent; Bottom: Next-Adjacent.
Left panels: Scatter plots of energy differences (ΔE); Right panels: Raw energy score profile curves along the sorted sequences.

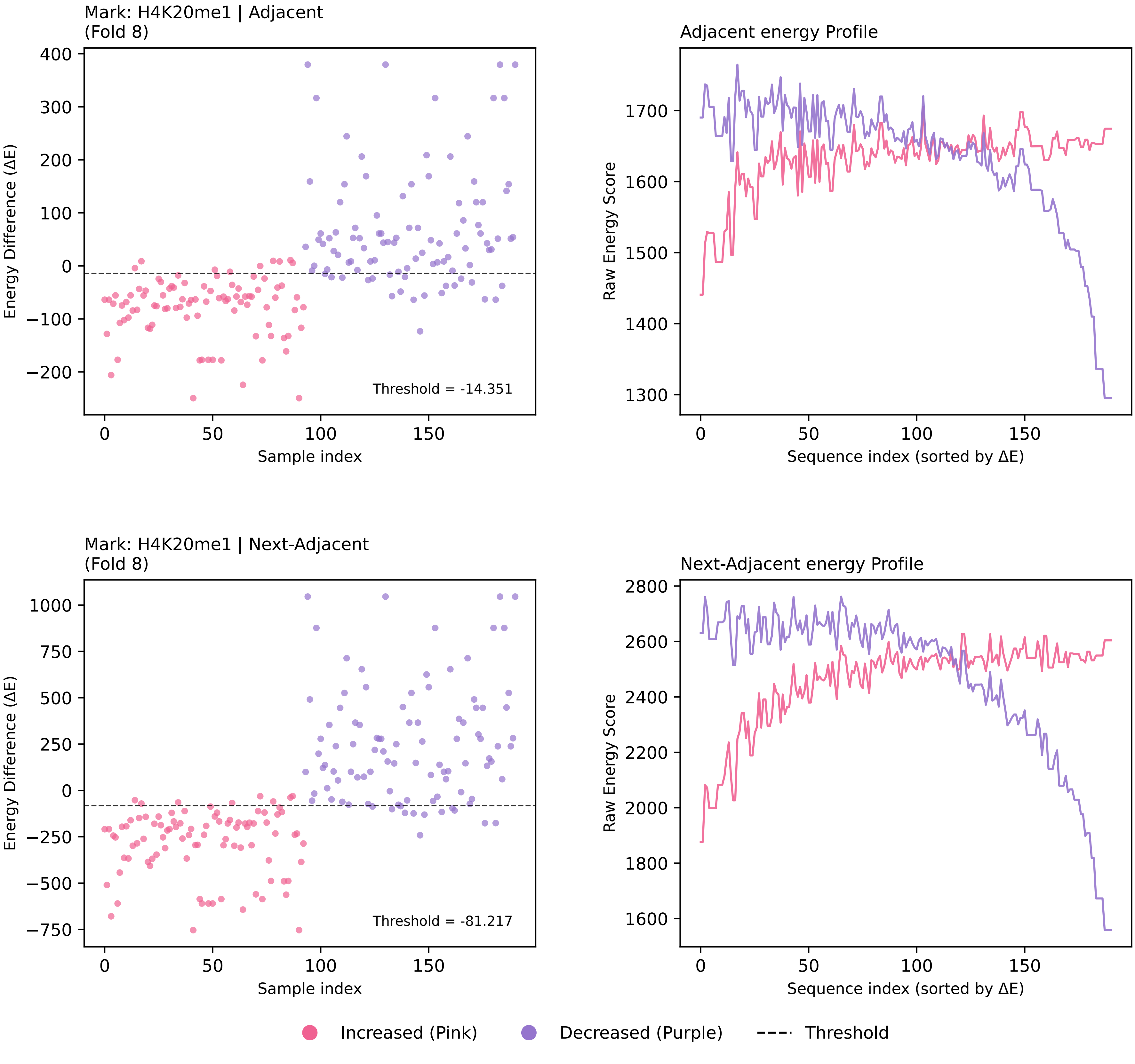

Figure S7 (Fold 8). Top: Adjacent; Bottom: Next-Adjacent.
Left panels: Scatter plots of energy differences (ΔE); Right panels: Raw energy score profile curves along the sorted sequences.

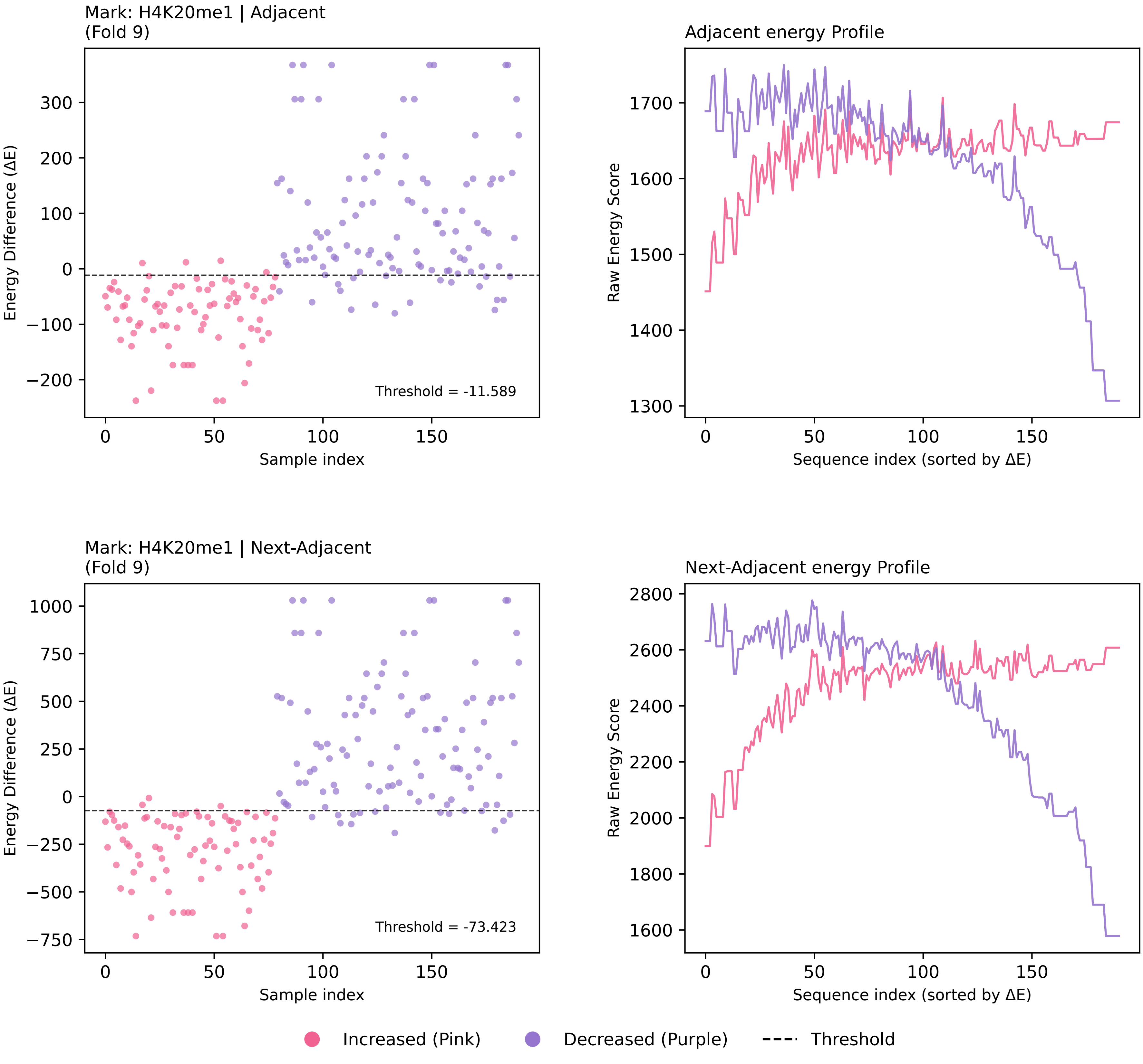

Figure S7 (Fold 9). Top: Adjacent; Bottom: Next-Adjacent.
Left panels: Scatter plots of energy differences (ΔE); Right panels: Raw energy score profile curves along the sorted sequences.

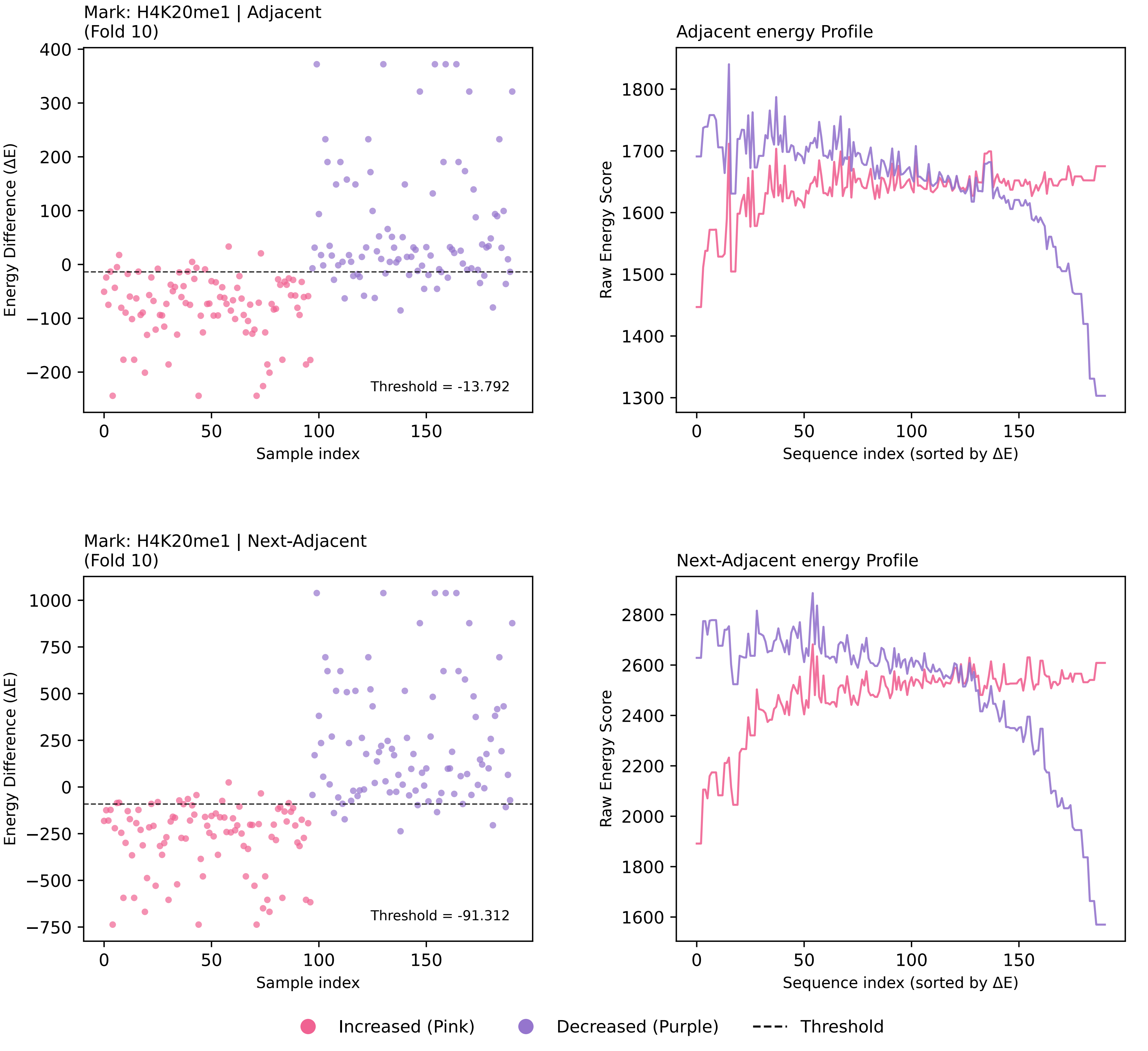

Figure S7 (Fold 10). Top: Adjacent; Bottom: Next-Adjacent.
Left panels: Scatter plots of energy differences (ΔE); Right panels: Raw energy score profile curves along the sorted sequences.
