# Supplementary material for: An Energy Model Based on Molecular Structure for Predicting Histone Modification Levels at lncRNA Promoter Regions in HepG2 Cells
Source: Int J Mol Sci. 2026 Jun 23;27(13):5653. doi: 10.3390/ijms27135653 (PMC13361589; doi:10.3390/ijms27135653)
Supplement: Supplementary file 1 [file ijms-27-05653-s001.zip › Figure_S8_H3K4me3_Report.pdf]

Performance Metrics: H3K4me3
(Folds 2 to 10)

Table S8_H3K4me3. Supplementary table showing per-fold quantitative metrics for H3K4me3.All values are presented as mean ± confidence interval (CI).

Model	Fold	Sn (%)	Sp (%)	Ac (%)	MCC	auROC
Adjacent	2	89.316	70.0	82.051	0.602	0.9
Adjacent	3	89.453	85.526	82.812	0.751	0.939
Adjacent	4	90.873	81.466	82.937	0.728	0.926
Adjacent	5	86.345	77.447	79.719	0.641	0.901
Adjacent	6	88.21	76.863	86.9	0.652	0.896
Adjacent	7	83.465	80.786	78.15	0.643	0.912
Adjacent	8	84.255	87.5	88.298	0.718	0.932
Adjacent	9	86.076	77.642	83.333	0.639	0.904
Adjacent	10	89.401	78.195	92.627	0.673	0.925
Next-Adjacent	2	92.308	84.8	91.453	0.772	0.96
Next-Adjacent	3	92.188	92.544	87.305	0.847	0.979
Next-Adjacent	4	93.254	90.948	88.492	0.843	0.977
Next-Adjacent	5	89.96	88.936	86.948	0.789	0.958
Next-Adjacent	6	93.013	84.706	93.668	0.777	0.964
Next-Adjacent	7	88.189	93.45	86.22	0.815	0.973
Next-Adjacent	8	89.787	94.355	94.681	0.843	0.975
Next-Adjacent	9	88.608	84.959	88.397	0.736	0.957
Next-Adjacent	10	91.705	92.857	102.765	0.845	0.978

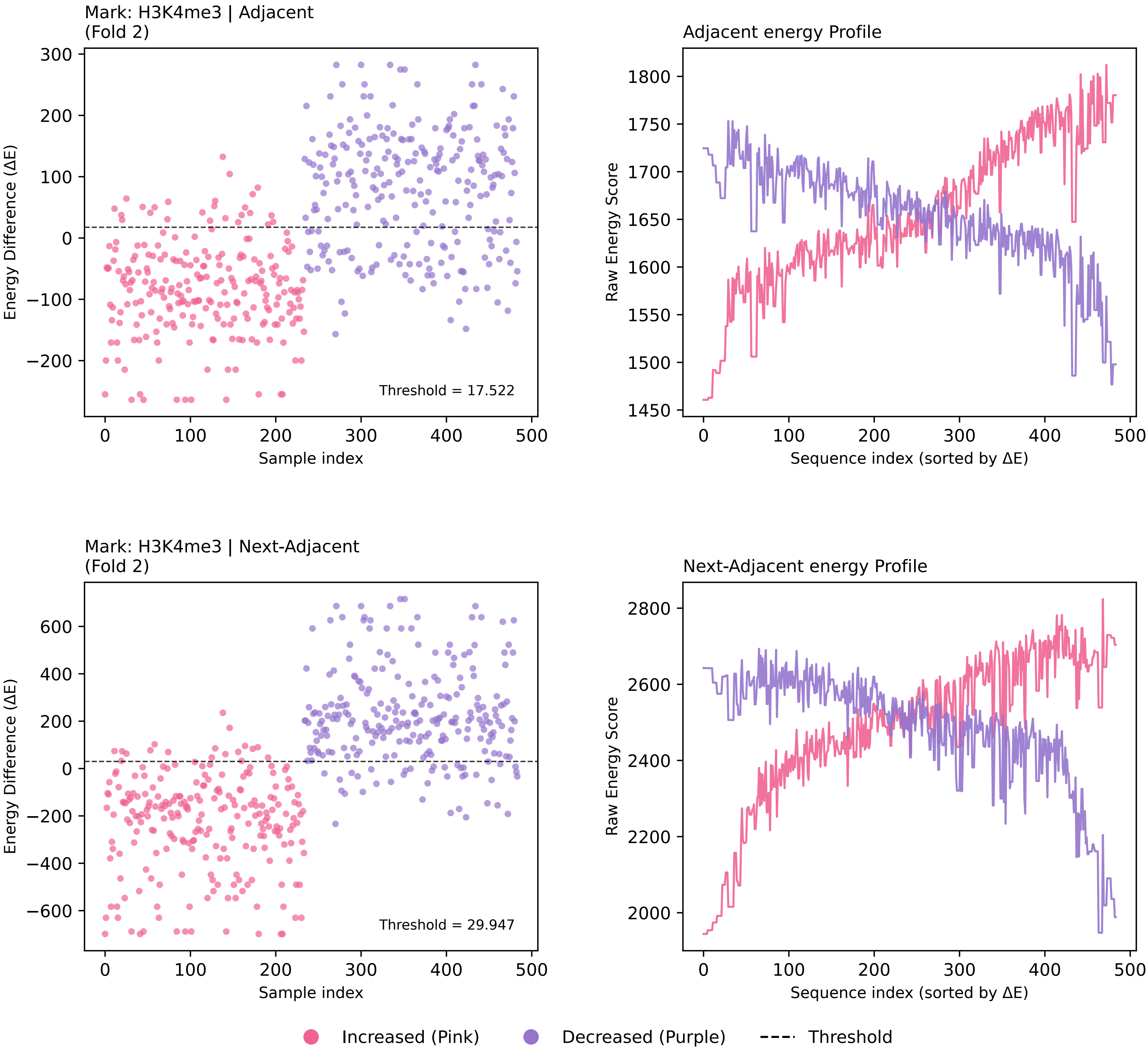

Figure S_Core_Remain_H3K4me3 (Fold 2). Top: Adjacent; Bottom: Next-Adjacent.
Left panels: Scatter plots of energy differences (ΔE); Right panels: Raw energy score profile curves along the sorted sequences.

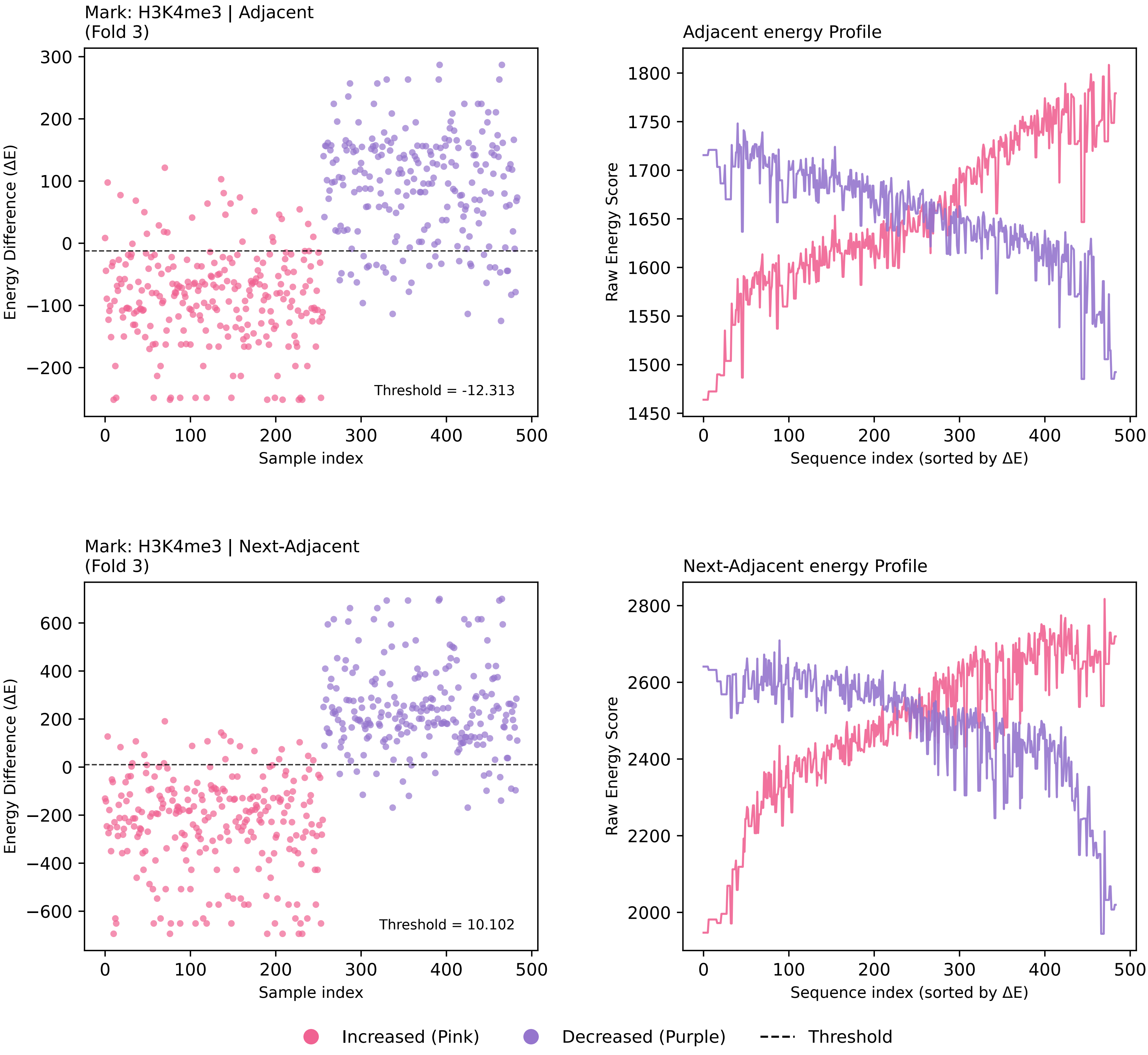

Figure S_Core_Remain_H3K4me3 (Fold 3). Top: Adjacent; Bottom: Next-Adjacent.
Left panels: Scatter plots of energy differences (ΔE); Right panels: Raw energy score profile curves along the sorted sequences.

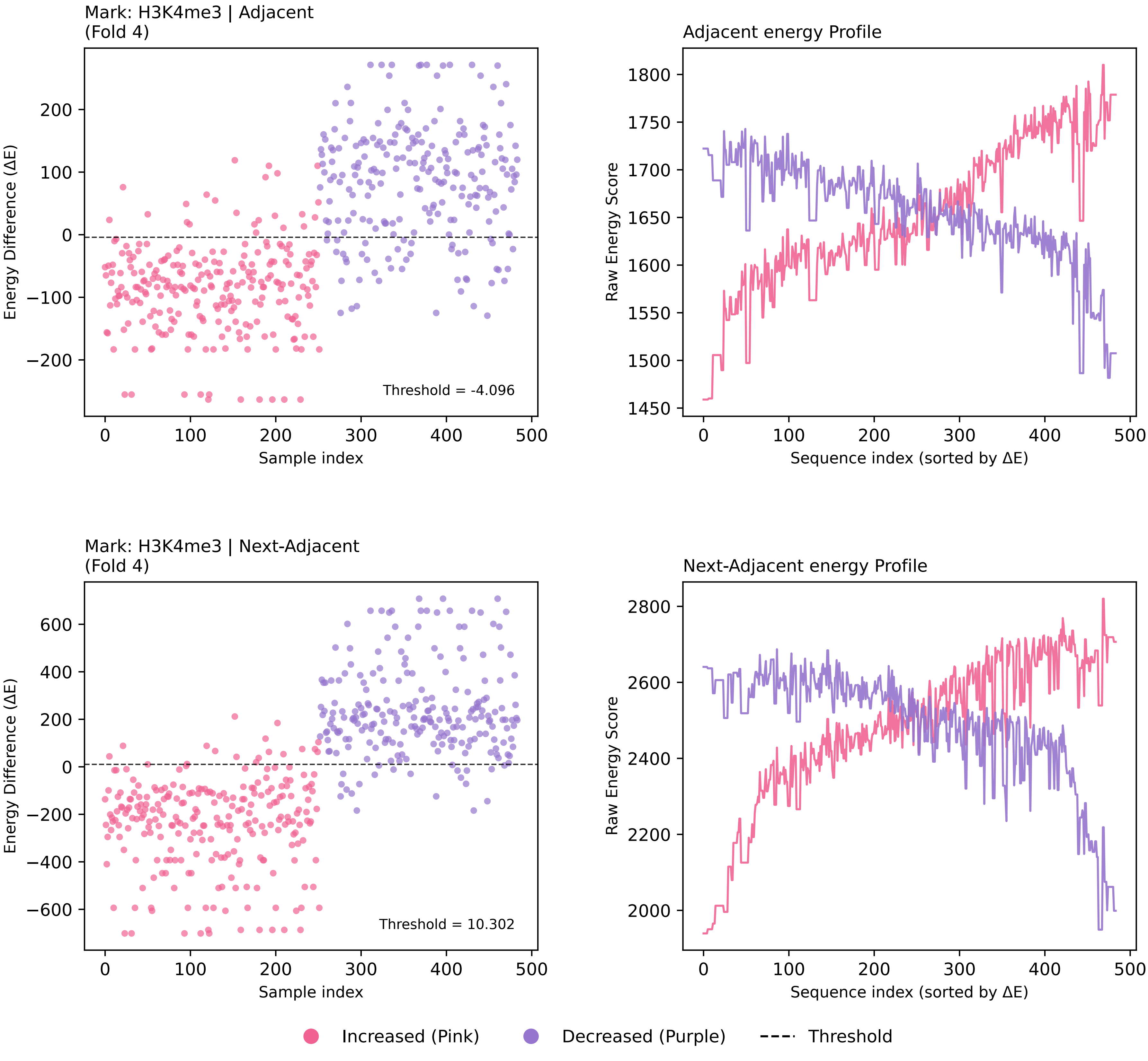

Figure S_Core_Remain_H3K4me3 (Fold 4). Top: Adjacent; Bottom: Next-Adjacent.
Left panels: Scatter plots of energy differences (ΔE); Right panels: Raw energy score profile curves along the sorted sequences.

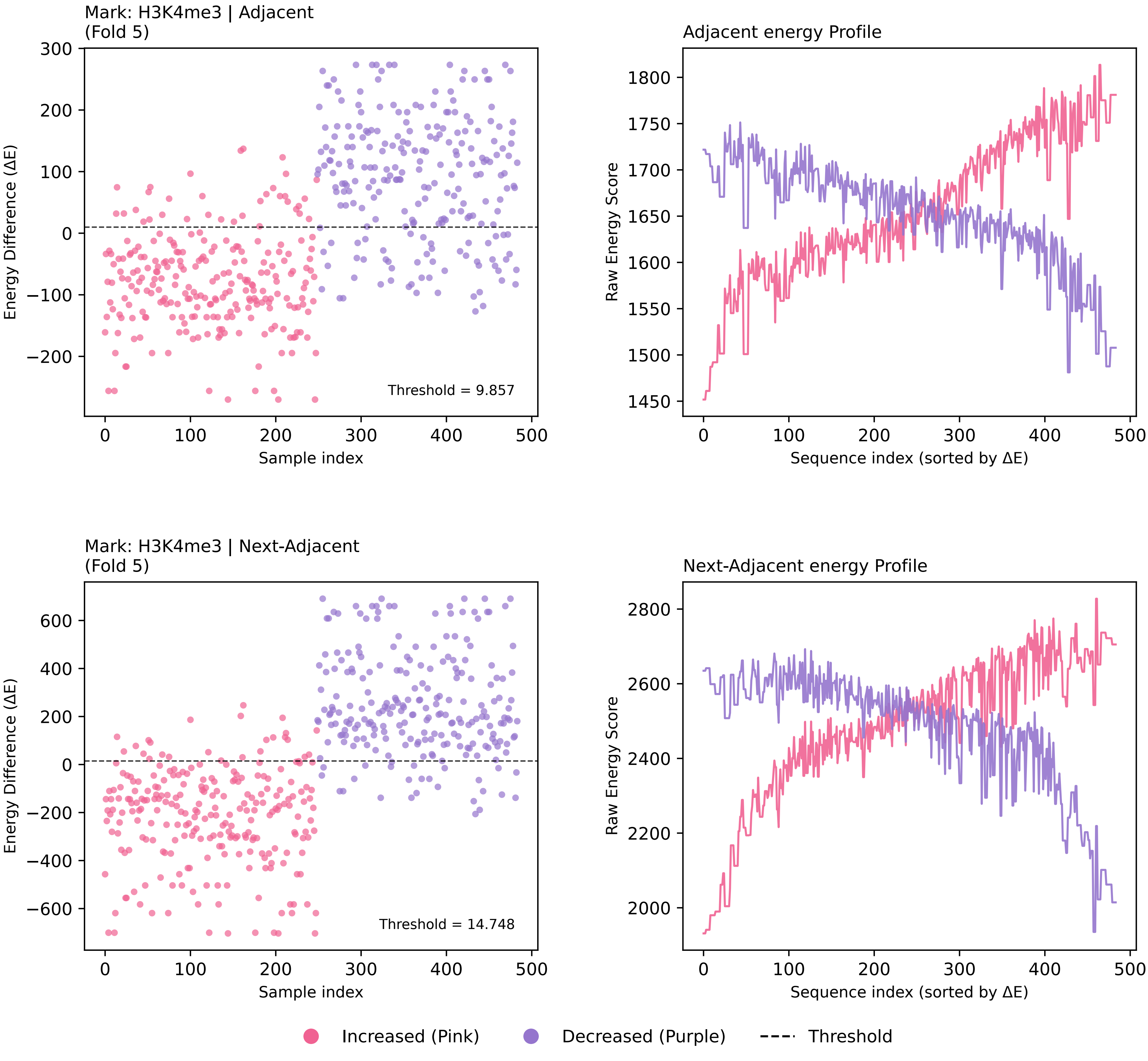

Figure S_Core_Remain_H3K4me3 (Fold 5). Top: Adjacent; Bottom: Next-Adjacent.
Left panels: Scatter plots of energy differences (ΔE); Right panels: Raw energy score profile curves along the sorted sequences.

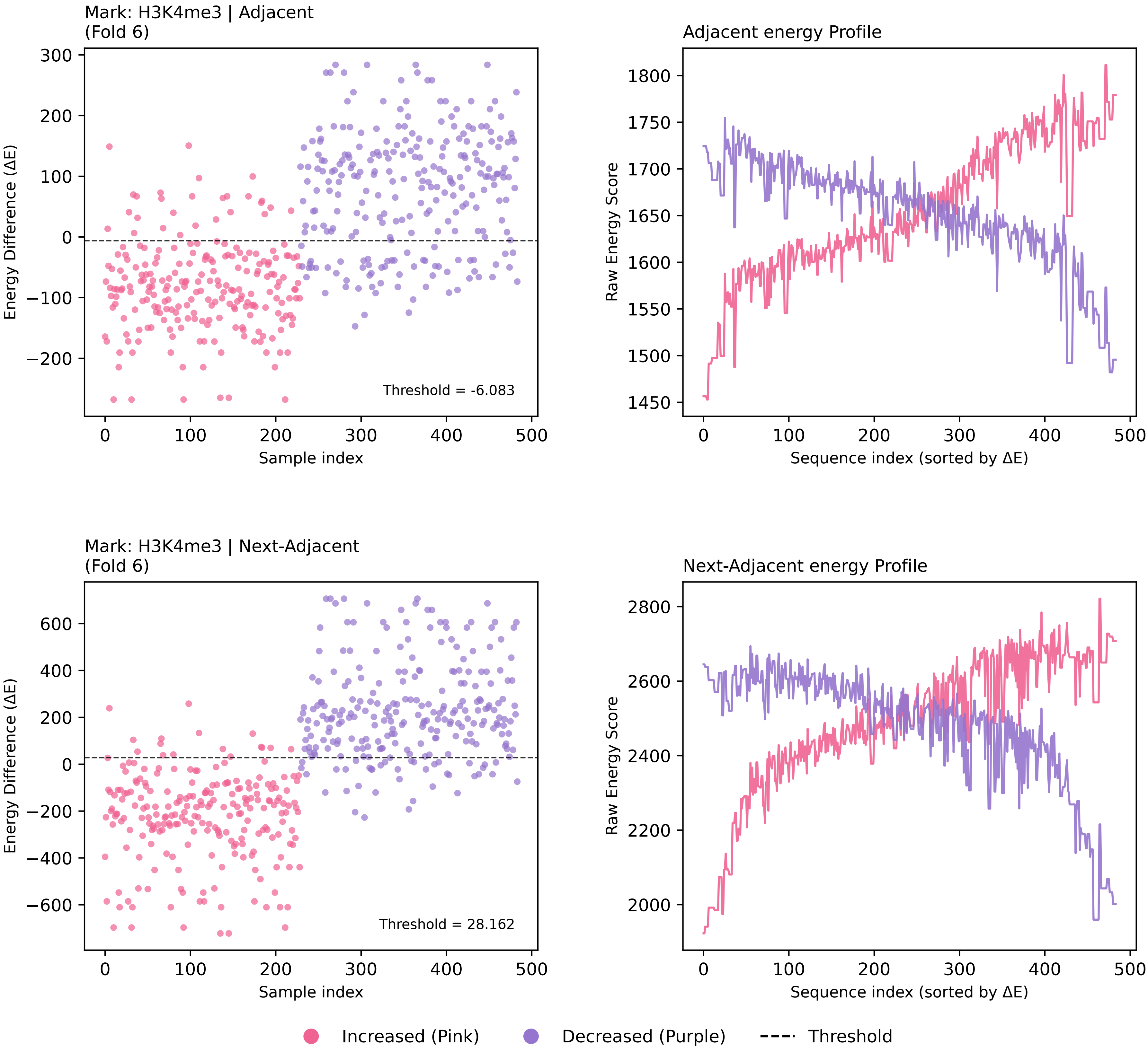

Figure S_Core_Remain_H3K4me3 (Fold 6). Top: Adjacent; Bottom: Next-Adjacent.
Left panels: Scatter plots of energy differences (ΔE); Right panels: Raw energy score profile curves along the sorted sequences.

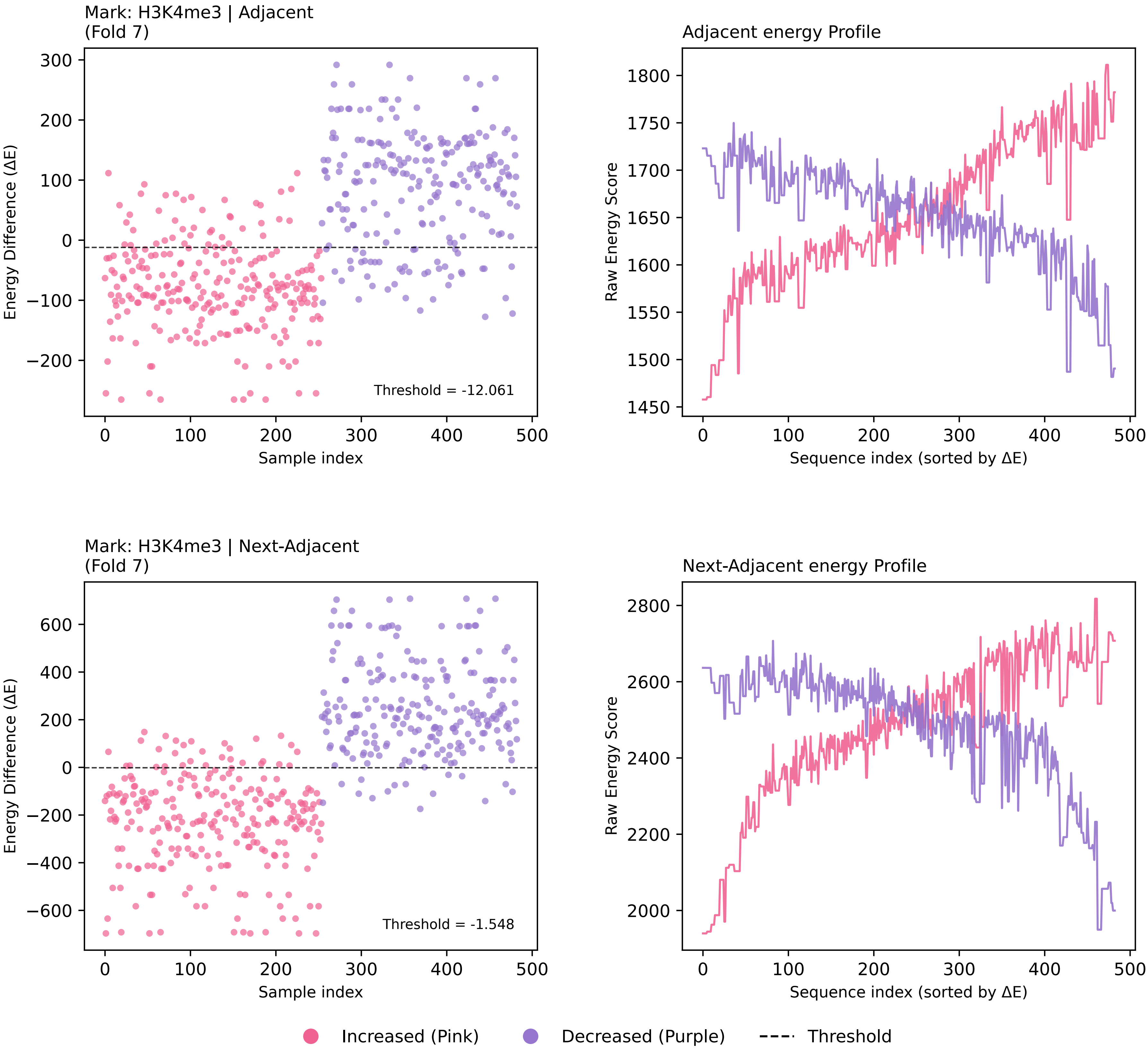

Figure S_Core_Remain_H3K4me3 (Fold 7). Top: Adjacent; Bottom: Next-Adjacent.
Left panels: Scatter plots of energy differences (ΔE); Right panels: Raw energy score profile curves along the sorted sequences.

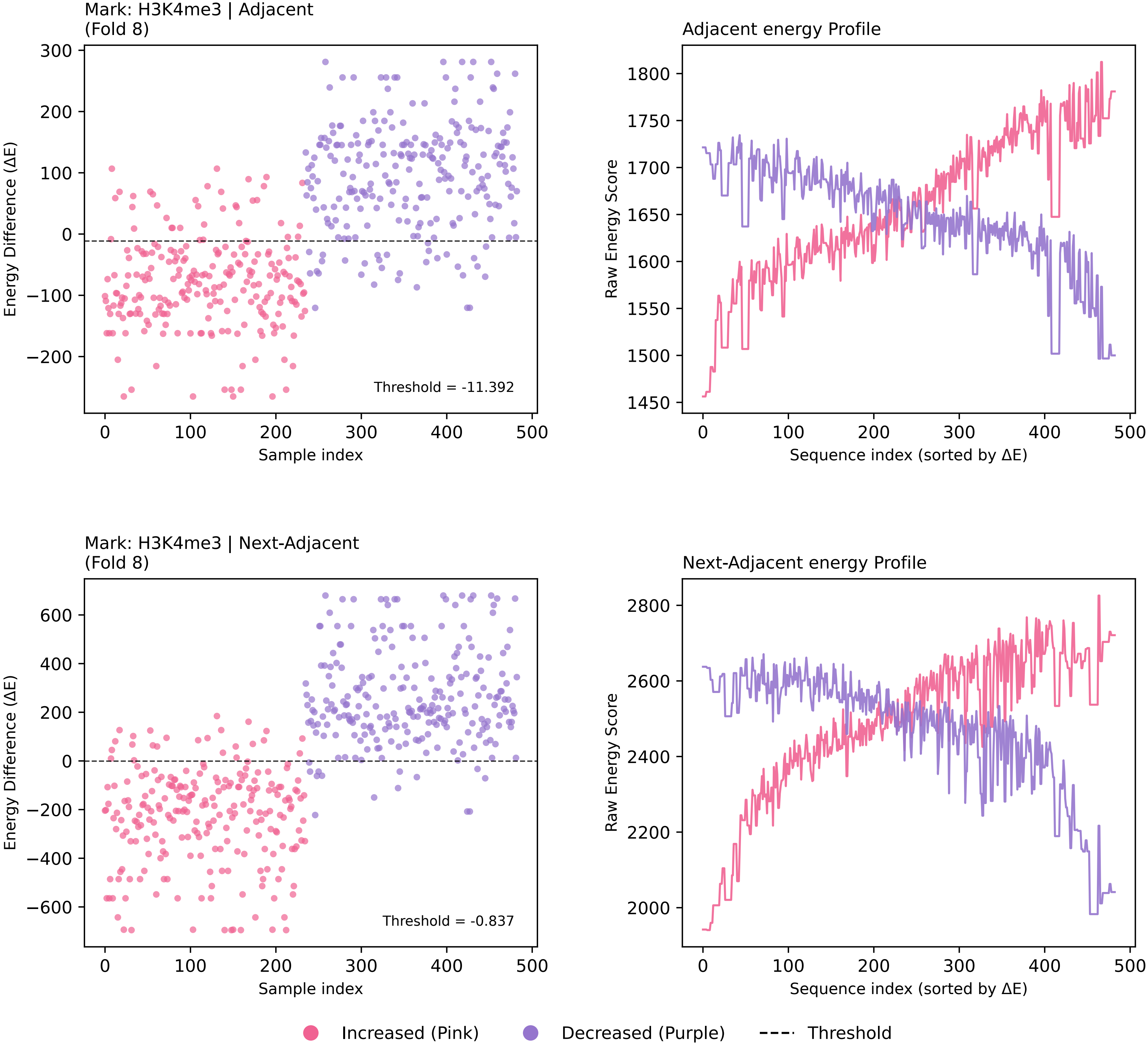

Figure S_Core_Remain_H3K4me3 (Fold 8). Top: Adjacent; Bottom: Next-Adjacent.
Left panels: Scatter plots of energy differences (ΔE); Right panels: Raw energy score profile curves along the sorted sequences.

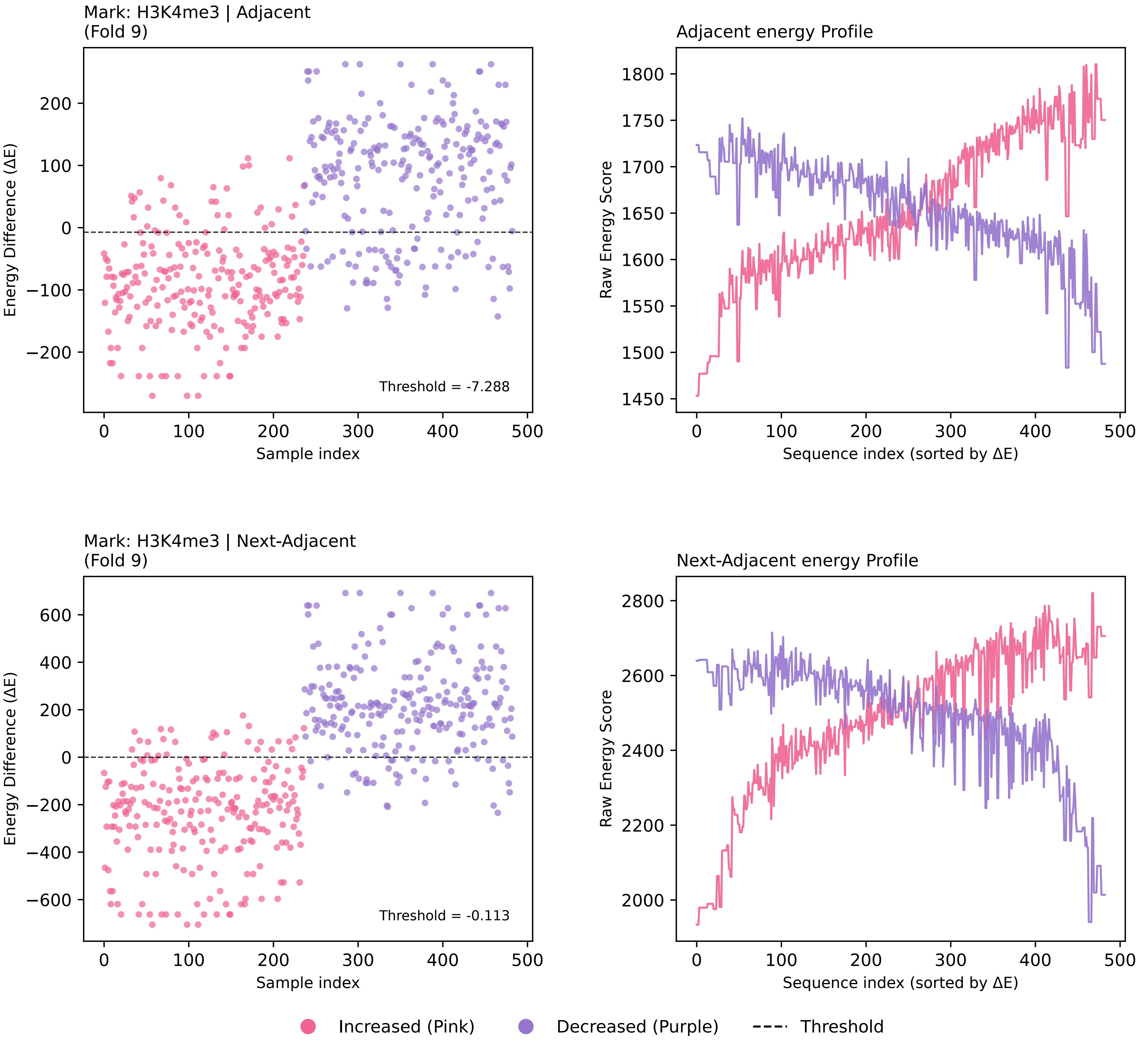

Figure S_Core_Remain_H3K4me3 (Fold 9). Top: Adjacent; Bottom: Next-Adjacent.
Left panels: Scatter plots of energy differences (ΔE); Right panels: Raw energy score profile curves along the sorted sequences.

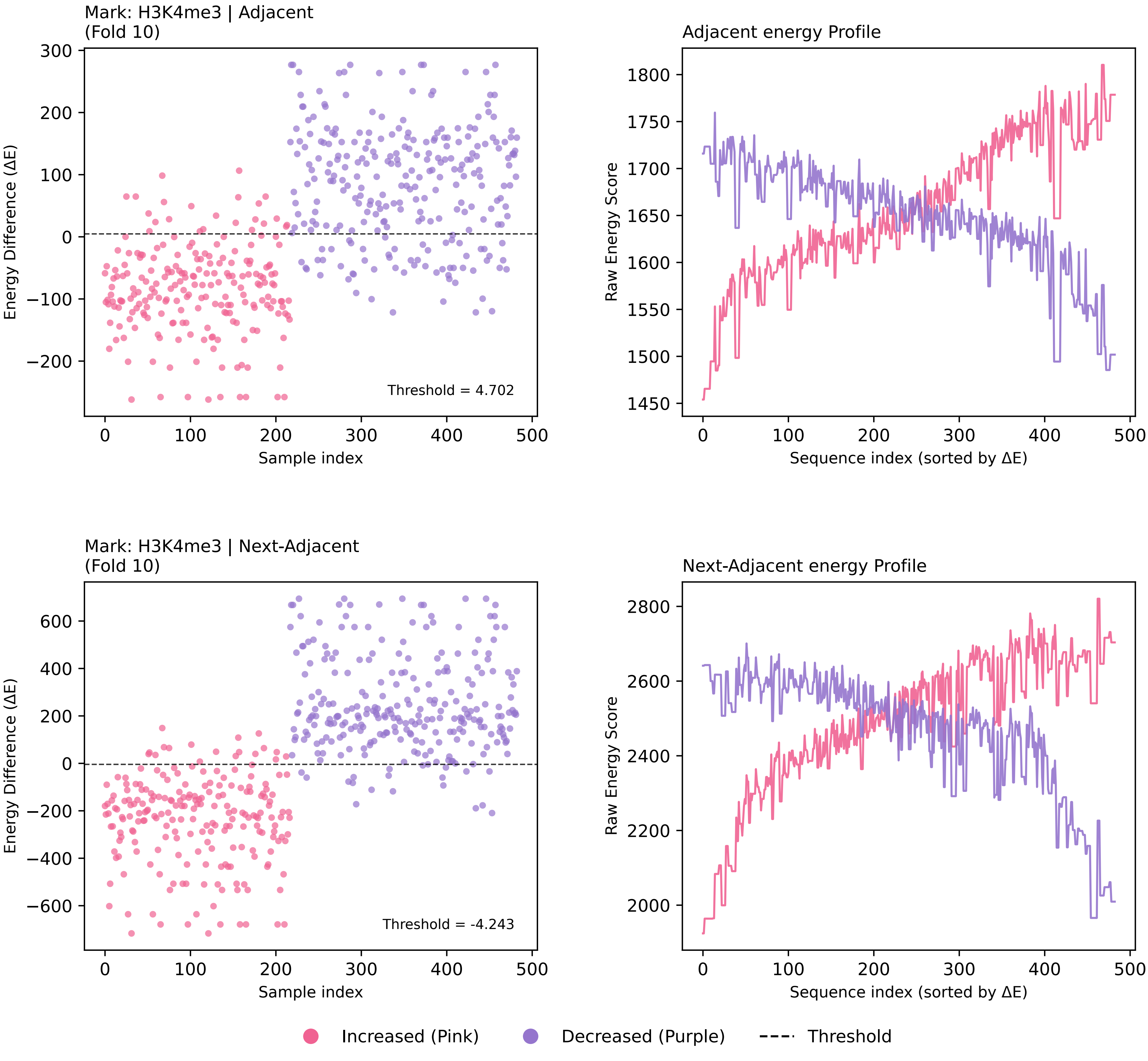

Figure S_Core_Remain_H3K4me3 (Fold 10). Top: Adjacent; Bottom: Next-Adjacent.
Left panels: Scatter plots of energy differences (ΔE); Right panels: Raw energy score profile curves along the sorted sequences.
