# Supplementary material for: An Energy Model Based on Molecular Structure for Predicting Histone Modification Levels at lncRNA Promoter Regions in HepG2 Cells
Source: Int J Mol Sci. 2026 Jun 23;27(13):5653. doi: 10.3390/ijms27135653 (PMC13361589; doi:10.3390/ijms27135653)
Supplement: Supplementary file 1 [file ijms-27-05653-s001.zip › Figure_S9_H3K27ac_Report.pdf]

Performance Metrics: H3K27ac
(Folds 2 to 10)

Table S9_H3K27ac. Supplementary table showing per-fold quantitative metrics for H3K27ac. All values are presented as mean ± confidence

Model	Fold	Sn (%)	Sp (%)	Ac (%)	MCC	auROC
Adjacent	2	85.169	90.0	84.534	0.751	0.952
Adjacent	3	88.053	86.522	88.053	0.746	0.962
Adjacent	4	86.667	88.745	88.889	0.754	0.957
Adjacent	5	81.279	92.827	90.868	0.748	0.95
Adjacent	6	90.254	82.273	83.475	0.729	0.952
Adjacent	7	84.681	92.308	85.745	0.771	0.951
Adjacent	8	87.442	87.137	92.558	0.745	0.95
Adjacent	9	81.557	93.365	81.148	0.749	0.951
Adjacent	10	84.689	86.585	93.301	0.712	0.944
Next-Adjacent	2	91.102	89.091	87.076	0.802	0.98
Next-Adjacent	3	87.168	94.783	91.814	0.822	0.979
Next-Adjacent	4	91.556	91.775	92.889	0.833	0.974
Next-Adjacent	5	91.324	89.03	93.836	0.803	0.979
Next-Adjacent	6	92.797	87.727	87.288	0.807	0.978
Next-Adjacent	7	88.936	93.213	88.298	0.821	0.971
Next-Adjacent	8	86.512	92.531	95.116	0.794	0.973
Next-Adjacent	9	88.115	90.995	83.402	0.789	0.973
Next-Adjacent	10	90.909	89.024	97.847	0.798	0.975

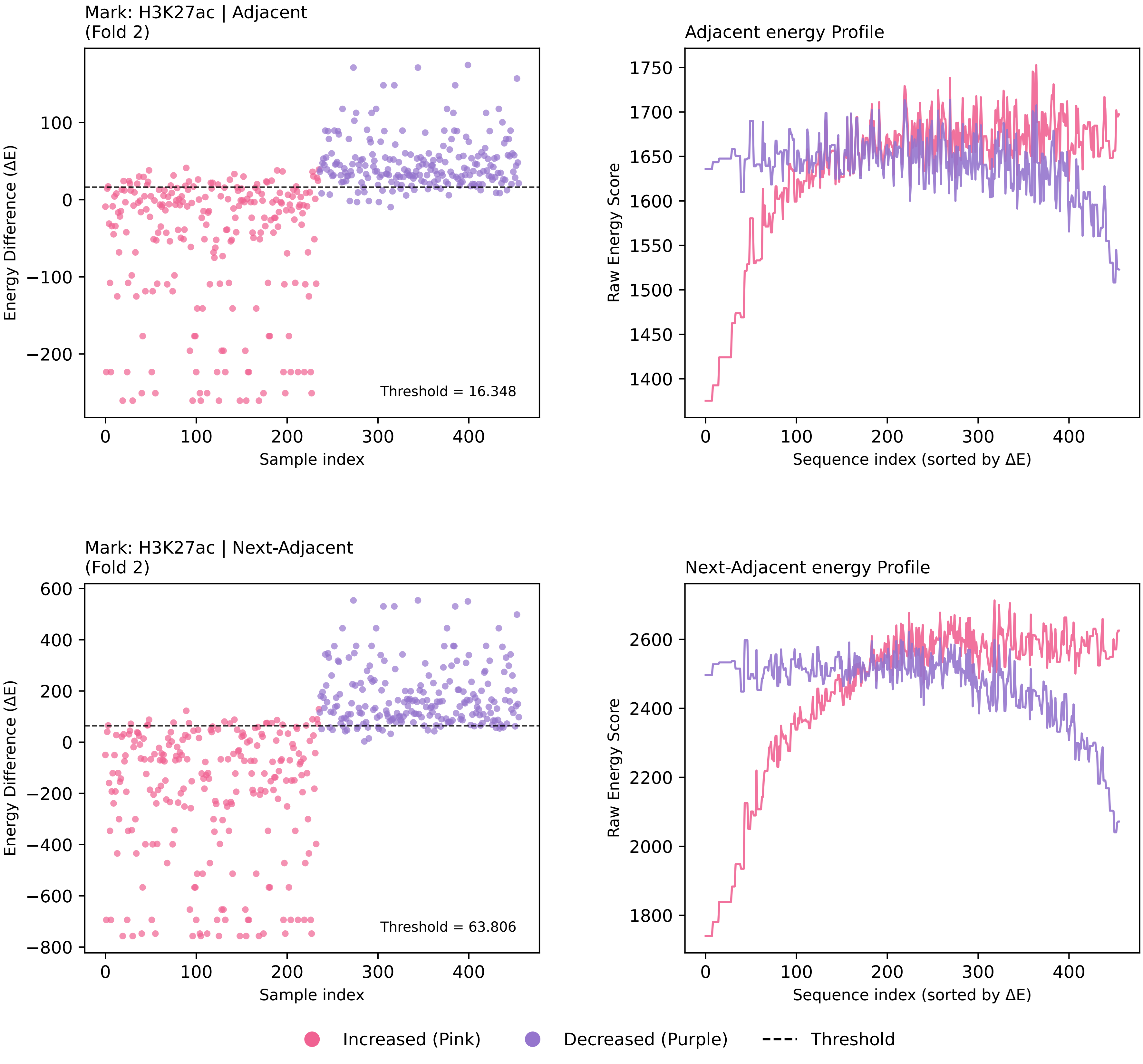

Figure S_Core_Remain_H3K27ac (Fold 2). Top: Adjacent; Bottom: Next-Adjacent.
Left panels: Scatter plots of energy differences (ΔE); Right panels: Raw energy score profile curves along the sorted sequences.

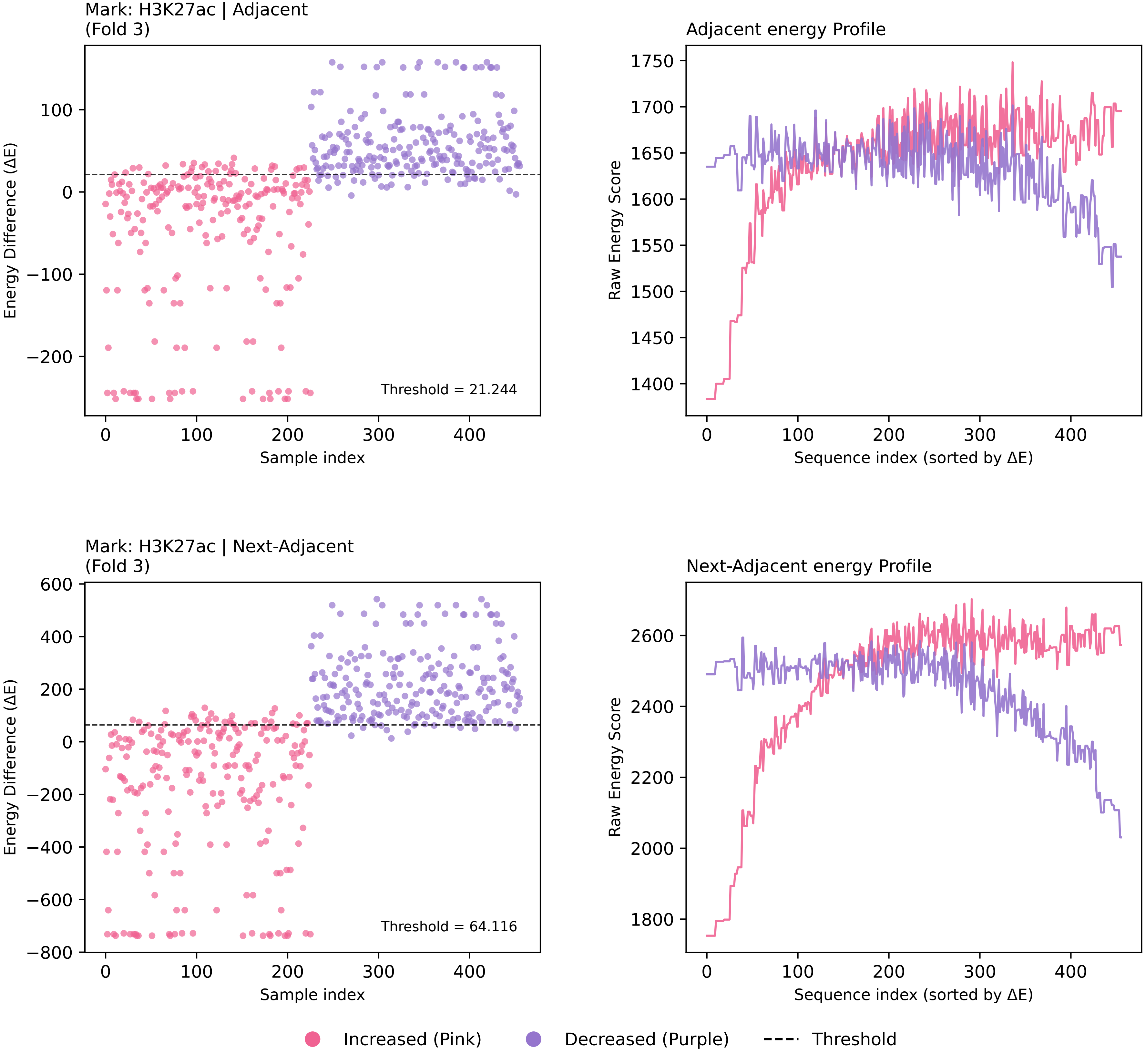

Figure S_Core_Remain_H3K27ac (Fold 3). Top: Adjacent; Bottom: Next-Adjacent.
Left panels: Scatter plots of energy differences (ΔE); Right panels: Raw energy score profile curves along the sorted sequences.

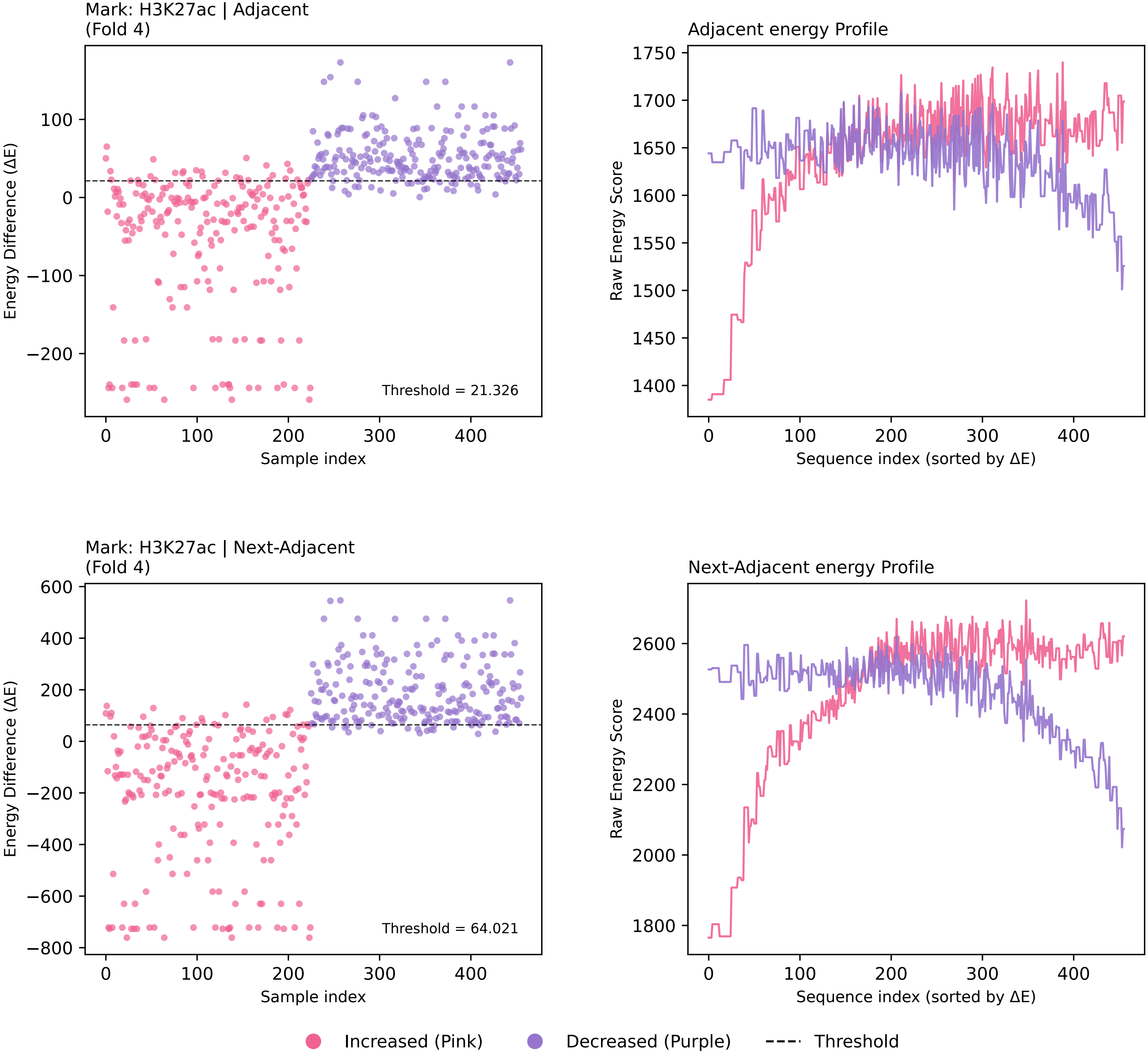

Figure S_Core_Remain_H3K27ac (Fold 4). Top: Adjacent; Bottom: Next-Adjacent.
Left panels: Scatter plots of energy differences (ΔE); Right panels: Raw energy score profile curves along the sorted sequences.

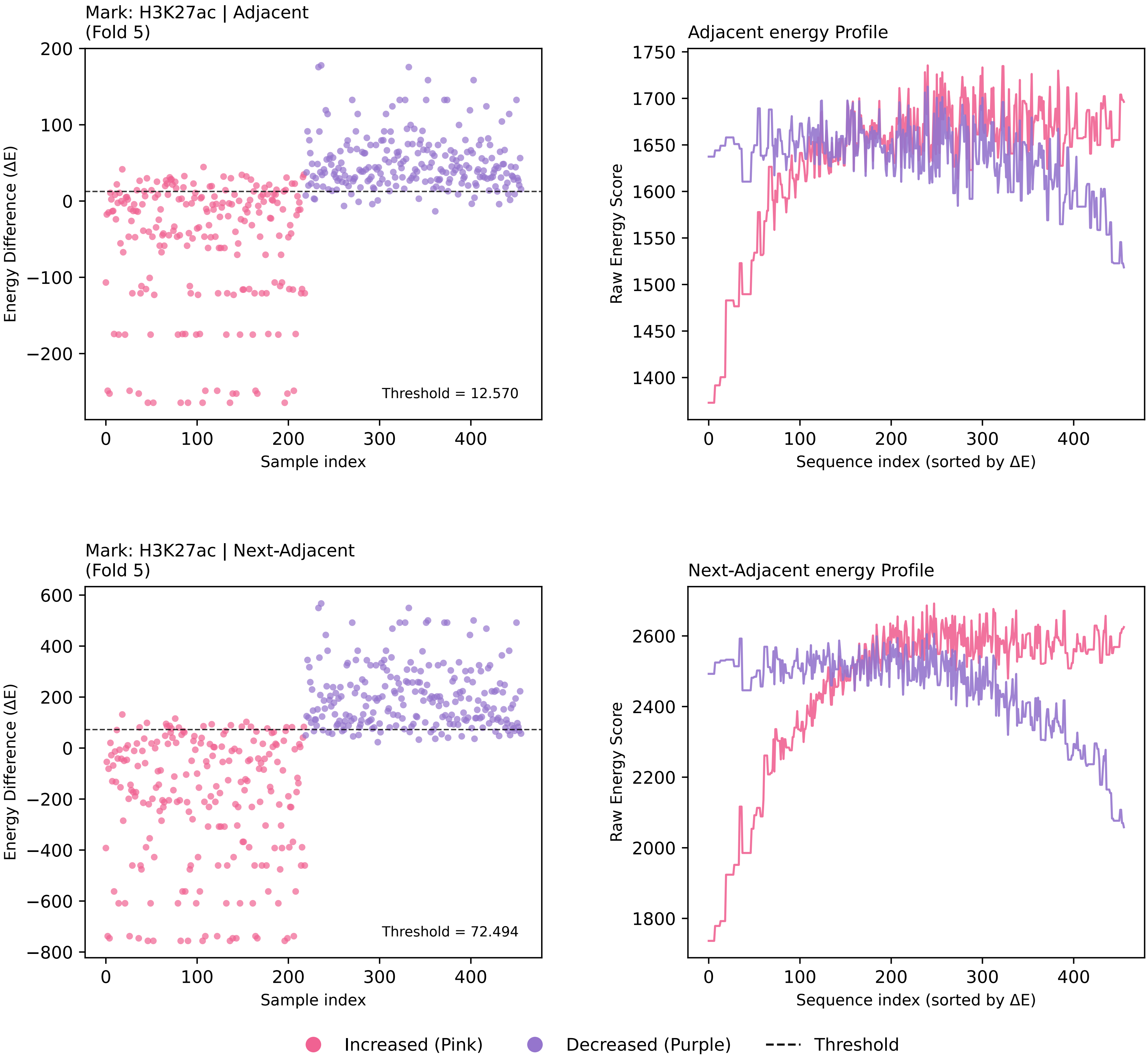

Figure S_Core_Remain_H3K27ac (Fold 5). Top: Adjacent; Bottom: Next-Adjacent.
Left panels: Scatter plots of energy differences (ΔE); Right panels: Raw energy score profile curves along the sorted sequences.

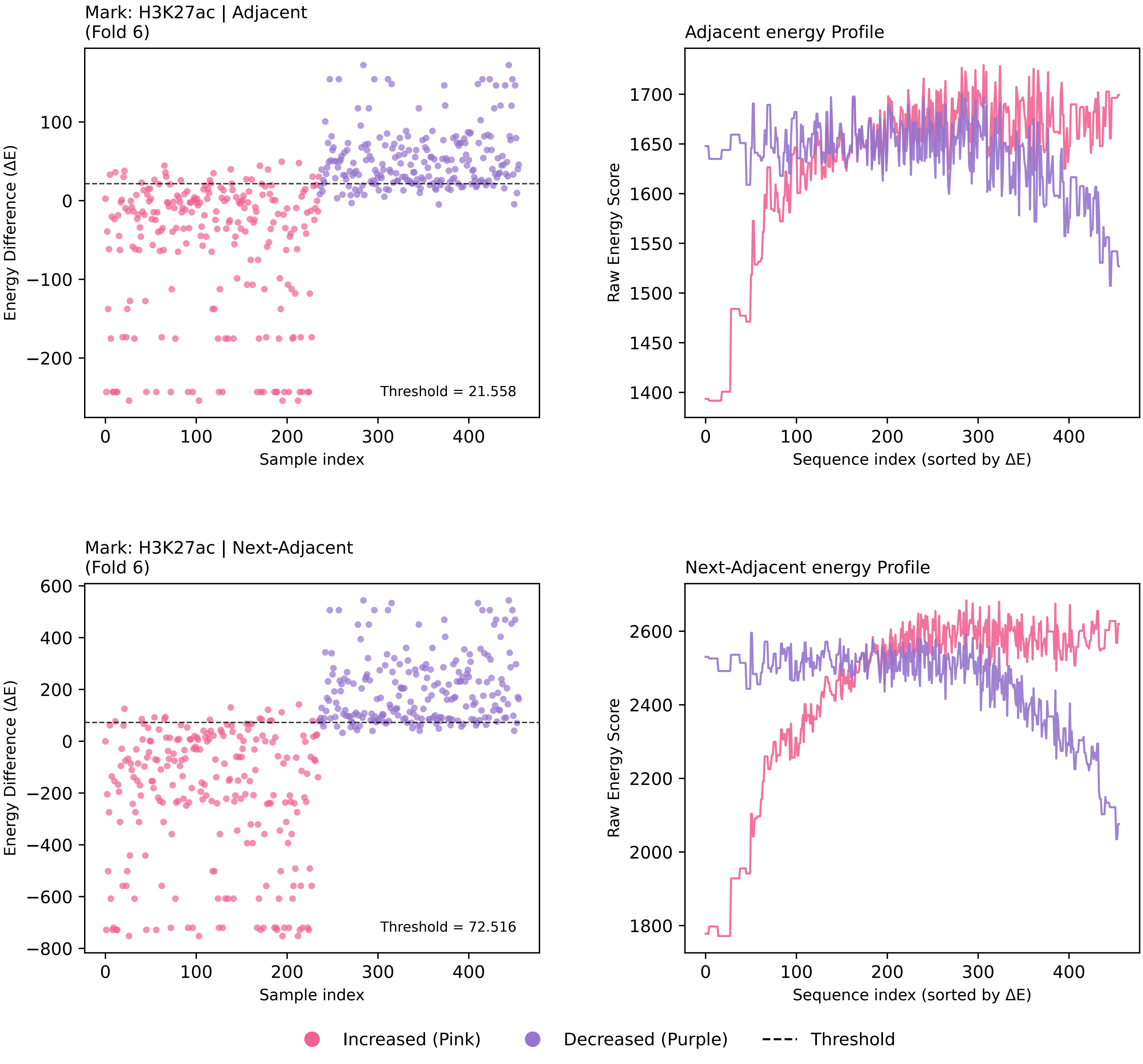

Figure S_Core_Remain_H3K27ac (Fold 6). Top: Adjacent; Bottom: Next-Adjacent.
Left panels: Scatter plots of energy differences (ΔE); Right panels: Raw energy score profile curves along the sorted sequences.

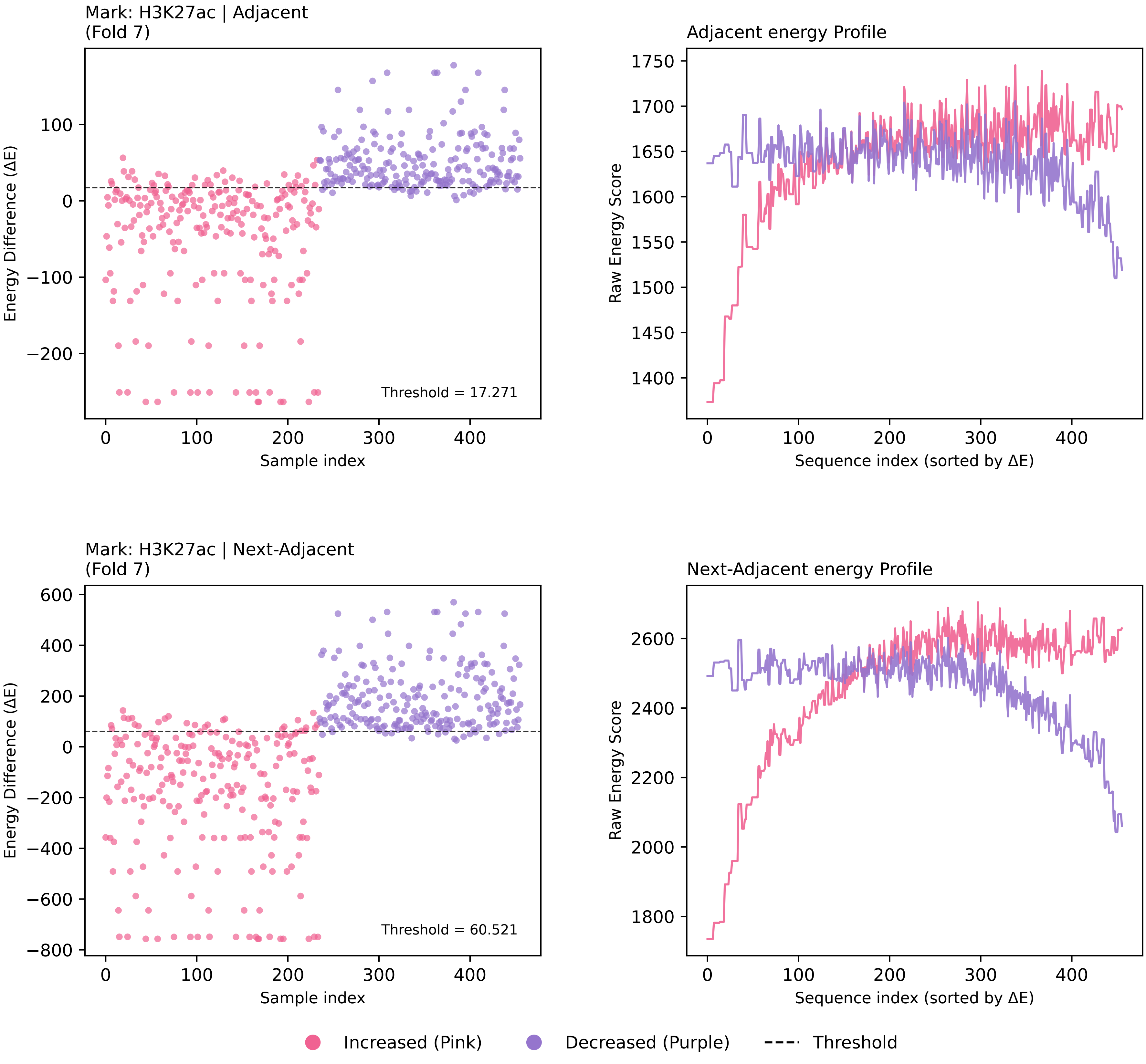

Figure S_Core_Remain_H3K27ac (Fold 7). Top: Adjacent; Bottom: Next-Adjacent.
Left panels: Scatter plots of energy differences (ΔE); Right panels: Raw energy score profile curves along the sorted sequences.

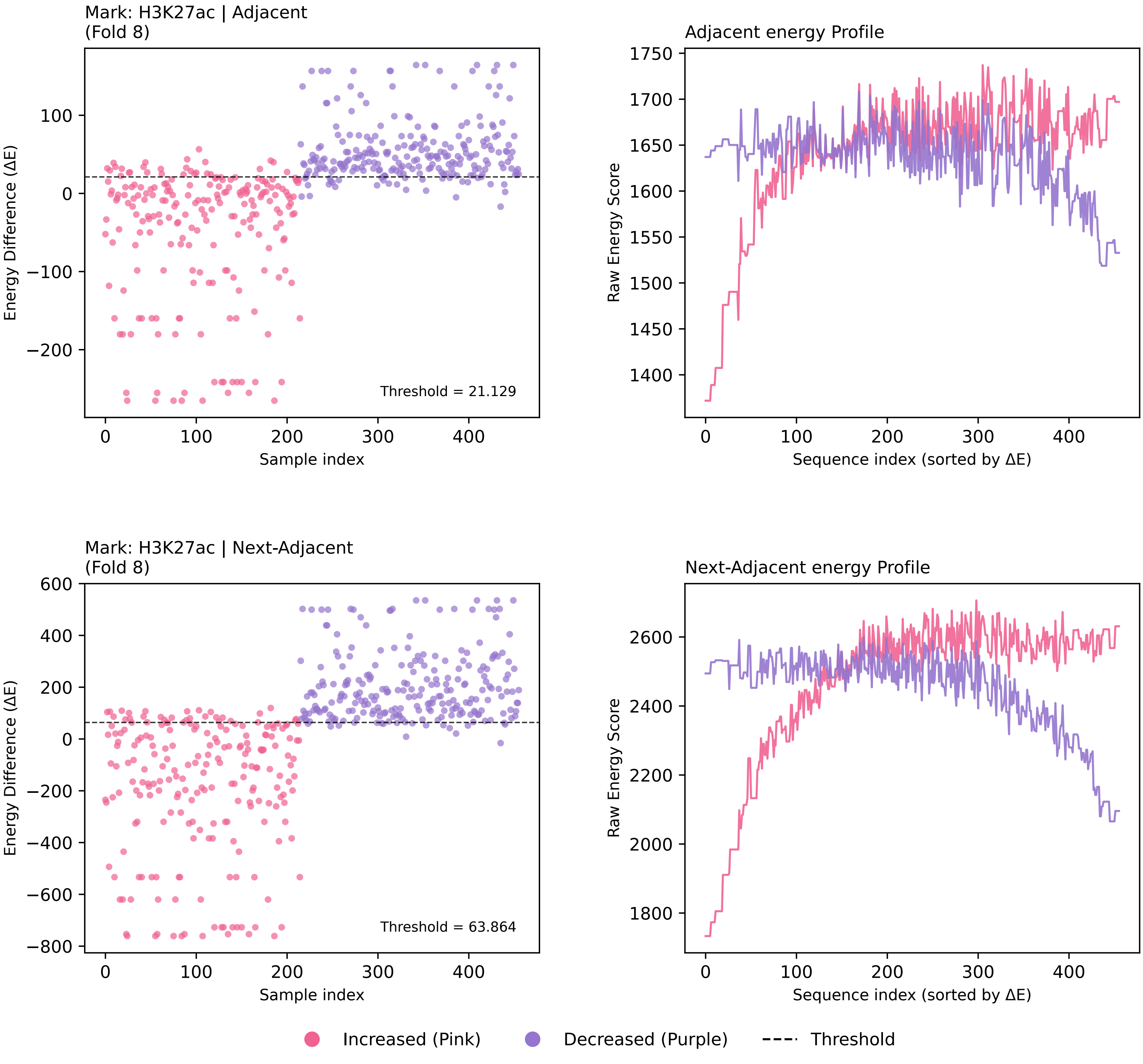

Figure S_Core_Remain_H3K27ac (Fold 8). Top: Adjacent; Bottom: Next-Adjacent.
Left panels: Scatter plots of energy differences (ΔE); Right panels: Raw energy score profile curves along the sorted sequences.

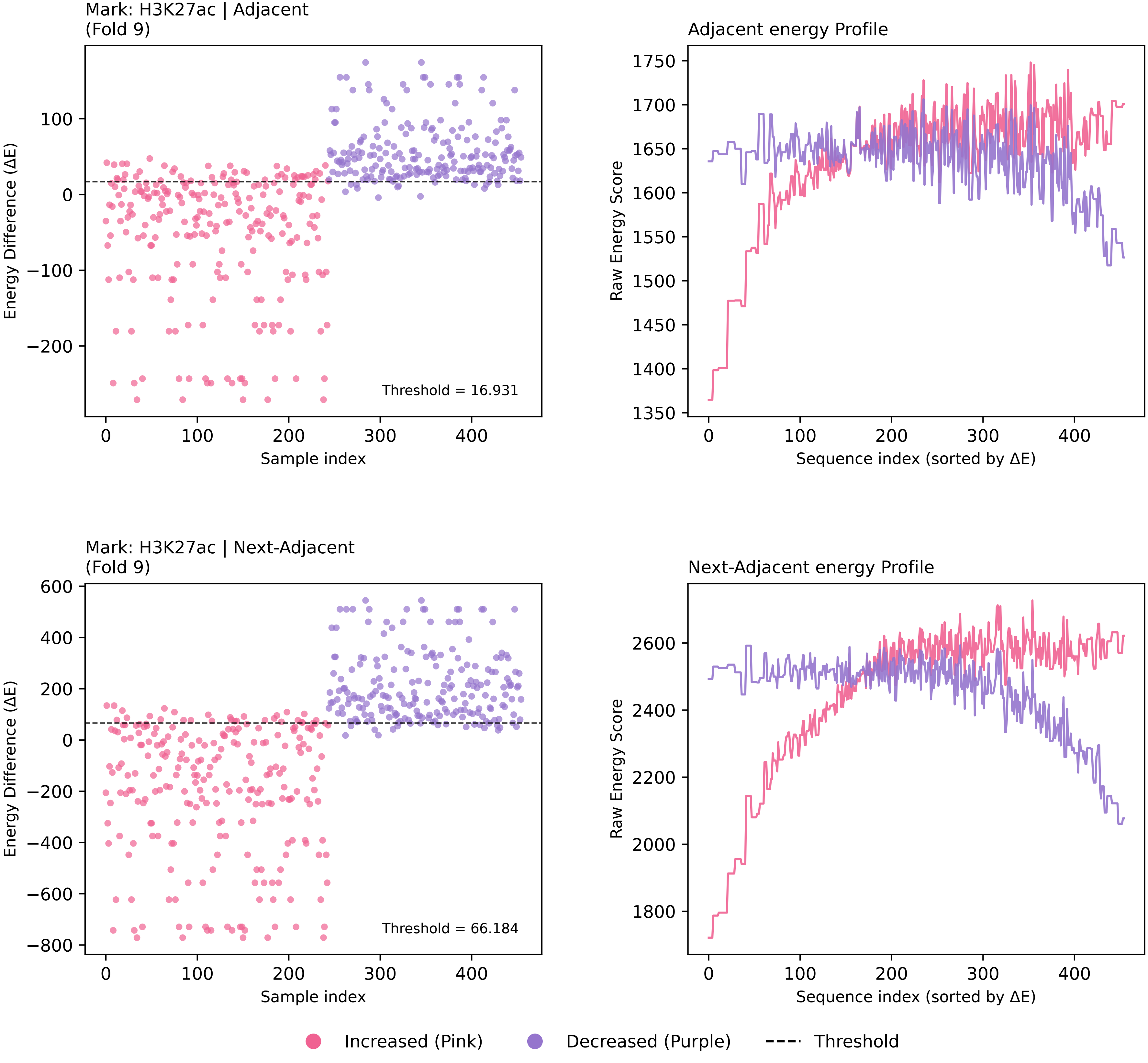

Figure S_Core_Remain_H3K27ac (Fold 9). Top: Adjacent; Bottom: Next-Adjacent.
Left panels: Scatter plots of energy differences (ΔE); Right panels: Raw energy score profile curves along the sorted sequences.

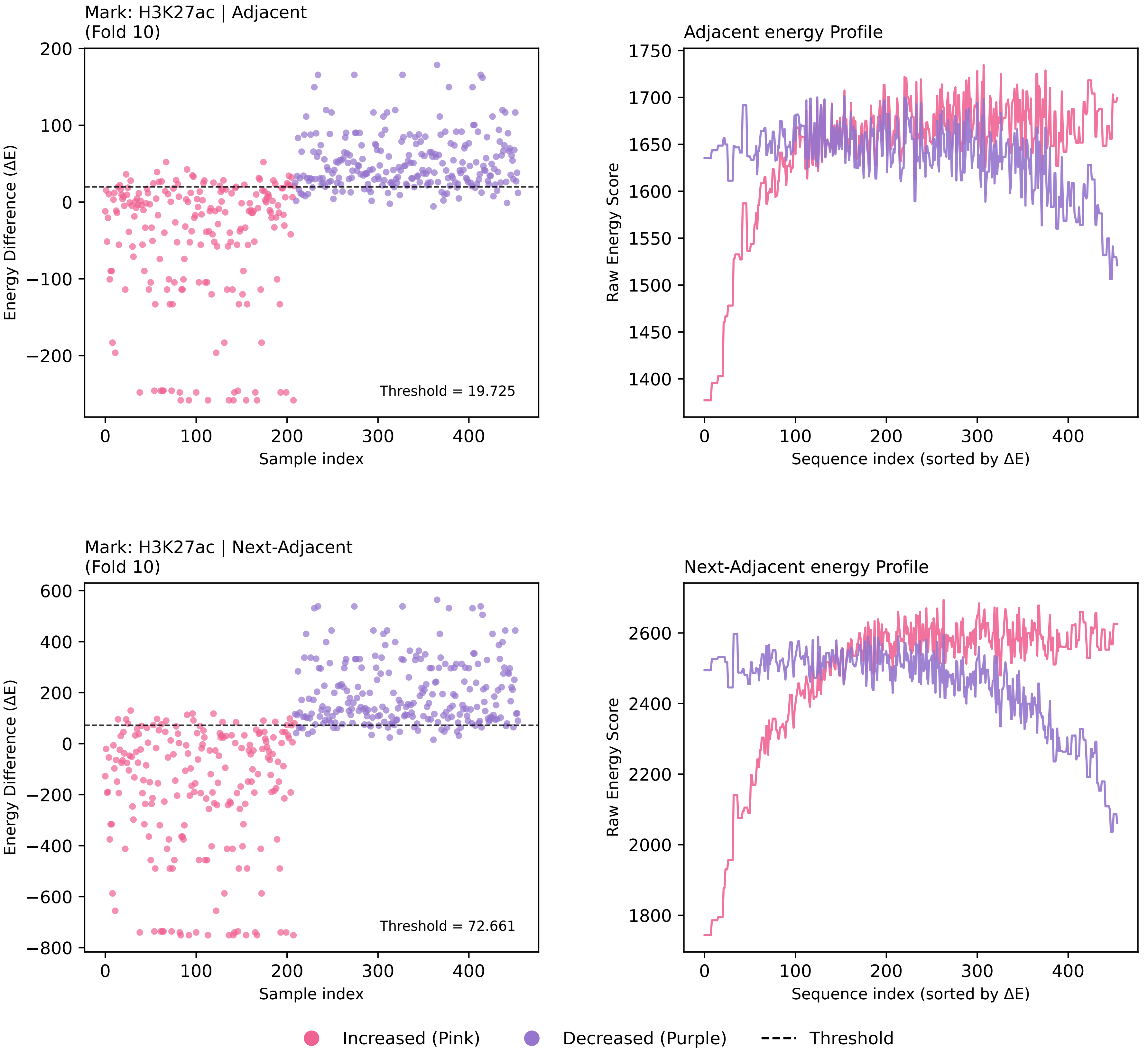

Figure S_Core_Remain_H3K27ac (Fold 10). Top: Adjacent; Bottom: Next-Adjacent.
Left panels: Scatter plots of energy differences (ΔE); Right panels: Raw energy score profile curves along the sorted sequences.
