# Supplementary material for: An Energy Model Based on Molecular Structure for Predicting Histone Modification Levels at lncRNA Promoter Regions in HepG2 Cells
Source: Int J Mol Sci. 2026 Jun 23;27(13):5653. doi: 10.3390/ijms27135653 (PMC13361589; doi:10.3390/ijms27135653)
Supplement: Supplementary file 1 [file ijms-27-05653-s001.zip › Table_S1.pdf]

Table S1. Comprehensive list of ENCODE datasets utilized in this study (GRCh38).

Part A: ChIP-seq Histone Modification Datasets (11 Marks)

Histone Mark	Cancer (HepG2) Peak	Cancer (HepG2) BigWig	Normal (Hepatocyte) Peak	Normal (Hepatocyte) BigWig
**H3K9ac**	ENCFF219XNL	ENCFF192NNA	ENCFF801YHR	ENCFF990IOY
**H3K79me2**	ENCFF500KVU	ENCFF764DUI	ENCFF503CCA	ENCFF233VCR
**H3K27ac**	ENCFF580KMC	ENCFF493VUL	ENCFF198IPN	ENCFF217WGE
**H3K4me2**	ENCFF800GTZ	ENCFF767KKA	ENCFF007NSA	ENCFF079JTD
**H3K36me3**	ENCFF489ZNJ	ENCFF094ZKB	ENCFF980QVG	ENCFF360BQU
**H3K4me1**	ENCFF424GUI	ENCFF554XSR	ENCFF280MMK	ENCFF357ZXR
**H4K20me1**	ENCFF942YUJ	ENCFF449LNO	ENCFF531JMX	ENCFF604UAY
**H2AFZ**	ENCFF303VCJ	ENCFF747JGO	ENCFF912CDH	ENCFF243ZML
**H3K27me3**	ENCFF853KUB	ENCFF942QHN	ENCFF374HIV	ENCFF034ZEQ
**H3K4me3**	ENCFF040IFA	ENCFF219ZOU	ENCFF646JEB	ENCFF510IFZ
**H3K9me3**	ENCFF745LNA	ENCFF125NHB	ENCFF503GSL	ENCFF305KLP

(Note: Peak files are in .bed.gz format; BigWig files are in .bw format representing SignalPval tracks.)

Part B: lncRNA Expression RNA-seq Datasets

Cell Line	Condition	Rep1 (expected_count.tsv)	Rep2 (expected_count.tsv)
**HepG2**	Cancer	ENCFF826QFM	ENCFF944RFV
**Hepatocytes**	Normal	ENCFF072XSA	ENCFF491FPY
